# Enantioselective Total Synthesis of (−)‐Limaspermidine and (−)‐Kopsinine by a Nitroaryl Transfer Cascade Strategy

**DOI:** 10.1002/anie.202210592

**Published:** 2022-09-12

**Authors:** Brendan Horst, Daniël S. Verdoorn, Sven Hennig, Gydo van der Heijden, Eelco Ruijter

**Affiliations:** ^1^ Department of Chemistry and Pharmaceutical Sciences Amsterdam Institute of Molecular and Life Sciences (AIMMS) De Boelelaan 1108 1081 HZ Amsterdam The Netherlands

**Keywords:** Alkaloids, Asymmetric Synthesis, Domino Reactions, Total Synthesis

## Abstract

We report an intramolecular conjugate addition/Truce‐Smiles/E1cb cascade of 2‐nitrobenzenesulfonamide‐functionalized cyclohexenones as a new entry to the core scaffold of monoterpene indole alkaloids. The method was applied to the asymmetric total synthesis of (−)‐limaspermidine, (−)‐kopsinilam, and (−)‐kopsinine, as well as the framework of the kopsifoline alkaloids, thus highlighting its complementarity to existing approaches involving the use of indole‐based starting materials or the interrupted Fischer indole synthesis. Furthermore, we show that the cascade tolerates various substituents on the nitroarene, opening the way to other natural products as well as non‐natural analogues.

## Graphical abstract

2‐Nitrobenzenesulfonamides can be exploited as functional protective groups by their use in a long‐range nitroarene transfer cascade reaction. Using a suitably functionalized precursor, this process was used as the key step in the enantioselective total synthesis of the monoterpene indole alkaloids (−)‐limaspermidine, (−)‐kopsinilam, and (−)‐kopsinine as well as the unnatural alkaloid (−)‐tetra‐hydrokopsifoline D.

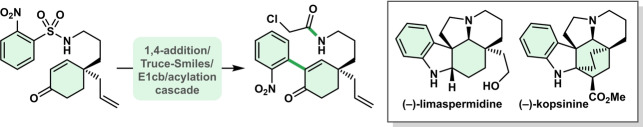

Monoterpene indole alkaloids (MIAs) constitute a very large class of natural products isolated from a wide range of flowering plants, most notably from the *Kopsia*, *Aspidosperma*, and *Strychnos* genera (Figure 1). MIAs often feature complex polycyclic molecular architectures as well as potent and diverse bioactivity, making them popular targets for total synthesis, dating back to Woodward's landmark synthesis of (±)‐strychnine.^[1]^ (+)‐Limaspermidine (**2**) features the typical framework of *Aspidosperma* alkaloids which curiously have the opposite absolute configuration of the pentacyclic core compared to *Strychnos* and *Kopsia* alkaloids. Limaspermidine was first isolated in 1979 from *A. rhombeosignatum* by Di Genova,^[2]^ but was already unwittingly synthesized by Ban in 1976 in their efforts towards aspidofractinine.^[3]^ Since then, numerous elegant strategies have been reported.^[4]^ (−)‐Kopsinine (**3**), first isolated in 1954 from *K. longiflora* by Michael, has demonstrated high antitussive properties.^[5]^ Its complex cage‐like structure is characteristic for the *Kopsia* alkaloids. Although the first enantioselective synthesis by Magnus dates back to 1985,^[6]^ its synthesis gained renewed interest in the past decade.^[7]^ The kopsifolines^[8]^ (including (−)‐kopsifoline D, **4**) feature an alternatively bridged framework.^[9]^

**Figure 1:**
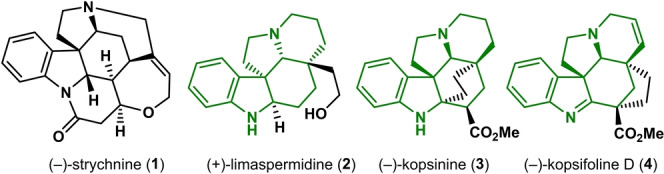
Selected Apocynaceae alkaloids, with the characteristic pentacyclic scaffold of the *Kopsia* and *Aspidosperma* alkaloids shown in green.

Biosynthetically, all MIAs are ultimately derived from strictosidine, the Pictet–Spengler product of tryptamine and the terpenoid aldehyde secologanin.^[10]^ It is therefore hardly surprising that most synthetic approaches toward MIAs employ tryptamine (or a related indole derivative) as a starting point, often featuring a dearomative spirocyclization of the indole moiety as a key step.^[11]^

However, the intrinsic nucleophilicity of indoles may be incompatible with other key steps in the synthesis, leading to the development of various methods for the late‐stage introduction of the indole or indoline moiety, including the (interrupted) Fischer indole synthesis and the reductive cyclization of nitroarenes. Although the former approach presents a direct and frequently efficient method for late‐stage indolization,^[12]^ it may suffer from regioselectivity issues or competing indole formation. The reductive cyclization of nitroarenes presents an interesting alternative to Fischer indolization.[^4g^, ^13^] However, also in this approach, the regioselective introduction of the nitroaryl moiety is far from trivial. Given our interest in the synthesis of indole alkaloids^[14]^ and related compounds,^[15]^ we became interested in new methods for the regioselective introduction of nitroaryl moieties to provide controlled access to polycyclic indole and indolenine frameworks.

In 2016, Canesi and co‐workers reported an intriguing cascade reaction of tyramine‐derived cyclohexadienones **5** bearing a 2‐nitrobenzenesulfonamide moiety (Scheme 1a).^[16]^ This domino process involves conjugate addition of the sulfonamide moiety to the enone and subsequent Truce‐Smiles rearrangement of the resulting enolate to give nitrobenzyl ketone **7**, which rapidly undergoes proton transfer, E1cb elimination, and conjugate addition on the opposite side of the ring to afford (racemic) tetrahydroindole derivatives **10**. The latter reaction can be considered undesirable, as it prevents further elaboration of the cascade products to MIAs. We envisioned that the use of enone **11** as an alternative substrate would allow the cascade to be interrupted at the stage of aza‐decalin **12** or the ring‐opened product **13** (Scheme 1b). Moreover, the use of a chiral substrate in the key cascade reaction would allow control over the absolute stereochemistry in a unified approach to *Kopsia* and *Aspidosperma* alkaloids.

**Scheme 1:**
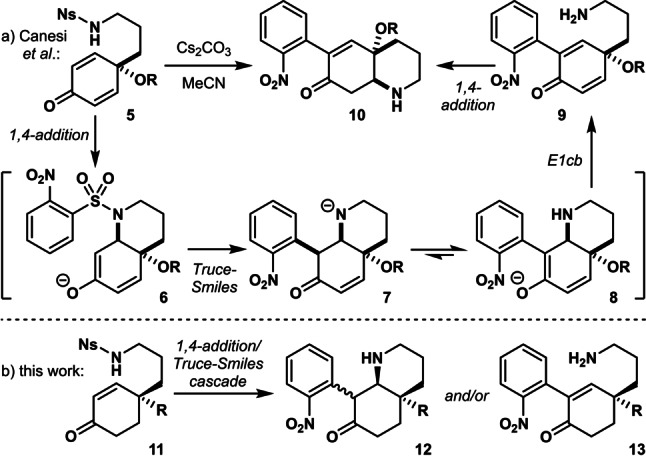
Conjugate addition/Truce‐Smiles cascade reaction.

Pentacycle **14** was considered as a key common intermediate in our retrosynthetic approach (Scheme 2). Construction of the final **B** and **E** rings was projected by reduction/condensation of the cascade product **15**, followed by nucleophilic attack of the indole C3 position on the tethered chloroacetamide. Similar cyclization sequences have shown to be fully diastereoselective, making the formation of the C4 stereocenter critical in our enantioselective synthesis.[^4b^, ^4e^, ^17^] Instalment of this all‐carbon quaternary stereocenter was envisioned by Pd‐catalyzed enantioselective decarboxylative allylation as pioneered by Stoltz.^[18]^ The resulting product **17** would be readily converted to the desired cascade precursor **16** by reduction, nosylation and Stork‐Danheiser transposition.^[19]^ In turn, ketoester **18** can be readily derived from 1,3‐cyclohexadione (**19**).

**Scheme 2:**
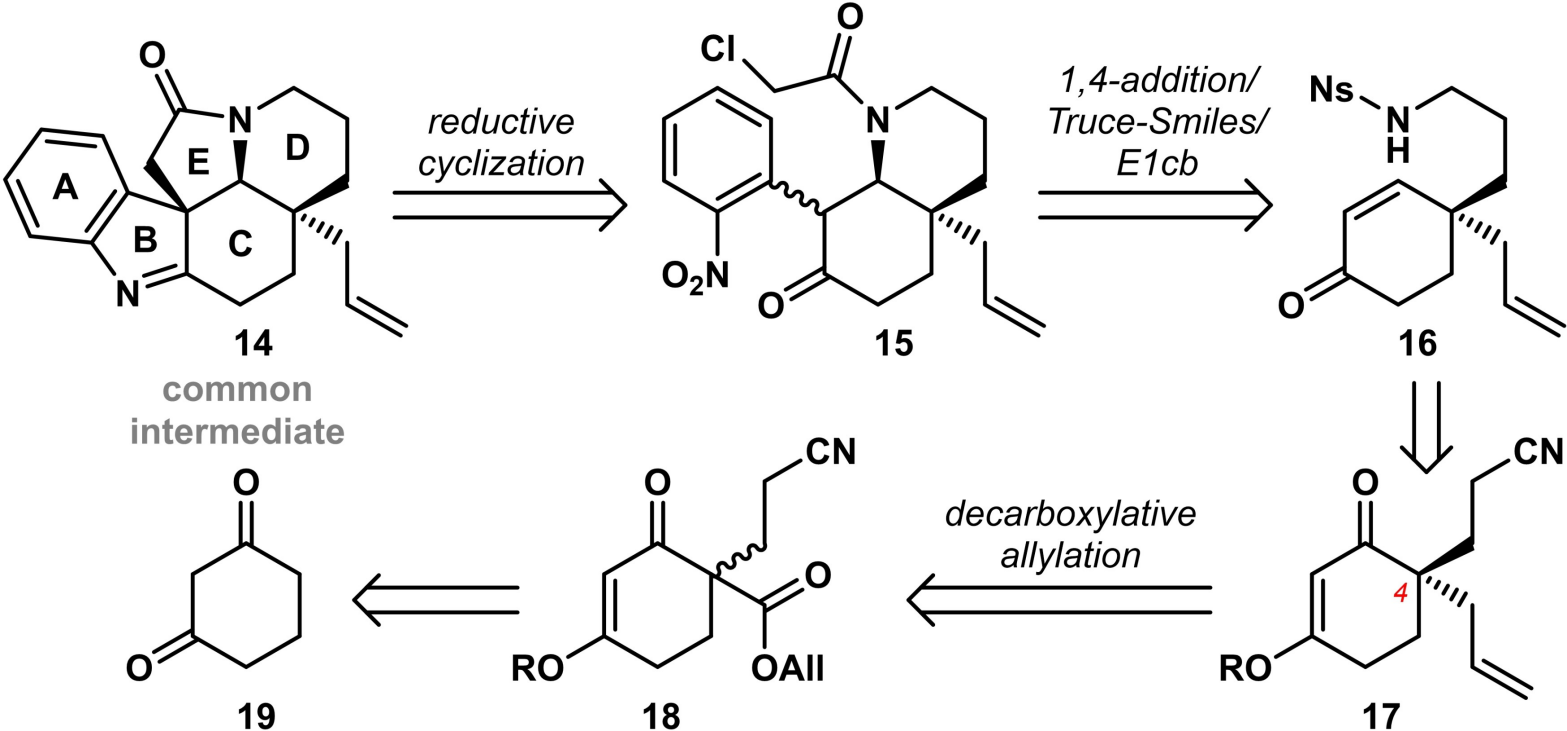
Retrosynthetic analysis of *Kopsia* and *Aspidosperma* alkaloids by a nitroaryl transfer cascade strategy. Ns=2‐nitrobenzenesulfonyl, All=allyl.

We started our synthetic approach with the protection of **19** under Dean–Stark conditions to provide the enol ether in near quantitative yield (Scheme 3). Acylation with allyl chloroformate, followed by Michael addition of the resulting ketoester to acrylonitrile furnished **18** in 78 % yield over three steps. Deracemization was accomplished by a palladium‐catalyzed decarboxylative allylation.^[18]^ A brief optimization (Supporting Information Table S1) revealed the optimal conditions [2 mol% Pd(dba)_2_, 4 mol% (*S*)‐*t*‐BuPHOX (**L3**), Et_2_O, RT] furnishing **17** in 85 % yield with 91 % *ee*, even on >15 g scale. Conversion of optically enriched **17** into cascade precursor **16** proved to be challenging: simultaneous reduction of the ketone and nitrile moieties with LiAlH_4_ resulted in the undesired 1,4‐reduction of the α,β‐unsaturated ketone, contrary to earlier reports.^[17a]^ Instead, DIBAL‐H proved suitable for selective 1,2‐reduction of the ketone moiety. Unfortunately, this procedure did not allow simultaneous reduction of the nitrile group. Thus, reduction of the ketone with DIBAL‐H followed by reduction of the nitrile with LiAlH_4_ gave us the desired alcohol and amine functionalities in product **20**. Immediate nosylation of the amine with nosyl chloride under basic conditions and hydrolysis of the β‐hydroxyenol ether with aqueous HCl gave the cascade precursor **16** in 69 % yield.

**Scheme 3:**
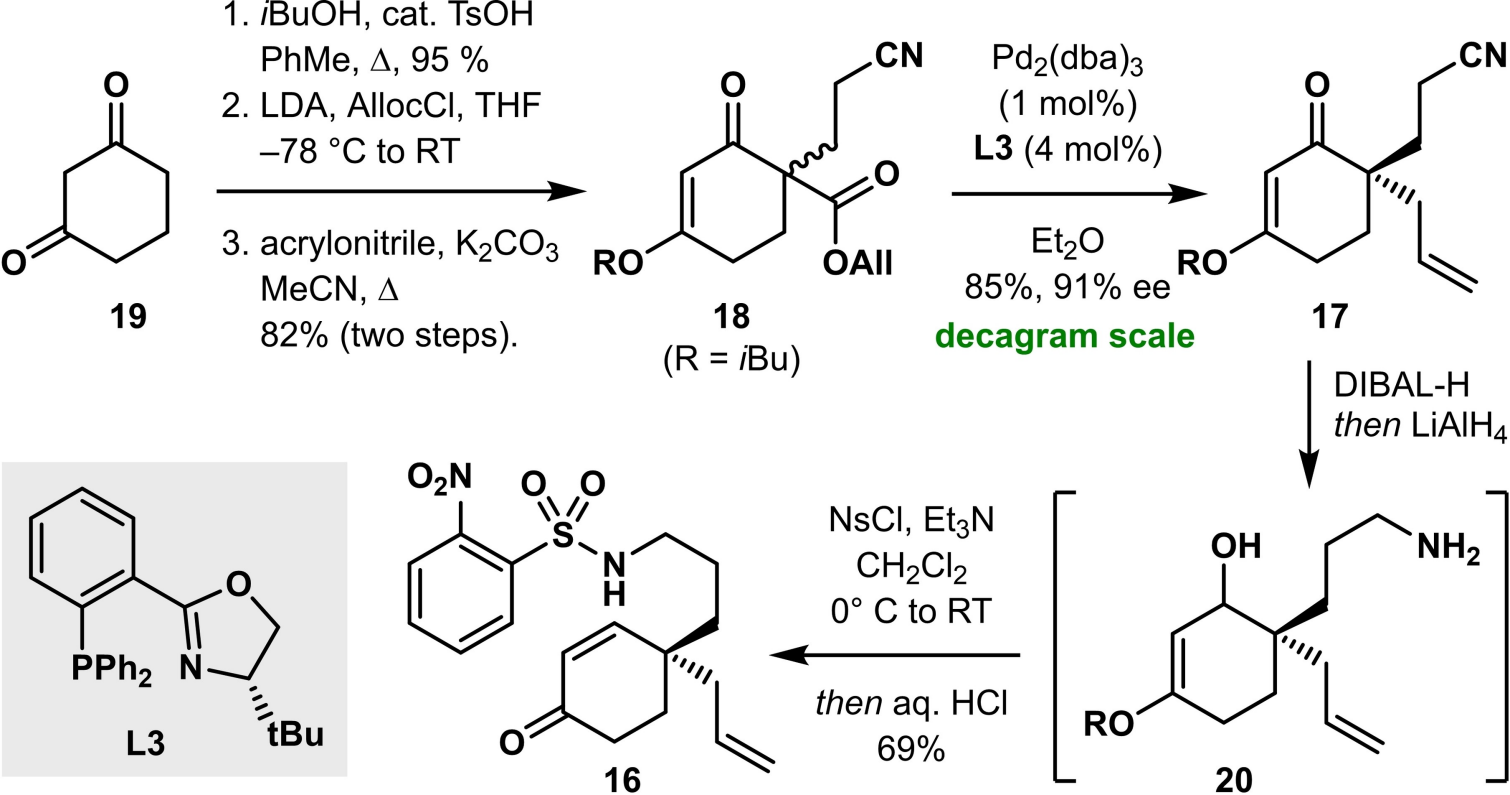
Synthesis of cascade precursor **16**. THF=tetrahydrofuran, dba=dibenzylideneacetone, Alloc=allyloxycarbonyl, DIBAL‐H=di‐isobutyl‐aluminum hydride.

With the cascade substrate in hand, we subjected it to the conditions reported by Canesi.^[16]^ Disappointingly, refluxing **16** in acetonitrile in the presence of Cs_2_CO_3_ gave full conversion to a range of unisolable products. Testing various conditions (Supporting Information Table S2**)** indicated that the expected aza‐decalin product **23** is in equilibrium with the corresponding ring‐opened primary amine **24** as a result of the acidic nature of the proton between the ketone carbonyl and nitroarene (Scheme 4). As isolation of this dynamic mixture proved challenging, we opted to acylate the product in situ with chloroacetyl chloride. Performing the reaction in acetone proved key in this transformation, since poorly separable mixtures were formed in other solvents. This procedure finally allowed the isolation of a single product (**25 a**), albeit in moderate yield (48 %).

**Scheme 4:**
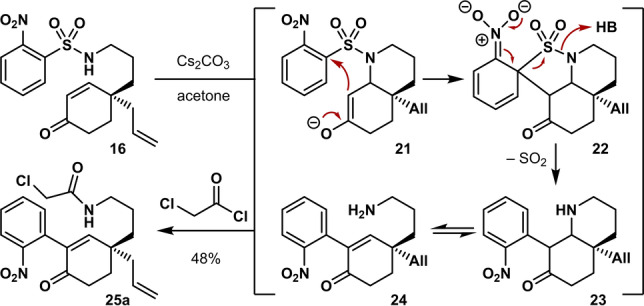
Nitroaryl transfer/acylation cascade reaction of **16**.

To further investigate the scope of this cascade process, we also tested several functionalized bicyclic sulfonamides (**26 a**–**h**, Scheme 5). Because of a slightly different approach during the synthesis of these sulfonamides (see Supporting Information), we performed the scope study with the piperidine **D** ring already closed. A control experiment with the closed parent cascade substrate **26 a** showed no difference in yield, suggesting that conjugate addition of the sulfonamide is not rate‐determining in the sequence. The introduction of a fluoro, chloro or methoxy substituent at the 4‐position (**26 b**,**c**,**e**) did not significantly change the outcome of the reaction. Notably, the halide‐substituted substrates **26 b**–**d** were fully converted in 2–3 h (vs. 18 h for **26 a**), while methoxy‐substituted **26 e** required 72 h to reach full conversion. The strongly electron‐withdrawing trifluoromethyl group at the same position drastically decreased the efficiency (**26 d**). Substitution at the 3‐position either led to no conversion (**26 f**) or a range of side products (**26 g**). Plausibly, delocalization of the lone pairs on the methoxy oxygen atom in **26 f** toward the nitro group considerably destabilizes the Meisenheimer intermediate in the Truce–Smiles rearrangement. The reaction with 2‐cyanobenzenesulfonamide **26 h** did not show any conversion either.

**Scheme 5:**
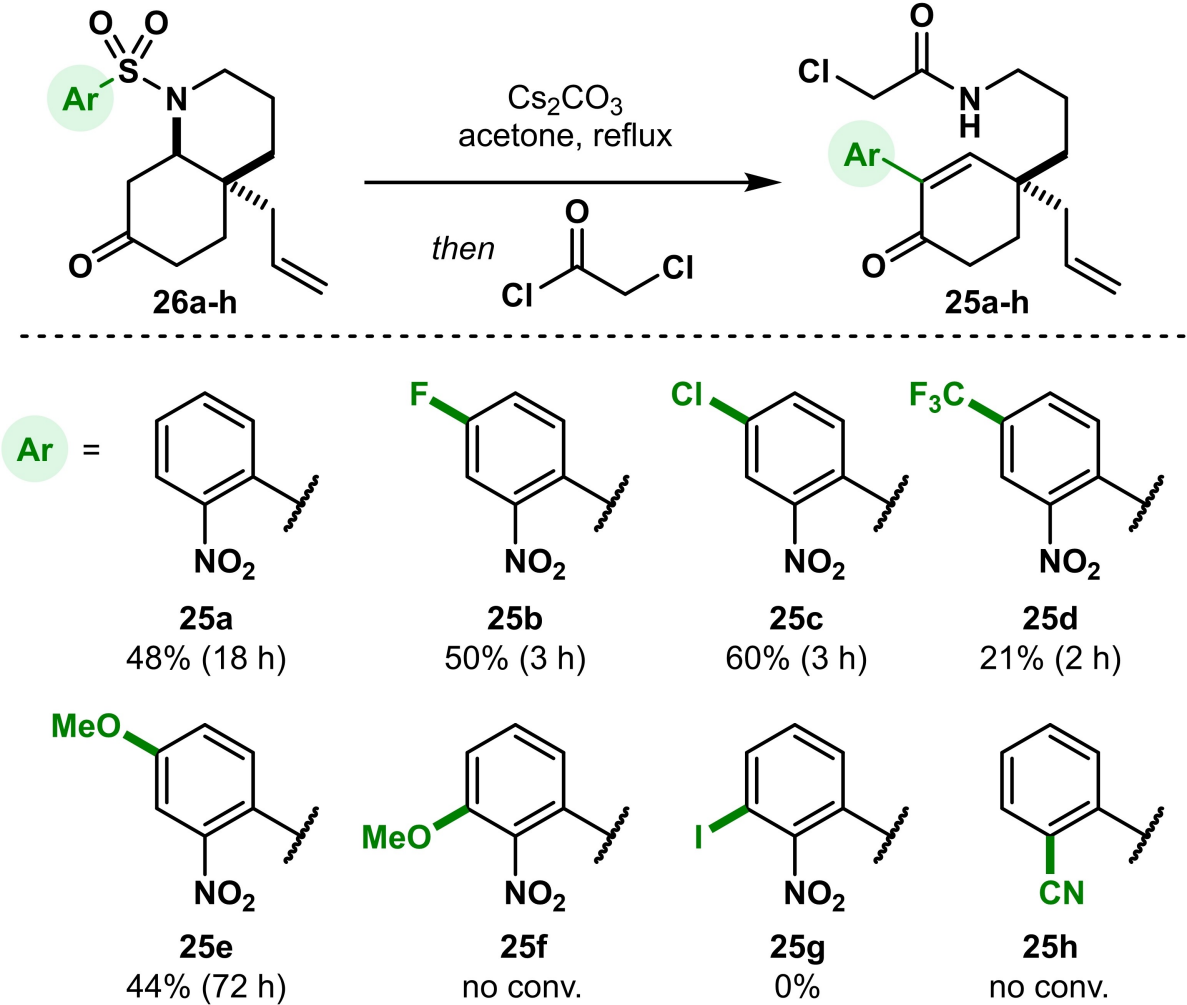
Scope of the cascade reaction.

To continue our efforts toward MIAs (Scheme 6), reduction of the nitro group in **25 a** proceeded most efficiently with iron in acetic acid. Advantageously, this reduction/condensation proceeded with concomitant closure of the **D** ring, affording tetracycle **27** in 62 % yield as a single diastereomer, as corroborated by X‐ray crystallography.^[20]^ Other reducing agents such as zinc or TiCl_3_ proved less effective. The **E** ring was closed in 81 % yield by nucleophilic attack of the indole on the chloroacetamide by a Finkelstein halide exchange followed by further activation with AgOTf. Indolenine **14** proved to be prone to degradation, and any further transformations had to be performed immediately afterward. As previously reported by Shao et al.,^[4e]^ all attempts to oxidatively cleave the alkene in **14** at this stage proved futile. Instead, we fully reduced indolenine **14** with LiAlH_4_ to give tertiary amine **28** in 99 % yield. Notably, **28** could be obtained from **27** in 89 % yield over two steps without intermediate purification of **14**. Unfortunately, neither conventional ozonolysis nor Lemieux‐Johnson oxidation (both with reductive workup [NaBH_4_]) provided the desired product **2**. Possibly, the basic nitrogen atoms in **28** are prone to oxidation, leading to a range of polar side products. Gratifyingly, conversion of **28** to the corresponding dihydrochloride followed by ozonolysis with reductive workup afforded (−)‐limaspermidine (*ent‐* **2**) in 59 % yield.

**Scheme 6:**
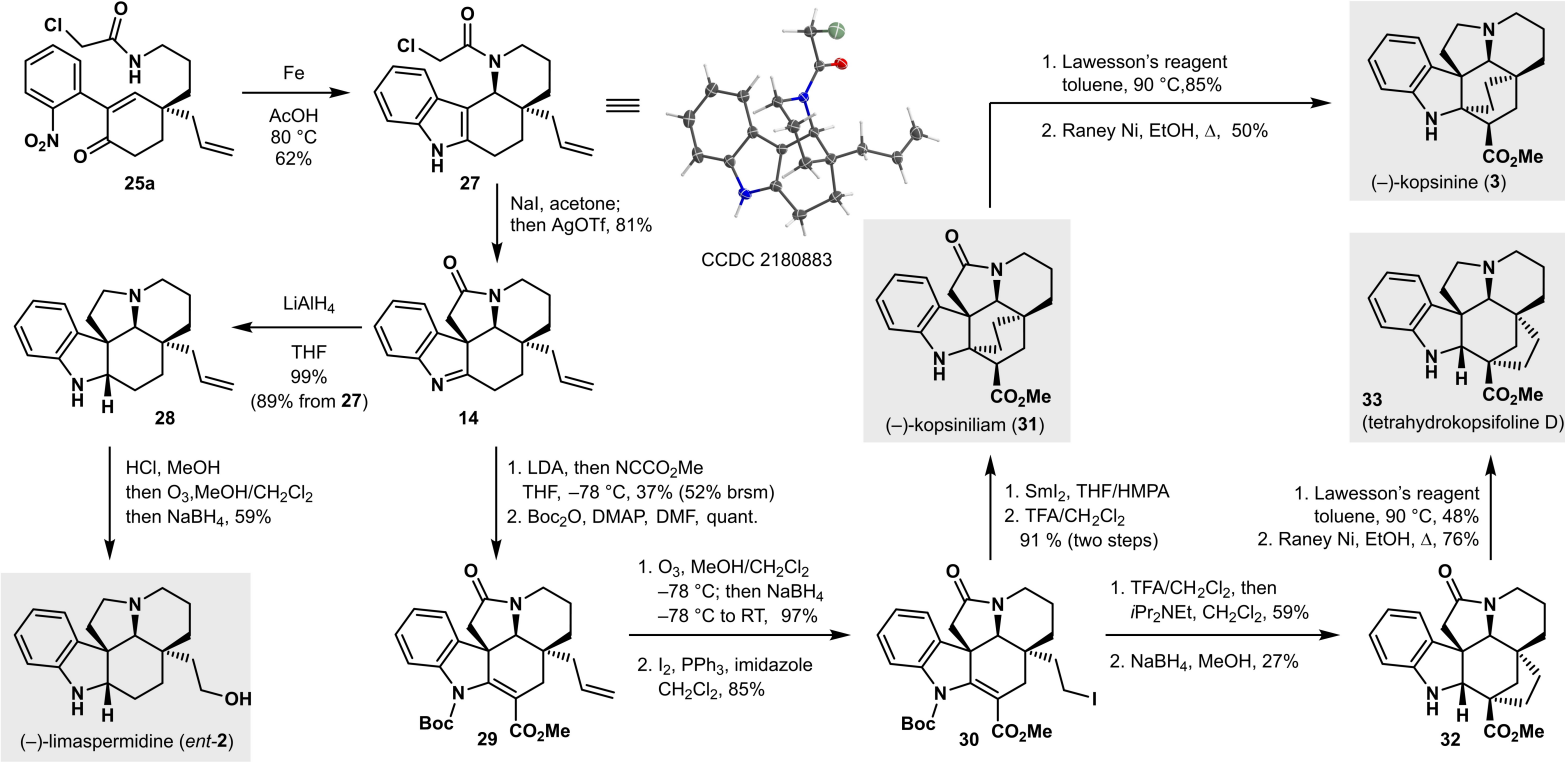
Total syntheses of (−)‐limaspermidine (*ent‐* **2**), (−)‐kopsinine (**3**), (−)‐kopsinilam (**31**), and tetrahydrokopsifoline D (**33**). THF=tetrahydrofuran, LDA=lithium diisopropylamide, DMAP=4‐dimethylaminopyridine, DMF=*N,N*‐dimethylformamide, HMPA=hexamethylphosphoramide, TFA=trifluoroacetic acid.

Routes towards (−)‐kopsinine and the kopsifoline core were also envisioned from indolenine **14**. Carboxylation of similar indolenines using Mander's reagent was previously reported by Andrade in his synthesis of tabersonine.^[21]^ The use of superstoichiometric amounts of base led to formation of a side product with an additional ester moiety at the α‐position of the lactam. Using one equivalent of base did not lead to full conversion of the starting indolenine, but exclusively gave the mono ester in 37 % yield (52 % based on recovered starting material). Boc protection of the indoline nitrogen then gave **29** in quantitative yield. Subsequent ozonolysis with reductive workup followed by Appel reaction cleanly afforded iodide **30**. Formation of the bicyclo[2.2.2]octane fragment was efficiently achieved using a SmI_2_‐mediated diastereoselective free radical cyclization, as previously demonstrated by Boger on a similar substrate.[^7c^, ^7e^] Boc deprotection then afforded (−)‐kopsinilam (**31**). The total synthesis of (−)‐kopsinine (**3**) was completed by converting **31** to the thiolactam followed by reduction with Raney nickel.[^6^, ^7c^]

Interestingly, when we deprotected iodide **30** and subjected it to slightly basic workup, we observed partial conversion to the bicyclo[1.2.3]octane framework of the kopsifoline alkaloids. We further optimized this transformation by treating the crude deprotected iodide with *i*Pr_2_NEt. Reduction of the resulting indolenine to indoline **32** with excess NaBH_4_ surprisingly also reduced the methyl ester to the primary alcohol for a substantial part. To reach the target unnatural alkaloid tetrahydrokopsifoline D (**33**),^[22]^ we again reduced the γ‐lactam to the analogous pyrrolidine via the thioamide.

In conclusion, we demonstrated the asymmetric total synthesis of three distinct natural product families from a single common intermediate which was prepared via a fully regioselective nitroaryl transfer cascade reaction. Importantly, this cascade approach allows for late‐stage variation by using differently substituted nitroarenesulfonamide precursors.

## Conflict of interest

The authors declare no conflict of interest.

## Supporting information

As a service to our authors and readers, this journal provides supporting information supplied by the authors. Such materials are peer reviewed and may be re‐organized for online delivery, but are not copy‐edited or typeset. Technical support issues arising from supporting information (other than missing files) should be addressed to the authors.

Supporting Information

Supporting Information

## Data Availability

The data that support the findings of this study are available from the corresponding author upon reasonable request.
