# Supplementary material for: Enantioselective Total Synthesis of (−)‐Limaspermidine and (−)‐Kopsinine by a Nitroaryl Transfer Cascade Strategy
Source: Angew Chem Int Ed Engl. 2022 Sep 12;61(42):e202210592. doi: 10.1002/anie.202210592 (PMC9826323; doi:10.1002/anie.202210592)
Supplement: Supplementary file 2 — Supporting Information [file ANIE-61-0-s002.pdf]

Angewandte Chemie
Eine Zeitschrift der Gesellschaft Deutscher Chemiker

Supporting Information

Enantioselective Total Synthesis of (−)-Limaspermidine and (−)-Kopsinine by a Nitroaryl Transfer Cascade Strategy

*B. Horst, D. S. Verdoorn, S. Hennig, G. van der Heijden, E. Ruijter**

Table of Contents

General information 3

Reaction optimization for the enantioselective decarboxylative Tsuji-Trost allylation 4

Conditions tested in the Michael/Truce-Smiles cascade 6

Synthetic procedures and characterisation 7

NMR Spectra 19

Chiral SFC traces of **17**, optimization of enantioselective decarboxlative Tsuji-Trost 60

Enantiomeric enrichment by crystallization of compound **27** 64

Crystallographic data for **27** 66

Supporting references 69

General information

Commercially available reagents were purchased from Merck (Sigma-Aldrich), Fischer scientific, Strem Chemicals, TCI Europe or Fluorochem and were used as received, unless mentioned otherwise. Solvents were purchased from VWR Chemicals or Sigma-Aldrich and used without purification, unless stated otherwise. Anhydrous, air-free solvents (DCM, toluene, THF) were obtained from a PureSolv MD 5 solvent purification system. Ozone was generated with the ozonetech ACT-3000 connected to compressed air, passing through a home-made drying system. Nuclear magnetic resonance (NMR) spectra were recorded on a Bruker Avance 600, Bruker Avance 500 or Bruker Avance 300, as indicated. Thereby using the residual $CHCl_3$ signal (^{1}H: δ 7.26 ppm) or the $CDCl_3$ signal (^{13}C{1H}: δ 77.16 ppm) as internal reference standard. Chemical shifts (δ) are given in ppm and coupling constants (J) are quoted in hertz (Hz). Resonances are described as s (singlet), d (doublet), t (triplet), q (quartet), br (broad singlet) and m (multiplet) or combinations thereof. Electrospray Ionization (ESI) high-resolution mass spectrometry was carried out using a Bruker QTOF impact II instrument in positive ion mode (capillary potential of 4500 V). Flash chromatography was performed on Silicycle Silica-P Flash Silica Gel (particle size 40-63 µm, pore diameter 60 Å) using the indicated eluent. Thin Layer Chromatography (TLC) was performed using TLC plates from Merck (SiO_2, Kieselgel 60 F254 neutral, on aluminium with fluorescence indicator) and compounds were visualized by UV detection (254 nm), potassium permanganate stain and/or cerium (IV) sulphate stain. SFC-MS analysis was conducted using a Shimadzu Nexera SFC-MS equipped with a Nexera X2 SIL-30AC autosampler, Nexera UC LC-30AD SF CO_2 pump, Nexera X2 LC-30AD liquid chromatograph, Nexera UC SFC-30A back pressure regulator, prominence SPD-M20A diode array detector, prominence CTO-20AC column oven and CBM-20A system controller, coupled to a. Shimadzu LCMS-2020 mass spectrometer. The data were acquired in full-scan APCI mode (MS) from m/z 100 to 800 in positive ionisation mode. Data was processed using Shimadzu Labsolutions 5.82. Enantiomeric excess was determined by SFC-MS analysis using: Method A) Lux® 3 µm Cellulose-3 column (cellulose tris(4-methylbenzoate), 150 x 4.6 mm) eluting with an isocratic mixture of supercritical CO_2 (99%) and methanol (1%) at 1 mL per minute, run length of 10 minutes, detection at 246 nm; Method B) Lux® 3 µm Cellulose-1 column (cellulose tris(3,5-dimethylphenylcarbamate). eluting with an isocratic mixture of supercritical CO_2 (70%) and methanol (30%) at 2 mL per minute, run length of 5.5 minutes, detection at 222 nm. The sample injection volume was 5 µL. Specific rotations were measured on a Krüss P3000 polarimeter, using a 0.5 dm cell and solvent as indicated.

Reaction optimization for the enantioselective decarboxylative Tsuji-Trost allylation

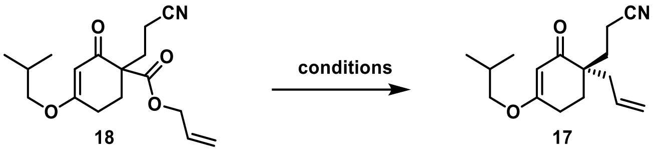

Table S1. Optimization Table.

Entry[a]	Cat	Ligand	Solvent	T (°C)	Yield (%)[b]	ee (%)[c]
1	$Pd_2(dba)_3$	**L1**	THF	65	77	-
2	$Pd_2(dba)_3$	**L2**	THF	65	93	68
3	$Pd_2(dba)_3$	**L3**	THF	65	92	81
4	$Pd_2(dba)_3$	**L4**	THF	65	94	81
5	$Pd_2(dba)_3$	**L5**	THF	65	92	-53
6	$Pd_2(dba)_3$	**L6**	THF	65	97	2
7	$Pd_2(dba)_3$	**L7**	THF	65	92	9
8	$Pd_2(dba)_3$	**L8**	THF	65	81	4
9	$Pd_2(dba)_3$	**L9**	THF	65	98	1
10	$Pd_2(dba)_3$	**L10**	THF	65	85	-6
11	$Pd_2(dba)_3$	**L11**	THF	65	80	1
12	$Pd_2(dba)_3$	**L12**	THF	65	90	1
13	$Pd_2(dba)_3$	**L13**	THF	65	69	-5
14	$Pd_2(dba)_3$	**L14**	THF	65	77	0
15	$Pd_2(dba)_3$	**L15**	THF	65	97	-10
16	$Pd_2(dba)_3$	**L16**	THF	65	no conv.[d]	-
17	$Pd_2(dba)_3$	**L17**	THF	65	no conv.[d]	-
18	$Pd_2(dba)_3$	**L18**	THF	65	no conv.[d]	-
19	$Pd_2(dba)_3$	**L3**	toluene	RT	35	87
20	$Pd_2(dba)_3$	**L4**	toluene	RT	40	90
21	$Pd_2(dba)_3$	**L3**	toluene	65	97	83
22	$Pd_2(dba)_3$	**L4**	toluene	65	46	89
23	$Pd_2(dba)_3$	**L3**	1,4-dioxane	RT	no conv.[d]	
24	$Pd_2(dba)_3$	**L4**	1,4-dioxane	RT	36	90
25	$Pd_2(dba)_3$	**L3**	1,4-dioxane	80	66	80
26	$Pd_2(dba)_3$	**L4**	1,4-dioxane	80	no conv.[d]	
27	$Pd_2(dba)_3$	**L3**	diethyl ether	RT	84	91
28	$Pd_2(dba)_3$	**L4**	diethyl ether	RT	86	90
29[e]	$Pd_2(dba)_3$	**L3**	diethyl ether	RT	96	91

[a] All reaction were performed using 0.20 mmol of **18**, at 0.05 M concentration. Catalyst loading (unless stated otherwise): 5.0 µmol $Pd_2(dba)_3$ (5 mol% Pd), 20 µmol ligand (10 mol%), Pd/ligand ratio 1:2.

[b] Isolated yield after automated column chromatography (silica gel, 0-20% EtOAc in cHex, 11 min.).

[c] Determined by chiral SFC-MS (Method A, see general information).

[d] Did not show conversion after 7 days.

[e] Performed at 16 g scale, with lower catalyst loading [1 mol% $Pd_2(dba)_3$, 4 mol% ligand], leading to an extended reaction time of 6 days to reach full completion.

List of ligands screened:

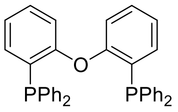

L1

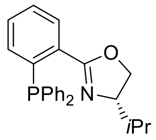

L2

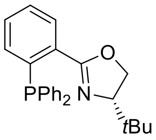

L3

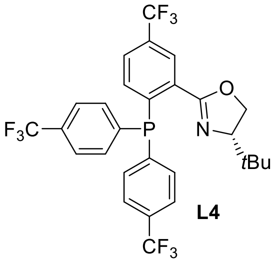

L4

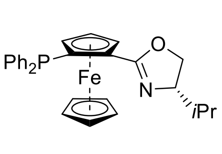

L5

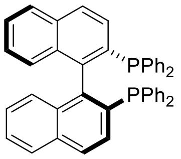

L6

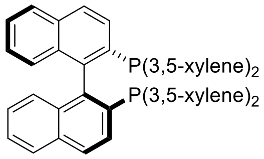

L7

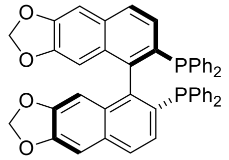

L8

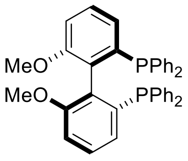

L9

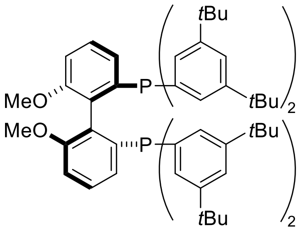

L10

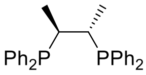

L11

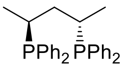

L12

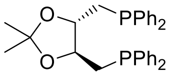

L13

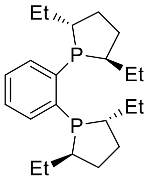

L15

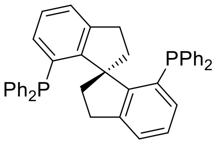

L14

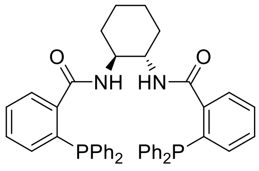

L16

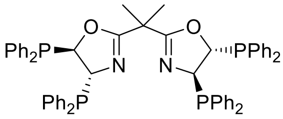

L17

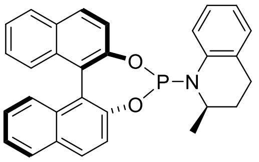

L18

Conditions tested in the Michael/Truce-Smiles cascade

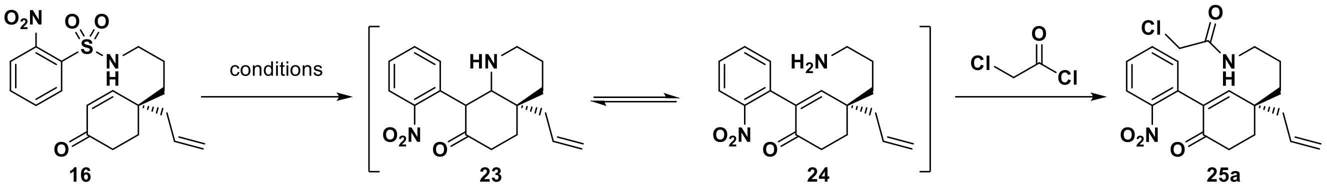

Table S2. List of conditions tested.

Entry	Solvent	Cs_2CO_3 (eq.)	Temperature (° C)	Time (h)	Electrophile (eq)[a]	Conversion of **16**	Yield of **25a** [b]
1	acetonitrile	3	60	18	No	Full	Mixture[c]
2	DMSO	3	60	18	No	Full	Mixture [c]
3	DMSO	**2**	60	18	No	Full	Mixture [c]
4	DMSO	**1**	60	18	No	Full	Mixture [c]
5	DMF	3	60	18	No	Full	Mixture [c]
6	acetone	2	56	18	No	Full	Mixture [c,d]
7	THF	2	60	18	No	Slow	Mixture [c]
8	chloroform	2	60	18	No	No	0%
9	DMSO	2	100	18	No	Full	Mixture [c]
10	DMSO	2	60	18	yes	Full	0%
11	acetonitrile	2	60	18	yes	Full	17%
12	DMF	2	60	18	yes	Full	17%
**13**	**acetone**	**2**	**56**	**18**	**yes**	**Full**	**50% (48%[e])**
14	acetone	2	100[f]	3	yes	Full	29%

[a] Electrophile added after indicated temperature and time, and after cooling to room temperature.
[b] Determined by ^1H NMR analysis using 0.5 eq. of durene as internal standard.
[c] No pure product could be isolated after column chromatography (silica gel or alumina, using cyclohexane/EtOAc; DCM/MeOH; or DCM/MeOH/Et_3N as eluent systems).
[d] ^1H NMR shows complete conversion to **24**, although this product could not be isolated.
[e] Isolated yield after chromatography.
[f[Microwave irradiation in pressure tube.

Synthetic procedures and characterisation

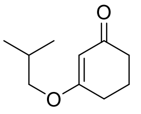

3-isobutoxycyclohex-2-en-1-one (S1)[1]

A flame-dried flask was charged with 1,3-cyclohexadione (25.0 g, 223 mmol, 1 eq.), isobutanol (41.2 mL, 446 mmol, 2.0 eq.), *p*-toluenesulfonic acid (0.425 g, 2.23 mmol, 0.010 eq.) and toluene (300 mL). This solution was brought to reflux under Dean-Stark conditions for 3 h. The reaction mixture was concentrated *in vacuo* and purified by vacuum distillation (2 mbar, 98 °C) to give the product as colorless oil (35.6 g, 212 mmol, 95 %) that solidifies in the freezer.

R$_f$ (30 % EtOAc in cyclohexane): 0.39 (visualized by UV and $KMnO_4$ stain).

^{1}H NMR (600 MHz, $CDCl_3$) δ 5.35 (s, 1H), 3.59 (d, *J* = 6.5 Hz, 2H), 2.41 (t, *J* = 6.2 Hz, 2H), 2.34 (dd, *J* = 7.3, 6.0 Hz, 2H), 2.09 – 2.00 (m, 1H), 2.00 – 1.94 (m, 2H), 0.97 (d, *J* = 6.7 Hz, 6H).

^{13}C NMR (151 MHz, $CDCl_3$) δ 200.02, 178.38, 102.86, 77.37, 77.16, 76.95, 74.86, 36.88, 29.15, 27.85, 21.38, 19.21.

HRMS (ESI) *m/z* calculated for $C_{10}H_{17}O_2$ $[M+H]^+$: 169.1223, found: 169.1223.

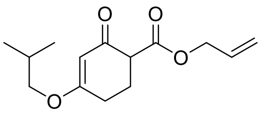

Allyl 4-isobutoxy-2-oxocyclohex-3-ene-1-carboxylate (S2)

A flame-dried flask was charged with diisopropyl amine (34.4 mL, 244 mmol, 2.05 eq.) and toluene (500 mL) and was cooled to –78 °C, after which a solution of *n*-buthyllithium (11 M in hexanes, 21.8 mL, 240 mmol, 2.0 eq.) was added dropwise. The mixture was stirred at –78 °C for 20 minutes and a solution of ketone **S1** (20.0 g, 119 mmol, 1 eq.) in toluene (150 mL) was added dropwise, resulting in an orange solution that was stirred for 1.5 h at –78 °C. Allyl chloroformate (13.9 mL, 131 mmol, 1.1 eq.) was added dropwise at –78 °C and the reaction was kept at this temperature for 15 minutes, after which it was allowed to warm to room temperature. After 18 h, the reaction was quenched by the addition of saturated aqueous ammonium chloride solution and extracted three times with ethyl acetate. The combined organic extracts were washed with brine and dried over magnesium sulphate. The crude product (~33 g) could directly be used in the next step without any further purification. The product can be obtained as a colourless oil after flash chromatographic purification (silica gel, 15% ethyl acetate in cyclohexane).

R$_f$ (30 % EtOAc in cyclohexane): 0.57 (visualized by UV and $KMnO_4$ stain).

^{1}H NMR (600 MHz, $CDCl_3$) δ 5.93 (ddt, *J* = 17.2, 10.5, 5.7 Hz, 1H), 5.38 (s, 1H), 5.34 (ddt, *J* = 17.2, 1.4 Hz, 1H), 5.24 (ddt, *J* = 10.5, 1.4 Hz, 1H), 4.70 – 4.61 (m, 2H), 3.66 – 3.55 (m, 2H), 3.36 (dd, *J* = 9.2, 5.0 Hz, 1H), 2.57 (ddd, *J* = 17.5, 6.3, 5.0 Hz, 1H), 2.49 – 2.40 (m, 1H), 2.41 – 2.32 (m, 1H), 2.26 – 2.14 (m, 1H), 2.07 – 1.98 (m, 1H), 0.97 (d, *J* = 6.7 Hz, 6H).

^{13}C NMR (151 MHz, $CDCl_3$) δ 193.70, 177.94, 170.25, 132.02, 118.54, 102.23, 77.37, 77.16, 76.95, 75.14, 65.89, 52.45, 27.84, 27.41, 24.30, 19.18.

HRMS (ESI) *m/z* calculated for $C_{14}H_{20}O_4$ $[M+Na]^+$: 275.1254, found: 275.1251.

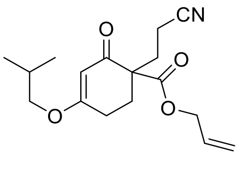

Allyl 1-(2-cyanoethyl)-4-isobutoxy-2-oxocyclohex-3-ene-1-carboxylate (18)

A flame-dried flask was charged with crude β-keto ester **S2** (33 g), acetonitrile (300 mL), potassium carbonate (32.9 g, 238 mmol, 2.0 eq.) and acrylonitrile (15.7 mL, 238 mmol, 2 eq.). The resulting suspension was heated to reflux for 6 h. The reaction mixture was quenched with aqueous hydrochloric acid (1 M) and extracted three times with ethyl acetate. The combined organic layers were washed with brine and dried over sodium sulphate. The product was purified by flash column chromatography (silica gel, 15% ethyl acetate in cyclohexane) and isolated as a yellow oil (30 g, 98 mmol, 82% over two steps) that solidified in the freezer.

R$_f$ (20% EtOAc in cyclohexane): 0.30 (visualized by UV and $KMnO_4$ stain).

^{1}H NMR (600 MHz, $CDCl_3$) δ 5.87 (ddt, *J* = 17.2, 10.4, 5.7 Hz, 1H), 5.37 (s, 1H), 5.30 (ddt, *J* = 17.2, 1.4 Hz, 1H), 5.25 (ddt, *J* = 10.4, 1.4 Hz, 1H), 4.72 – 4.55 (m, 2H), 3.61 (d, *J* = 6.5 Hz, 2H), 2.63 – 2.39 (m, 5H), 2.25 (ddd, *J* = 14.0, 9.2, 6.5 Hz, 1H), 2.16 (ddd, *J* = 14.0, 9.2, 6.5 Hz, 1H), 2.07 – 1.91 (m, 2H), 0.97 (d, *J* = 6.7 Hz, 6H).

^{13}C NMR (151 MHz, $CDCl_3$) δ 194.72, 177.10, 170.88, 131.46, 119.76, 119.12, 102.15, 75.33, 66.27, 55.07, 30.04, 29.41, 27.84, 26.26, 19.18, 19.16, 13.40.

HRMS (ESI) *m/z* calculated for $C_{17}H_{24}NO_4$ $[M+H]^+$: 306.1700, found: 306.1697.

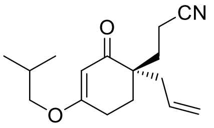

General procedure for the Tsuji-Trost decarboxylative allylation, ligand screening

In a flame dried Schlenk flask, under argon atmosphere, $Pd_2(dba)_3$ (4.60 mg, 5.0 µmol, 2.5 mol%) and ligand (20 µmol, 10 mol%) were dissolved in degassed solvent (1.00 mL). The resulting mixture was stirred at room temperature for 15 minutes until no purple colour was observed anymore. A solution of allylic ester **18** (61.0 mg, 0.20 mmol, 1 eq.) in solvent (3.00 mL) was added to the catalytic mixture, which was then heated to the indicated temperature until full conversion of starting material was observed on TLC. The mixture was cooled down to room temperature and all volatiles were removed *in vacuo*. The product was purified by automated column chromatography (silica gel, eluting at 25% EtOAc in cyclohexane) and isolated as a yellow oil. Enantiomeric excess was determined using chiral SFC (method A).

Optimized large scale procedure for (*S*)-3-(1-allyl-4-isobutoxy-2-oxocyclohex-3-en-1-yl)propanenitrile (17)

In a flame dried Schlenk flask, under argon atmosphere, $Pd_2(dba)_3$ (480 mg, 0.524 mmol, 1 mol%) and (*S*)-*tert*-butyl PHOX ligand (812 mg, 2.10 mmol, 4 mol%) were dissolved in degassed diethyl ether (200 mL). The resulting mixture was stirred at room temperature for 30 minutes until no purple color was observed anymore. A solution of allylic ester **18** (16.0 g, 52.4 mmol, 1 eq.) in degassed diethyl ether (800 mL) was added to the catalytic mixture, which was then stirred at room temperature until full conversion of starting material was observed on TLC (6 days). All volatiles were removed *in vacuo* and the product was purified by flash column chromatography (silica gel, 10-20% EtOAc in cyclohexane) to give a yellow oil (11.6 g, 43.4 mmol, 85%). Enantiomeric excess was determined using chiral SFC (method A) at 91%.

R_f (25% EtOAc in cyclohexane): 0.51 (visualized by UV and $KMnO_4$ stain).

^{1}H NMR (600 MHz, $CDCl_3$) δ 5.68 (ddt, *J* = 17.4, 10.1, 7.4 Hz, 1H), 5.26 (s, 1H), 5.15 – 5.08 (m, 2H), 3.58 (d, *J* = 6.5 Hz, 2H), 2.51 (dddd, *J* = 18.1, 8.0, 5.9, 0.8 Hz, 1H), 2.43 (dt, *J* = 18.1, 5.9 Hz, 1H), 2.40 – 2.25 (m, 3H), 2.22 (ddt, *J* = 14.2, 7.6, 1.2 Hz, 1H), 2.05 – 1.98 (m, 2H), 1.92 – 1.86 (m, 2H), 1.81 (ddd, *J* = 13.9, 10.4, 5.6 Hz, 1H), 0.97 (dd, *J* = 6.8, 1.3 Hz, 7H).

^{13}C NMR (151 MHz, $CDCl_3$) δ 201.05, 176.46, 132.87, 120.28, 119.30, 101.73, 75.10, 45.83, 39.10, 30.70, 29.28, 27.85, 25.63, 19.19, 19.17, 12.47.

HRMS (ESI) *m/z* calculated for $C_{16}H_{24}NO_2$ $[M+H]^+$: 262.1802, found: 262.1800.

$[\alpha]_D^{20}$ +2.0 (c = 1.00, $CHCl_3$).

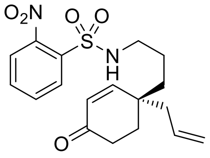

(*S*)-*N*-(3-(1-allyl-4-oxocyclohex-2-en-1-yl)propyl)-2-nitrobenzenesulfonamide (16)

In a flame-dried flask, nitrile **17** (13.2 g, 50.3 mmol, 1 eq.) was dissolved in dry THF (500 mL) and cooled to −78 °C. A solution of diisobutylaluminium hydride in hexanes (1.0 M, 56 mL, 56 mmol, 1.1 eq.) was added dropwise to the mixture and the reaction was allowed to room temperature slowly overnight. The reaction mixture was transferred to a dropping funnel and slowly added to a pre-cooled suspension of lithium aluminium hydride (5.78 g, 151 mmol, 3.0 eq.) in dry THF (200 mL) at 0 °C. The resulting mixture was allowed to room temperature and stirred overnight. The reaction was diluted with diethyl ether (500 mL), cooled to 0 °C and quenched with water (8 mL, NOTE: violent gas evolution!), aqueous sodium hydroxide (15% m/v, 8 mL) and water (23 mL). The mixture was stirred for 15 minutes after which anhydrous sodium sulphate was added. The mixture was stirred for 15 minutes and filtered over celite, washing the residue extensively with diethyl ether and THF. All volatiles were removed *in vacuo* and the crude amino alcohol (12.7 g) was used directly in the next step.

The amino alcohol of the previous step was dissolved in dry DCM (250 mL) and triethyl amine (21 mL, 151 mmol, 3.0 eq.). The solution was cooled to 0 °C and 2-nitrobenzenesulfonyl chloride (16.7 g, 75.5 mmol, 1.5 eq.) was added portion wise over 10 minutes. The reaction mixture was allowed to room temperature and stirred overnight. All volatiles were thoroughly removed *in vacuo* and the residue was taken up in methanol (200 mL) and aqueous hydrochloric acid (1.0 M, 200 mL). Minimal amounts of DCM (~25 mL) were added to create two clear layers, which were then vigorously stirred for 1 hour. The whole mixture was extracted three times with DCM, and the combined organic layers were washed with saturated aqueous sodium bicarbonate solution and brine. The organic layer was dried over magnesium sulphate and concentrated *in vacuo*. The crude mixture was purified by flash column chromatography (silica gel, 10–40% EtOAc in cyclohexane) to give the title compound as slightly yellow oil (13.2 g, 34. 9 mmol, 69%) with trace impurities of the intramolecular Michael adduct.

R_f (50% EtOAc in cyclohexane): 0.49 (visualized by UV and $KMnO_4$ stain).

^{1}H NMR (600 MHz, $CDCl_3$) δ 8.12 (ddd, *J* = 5.8, 3.1, 1.0 Hz, 1H), 7.87 – 7.84 (m, 1H), 7.77 – 7.73 (m, 2H), 6.59 (d, *J* = 10.3 Hz, 1H), 5.90 (d, *J* = 11.4 Hz, 1H), 5.70 (ddd, *J* = 16.9, 10.1, 7.4 Hz, 1H), 5.36 (t, *J* = 5.7 Hz, 1H), 5.12 (dd, *J* = 10.1, 1.6 Hz, 1H), 5.09 (dd, *J* = 16.9, 1.6 Hz, 1H), 3.17 – 3.07 (m, 2H), 2.44 (ddd, *J* = 17.4, 8.0, 5.4 Hz, 1H), 2.36 (ddd, *J* = 17.4, 8.0, 5.4 Hz, 1H), 2.19 (d, *J* = 7.4 Hz, 1H), 1.91 – 1.76 (m, 2H), 1.60 – 1.43 (m, 4H).

^{13}C NMR (151 MHz, $CDCl_3$) δ 199.20, 157.04, 148.22, 133.89, 133.79, 133.07, 132.96, 131.14, 128.78, 125.56, 119.24, 44.29, 42.45, 38.28, 34.85, 33.91, 31.01, 24.64.

HRMS (ESI) *m/z* calculated for $C_{18}H_{23}N_2O_5S$ $[M+H]^+$: 379.1322, found: 379.1315.

$[\alpha]_D^{20}$ –24 (c = 1.0, $CHCl_3$).

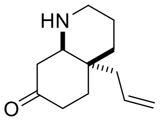

4a-allyloctahydroquinolin-7(1*H*)-one (S3)

In a flame-dried flask, cyanide **17** (13.2 g, 50.3 mmol, 1 eq.) was dissolved in dry THF (500 mL) and cooled to –78 °C. Then, a solution of diisobutylaluminium hydride in hexanes (1 M, 56 mL, 56 mmol, 1.1 eq.) was added dropwise to the mixture and the reaction was allowed to room temperature slowly overnight. The reaction mixture was transferred to a dropping funnel and slowly added to a pre-cooled suspension of lithium aluminium hydride (5.78 g, 151 mmol, 3.0 eq.) in dry THF (200 mL) at 0°C. The resulting mixture was allowed to room temperature and stirred overnight. The reaction was diluted with diethyl ether (500 mL), cooled to 0 °C and quenched with water (8 mL, NOTE violent gas evolution!), aqueous sodium hydroxide (15% m/v, 8 mL) and water (23 mL). The mixture was stirred for 15 minutes after which anhydrous sodium sulphate was added. The mixture was stirred for 15 minutes and filtered over celite, washing the residue extensively with diethyl ether and THF. All volatiles were removed *in vacuo* and the crude amino alcohol (12.7 g). This residue was taken up in methanol (200 mL) and aqueous hydrochloric acid (1 M, 200 mL). Minimal amounts of DCM (~25 mL) were added to create two clear layers, which were then vigorously stirred for 1 hour. Subsequently, the mixture was basified to pH>14 and extracted three times with DCM The combined organic layers were washed with

saturated aqueous sodium bicarbonate solution and brine and dried over magnesium sulphate and concentrated *in vacuo*. The crude mixture was purified by flash column chromatography (silica gel, cyclohexane/EtOAc/Et_3N 16:8:1) to give the title compound as colourless oil (13.2 g, 34. 9 mmol, 69%).

R_f (8:8:1 cyclohexane/EtOAc/Et_3N): 0.50 (visualized by UV and $KMnO_4$ stain).

^{1}H NMR (500 MHz, $CDCl_3$) δ 5.90 (ddt, *J* = 16.0, 10.9, 7.5 Hz, 1H), 5.17 – 5.08 (m, 2H), 3.03 (dddd, *J* = 11.4, 4.4, 2.6, 1.6 Hz, 1H), 2.89 – 2.84 (m, 1H), 2.76 (dd, *J* = 15.1, 4.0 Hz, 1H), 2.57 (td, *J* = 11.7, 3.1 Hz, 1H), 2.52 – 2.37 (m, 3H), 2.32 – 2.25 (m, 1H), 2.22 (dd, *J* = 13.9, 7.5 Hz, 1H), 2.05 (dt, *J* = 15.0, 2.7 Hz, 1H), 1.69 (dddd, *J* = 17.2, 11.7, 8.6, 4.3 Hz, 1H), 1.61 – 1.53 (m, 1H), 1.49 – 1.31 (m, 4H).

^{13}C NMR (126 MHz, $CDCl_3$) δ 210.97, 133.70, 118.22, 62.27, 47.26, 44.64, 41.53, 37.17, 35.55, 33.69, 27.82, 21.89.

HRMS (ESI) *m/z* calculated for $C_{12}H_{20}NO$ $[M+H]^+$: 194.1539, found: 194.1542.

General procedure for the synthesis of *ortho*-substituted 2-nitrobenzenesulfonyl chlorides[2]

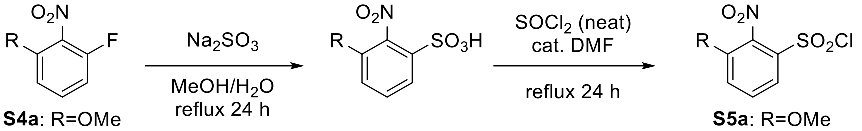

2-Fluoronitrobenzene **S4** (1.5 mmol, 1 eq.) was dissolved in methanol (10.0 mL) and a solution of sodium sulphite (0.38 g, 3.0 mmol, 2 eq.) in water (7 mL) was added. Nitrogen gas was bubbled through the solution for 15 minutes and the mixture was heated to reflux for 24 h. After full conversion of the starting fluoride, the reaction mixture was allowed to room temperature and acidified with aqueous HCl (2M) to pH<3. After removal of all solvents *in vacuo*, the product was recrystallized from boiling brine (6 mL) and collected by vacuum filtration (avoiding washing with water). The sulfonic acid was dissolved in thionyl chloride (1.1 mL, 11 mmol, 7 eq.) and catalytic DMF (48 µL, 0.62 mmol, 0.41 eq.) was added. The reaction mixture was heated to reflux for 24 h, after which it was allowed to room temperature. Evaporation of all volatiles *in vacuo* (caution! Thionyl chloride vapours are highly corrosive) yielded a solid that could be dissolved in DCM and water. After extraction of the aqueous layer with DCM, the combined organic layers were washed with brine and dried over sodium sulphate. Filtration and evaporation of the solvents *in vacuo* yielded the crude sulfonyl chlorides that were used immediately without further purification. Purity of the material was confirmed by ^{1}H NMR spectroscopy.

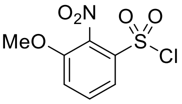

3-methoxy-2-nitrobenzenesulfonyl chloride (S5a)

Isolated as a brown solid.

^{1}H NMR (500 MHz, $CDCl_3$) δ 7.81 – 7.67 (m, 2H), 7.54 (dd, *J* = 7.8, 1.8 Hz, 1H), 4.03 (s, 3H).

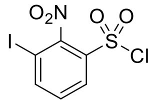

3-iodo-2-nitrobenzenesulfonyl chloride (S5b)

Isolated as an orange solid.

^{1}H NMR (500 MHz, $CDCl_3$) δ 8.30 (dd, *J* = 8.0, 1.3 Hz, 1H), 8.17 (dd, *J* = 8.1, 1.2 Hz, 1H), 7.50 (dd, *J* = 8.0 Hz, 1H).

General procedure for the synthesis of aryl-substituted 2-nitrobenzenesulfonamides

Secondary amine **S3** (100 mg, 0.52 mmol, 1 eq.) was dissolved in dry DCM (5.0 mL) and triethylamine (0.22 mL, 1.6 mmol, 3.0 eq.). The mixture was cooled to 0 °C and the appropriately substituted *ortho*-nitrobenzenesulfonyl chloride (0.78 mmol, 1.5 eq.) was added in one portion. The reaction mixture was allowed to room temperature and was stirred for 18 h, after which water was added. The aqueous layer was extracted with DCM and the combined organic layers were washed with aqueous hydrochloric acid (1 M), saturated aqueous sodium bicarbonate and brine, before it was dried over sodium sulphate. Concentration of the crude *in vacuo* and purification by flash column chromatography (silica gel, 10% EtOAc in toluene) yielded the 2-nitrobenzenesulfonamides described below.

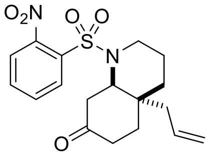

2-nitrobenzenesulfonamide 26a

Isolated as white solid, quantitative yield.

R_f (50% EtOAc in cyclohexane): 0.36 (visualized by UV and $KMnO_4$ stain).

^{1}H NMR (500 MHz, $CDCl_3$) δ 8.05 – 7.99 (m, 1H), 7.74 – 7.61 (m, 3H), 5.67 (dddd, *J* = 16.9, 10.1, 8.3, 6.7 Hz, 1H), 5.09 – 5.04 (m, 1H), 5.04 – 4.97 (m, 1H), 3.98 (dd, *J* = 13.3, 5.0 Hz, 1H), 3.86 (dd, *J* = 12.3, 5.3 Hz, 1H), 3.10 (td, *J* = 13.3, 3.2 Hz, 1H), 2.82 (dd, *J* = 14.6, 12.3 Hz, 1H), 2.53 (dd, *J* = 14.1, 8.3 Hz, 1H), 2.37 (td, *J* = 14.6, 6.2 Hz, 1H), 2.30 – 2.20 (m, 2H), 2.06 – 1.92 (m, 1H), 1.91 – 1.78 (m, 3H), 1.70 – 1.62 (m, 1H), 1.56 (td, *J* = 14.4, 4.9 Hz, 1H), 1.49 – 1.42 (m, 1H).

^{13}C NMR (126 MHz, $CDCl_3$) δ 208.40, 147.77, 133.89, 133.83, 132.79, 132.07, 130.99, 124.49, 119.05, 58.97, 40.92, 40.35, 39.93, 36.53, 35.66, 33.46, 24.44, 20.50.

HRMS (ESI) *m/z* calculated for $C_{18}H_{23}N_2O_5S$ $[M+H]^+$: 379.1322, found: 379.1324.

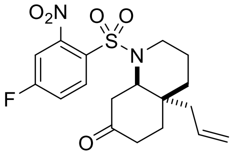

4-fluoro-2-nitrobenzenesulfonamide 26b
Isolated as yellow foam, 35% yield.
R_f (50% EtOAc in cyclohexane): 0.57 (visualized by UV and $KMnO_4$ stain).
^{1}H NMR (500 MHz, $CDCl_3$) δ 8.06 (dd, *J* = 9.5, 5.1 Hz, 1H), 7.43 – 7.34 (m, 2H), 5.68 (dddd, *J* = 16.9, 10.1, 8.3, 6.7 Hz, 1H), 5.09 (dd, *J* = 10.1, 2.0 Hz, 1H), 5.04 (dd, *J* = 16.9, 2.0 Hz, 1H), 3.96 (dd, *J* = 13.7, 5.0 Hz, 1H), 3.84 (dd, *J* = 12.4, 5.3 Hz, 1H), 3.10 (td, *J* = 13.3, 3.3 Hz, 1H), 2.83 (dd, *J* = 14.5, 12.4 Hz, 1H), 2.54 (dd, *J* = 14.1, 8.3 Hz, 1H), 2.38 (td, *J* = 14.7, 6.0 Hz, 1H), 2.31 – 2.22 (m, 2H), 1.99 (qdd, *J* = 13.1, 5.1, 3.9 Hz, 1H), 1.92 – 1.79 (m, 3H), 1.71 – 1.63 (m, 1H), 1.62 – 1.52 (m, 1H), 1.51 – 1.43 (m, 1H).
^{13}C NMR (126 MHz, $CDCl_3$) δ 208.26, 143.38, 135.74, 133.30, 132.81, 132.62, 130.17, 119.18, 116.38, 110.28, 58.77, 40.99, 40.22, 40.11, 36.49, 35.64, 33.39, 24.60, 20.48 (the aromatic carbon atom attached to the sulfonamide could not be found).
^{19}F NMR (470 MHz, $CDCl_3$) δ –100.6.
HRMS (ESI) *m/z* calculated for $C_{18}H_{22}N_2O_5FS$ $[M+H]^+$: 397.1228, found: 397.1248.

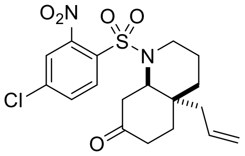

4-chloro-2-nitrobenzenesulfonamide 26c
Isolated as yellow foam, 37% yield.
R_f (50% EtOAc in cyclohexane): 0.62 (visualized by UV and $KMnO_4$ stain).
^{1}H NMR (500 MHz, $CDCl_3$) δ 7.96 (d, *J* = 8.6 Hz, 1H), 7.67 – 7.62 (m, 2H), 5.68 (dddd, *J* = 16.9, 10.1, 8.3, 6.8 Hz, 1H), 5.09 (dd, *J* = 10.1, 1.9 Hz, 1H), 5.04 (dd, *J* = 16.9, 1.9 Hz, 1H), 3.95 (dd, *J* = 13.5, 5.0 Hz, 1H), 3.85 (dd, *J* = 12.4, 5.3 Hz, 1H), 3.10 (td, *J* = 13.5, 3.2 Hz, 1H), 2.83 (dd, *J* = 14.6, 12.4 Hz, 1H), 2.54 (dd, *J* = 14.1, 8.2 Hz, 1H), 2.38 (td, *J* = 14.7, 6.2 Hz, 1H), 2.26 (dq, *J* = 12.5, 3.0 Hz, 2H), 2.06 – 1.92 (m, 1H), 1.92 – 1.79 (m, 3H), 1.67 (dt, *J* = 13.6, 2.9 Hz, 1H), 1.57 (td, *J* = 14.4, 4.8 Hz, 1H), 1.51 – 1.42 (m, 1H).
^{13}C NMR (126 MHz, $CDCl_3$) δ 208.22, 148.07, 139.99, 132.66, 132.48, 132.24, 132.19, 124.74, 119.22, 59.10, 40.94, 40.41, 40.07, 36.52, 35.70, 33.46, 24.41, 20.50.
HRMS (ESI) *m/z* calculated for $C_{18}H_{22}N_2O_5SCl$ $[M+H]^+$: 413.0932, found: 413.0933.

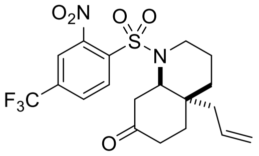

2-nitro-4-(trifluoromethyl)benzenesulfonamide 26d
Isolated as yellow oil, 37% yield.
R_f (50% EtOAc in cyclohexane): 0.66 (visualized by UV and $KMnO_4$ stain).
^{1}H NMR (500 MHz, $CDCl_3$) δ 8.18 (d, *J* = 8.2 Hz, 1H), 7.94 (dd, *J* = 8.2, 1.8 Hz, 1H), 7.90 (d, *J* = 1.8 Hz, 1H), 5.67 (dddd, *J* = 16.9, 10.2, 8.2, 6.7 Hz, 1H), 5.09 (dd, *J* = 10.2, 2.0 Hz, 1H), 5.03 (dd, *J* = 16.8, 2.0 Hz, 1H), 3.98 (dd, *J* = 13.7, 5.1 Hz, 1H), 3.89 (dd, *J* = 12.4, 5.3 Hz, 1H), 3.12 (td, *J* = 13.7, 13.2, 3.2 Hz, 1H), 2.84 (t, *J* = 14.5, 12.4 Hz, 1H), 2.52 (dd, *J* = 14.2, 8.2 Hz, 1H), 2.39 (td, *J* = 14.6, 6.2 Hz, 1H), 2.33 – 2.22 (m, 2H), 2.07 – 1.94 (m, 1H), 1.94 – 1.80 (m, 3H), 1.69 (dt, *J* = 13.1, 3.0 Hz, 1H), 1.63 – 1.53 (m, 1H), 1.48 (dt, *J* = 13.9, 3.4 Hz, 1H).
^{13}C NMR (126 MHz, $CDCl_3$) δ 207.97, 147.83, 137.45, 135.78 (q, *J* = 34.9 Hz), 132.50, 132.07, 128.94 (q, *J* = 3.5 Hz), 122.07 (q, *J* = 273.6 Hz), 121.96 (q, *J* = 3.6 Hz),.119.30, 59.28, 40.94, 40.59, 40.26, 36.51, 35.75, 33.44, 24.41, 20.51.
^{19}F NMR (470 MHz, $CDCl_3$) δ –63.2.
HRMS (ESI) *m/z* calculated for $C_{19}H_{22}N_2O_5F_3S$ $[M+H]^+$: 447.1196, found: 447.1172.

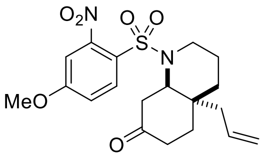

4-methoxy-2-nitrobenzenesulfonamide 26e
Isolated as yellow foam, 74% yield.
R_f (50% EtOAc in cyclohexane): 0.31 (visualized by UV and $KMnO_4$ stain).
^{1}H NMR (500 MHz, $CDCl_3$) δ 7.92 (d, *J* = 9.5 Hz, 1H), 7.16 – 7.06 (m, 2H), 5.69 (dddd, *J* = 17.0, 10.2, 8.3, 6.7 Hz, 1H), 5.08 (dd, *J* = 10.2, 2.0 Hz, 1H), 5.04 (dd, *J* = 17.0, 2.0 Hz, 1H), 3.98 – 3.91 (m, 1H), 3.92 (s, 3H), 3.84 (dd, *J* = 12.3, 5.3 Hz, 1H), 3.08 (td, *J* = 13.3, 3.2 Hz, 1H), 2.81 (dd, *J* = 14.7, 12.3 Hz, 1H), 2.57 (dd, *J* = 14.1, 8.3 Hz, 1H), 2.37 (td, *J* = 14.7, 6.2 Hz, 1H), 2.30 – 2.20 (m, 1H), 2.05 – 1.92 (m, 1H), 1.93 – 1.79 (m, 3H), 1.65 (d, *J* = 13.7 Hz, 1H), 1.56 (td, *J* = 14.3, 4.8 Hz, 1H), 1.45 (d, *J* = 13.7 Hz, 1H).
^{13}C NMR (126 MHz, $CDCl_3$) δ 208.68, 163.18, 149.13, 132.96, 132.85, 125.50, 119.03, 116.78, 110.25, 58.81, 56.50, 40.98, 40.22, 39.84, 36.58, 35.67, 33.53, 24.59, 20.58.
HRMS (ESI) *m/z* calculated for $C_{19}H_{25}N_2O_6S$ $[M+H]^+$: 409.1428, found: 409.1420.

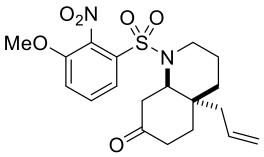

3-methoxy-2-nitrobenzenesulfonamide 26f
Isolated as orange foam, 72%yield.
R_f (50% EtOAc in cyclohexane): 0.25 (visualized by UV and $KMnO_4$ stain).
^{1}H NMR (500 MHz, $CDCl_3$) δ 7.54 (t, J = 8.2 Hz, 1H), 7.45 (dd, J = 8.2, 1.2 Hz, 1H), 7.28 – 7.22 (m, 1H), 5.70 (dddd, J = 16.8, 10.1, 8.3, 6.7 Hz, 1H), 5.12 – 5.01 (m, 2H), 3.98 – 3.84 (m, 6H), 2.99 (td, J = 13.2, 3.3 Hz, 1H), 2.72 (dd, J = 14.6, 12.3 Hz, 1H), 2.52 (dd, J = 14.1, 8.3 Hz, 1H), 2.35 (td, J = 14.6, 6.2 Hz,

1H), 2.23 (ddt, J = 15.2, 5.0, 2.6 Hz, 1H), 2.15 (ddd, J = 14.6, 5.4, 2.3 Hz, 1H), 2.00 – 1.87 (m, 2H), 1.88 – 1.75 (m, 2H), 1.63 (dt, J = 12.9, 2.7 Hz, 1H), 1.57 (td, J = 14.3, 5.0 Hz, 1H), 1.48 – 1.39 (m, 1H).
^{13}C NMR (126 MHz, $CDCl_3$) δ 208.43, 151.56, 137.88, 133.98, 132.87, 131.27, 120.55, 119.08, 116.95, 58.73, 57.13, 40.87, 39.96, 39.65, 36.50, 35.56, 33.35, 24.44, 20.54.
HRMS (ESI) *m/z* calculated for $C_{19}H_{25}N_2O_6S$ [M+H]$^+$: 409.1428, found: 409.1419.

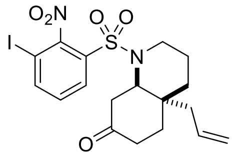

3-iodo-2-nitrobenzenesulfonamide 26g
Isolated as yellow foam, 30% yield.
R$_f$ (50% EtOAc in cyclohexane): 0.53 (visualized by UV and $KMnO_4$ stain).
^{1}H NMR (500 MHz, $CDCl_3$) δ 8.09 (dd, *J* = 8.0, 1.2 Hz, 1H), 7.93 (dd, *J* = 8.0, 1.2 Hz, 1H), 7.34 (t, *J* = 8.0 Hz, 1H), 5.69 (dddd, *J* = 16.9, 10.2, 8.4, 6.7 Hz, 1H), 5.10 (dd, *J* = 10.2, 1.8 Hz, 1H), 5.06 (dd, *J* = 16.9, 1.8 Hz, 1H), 3.91 – 3.80 (m, 2H), 3.02 (td, *J* = 13.2, 3.2 Hz, 1H), 2.76 (dd, *J* = 14.6, 12.4 Hz, 1H), 2.50 (dd, *J* = 14.1, 8.4 Hz, 1H), 2.36 (td, *J* = 14.6, 6.2 Hz, 1H), 2.29 – 2.21 (m, 1H), 2.20 – 2.12 (m, 1H), 2.02 – 1.78 (m, 4H), 1.70 – 1.61 (m, 1H), 1.57 (td, *J* = 14.3, 5.0 Hz, 1H), 1.49 – 1.41 (m, 1H).
^{13}C NMR (126 MHz, $CDCl_3$) δ 208.12, 151.32, 144.64, 134.57, 132.65, 131.61, 129.82, 119.24, 88.40, 58.97, 40.91, 40.24, 39.83, 36.50, 35.64, 33.41, 24.38, 20.49.
HRMS (ESI) *m/z* calculated for $C_{18}H_{22}N_2O_5SI$ [M+H]$^+$: 505.0289, found: 505.0271.

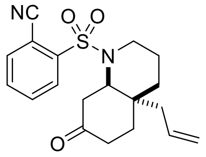

2-cyanobenzenesulfonamide 26h
Isolated as orange oil, 60% yield.
R$_f$ (50% EtOAc in cyclohexane): 0.42 (visualized by UV and $KMnO_4$ stain).
^{1}H NMR (500 MHz, $CDCl_3$) δ 8.09 (dd, *J* = 7.9, 1.3 Hz, 1H), 7.86 (dd, *J* = 7.6, 1.4 Hz, 1H), 7.75 (td, *J* = 7.9, 1.4 Hz, 1H), 7.68 (td, *J* = 7.6, 1.3 Hz, 1H), 5.60 (dddd, *J* = 16.9, 10.1, 8.3, 6.7 Hz, 1H), 5.09 – 4.98 (m, 2H), 4.21 (dd, *J* = 13.5, 5.1 Hz, 1H), 3.79 (dd, *J* = 12.3, 5.4 Hz, 1H), 3.11 (td, *J* = 13.5, 3.3 Hz, 1H), 2.78 (dd, *J* = 14.5, 12.2 Hz, 1H), 2.48 (dd, *J* = 14.1, 8.3 Hz, 1H), 2.36 (td, *J* = 14.7, 6.2 Hz, 1H), 2.21 (dddd, *J* = 20.5, 14.3, 5.2, 2.4 Hz, 2H), 2.06 – 1.92 (m, 1H), 1.87 (dd, *J* = 13.8, 5.0 Hz, 2H), 1.83 – 1.76 (m, 1H), 1.71 – 1.65 (m, 1H), 1.53 (td, *J* = 14.3, 4.8 Hz, 1H), 1.44 (ddt, *J* = 14.7, 5.1, 2.3 Hz, 1H).
^{13}C NMR (126 MHz, $CDCl_3$) δ = 208.26, 143.38, 135.74, 133.30, 132.81, 132.62, 130.18, 119.18, 116.38, 110.28, 58.77, 40.99, 40.22, 40.11, 36.49, 35.64, 33.39, 24.60, 20.48.
HRMS (ESI) *m/z* calculated for $C_{19}H_{23}N_2O_3S$ [M+H]$^+$: 359.1424, found: 359.1433.

Optimized nitroaryl transfer/acylation cascade procedures

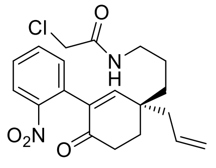

(*S*)-2-(2-nitrophenyl)cyclohexenone 25a
*From open sulfonamide **16**:*
To a solution of *ortho*-nitrobenzenesulfonamide **16** (2.03 g, 5.36 mmol, 1 eq.) in acetone (50 mL) was added caesium carbonate (3.42 g, 10.5 mmol, 2.0 eq.) and the resulting suspension was stirred vigorously at reflux for 18 h. The reaction mixture was allowed to room temperature and chloroacetyl chloride (4.2 mL, 53 mmol, 10 eq.) in acetone (40 mL) was added dropwise, after which the mixture was stirred for another 18 h at room temperature. The reaction mixture was concentrated *in vacuo* and water (50 mL) and DCM (50 mL) were added. The organic layer was collected, and the aqueous layer was extracted twice more with DCM (50 mL). The combined organic layers were washed with brine and dried over magnesium sulphate. Evaporation of all volatiles *in vacuo* and purification by flash column chromatography (silica gel, 0.5% acetic acid and 30% EtOAc in cyclohexane) provided the product as a slightly yellow oil (1.0 g, 2.6 mmol, 48%).
*From closed sulfonamide **26a**:*
To a solution of *ortho*-nitrobenzene sulfonamide **26a** (378 mg, 1.00 mmol, 1 eq.) in acetone (15 mL) was added caesium carbonate (652 g, 2.00 mmol, 2.0 eq.) and the resulting suspension was stirred vigorously at reflux for 18 h. The reaction mixture was allowed to room temperature and pyridine (0.81 mL, 10 mmol, 10 eq.) and chloroacetyl chloride (0.48 mL, 6.0 mmol, 6.0 eq.) were added dropwise, after which the mixture was stirred for another 18 h at room temperature. The reaction mixture was concentrated *in vacuo* and water (30 mL) and DCM (30 mL) were added. The organic layer was collected, and the aqueous layer was extracted twice more with DCM (30 mL). The combined organic layers were washed with brine and dried over magnesium sulphate. Evaporation of all volatiles *in vacuo* and purification by flash column chromatography (silica gel, 0.5% acetic acid and 30% EtOAc in cyclohexane) provided the product as a slightly yellow oil (188 mg, 0.48 mmol, 48%).
R$_f$ (50% EtOAc in cyclohexane): 0.26 (visualized by UV and $KMnO_4$ stain).
^{1}H NMR (600 MHz, $CDCl_3$) δ 8.01 (dd, *J* = 7.9, 1.3 Hz, 1H), 7.59 (ddd, *J* = 7.9, 7.6, 1.3 Hz, 1H), 7.47 (td, *J* = 7.9, 1.5 Hz, 1H), 7.21 (dd, *J* = 7.6, 1.5 Hz, 1H), 6.71 (br. s, 1H), 6.65 (s, 1H), 5.82 (ddt, *J* = 17.6, 10.3, 7.4 Hz, 1H), 5.22 – 5.12 (m, 2H), 4.02 (s, 2H), 3.35 – 3.28 (m, 2H), 2.64 – 2.52 (m, 2H), 2.35 (d, *J* = 7.4 Hz, 2H), 2.00 (t, *J* = 6.9 Hz, 2H), 1.71 – 1.52 (m, 4H).
^{13}C NMR (151 MHz, $CDCl_3$) δ 196.28, 166.11, 153.21, 148.63, 138.41, 133.52, 133.13, 132.13, 131.80, 129.06, 124.37, 119.30, 42.73, 42.58, 40.27, 38.89, 35.20, 34.25, 30.85, 24.33.
HRMS (ESI) *m/z* calculated for $C_{20}H_{24}N_2O_4Cl$ [M+H]$^+$: 391.1419, found: 391.1420.
$[\alpha]_D^{20}$ +8.0 (c = 2.0, $CHCl_3$).

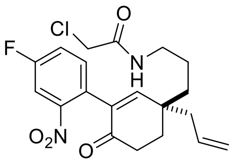

2-(4-fluoro-2-nitrophenyl)cyclohexenone 25b

To a solution of *ortho*-nitrobenzene sulfonamide **26b** (44 mg, 0.11 mmol, 1 eq.) in acetone (2 mL) was added caesium carbonate (72 mg, 0.22 mmol, 2.0 eq.) and the resulting suspension was stirred vigorously at reflux for 3 h. The reaction mixture was allowed to room temperature and pyridine (89 µL, 1.1 mmol, 10 eq.) and chloroacetyl chloride (80 µL, 1.0 mmol, 10 eq.) were added dropwise, after which the mixture was stirred for another 18 h at room temperature. The reaction mixture was concentrated *in vacuo* and aqueous sodium hydroxide (1M, 5 mL) and DCM (5 mL) were added. The organic layer was collected, and the aqueous layer was extracted thrice more with DCM (5 mL). The combined organic layers were washed with aqueous hydrochloric acid (1 M), brine and dried over magnesium sulphate. Evaporation of all volatiles *in vacuo* and purification by flash column chromatography (silica gel, 30% EtOAc in toluene) provided the product as a yellow oil (23 mg, 55 µmol, 50%).

R_f (50% EtOAC in cyclohexane): 0.28 (visualized by UV and $KMnO_4$ stain).

^{1}H NMR (500 MHz, $CDCl_3$) δ 7.78 (dd, *J* = 8.3, 2.6 Hz, 1H), 7.34 (td, *J* = 8.5, 7.4, 2.6 Hz, 1H), 7.21 (dd, *J* = 8.5, 5.5 Hz, 1H), 6.68 – 6.63 (m, 2H), 5.82 (dddd, *J* = 17.6, 10.3, 7.4 Hz, 1H), 5.22 – 5.13 (m, 2H), 4.06 (s, 2H), 3.40 – 3.31 (m, 2H), 2.67 – 2.53 (m, 2H), 2.36 (d, *J* = 7.4 Hz, 2H), 2.02 (t, *J* = 6.9 Hz, 2H), 1.73 – 1.57 (m, 3H).

^{13}C NMR (126 MHz, $CDCl_3$) δ 196.17, 166.14, 161.92 (d, *J* = 252.0 Hz), 153.49, 149.14, 137.69, 133.30 (d, *J* = 7.8 Hz), 133.06, 128.25 (d, *J* = 3.7 Hz), 120.71 (d, *J* = 21.2 Hz), 119.50, 112.35 (d, *J* = 26.7 Hz), 42.81, 42.61, 40.32, 38.99, 35.23, 34.23, 30.90, 24.44.

^{19}F NMR (470 MHz, $CDCl_3$) δ –109.96

HRMS (ESI) *m/z* calculated for $C_{20}H_{23}N_2O_4FCl$ $[M+H]^+$: 409.1325, found: 409.1306.

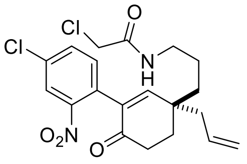

2-(4-chloro-2-nitrophenyl)cyclohexenone 25c

To a solution of *ortho*-nitrobenzene sulfonamide **26c** (45 mg, 0.11 mmol, 1 eq.) in acetone (2 mL) was added caesium carbonate (72 mg, 0.22 mmol, 2.0 eq.) and the resulting suspension was stirred vigorously at reflux for 3 h. The reaction mixture was allowed to room temperature and pyridine (89 µL, 1.1 mmol, 10 eq.) and chloroacetyl chloride (80 µL, 1.0 mmol, 10 eq.) were added dropwise, after which the mixture was stirred for another 18 h at room temperature. The reaction mixture was concentrated *in vacuo* and aqueous sodium hydroxide (1M, 5 mL) and DCM (5 mL) were added. The organic layer was collected, and the aqueous layer was extracted thrice more with DCM (5 mL). The combined organic layers were washed with aqueous hydrochloric acid (1 M), brine and dried over magnesium sulphate. Evaporation of all volatiles *in vacuo* and purification by flash column chromatography (silica gel, 30% EtOAc in toluene) provided the product as a yellow oil (23 mg, 55 µmol, 60%).

R_f (50% EtOAC in cyclohexane): 0.34 (visualized by UV and $KMnO_4$ stain).

^{1}H NMR (500 MHz, $CDCl_3$) δ 8.03 (d, *J* = 2.1 Hz, 1H), 7.58 (dd, *J* = 8.2, 2.1 Hz, 1H), 7.17 (d, *J* = 8.2 Hz, 1H), 6.66 (s, 2H), 5.82 (ddt, *J* = 17.5, 10.2, 7.4 Hz, 1H), 5.22 – 5.13 (m, 2H), 4.06 (s, 2H), 3.38 – 3.30 (m, 2H), 2.60 (dd, *J* = 9.5, 6.8 Hz, 2H), 2.36 (d, *J* = 7.4 Hz, 2H), 2.02 (t, *J* = 6.9 Hz, 2H), 1.72 – 1.55 (m, 4H).

^{13}C NMR (126 MHz, $CDCl_3$) δ 196.01, 166.14, 153.63, 148.98, 137.61, 134.92, 133.54, 133.01, 132.86, 130.53, 124.74, 119.54, 42.80, 42.59, 40.31, 39.02, 35.20, 34.20, 30.88, 24.43.

HRMS (ESI) *m/z* calculated for $C_{20}H_{23}N_2O_4Cl_2$ $[M+H]^+$: 425.1029, found: 425.1021.

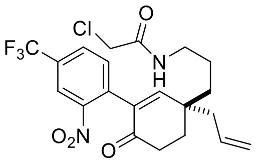

2-(2-nitro-4-(trifluoromethyl)phenyl)cyclohexenone 25d

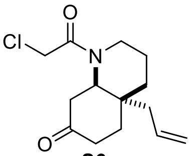

S6

To a solution of *ortho*-nitrobenzene sulfonamide **26d** (134 mg 0.30 mmol, 1 eq.) in acetone (6 mL) was added caesium carbonate (195 mg, 0.20 mmol, 2.0 eq.) and the resulting suspension was stirred vigorously at reflux for 2 h. The reaction mixture was allowed to room temperature and chloroacetyl chloride (240 µL, 1.0 mmol, 10 eq.) was added dropwise, after which the mixture was stirred for another 18 h at room temperature. The reaction mixture was concentrated *in vacuo* and water (15 mL) and DCM (15 mL) were added. The organic layer was collected, and the aqueous layer was extracted twice more with DCM (10 mL). The combined organic layers were washed with brine and dried over magnesium sulphate. Evaporation of all volatiles *in vacuo* and purification by flash column chromatography (silica gel, 0.5% acetic acid and 40% EtOAc in cyclohexane) provided the product as a yellow oil (32.5 mg, 24%), inseparable from compound **S6** (molar ratio 1:0.26, mass ratio: 1:0.15), corrected product yield: 21%.

R_f (50% EtOAC in cyclohexane): 0.39 (visualized by UV and $KMnO_4$ stain).

^{1}H NMR (600 MHz, $CDCl_3$) δ 8.28 (d, *J* = 1.8 Hz, 1H), 7.85 (dd, *J* = 7.9, 1.8 Hz, 1H), 7.38 (d, *J* = 7.9 Hz, 1H), 6.72 (s, 1H), 6.70 (s, 1H), 5.82 (ddt, *J* = 17.4, 10.2, 7.4 Hz, 1H), 5.22 – 5.13 (m, 2H), 4.04 (s, 2H), 3.39 – 3.28 (m, 2H), 2.68 – 2.54 (m, 2H), 2.37 (d, *J* = 7.4 Hz, 2H), 2.03 (t, *J* = 6.9 Hz, 2H), 1.73 – 1.57 (m, 4H).

^{13}C NMR (151 MHz, $CDCl_3$) δ 195.73, 166.19, 154.34, 148.72, 137.48, 135.60, 132.92, 132.87, 132.75, 131.69 (q, *J* = 34.3 Hz), 129.97 (q, *J* = 3.4 Hz), 122.87 (q, *J* = 272.7 Hz), 121.77 (q, *J* = 3.9 Hz), 119.61, 53.85, 42.76, 42.50, 40.28, 39.09, 35.11, 34.12, 30.79, 24.39.

^{19}F NMR (470 MHz, $CDCl_3$) δ –62.88.

HRMS (ESI) *m/z* calculated for $C_{21}H_{23}N_2O_4F_3Cl$ $[M+H]^+$: 459.1293, found: 459.1294.

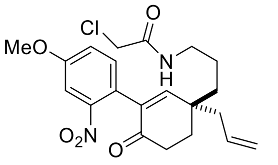

2-(4-methoxy-2-nitrophenyl)cyclohexenone 25e

To a solution of *ortho*-nitrobenzene sulfonamide **26e** (41 mg, 0.10 mmol, 1 eq.) in acetone (2 mL) was added caesium carbonate (65 mg, 0.20 mmol, 2.0 eq.) and the resulting suspension was stirred vigorously at reflux for 3 days. The reaction mixture was allowed to room temperature and chloroacetyl chloride (80 µL, 1.0 mmol, 10 eq.) was added dropwise, after which the mixture was stirred for another 18 h at room temperature. The reaction mixture was concentrated *in vacuo* and water (5 mL) and DCM (5 mL) were added. The organic layer was collected, and the aqueous layer was extracted twice more with DCM (5 mL). The combined organic layers were washed with brine and dried over magnesium sulphate. Evaporation of all volatiles *in vacuo* and purification by flash column chromatography (silica gel, 0.5 % acetic acid and 50% EtOAc in cyclohexane) provided the product as a yellow oil (23.5 mg, 56%).

R_f (50% EtOAC in cyclohexane): 0.19 (visualized by UV and $KMnO_4$ stain).

^{1}H NMR (500 MHz, $CDCl_3$) δ 7.56 (d, *J* = 2.4 Hz, 1H), 7.14 (dd, *J* = 8.4, 2.4 Hz, 1H), 7.11 (d, *J* = 8.4 Hz, 1H), 6.66 (br. s, 1H), 6.60 (s, 1H), 5.82 (ddt, *J* = 16.6, 10.3, 7.4 Hz, 1H), 5.21 – 5.12 (m, 2H), 4.05 (s, 2H), 3.88 (s, 3H), 3.39 – 3.27 (m, 2H), 2.66 – 2.51 (m, 2H), 2.35 (d, *J* = 7.4 Hz, 2H), 2.01 (t, *J* = 6.9 Hz, 2H), 1.72 – 1.54 (m, 4H).

^{13}C NMR (126 MHz, $CDCl_3$) δ 196.62, 166.11, 159.87, 152.69, 149.22, 138.21, 133.25, 132.64, 124.33, 119.95, 119.32, 109.35, 56.08, 42.81, 42.71, 40.35, 38.90, 35.32, 34.34, 30.97, 24.43.

HRMS (ESI) *m/z* calculated for $C_{21}H_{26}N_2O_5Cl$ $[M+H]^+$: 421.1525, found: 421.1510.

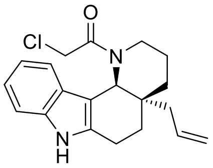

Tetracyclic indole 27[3]

A nitrogen-flushed and flame-dried Schlenk flask equipped with reflux condenser was charged with nitroaryl chloroacetamide **25a** (0.99 g, 2.5 mmol, 1 eq.) and thoroughly degassed acetic acid (51 mL). Iron dust (0.71 g, 13 mmol, 5 eq.) was added and the reaction mixture was heated to 85 °C and stirred vigorously for 18 h. The mixture was then allowed to room temperature and all volatiles were removed *in vacuo*. The residue was taken up in water and ethyl acetate, extracting the aqueous layer twice more with ethyl acetate. The combined organic layers were washed with brine and dried over sodium sulphate. After evaporation of all solvents, the product was obtained by flash column chromatography (silica gel, 0–20% EtOAc in cyclohexane) as a colourless crystalline powder (0.54 g, 1.58 mmol, 62%) that could be recrystallized from acetone.

R_f (30% EtOAc in cyclohexane): 0.43 (visualized by UV and $Ce(SO_4)_2$ stain).

^{1}H NMR (600 MHz, $CDCl_3$, rotamers observed in a ~0.7:0.3 ratio) δ 8.32 (s, 0.3H), 8.11 (s, 0.7H), 7.34 – 7.24 (m, 1.7H), 7.20 (d, *J* = 8.0 Hz, 0.3H), 7.14 – 7.06 (m, 1H), 7.05 – 6.96 (m, 1H), 5.91 – 5.79 (m, 1H), 5.75 (s, 0.7H), 5.19 – 5.07 (m, 2H), 4.77 (s, 0.3H), 4.54 (ddd, *J* = 13.2, 4.4, 1.9 Hz, 0.3H), 4.42 (d, *J* = 13.0 Hz, 0.3H), 4.39 (d, *J* = 13.0 Hz, 0.3H), 4.31 (d, *J* = 11.9 Hz, 0.7H), 4.25 (d, *J* = 11.9 Hz, 0.7H), 3.61 (ddd, *J* = 13.8, 4.1, 2.1 Hz, 0.7H), 2.89 (td, *J* = 13.4, 2.9 Hz, 0.7H), 2.82 – 2.70 (m, 1.3H), 2.69 – 2.58 (m, 1H), 2.43 (td, *J* = 13.2, 3.1 Hz, 0.3H), 2.37 (dd, *J* = 14.0, 8.1 Hz, 0.7H), 2.21 (dd, *J* = 14.0, 7.0 Hz, 0.7H), 2.12 (dd, *J* = 14.0, 7.4 Hz, 0.3H), 1.95 (ddd, *J* = 13.8, 5.7, 1.9 Hz, 0.3H), 1.84 (dt, *J* = 13.2, 4.0 Hz, 0.7H), 1.82 – 1.68 (m, 2.7H), 1.62 (td, *J* = 13.3, 12.8, 4.0 Hz, 0.3H), 1.45 – 1.38 (m, 0.6H), 1.38 – 1.31 (m, 1.4H).

^{13}C NMR (151 MHz, $CDCl_3$, rotamers observed in a ~0.7:0.3 ratio) δ 166.22, 166.10, 136.36, 136.36, 135.17, 135.05, 134.04, 133.54, 126.26, 125.98, 121.59, 121.41, 120.27, 119.77, 118.87, 118.47, 118.45, 118.17, 110.97, 110.67, 107.58, 106.84, 59.10, 53.24, 42.30, 42.04, 41.82, 40.81, 40.51, 37.87, 37.35, 36.84, 32.20, 32.16, 25.54, 24.70, 21.68, 20.26, 19.65, 19.57.

Mp 174 °C (decomp.).

HRMS (ESI) *m/z* calculated for $C_{20}H_{24}N_2OCl$ $[M+H]^+$: 343.1572, found: 343.1570.

$[\alpha]_D^{20}$ –132 (c = 2.0, $CHCl_3$).

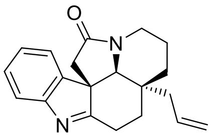

Pentacyclic indolenine 14[4]

Indole **27** (40 mg, 0.12 mmol, 1 eq.) and sodium iodide (175 mg, 1.17 mmol, 10 eq.) were dissolved in acetone (6 mL) and the mixture was heated to reflux for 2 h, resulting in an off-white precipitate. The mixture was allowed to room temperature and water (6 mL) and EtOAc (6 mL) were added. The organic layer was drained off and the aqueous layer was extracted once more with EtOAc (6 mL). The combined organic layers were washed with brine and dried over sodium sulphate. Evaporation of all volatiles yielded the iodide as a yellow foam that was immediately dissolved in THF (6 mL) and cooled to 0 °C. Then, in the dark, silver triflate (76 mg, 0.29 mmol, 2.5 eq.) was added in one portion and the mixture was stirred at 0 °C for 0.5 h. Water (6 mL) and EtOAc (12 mL) were added, and the aqueous layer was quickly extracted (to avoid excessive precipitation) with EtOAc (2 x 6 mL). The combined organic layers were washed with brine and dried over sodium sulphate. Evaporation of all volatiles yielded the indolenine as a pinkish foam that could be purified by flash column chromatography (silica gel, EtOAc) to provide a white foam with minor inseparable impurities (29 mg, 81%). The product showed quick (<1 week) decomposition, even when stored under argon at –20 °C.

R_f (EtOAc): 0.27 (visualized by UV and $Ce(SO_4)_2$ stain).

^{1}H NMR (500 MHz, $CDCl_3$) δ 7.55 (d, *J* = 7.7 Hz, 1H), 7.40 – 7.29 (m, 2H), 7.23 (td, *J* = 7.5, 1.0 Hz, 1H), 5.60 (ddt, *J* = 17.4, 10.1, 7.4 Hz, 1H), 5.01 – 4.95 (m, 1H), 4.85 – 4.77 (m, 1H), 4.33 (ddt, *J* = 13.0, 4.5, 2.0 Hz, 1H), 3.66 (d, *J* = 2.0 Hz, 1H), 3.03 – 2.95 (m, 2H), 2.83 – 2.71 (m, 2H), 2.55 (dd, *J* = 18.2, 1.5 Hz, 1H), 2.28 – 2.14 (m, 1H), 1.84 – 1.72 (m, 2H), 1.68 – 1.41 (m, 5H).

^{13}C NMR (126 MHz, $CDCl_3$) δ 186.76, 170.46, 154.60, 145.49, 132.08, 128.67, 126.35, 121.06, 120.60, 119.33, 69.34, 53.96, 41.32, 40.85, 38.80, 37.35, 34.46, 24.43, 24.21, 20.32.

HRMS (ESI) *m/z* calculated for $C_{20}H_{23}N_2O$ $[M+H]^+$: 307.1805, found: 307.1802.

$[\alpha]_D^{20}$ –549 (c = 0.10, $CHCl_3$).

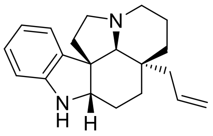

Pentacyclic indoline 28

Indole **27** (150 mg, 0.44 mmol, 1 eq.) and sodium iodide (656 mg, 4.37 mmol, 10 eq.) were dissolved in acetone (25 mL) and the mixture was heated to reflux for 2 h, resulting in an off-white precipitate. The mixture was allowed to room temperature and water (25 mL) and EtOAc (25 mL) were added. The organic layer was drained off and the aqueous layer was extracted once more with EtOAc (25 mL). The combined organic layers were washed with brine and dried over sodium sulphate. Evaporation of all volatiles yielded the iodide as a yellow foam that was immediately dissolved in THF (25 mL) and cooled to 0 °C. Then, in the dark, silver triflate (281 mg, 1.09 mmol, 2.5 eq.) was added in one portion and the mixture was stirred at 0 °C for 0.5 h. Water (25 mL) and EtOAc (50 mL) were added, and the aqueous layer was quickly extracted (to avoid excessive precipitation) with EtOAc (2 x 25 mL). The combined organic layers were washed with brine and dried over sodium sulphate. Evaporation of all volatiles *in vacuo* yielded the indolenine as a pinkish foam. The crude indolenine **14** was dissolved in dry THF (10 mL) and cooled to 0 °C. Lithium aluminium hydride (66 mg, 1.74 mmol, 4 eq.) was added to the solution and the resulting black mixture was stirred at reflux for 18 h. The reaction was cooled to 0 °C and water (66 µL), aq. sodium hydroxide solution (15%, 66 µL) and water (198 µL) were added sequentially. After 15 minutes of vigorous stirring, sodium sulphate was added after which the mixture was stirred another 15 minutes. The reaction mixture was filtered over celite, and the residue was washed extensively with THF. Evaporation of all volatiles and purification by flash column chromatography (silica gel, 1% MeOH and 1% Et_3N in DCM) yielded the product as white waxy solid (115 mg, 0.39 mmol, 89% over three steps).

R_f (10 % MeOH in DCM): 0.39 (visualized by UV and $Ce(SO_4)_2$ stain).

^{1}H NMR (500 MHz, $CDCl_3$) δ 7.08 (dd, *J* = 7.4, 1.0 Hz, 1H), 7.01 (ddd, *J* = 7.7, 7.4, 1.3 Hz, 1H), 6.73 (td, *J* = 7.4, 1.0 Hz, 1H), 6.64 (app. d, *J* = 7.7 Hz, 1H), 5.67 (ddt, *J* = 17.0, 10.1, 7.5 Hz, 1H), 4.93 (dd, *J* = 10.1, 2.4 Hz, 1H), 4.82 (ddt, *J* = 17.0, 2.4, 1.4 Hz, 1H), 3.52 (dd, *J* = 11.0, 6.2 Hz, 1H), 3.16 – 3.08 (m, 1H), 3.08 – 3.02 (m, 1H), 2.36 – 2.16 (m, 4H), 2.06 – 1.91 (m, 2H), 1.79 – 1.41 (m, 7H), 1.20 (td, *J* = 13.5, 4.5 Hz, 1H), 1.03 (dt, *J* = 13.7, 3.7 Hz, 1H).

^{13}C NMR (151 MHz, $CDCl_3$) δ 149.60, 135.38, 134.36, 127.37, 122.98, 119.25, 117.29, 110.56, 70.87, 70.75, 65.57, 53.92, 53.07, 42.41, 38.78, 35.47, 28.14, 26.65, 23.99, 21.89.

HRMS (ESI) *m/z* calculated for $C_{20}H_{27}N_2$ $[M+H]^+$: 295.2169, found: 295.2170.

$[\alpha]_D^{20}$ +6.0 (c = 1.0, $CHCl_3$).

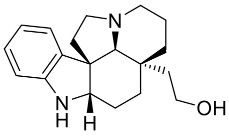

(–)-limaspermidine

(–)-Limaspermidine (*ent-2*)

Pentacycle **28** (50 mg, 0.17 mmol, 1 eq.) was dissolved in MeOH (1 mL) and HCl in MeOH (0.5 M, 0.86 mL, 2.5 eq.) was added. The mixture was stirred at room temperature for 10 minutes after which all volatiles were removed thoroughly *in vacuo* yielding the double HCl salt of **28** (61.4 mg). This salt was dissolved in DCM/methanol (1:1, 8 mL) and the mixture was cooled to –78 °C. Ozone was bubbled through the solution for 2 h, until full conversion of the starting material was observed on TLC. Nitrogen was then bubbled through the intensely purple solution for 10 min, and sodium borohydride (163 mg, 4.3 mmol, 10 eq.) was added in one portion at –78 °C. The reaction mixture was allowed to warm to room temperature and stirred for 1 h. All volatiles were removed *in vacuo* and water and DCM were added. The aqueous layer was extracted twice with DCM and the combined organic layers were washed with brine and dried over sodium sulphate. Evaporation of all volatiles and purification by flash column chromatography (silica gel, cyclohexane/EtOAc/Et_3N 8/8/1) yielded the title compound as a white solid (30 mg, 0.10 mmol, 59%).

R_f (cyclohexane/EtOAc/Et_3N 8:8:1): 0.29 (visualized by UV and $Ce(SO_4)_2$ stain).

^{1}H NMR (600 MHz, $CDCl_3$) δ 7.08 (d, *J* = 7.4 Hz, 1H), 7.00 (d, *J* = 7.4 Hz, 1H), 6.72 (t, *J* = 7.4 Hz, 1H), 6.63 (d, *J* = 7.4 Hz, 1H), 3.60 (td, *J* = 10.1, 5.4 Hz, 1H), 3.55 – 3.47 (m, 2H), 3.18 – 3.07 (m, 1H), 3.07 – 2.99 (m, 1H), 2.33 – 2.21 (m, 3H), 2.04 (td, *J* = 13.9, 3.5 Hz, 1H), 1.97 (td, *J* = 11.7, 2.9 Hz, 1H), 1.80 – 1.71 (m, 2H), 1.69 (ddd, *J* = 13.2, 6.5, 3.5 Hz, 1H), 1.67 – 1.59 (m, 1H), 1.54 – 1.42 (m, 3H), 1.26 (td, *J* = 13.6, 4.6 Hz, 1H), 1.17 (ddd, *J* = 14.3, 9.3, 5.4 Hz, 1H), 1.02 (dt, *J* = 13.9, 3.7 Hz, 1H).

^{13}C NMR (151 MHz, $CDCl_3$) δ 149.60, 135.40, 127.48, 122.90, 119.30, 110.63, 70.86, 65.48, 58.69, 53.88, 53.58, 52.99, 40.62, 38.69, 35.61, 35.53, 28.36, 24.39, 21.83.

HRMS (ESI) *m/z* calculated for $C_{19}H_{27}N_2O$ $[M+H]^+$: 299.2118, found: 299.2108.

$[\alpha]_D^{20}$ –20 (c = 0.5, $CHCl_3$).

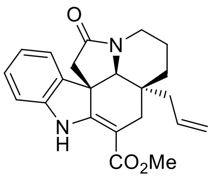

Pentacyclic methyl ester S7[5]

Thoroughly dried crude indolenine **14** (124 mg, 0.40 mmol, 1 eq.) was dissolved in dry THF (2 mL) and cooled to –78 °C. Freshly prepared LDA [prepared by adding *n*-buthyllithium (1.6 M in hexanes, 253 µL, 0.40 mmol, 1.0 eq.) to diisopropylamine (59 µL, 0.42 mmol, 1.05 eq.) in THF (2.5 mL) at 0 °C] was added dropwise over 5 minutes. The resulting yellow solution was stirred at –78 °C for 1.5 h, after which methyl cyanoformate (64 µL, 0.80 mmol, 2.0 eq.) was added by quick injection. After 1 h at –78 °C, the reaction was quenched at –78 °C with saturated aqueous ammonium chloride and allowed to room temperature. The reaction mixture was extracted three times with ethyl acetate; the combined organic layers were washed with brine and dried over sodium sulphate. The mixture was purified by flash column chromatography (silica gel, 25–50–100% EtOAc in cyclohexane) to provide the product (54 mg, 0.15 mmol, 37%) as a white powder, as well as the starting indolenine (36 mg, 0.12 mmol, 30%).

R_f (60% EtOAc in cyclohexane): 0.47 (visualized by UV and $Ce(SO_4)_2$ stain).

^{1}H NMR (500 MHz, $CDCl_3$) δ 8.99 (s, 1H), 7.22 – 7.15 (m, 2H), 6.93 (t, *J* = 7.5 Hz, 1H), 6.85 (d, *J* = 7.8 Hz, 1H), 5.57 (ddt, *J* = 17.1, 10.1, 7.4 Hz, 1H), 4.96 (dd, *J* = 10.1, 2.2 Hz, 1H), 4.77 (dd, *J* = 17.1, 2.2 Hz, 1H), 4.33 – 4.24 (m, 1H), 3.79 – 3.71 (m, 4H), 2.87 – 2.75 (m, 2H), 2.58 (d, *J* = 17.8 Hz, 1H), 2.34 (d, *J* = 15.7 Hz, 1H), 2.28 (d, *J* = 15.7 Hz, 1H), 1.87 – 1.78 (m, 2H), 1.71 – 1.59 (m, 3H), 1.54 (dd, *J* = 13.9, 7.5 Hz, 1H).
^{13}C NMR (126 MHz, $CDCl_3$) δ 170.63, 168.69, 164.91, 143.24, 136.08, 132.53, 128.77, 121.75, 121.55, 118.54, 109.80, 91.98, 67.83, 51.25, 48.71, 47.98, 41.99, 40.38, 38.11, 33.72, 26.06, 20.33.
HRMS (ESI) *m/z* calculated for $C_{22}H_{25}N_2O_3$ $[M+H]^+$: 365.1860, found: 365.1867.
$[\alpha]_D^{20}$ –164 (c = 1.0, $CHCl_3$).

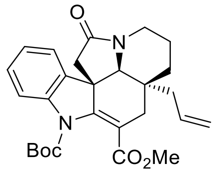

Boc-protected pentacyclic methyl ester 29

Pentacycle **S7** (109 mg, 0.30 mmol, 1 eq.) and DMAP (18.3 mg, 0.15 mmol, 0.5 eq.) were suspended in dry DMF (3 mL). The mixture was cooled to 0 °C and Boc_2O (350 mg, 1.60 mmol, 5.3 eq.) was added in one portion. The mixture was allowed to room temperature, whereupon all solids had dissolved, and stirred for 18 h. Another portion of DMAP (6 mg, 0.028 mmol, 0.1 eq.) and Boc_2O (100 mg, 0.82 mmol, 2.7 eq.) were added and the mixture was stirred for another hour. All volatiles were removed *in vacuo* and the product was obtained after purification by flash column chromatography (silica gel, 20–40% EtOAc in cyclohexane) as a colourless oil (140 mg, quant.).
R_f (50% EtOAc in cyclohexane): 0.48 (visualized by UV and $KMnO_4$ stain).
^{1}H NMR (500 MHz, CDCl3) δ 7.67 (d, *J* = 8.0 Hz, 1H), 7.29 – 7.22 (m, 1H), 7.13 (dd, *J* = 7.5, 1.4 Hz, 1H), 7.08 (td, *J* = 7.5, 1.0 Hz, 1H), 5.54 (dddd, *J* = 16.9, 10.2, 8.1, 6.9 Hz, 1H), 4.95 (dd, *J* = 10.2, 2.1 Hz, 1H), 4.75 (ddt, *J* = 16.9, 2.1, 1.4 Hz, 1H), 4.35 – 4.28 (m, 1H), 3.82 (d, *J* = 2.0 Hz, 1H), 3.73 (s, 3H), 2.86 (d, *J* = 17.8 Hz, 1H), 2.79 – 2.70 (m, 1H), 2.59 (d, *J* = 15.7 Hz, 1H), 2.47 (dd, *J* = 17.9, 1.7 Hz, 1H), 2.04 (dd, *J* = 15.7, 2.0 Hz, 1H), 1.92 (dd, *J* = 13.9, 8.1 Hz, 1H), 1.88 – 1.80 (m, 1H), 1.69 – 1.58 (m, 4H), 1.52 (s, 9H).
^{13}C NMR (126 MHz, CDCl3) δ 170.14, 167.44, 150.94, 148.41, 140.94, 136.12, 132.01, 128.47, 124.44, 120.73, 119.26, 116.38, 112.19, 82.98, 65.36, 51.76, 46.74, 46.63, 40.87, 40.07, 37.59, 33.53, 28.18, 27.75, 19.90.
HRMS (ESI) m/z calculated for $C_{27}H_{33}N_2O_5$ [M+H]+: 465.2384, found: 465.2379.
$[\alpha]_D^{20}$ –52 (c = 1.0, CHCl3).

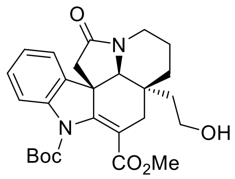

Boc-protected pentacyclic alcohol S8

Pentacycle **29** (44 mg, 95 µmol, 1 eq.) was dissolved in DCM/methanol (1:1, 5 mL) and cooled to –78 °C. Ozone was bubbled through the solution for 1 h, until full conversion of the starting material was observed. Nitrogen was then bubbled through the intensely purple solution for 10 min, and sodium borohydride (36 mg, 1.0 mmol, 10 eq.) was added in one portion at –78 °C. The reaction mixture was allowed to warm to room temperature and stirred for 2 h. All volatiles were removed *in vacuo* and water and DCM were added. The aqueous layer was extracted twice with DCM and the combined organic layers were washed with brine and dried over sodium sulphate. Evaporation of all volatiles and purification by flash column chromatography (silica gel, EtOAc) yielded the product as a white foam (43 mg, 0.12 mmol, 97%).
R_f (EtOAc): 0.28 (visualized by UV and $KMnO_4$ stain).
^{1}H NMR (500 MHz, $CDCl_3$) δ 7.63 (d, *J* = 8.0 Hz, 1H), 7.23 (dd, *J* = 8.0, 7.4 Hz, 1H), 7.13 (d, *J* = 7.4 Hz, 1H), 7.06 (t, *J* = 7.4 Hz, 1H), 4.32 – 4.25 (m, 1H), 3.81 (s, 1H), 3.72 (s, 3H), 3.52 – 3.38 (m, 2H), 2.83 (d, *J* = 17.8 Hz, 1H), 2.79 – 2.69 (m, 1H), 2.61 (d, *J* = 15.5 Hz, 1H), 2.44 (d, *J* = 17.8 Hz, 1H), 2.17 – 2.07 (m, 2H), 2.02 – 1.92 (m, 2H), 1.68 – 1.56 (m, 2H), 1.51 (s, 9H), 1.45 (dt, *J* = 13.6, 7.0 Hz, 1H), 1.03 (dt, *J* = 13.6, 6.4 Hz, 1H).
^{13}C NMR (126 MHz, $CDCl_3$) δ 170.19, 167.75, 150.92, 147.99, 140.87, 136.17, 128.46, 124.49, 120.74, 116.29, 112.27, 83.05, 65.97, 57.81, 51.91, 46.75, 46.73, 40.08, 38.79, 36.23, 34.01, 28.17, 27.62, 19.81.
HRMS (ESI) *m/z* calculated for $C_{26}H_{33}N_2O_6$ $[M+H]^+$: 469.2333, found: 469.2334.
$[\alpha]_D^{20}$ –29 (c = 1.0, $CHCl_3$).

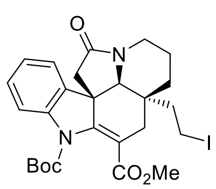

Boc-protected pentacyclic iodide 30

Alcohol **S8** (57 mg, 0.12 mmol, 1 eq.) was dissolved in dry DCM (1.2 mL) and cooled to 0 °C. Imidazole (25 mg, 0.36 mmol, 3.0 eq.), triphenyl phosphine (48 mg, 0.18 mmol, 1.5 eq.) and iodine (46 mg, 0.18 mmol, 1.5 eq.) were added sequentially. The reaction mixture was allowed to room temperature and stirred for 2 h. Water was added and the mixture was extracted three times with DCM. The combined organic layers were washed with brine and dried over sodium sulphate. Evaporation of all volatiles *in vacuo* and purification by flash column chromatography (silica gel, 1% MeOH in DCM) yielded the product as a white solid (60 mg, 0.10 mmol, 85%).
R_f (50% EtOAc in cyclohexane): 0.30 (visualized by UV and $KMnO_4$ stain).
^{1}H NMR (500 MHz, $CDCl_3$) δ 7.65 (d, *J* = 7.9 Hz, 1H), 7.30 – 7.23 (m, 1H), 7.19 – 7.06 (m, 2H), 4.33 – 4.26 (m, 1H), 3.78 – 3.73 (m, 4H), 2.97 (ddd, *J* = 12.5, 9.4, 4.6 Hz, 1H), 2.84 (d, *J* = 17.9 Hz, 1H), 2.76 (ddd, *J* = 12.3, 9.4, 5.2 Hz, 2H), 2.53 (d, *J* = 15.9 Hz, 1H), 2.47 (dd, *J* = 17.9, 1.4 Hz, 1H), 2.13 (dd, *J* = 15.9, 2.1 Hz, 1H), 1.94 – 1.88 (m, 1H), 1.83 (ddd, *J* = 14.0, 12.4, 5.2 Hz, 1H), 1.70 – 1.61 (m, 3H), 1.55 – 1.44 (m, 10H).

^{13}C NMR (126 MHz, $CDCl_3$) δ 170.08, 167.38, 150.85, 148.77, 140.79, 135.95, 128.67, 124.71, 120.67, 116.46, 111.02, 83.31, 65.72, 52.00, 46.74, 46.73, 41.79, 40.03, 39.60, 33.27, 28.17, 27.14, 19.78, –3.39.
HRMS (ESI) *m/z* calculated for $C_{26}H_{32}N_2O_5I$ $[M+H]^+$: 579.1350, found: 579.1353.
$[\alpha]_D^{20}$ –39 (c = 1.0, $CHCl_3$).

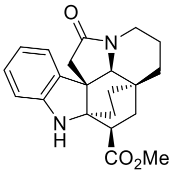

(–)-Kopsinilam (31)
A solution of iodide **30** (62 mg, 0.11 mmol, 1 eq.) in dry THF (2.6 mL) was added dropwise to a mixture of samarium (II) iodide (0.1M in THF, 10.7 mL, 1.07 mmol, 10 eq.) and HMPA (1.3 mL) at room temperature. After 2.5 h, the reaction was quenched by the addition of saturated aqueous ammonium chloride and water. The reaction mixture was extracted three times with ethyl acetate and the combined organic layers were washed with brine and dried over sodium sulphate. All volatiles were removed *in vacuo* (note: most of the HMPA had remained) and the resulting oil was dissolved in DCM (2 mL) and cooled to 0 °C. TFA (2 mL) was added and the reaction was allowed to room temperature. After 3 h, the reaction was quenched by the addition of saturated aqueous potassium carbonate (note: substantial gas evolution occurred) and water. The mixture was extracted three times with DCM and the combined organic layers were washed with brine and dried over sodium sulphate. Evaporation of all volatiles and purification by flash column chromatography (silica gel, 50–100% EtOAc in cyclohexane, loading the reaction mixture with cyclohexane) yielded the product as a white foam (34.4 mg, 0.098 mmol, 91%).
R_f (50% EtOAc in cyclohexane): 0.10 (visualized by UV and $Ce(SO_4)_2$ stain).
^{1}H NMR (500 MHz, $CDCl_3$) δ 7.08 – 7.00 (m, 2H), 6.77 (td, *J* = 7.5, 1.0 Hz, 1H), 6.69 (d, *J* = 7.7 Hz, 1H), 4.33 – 4.25 (m, 1H), 3.75 (s, 3H), 3.59 (d, *J* = 1.7 Hz, 1H), 3.41 (d, *J* = 18.6 Hz, 1H), 2.97 (t, *J* = 10.2 Hz, 1H), 2.81 – 2.71 (m, 1H), 2.34 (ddd, *J* = 13.7, 10.2, 3.0 Hz, 1H), 2.04 – 1.92 (m, 2H), 1.74 – 1.58 (m, 3H), 1.57 – 1.48 (m, 1H), 1.47 – 1.23 (m, 5H).
^{13}C NMR (126 MHz, $CDCl_3$) δ 173.62, 172.43, 149.06, 137.72, 127.82, 121.13, 120.32, 111.54, 66.23, 66.01, 52.36, 49.82, 44.00, 41.45, 40.23, 35.64, 33.47, 32.64, 31.66, 28.30, 20.99.
HRMS (ESI) *m/z* calculated for $C_{21}H_{25}N_2O_3$ $[M+H]^+$: 353.1860, found: 353.1861.
$[\alpha]_D^{20}$ –11 (c = 1.5, $CHCl_3$).

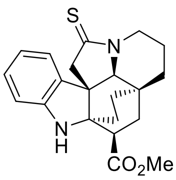

(+)-10-Thioxokopsinine (S9)[6]
(–)-Kopsinilam (**31**) (17 mg, 48 µmol, 1 eq.) was dissolved in toluene (10 mL) with gentle heating. Lawesson's reagent (59 mg, 0.15 mmol, 3 eq.) was added and the mixture was heated to 90 °C for 30 minutes, after which it was slowly allowed to room temperature. All volatiles were removed *in vacuo* and the product was obtained after flash column chromatography (silica gel, 25–40% EtOAc in cyclohexane) as a white solid (15 mg, 41 µmol, 85%).
R_f (50% EtOAc in cyclohexane): 0.48 (visualized by UV and $Ce(SO_4)_2$ stain).
^{1}H NMR (500 MHz, $CDCl_3$) δ 7.04 (ddd, *J* = 7.8, 7.7, 1.3 Hz, 1H), 6.99 (dd, *J* = 7.4, 1.3 Hz, 1H), 6.76 (ddd, *J* = 7.7, 7.4, 0.9 Hz, 1H), 6.69 (dd, *J* = 7.8, 0.9 Hz, 1H), 5.04 (ddd, *J* = 12.7, 4.8, 1.8 Hz, 1H), 4.00 (dd, *J* = 19.8, 1.3 Hz, 1H), 3.91 (br. s, 1H), 3.87 – 3.80 (m, 1H), 3.77 (s, 2H), 3.05 (ddd, *J* = 12.7, 3.6, 1.9 Hz, 1H), 3.00 – 2.93 (m, 1H), 2.69 (dd, *J* = 19.8, 1.9 Hz, 1H), 2.24 (ddd, *J* = 13.7, 10.2, 3.1 Hz, 1H), 1.96 (dddd, *J* = 12.5, 10.9, 7.9, 1.3 Hz, 1H), 1.85 – 1.69 (m, 3H), 1.69 – 1.57 (m, 1H), 1.52 (td, *J* = 13.7, 4.1 Hz, 1H), 1.45 – 1.26 (m, 3H).
^{13}C NMR (126 MHz, $CDCl_3$) 198.41, 173.40, 148.93, 136.84, 128.04, 121.12, 120.37, 111.66, 73.76, 65.90, 54.94, 52.46, 51.35, 45.99, 43.94, 35.53, 35.10, 32.80, 31.52, 28.44, 20.81.
HRMS (ESI) *m/z* calculated for $C_{21}H_{25}N_2O_2S$ $[M+H]^+$: 369.1631, found: 369.1630.
$[\alpha]_D^{20}$ +95 (c = 1.0, $CHCl_3$).

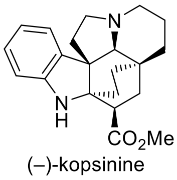

(–)-kopsinine

(–)-Kopsinine (**3**)[6]
Thiolactam **S9** (5 mg, 14 µmol) was dissolved in absolute ethanol (4 mL) and a large excess of Raney nickel was added. The mixture was heated to reflux and stirred vigorously for 1.5 h. The reaction was allowed to room temperature, filtered over celite and concentrated *in vacuo*. The product was obtained after purification by flash column chromatography (alumina, 0–5% MeOH in DCM) as a white solid (2.3 mg, 6.8 µmol, 50%).
R_f (1/8/8 Et_3N/EtOAc/cyclohexane): 0.58 (visualized by UV and $Ce(SO_4)_2$ stain).
^{1}H NMR (600 MHz, $CDCl_3$) δ 7.18 (d, *J* = 7.4 Hz, 1H), 6.99 (t, *J* = 7.6 Hz, 1H), 6.75 (t, *J* = 7.4 Hz, 1H), 6.66 (d, *J* = 7.6 Hz, 1H), 3.76 (s, 3H), 3.74 (br. s, 1H), 3.35 (q, *J* = 8.2 Hz, 1H), 3.12 (dd, *J* = 13.4, 3.8 Hz, 1H), 3.03 – 2.93 (m, 3H), 2.89 (t, *J* = 9.7 Hz, 1H), 2.78 (ddd, *J* = 13.1, 9.3, 3.2 Hz, 1H), 2.64 (ddd, *J* = 14.1, 8.2, 3.4 Hz, 1H), 1.98 – 1.86 (m, 2H), 1.65 – 1.49 (m, 2H), 1.48 – 1.18 (m, 6H).
^{13}C NMR (151 MHz, $CDCl_3$) δ 175.03, 149.25, 140.86, 126.82, 121.83, 119.96, 111.02, 77.37, 77.16, 76.95, 68.52, 66.87, 58.14, 52.16, 50.89, 47.77, 44.02, 36.68, 34.92, 34.11, 34.08, 32.27, 31.99, 17.27.
HRMS (ESI) *m/z* calculated for $C_{21}H_{27}N_2O_2$ $[M+H]^+$: 339.2067, found: 399.2077.
$[\alpha]_D^{20}$ –47 (c = 0.4, $CHCl_3$).

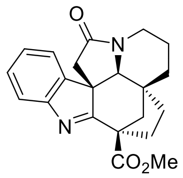

(–)-6,7-Dihydro-10-oxokopsifoline D (S10)

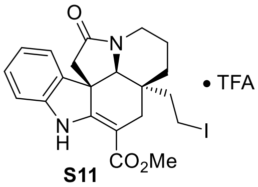

S11

Boc-protected iodide **30** (46 mg, 80 µmol, 1 eq.) was dissolved in DCM (1 mL) and cooled to 0 °C. TFA (1 mL) was added, the mixture was allowed to room temperature and stirred for 1 h. Evaporation of all volatiles *in vacuo*, co-evaporating all TFA twice with toluene, yielded **S11**. This salt was immediately dissolved in DCM (2 mL) and DIPEA (69 µL, 0.4 mmol, 5 eq.) was added. The reaction was stirred at room temperature for 18 h, after which all volatiles were removed *in vacuo.* Purification by flash column chromatography (silica gel, 1% Et_3N and 50% EtOAc in cyclohexane) yielded the indolenine product as a slightly yellow oil (16 mg, 47 µmol, 59%).

R_f (50% ETOAc in cyclohexane): 0.25 (visualized by UV and $Ce(SO_4)_2$ stain).

^{1}H NMR (500 MHz, $CDCl_3$) δ 7.58 (dt, *J* = 7.7, 0.9 Hz, 1H), 7.38 – 7.29 (m, 2H), 7.23 (td, *J* = 7.5, 1.0 Hz, 1H), 4.35 (ddt, *J* = 12.9, 4.7, 1.8 Hz, 1H), 3.77 (s, 3H), 3.44 (d, *J* = 2.0 Hz, 1H), 3.24 (d, *J* = 18.4 Hz, 1H), 2.80 (tdd, *J* = 12.9, 3.4, 1.3 Hz, 1H), 2.54 (dd, *J* = 18.4, 1.4 Hz, 1H), 2.45 (dd, *J* = 12.6, 2.5 Hz, 1H), 2.33 – 2.18 (m, 2H), 1.90 – 1.82 (m, 1H), 1.82 – 1.71 (m, 1H), 1.69 – 1.61 (m, 1H), 1.61 – 1.45 (m, 3H), 1.07 (ddd, *J* = 14.4, 9.8, 8.2 Hz, 1H).

^{13}C NMR (126 MHz, $CDCl_3$) δ 185.82, 171.48, 171.24, 154.55, 145.02, 128.65, 126.96, 121.17, 120.76, 67.53, 56.89, 55.19, 52.86, 44.52, 40.88, 39.23, 39.06, 38.60, 36.04, 33.61, 22.72.

HRMS (ESI) *m/z* calculated for $C_{21}H_{23}N_2O_3$ $[M+H]^+$: 351.1703, found: 351.1712.

$[\alpha]_D^{20}$ –34 (c = 1.6, $CHCl_3$).

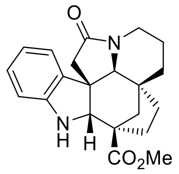

(–)-1,2,6,7-Tetrahydro-10-oxokopsifoline D (32)

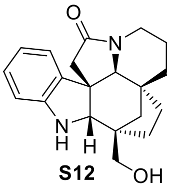

S12

Indolenine **S10** (13.9 mg, 39.7 µmol, 1 eq.) was dissolved in methanol and sodium borohydride (15 mg, 0.40 mmol, 10 eq.) was added in one portion. The mixture was stirred at room temperature for 2 h, after which another portion of sodium borohydride (15 mg, 0.40 mmol, 10 eq.) was added. After two more hours at room temperature, all volatiles were removed *in vacuo* and water was added. The mixture was extracted three timed with DCM and the combined organic layers were washed with brine and dried over sodium sulphate. Purification by flash column chromatography (silica gel, 50–100% EtOAc in cyclohexane) yielded the product as a white solid (3.8 mg, 11 µmol, 27%) as well as the primary alcohol **S12** as a result of ester reduction (6.0 mg, 18 µmol, 47%).

R_f (50% EtOAc in cyclohexane): 0.23 (visualized by UV and $Ce(SO_4)_2$ stain).

^{1}H NMR (500 MHz, $CDCl_3$) δ 7.07 (td, *J* = 7.8, 1.2 Hz, 1H), 7.03 (dd, *J* = 7.8, 1.0 Hz, 1H), 6.71 (td, *J* = 7.8, 1.0 Hz, 1H), 6.58 (d, *J* = 7.8 Hz, 1H), 4.61 (br. s, 1H), 4.24 (ddd, *J* = 13.3, 5.6, 1.7 Hz, 1H), 3.75 (d, *J* = 1.5 Hz, 1H), 3.73 (s, 2H), 3.62 (d, *J* = 1.6 Hz, 1H), 2.80 (d, *J* = 17.7 Hz, 1H), 2.75 – 2.67 (m, 1H), 2.35 (dd, *J* = 17.7, 1.7 Hz, 1H), 1.95 (dt, *J* = 12.1, 2.0 Hz, 1H), 1.78 – 1.66 (m, 1H), 1.66 – 1.50 (m, 4H), 1.48 – 1.42 (m, 1H), 1.35 – 1.22 (m, 3H).

^{13}C NMR (126 MHz, $CDCl_3$) δ 176.40, 171.80, 150.30, 132.64, 128.93, 122.05, 118.56, 107.82, 71.38, 69.08, 54.80, 52.41, 49.25, 47.02, 43.71, 40.25, 37.19, 35.30, 34.39, 26.47, 21.53.

HRMS (ESI) *m/z* calculated for $C_{21}H_{25}N_2O_3$ $[M+H]^+$: 353.1860, found: 353.1868.

$[\alpha]_D^{20}$ –32 (c = 0.4, $CHCl_3$).

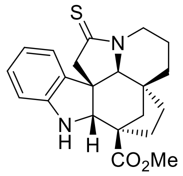

(–)-1,2,6,7-Tetrahydro-10-thioxokopsifoline D (S13)[6]

Lactam **32** (4 mg, 11 µmol, 1 eq.) was dissolved in toluene (2.2 mL) with gentle heating. Lawesson's reagent (14 mg, 34 µmol, 3 eq.) was added and the mixture was heated to 90 °C for 30 minutes, after which it was slowly allowed to room temperature. All volatiles were removed *in vacuo* and the product was obtained after flash column chromatography (silica gel, 25–40% EtOAc in cyclohexane) as a white solid (2.0 mg, 5.4 µmol, 48%).

R_f (50% EtOAc in cyclohexane): 0.80 (visualized by UV and $Ce(SO_4)_2$ stain).

^{1}H NMR (500 MHz, $CDCl_3$) δ 7.07 (td, *J* = 7.5, 1.0 Hz, 1H), 7.01 (dd, *J* = 7.5, 1.3 Hz, 1H), 6.71 (td, *J* = 7.5, 1.0 Hz, 1H), 6.59 (d, *J* = 7.5 Hz, 1H), 5.01 – 4.95 (m, 1H), 4.60 (br. s, 1H), 3.87 (s, 1H), 3.76 (d, *J* = 1.5 Hz, 1H), 3.73 (s, 3H), 3.43 (d, *J* = 18.4 Hz, 1H), 2.93 – 2.81 (m, 2H), 1.86 – 1.80 (m, 2H), 1.80 – 1.59 (m, 5H), 1.59 – 1.52 (m, 1H), 1.52 – 1.47 (m, 1H), 1.31 – 1.28 (m, 1H).

^{13}C NMR (126 MHz, $CDCl_3$) δ 198.06, 150.33, 131.34, 129.13, 121.87, 118.62, 107.94, 75.72, 70.76, 61.31, 54.55, 52.47, 48.58, 45.30, 44.08, 37.37, 34.58, 34.43, 29.85, 26.54, 21.42.

HRMS (ESI) *m/z* calculated for $C_{21}H_{25}N_2O_2S$ $[M+H]^+$: 369.1631, found: 369.1637.

$[\alpha]_D^{20}$ –53 (c = 0.15, $CHCl_3$).

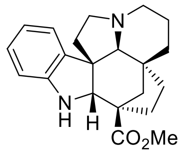

(–)-1,2,6,7-tetrahydrokopsifoline D (33)[6]

Thiolactam **S13** (2 mg, 5.4 µmol) was dissolved in absolute ethanol (2 mL) and a large excess of Raney nickel was added. The mixture was heated to reflux and stirred vigorously for 1.5 h. The reaction was allowed to room temperature, filtered over celite and concentrated *in vacuo*. The product was obtained after purification by flash column chromatography (silica gel, 50% EtOAc in cyclohexane) as a white solid (1.4 mg, 4.1 µmol, 76%).

R_f (1/8/8 Et_3N/EtOAc/cyclohexane): 0.72 (visualized by UV and $Ce(SO_4)_2$ stain).

^{1}H NMR (600 MHz, $CDCl_3$) δ 7.07 (d, *J* = 7.2 Hz, 1H), 7.02 (td, *J* = 7.2, 1.3 Hz, 1H), 6.66 (t, *J* = 7.2 Hz, 1H), 6.56 (d, *J* = 7.2 Hz, 1H), 4.51 (s, 1H), 3.80 (d, *J* = 1.5 Hz, 1H), 3.71 (s, 3H), 3.11 (t, *J* = 8.3 Hz, 2H), 2.36 (dt, *J* = 11.5, 2.0 Hz, 1H), 2.31 (ddd, *J* = 11.1, 8.7, 7.4 Hz, 1H), 2.19 (ddd, *J* = 12.9, 11.1, 7.8 Hz, 1H), 2.14 (s, 1H), 2.01 (td, *J* = 11.5, 3.1 Hz, 1H), 1.75 (dd, *J* = 12.8, 7.3 Hz, 1H), 1.71 – 1.57 (m, 3H), 1.54 – 1.48 (m, 2H), 1.46 – 1.38 (m, 2H), 1.35 – 1.29 (m, 1H), 1.22 – 1.16 (m, 1H).
^{13}C NMR (151 MHz, $CDCl_3$) δ 177.29, 150.09, 136.48, 127.93, 121.99, 118.00, 107.62, 76.73, 72.96, 57.11, 54.69, 53.48, 52.13, 45.43, 43.53, 38.57, 35.43, 35.35, 26.49, 23.87.
HRMS (ESI) *m/z* calculated for $C_{21}H_{27}N_2O_2$ $[M+H]^+$: 339.2067, found: 339.2060.
$[\alpha]_D^{20}$ –60 (c = 0.10, $CHCl_3$).

NMR Spectra

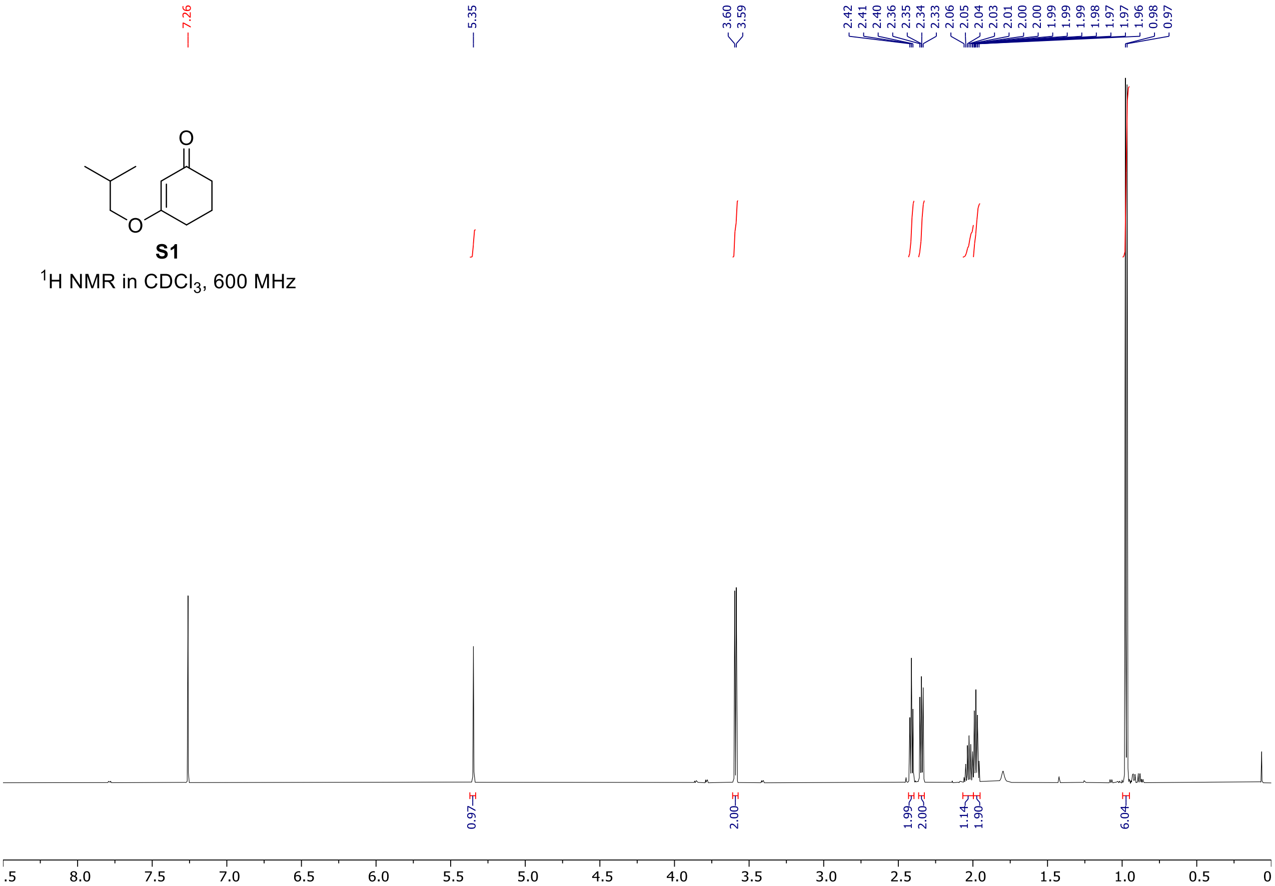
O
O
S1
^{1}H NMR in $CDCl_3$, 600 MHz
7.26
5.35
3.60
3.59
2.42
2.41
2.40
2.36
2.35
2.34
2.33
2.06
2.05
2.04
2.03
2.01
2.00
2.00
1.99
1.99
1.99
1.98
1.97
1.97
1.96
0.98
0.97
0.97
2.00
1.99
2.00
1.14
1.90
6.04
.5
8.0
7.5
7.0
6.5
6.0
5.5
5.0
4.5
4.0
3.5
3.0
2.5
2.0
1.5
1.0
0.5
0

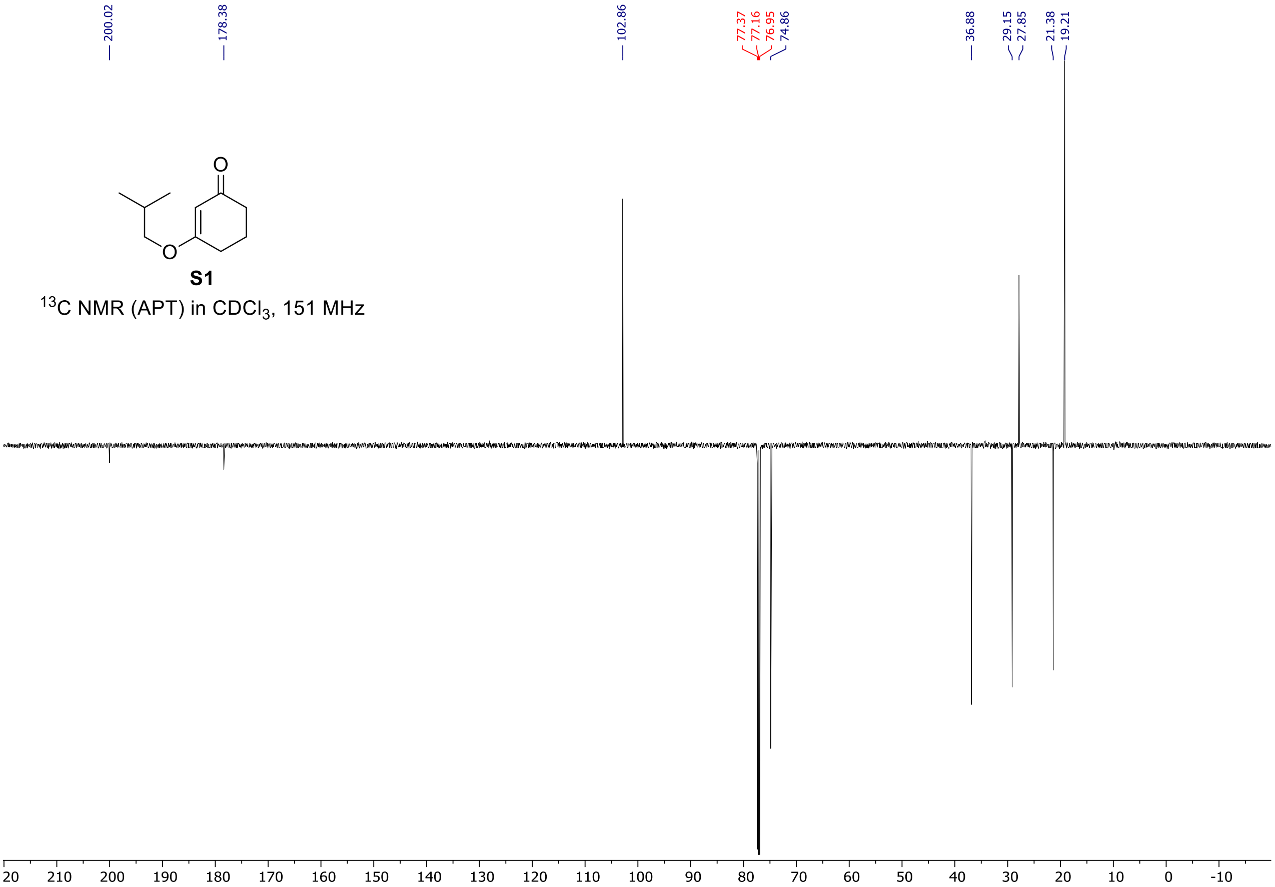
O
O
S1
^{13}C NMR (APT) in $CDCl_3$, 151 MHz
200.02
178.38
102.86
77.37
77.16
76.95
74.86
36.88
29.15
27.85
21.38
19.21
20
210
200
190
180
170
160
150
140
130
120
110
100
90
80
70
60
50
40
30
20
10
0
-10

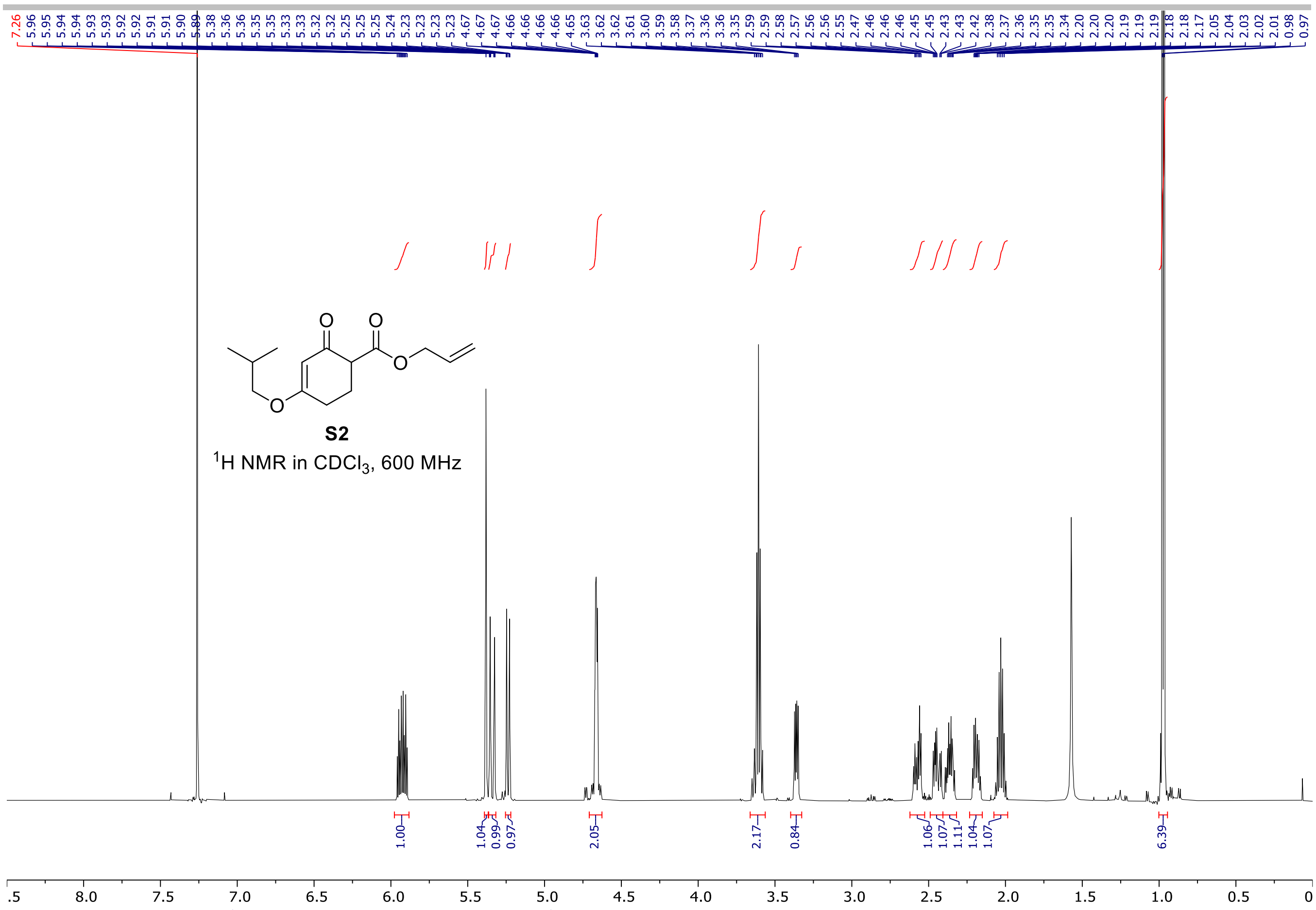
S2
^{1}H NMR in $CDCl_3$, 600 MHz

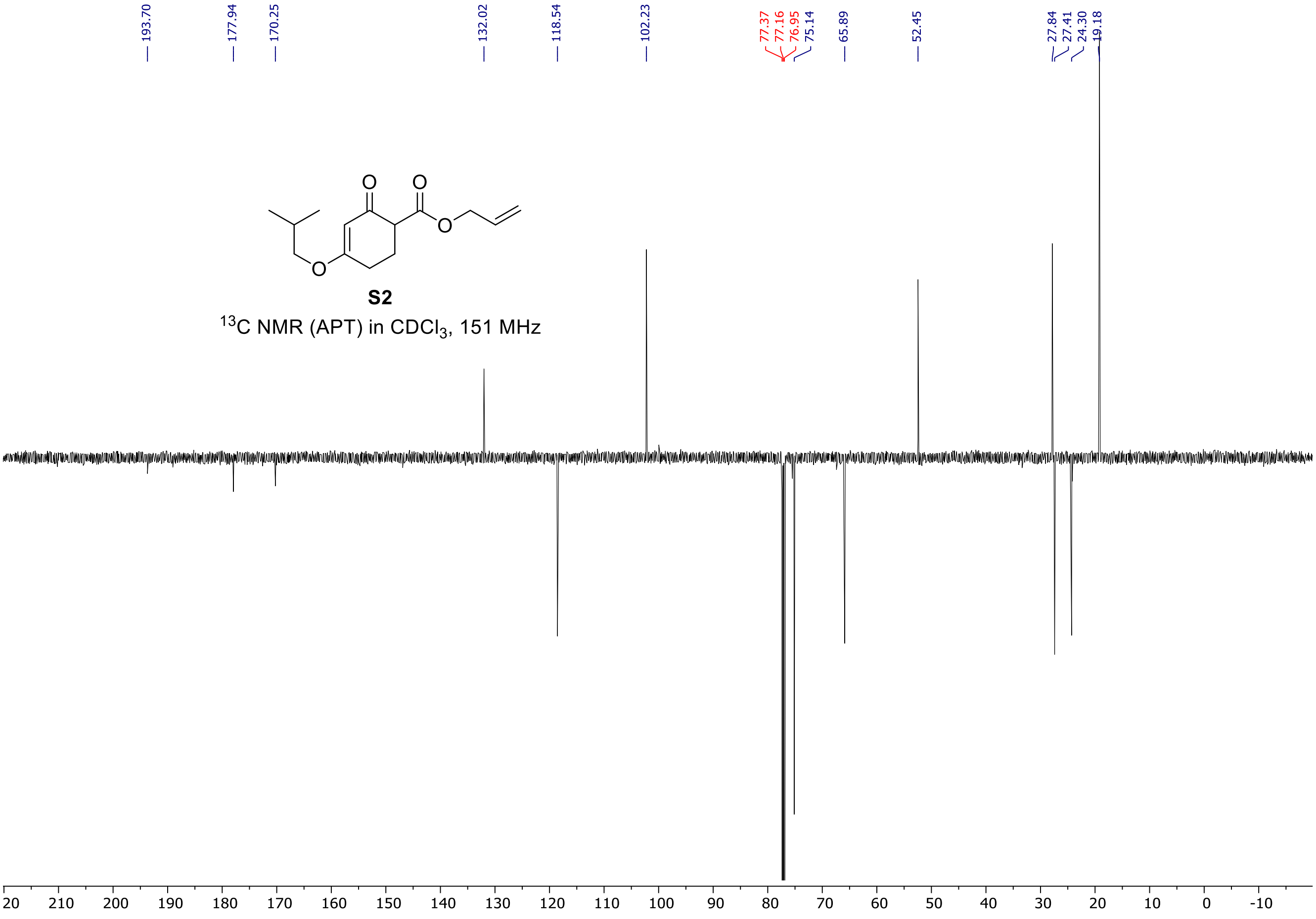
S2
^{13}C NMR (APT) in $CDCl_3$, 151 MHz
193.70
177.94
170.25
132.02
118.54
102.23
77.37
77.16
76.95
75.14
65.89
52.45
27.84
27.41
24.30
19.18

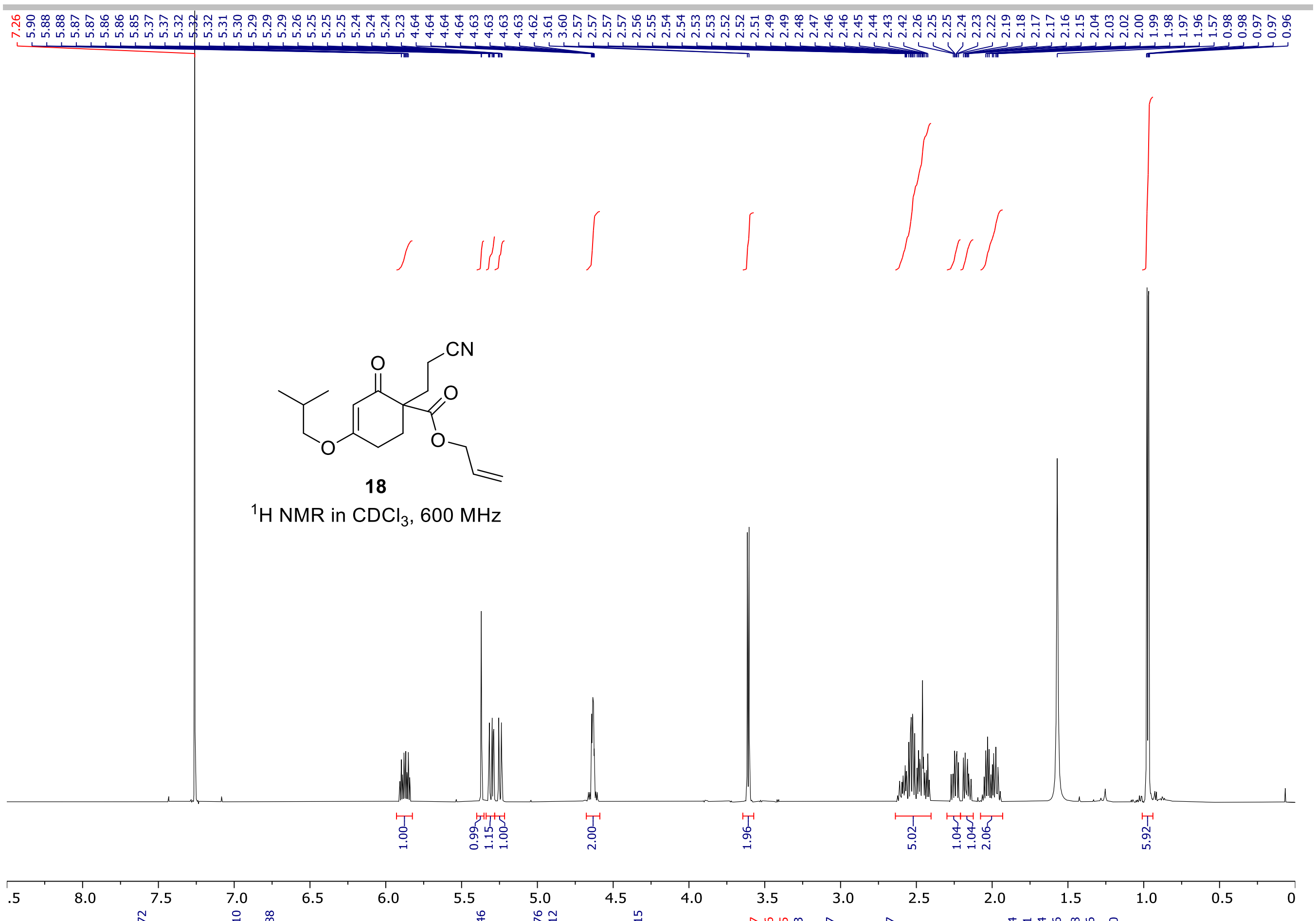

18

^{1}H NMR in $CDCl_3$, 600 MHz

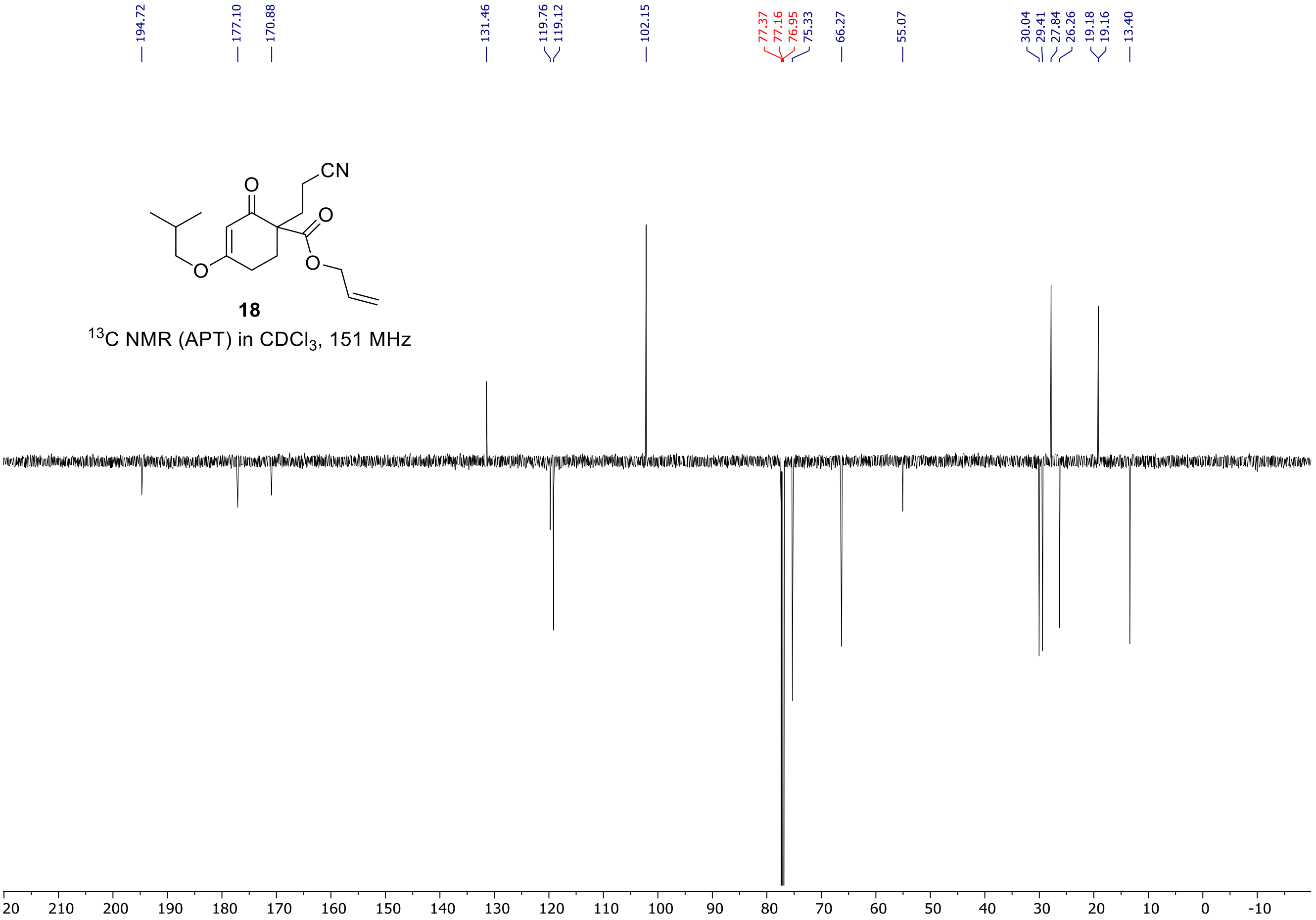

18

^{13}C NMR (APT) in $CDCl_3$, 151 MHz

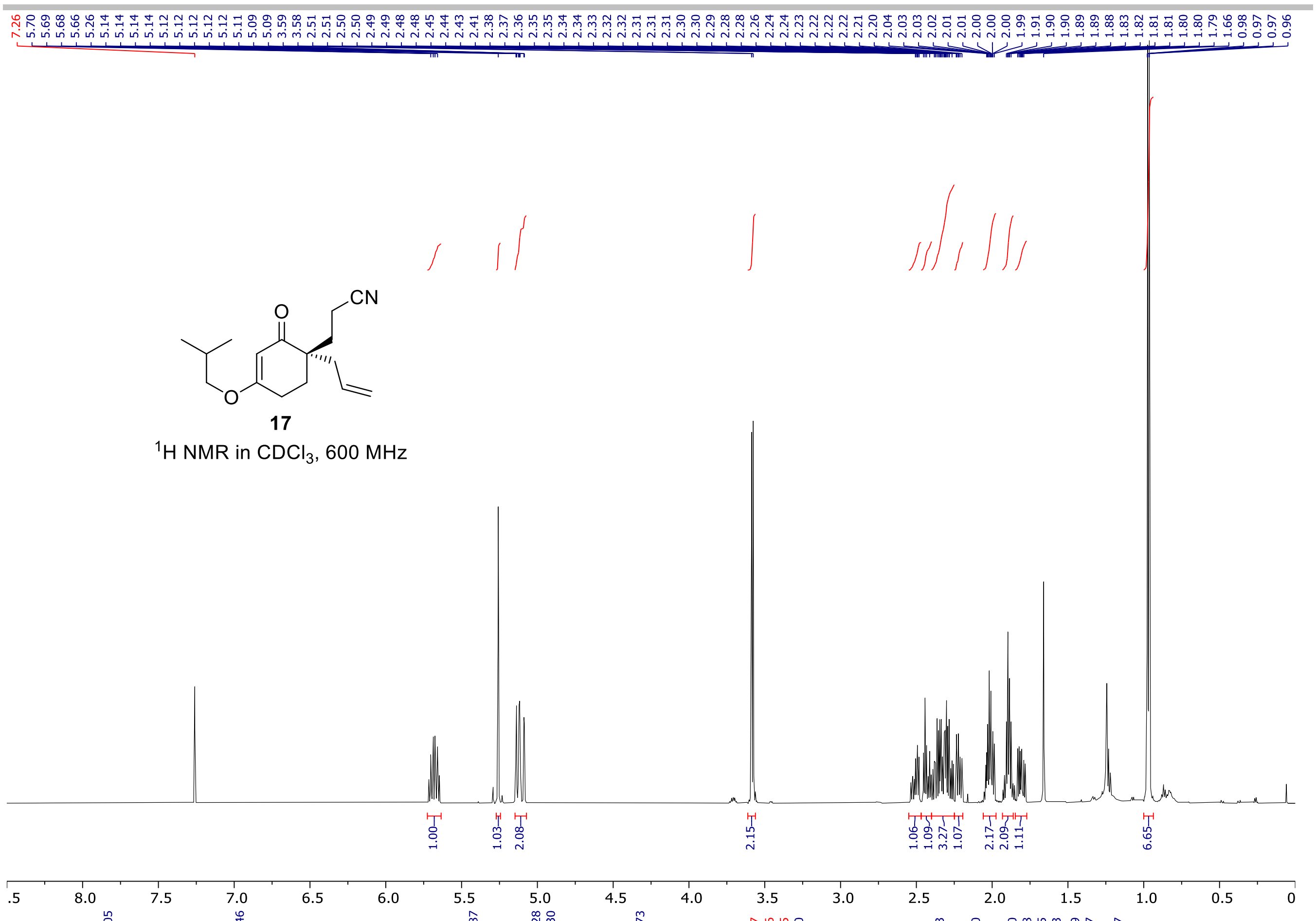
17
1H NMR in CDCl3, 600 MHz

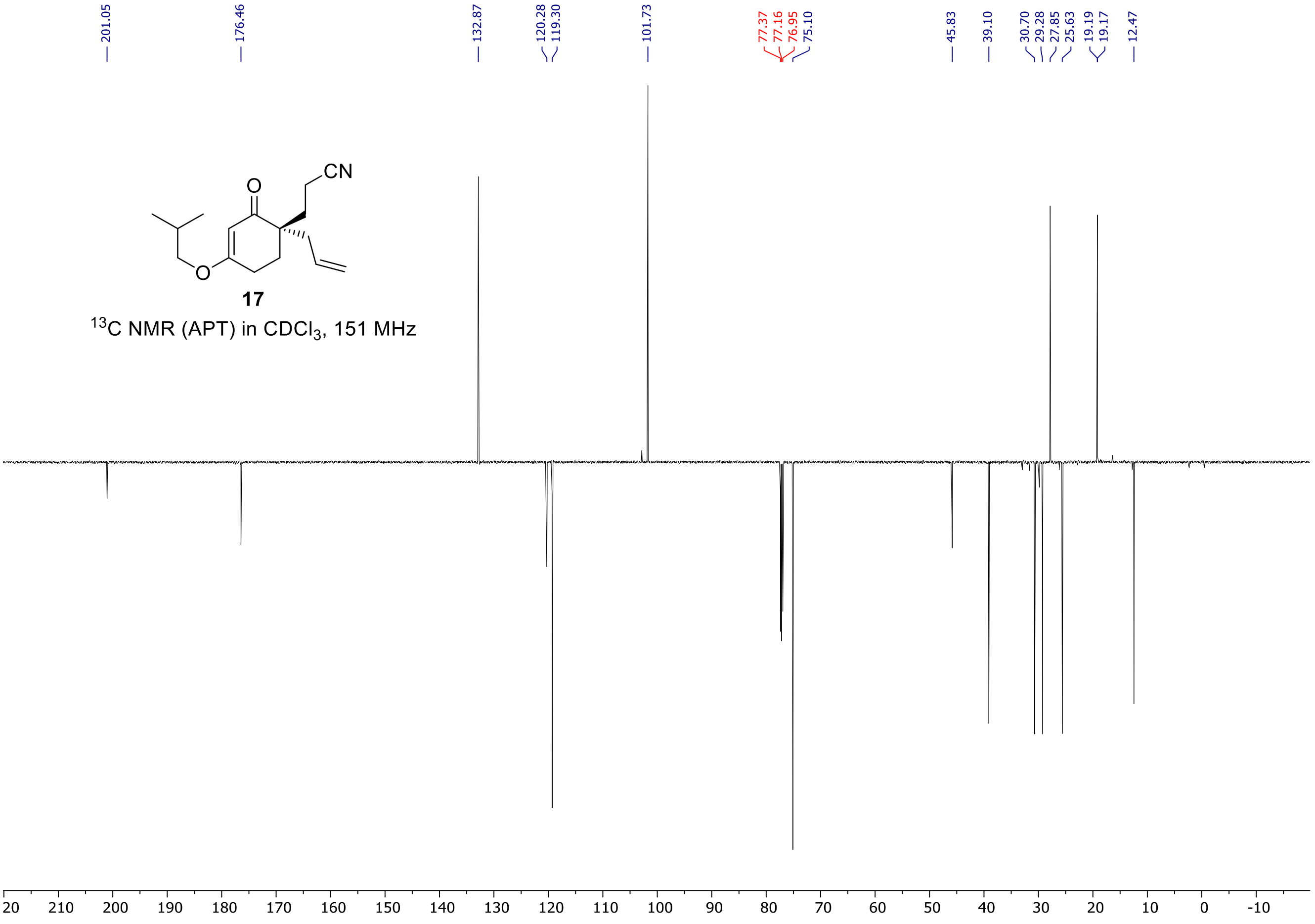
17
13C NMR (APT) in CDCl3, 151 MHz

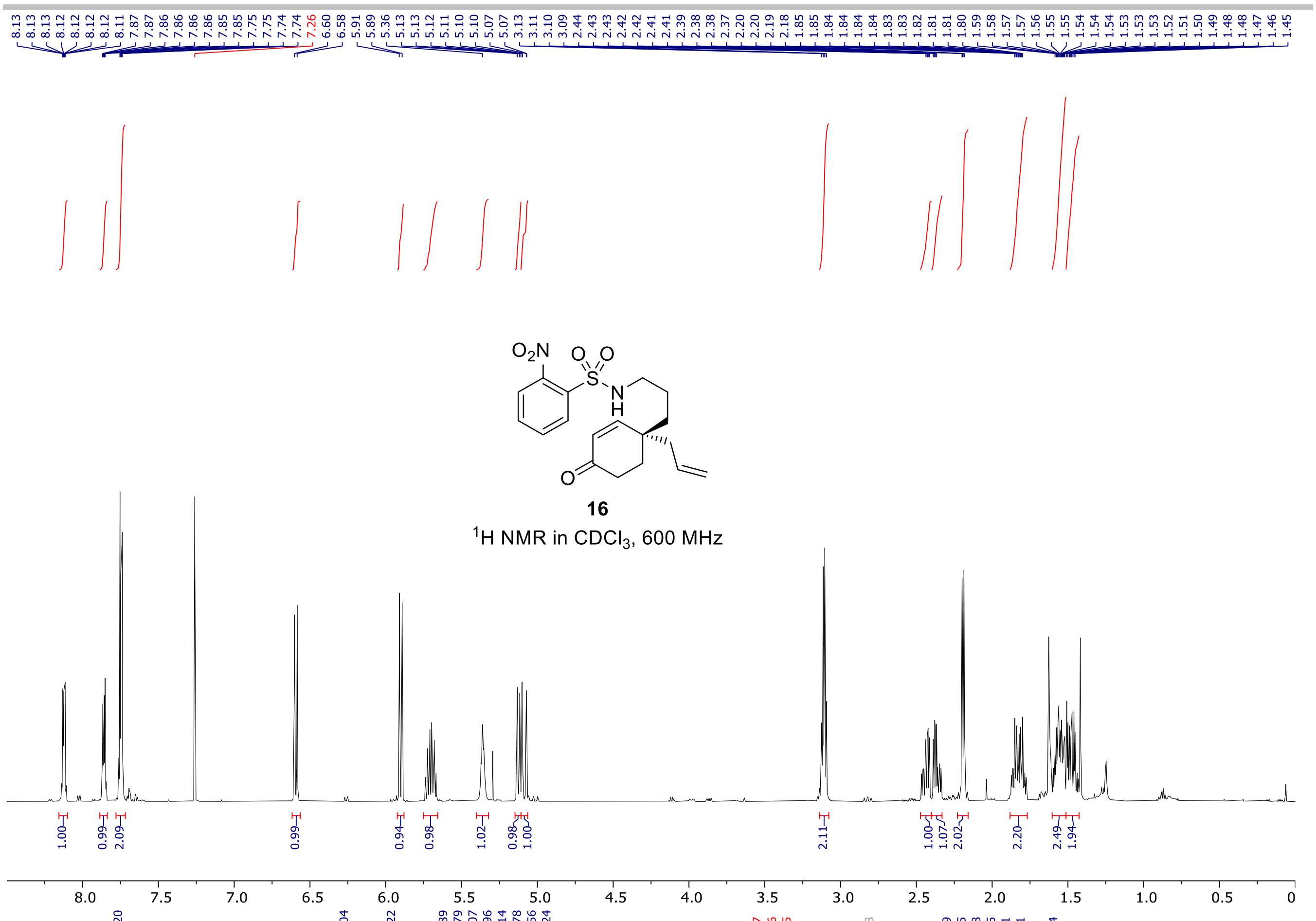
16
^{1}H NMR in CDCl$_3$, 600 MHz

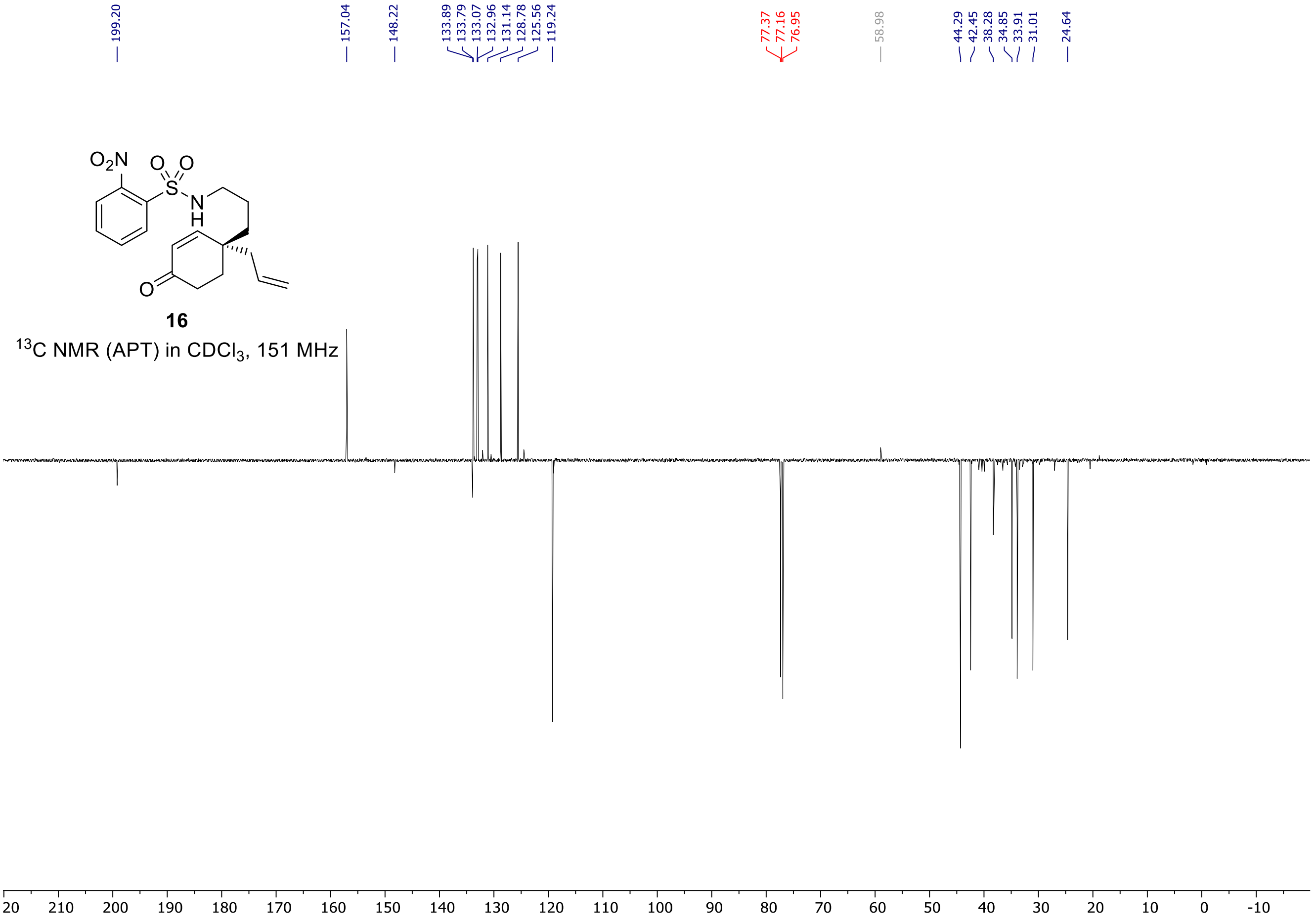
16
^{13}C NMR (APT) in CDCl$_3$, 151 MHz

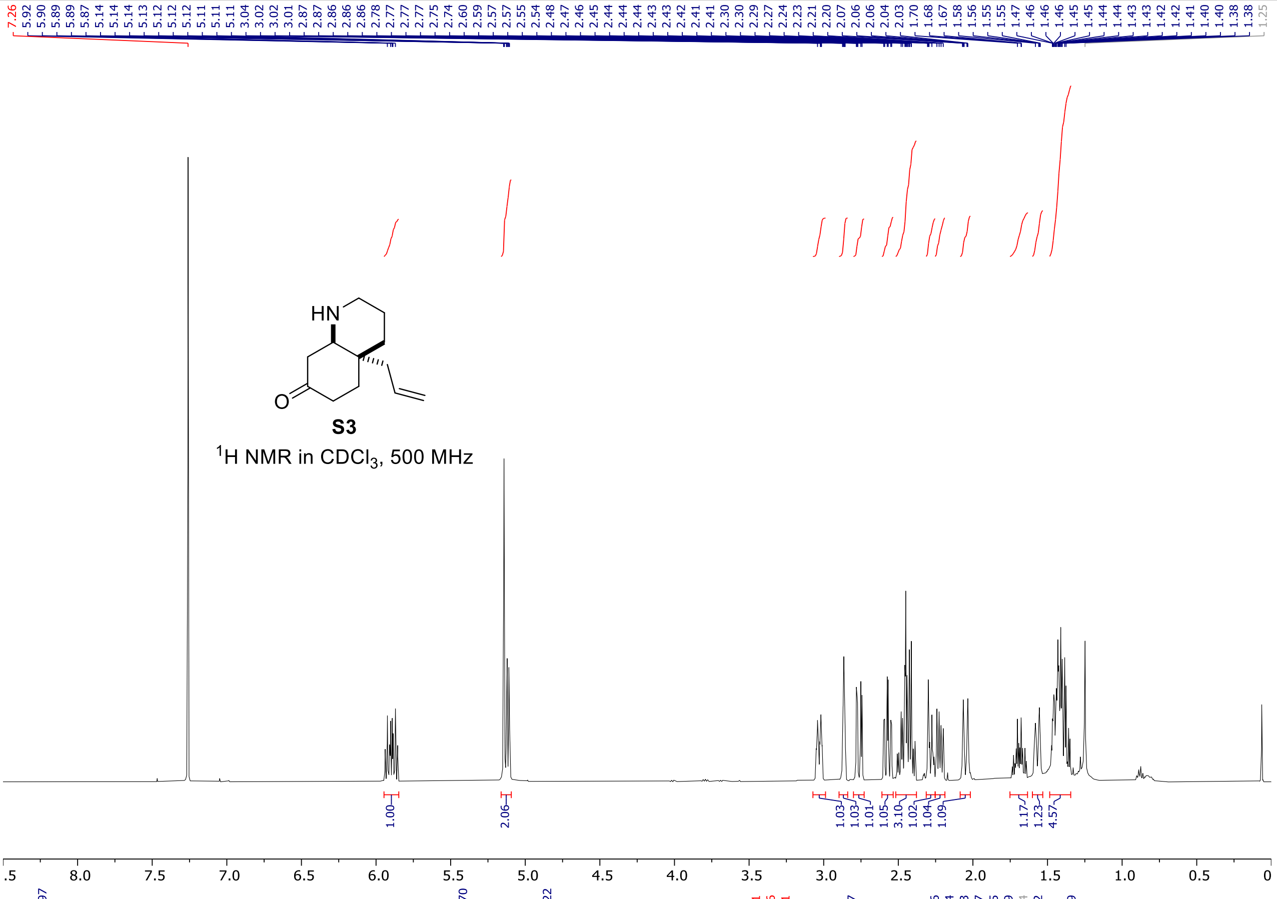
HN
O
S3
^{1}H NMR in $CDCl_3$, 500 MHz

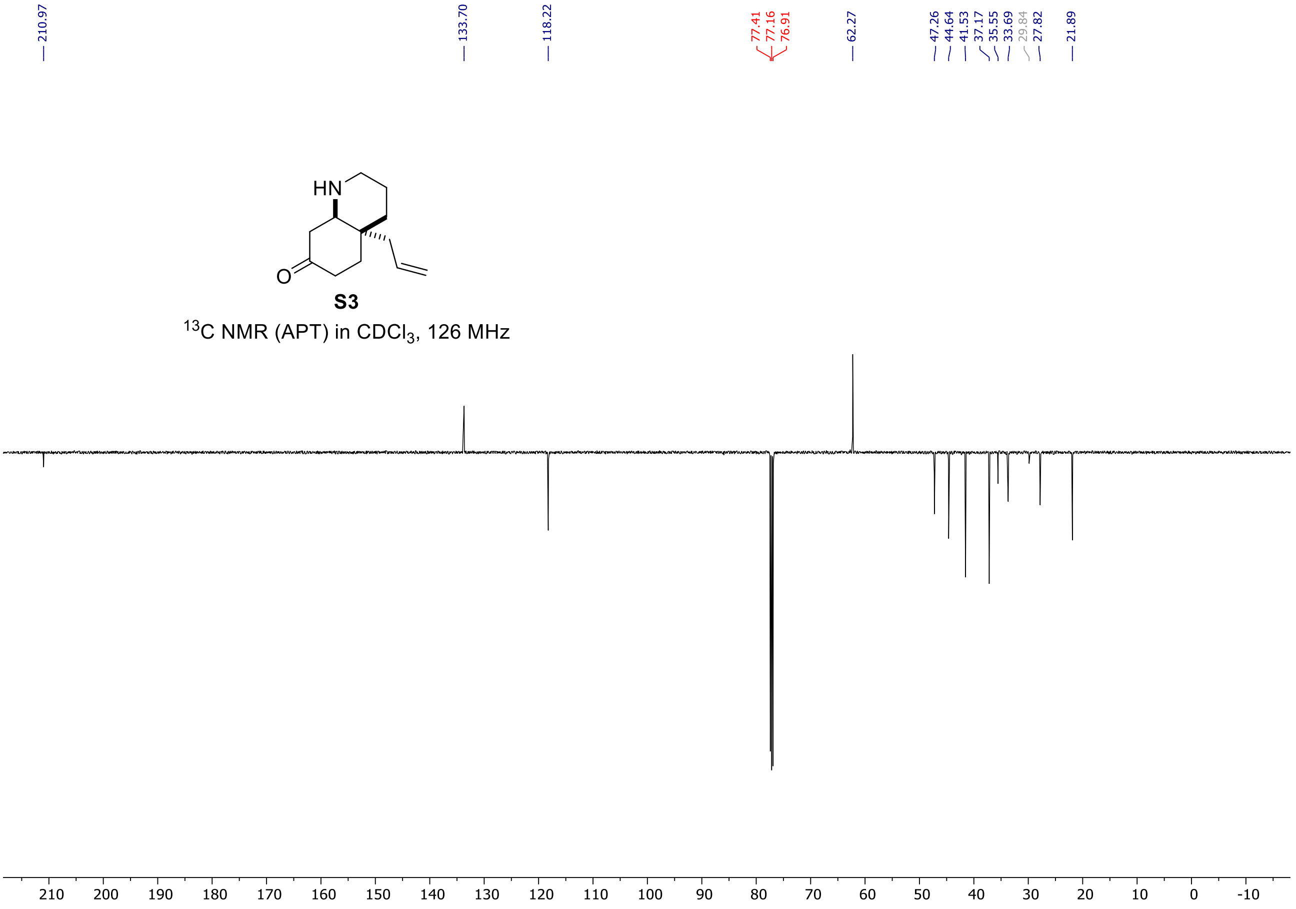
HN
O
S3
^{13}C NMR (APT) in $CDCl_3$, 126 MHz

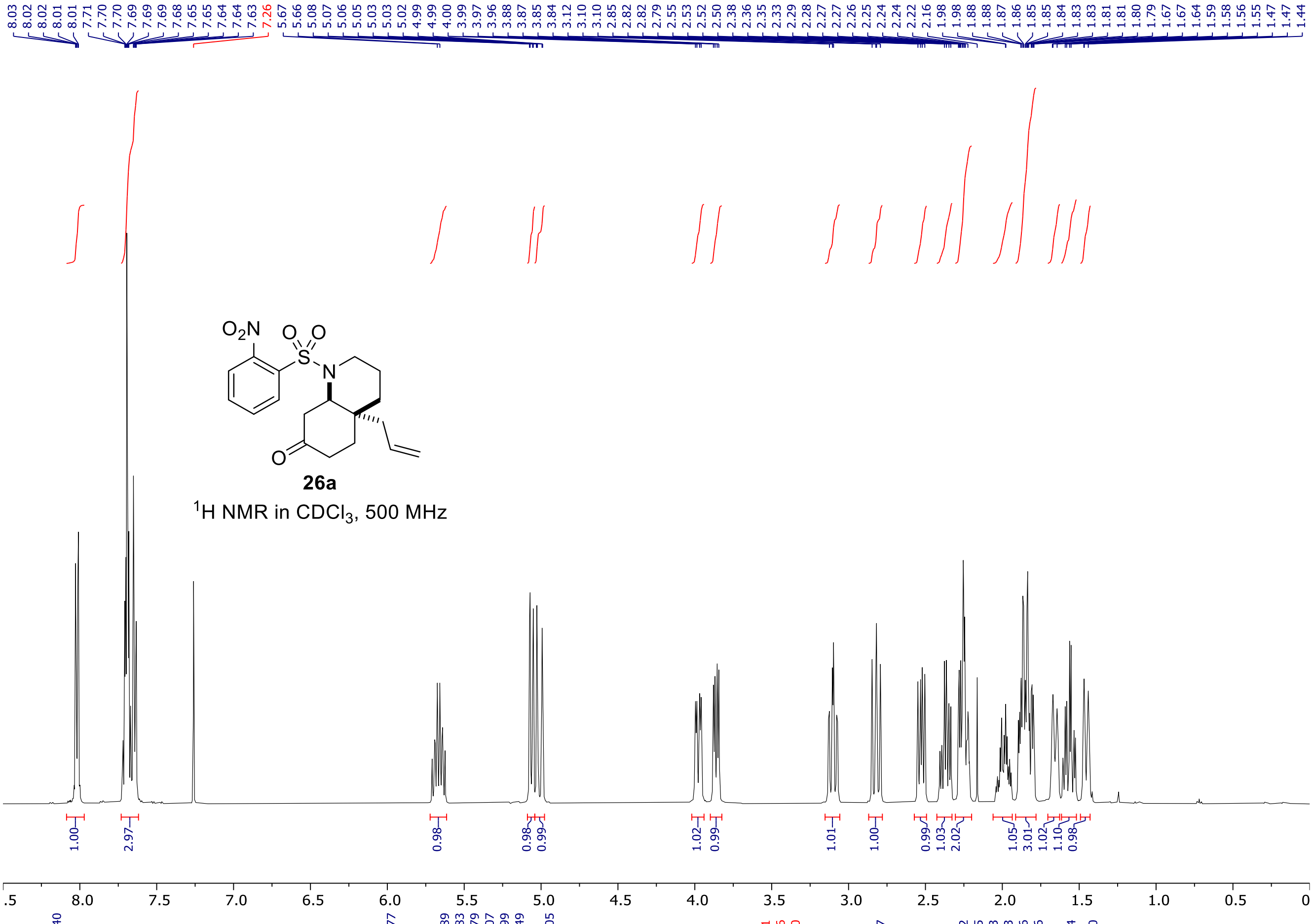
26a
^{1}H NMR in $CDCl_3$, 500 MHz

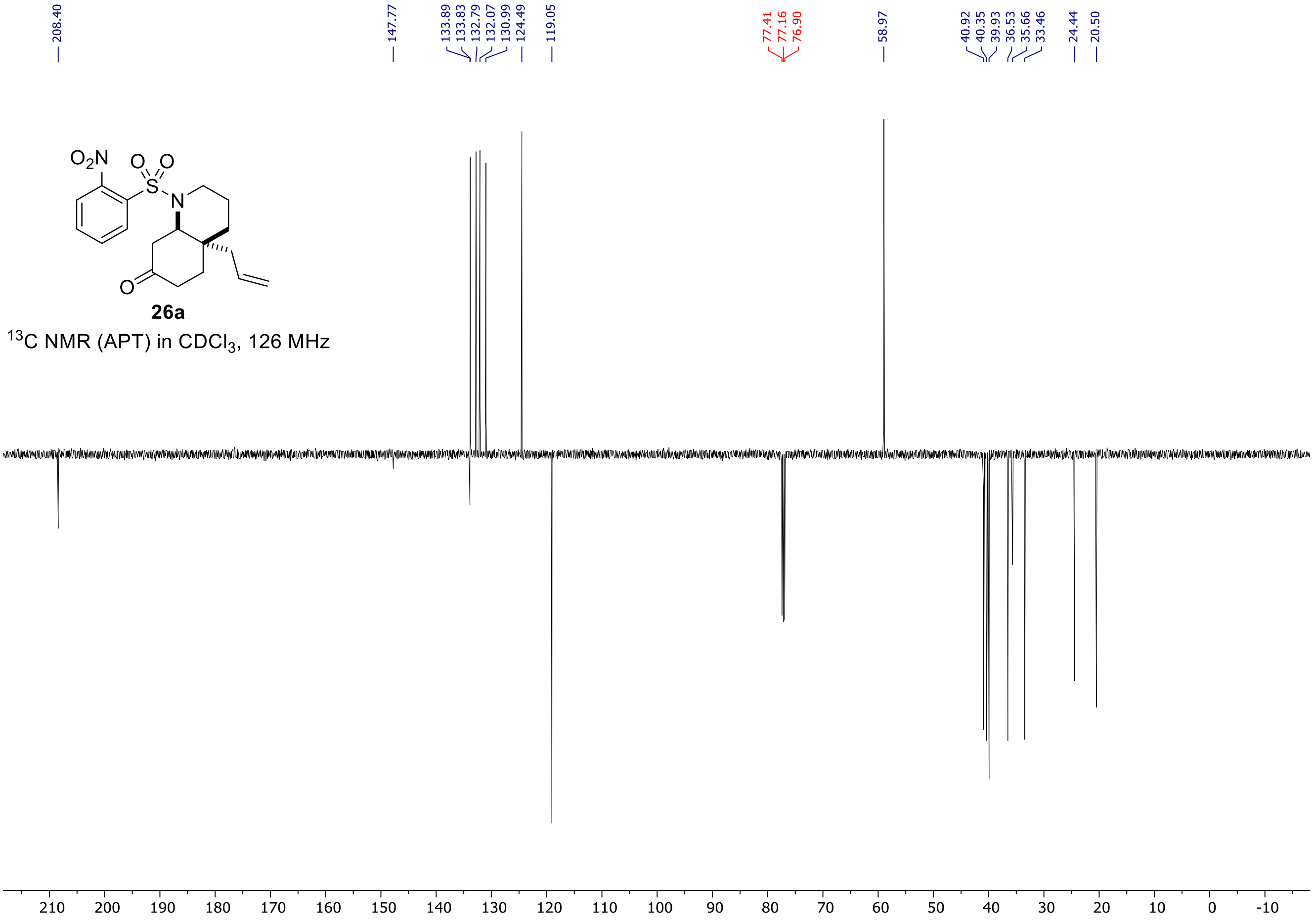
26a
^{13}C NMR (APT) in $CDCl_3$, 126 MHz

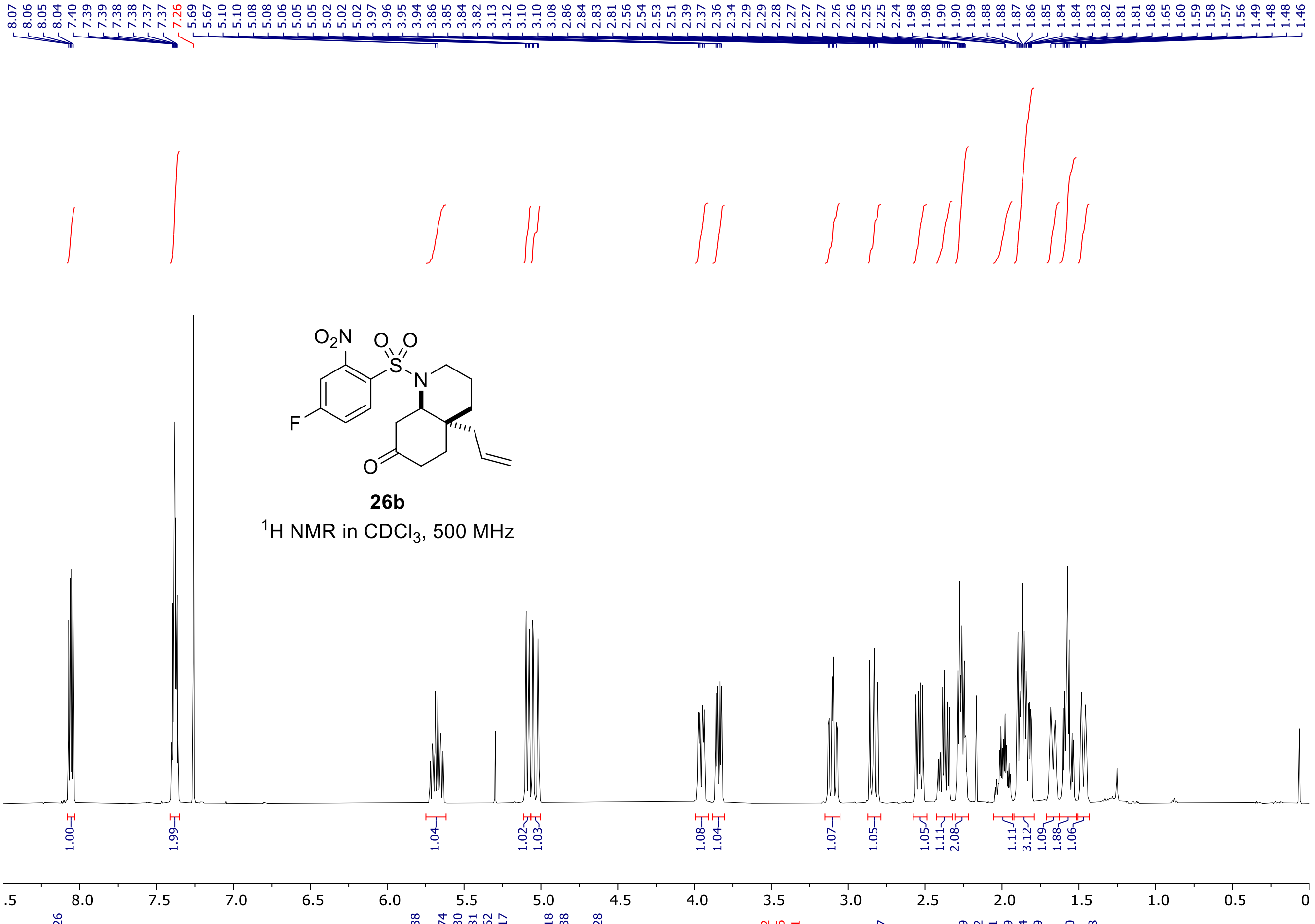
26b
^{1}H NMR in $CDCl_3$, 500 MHz

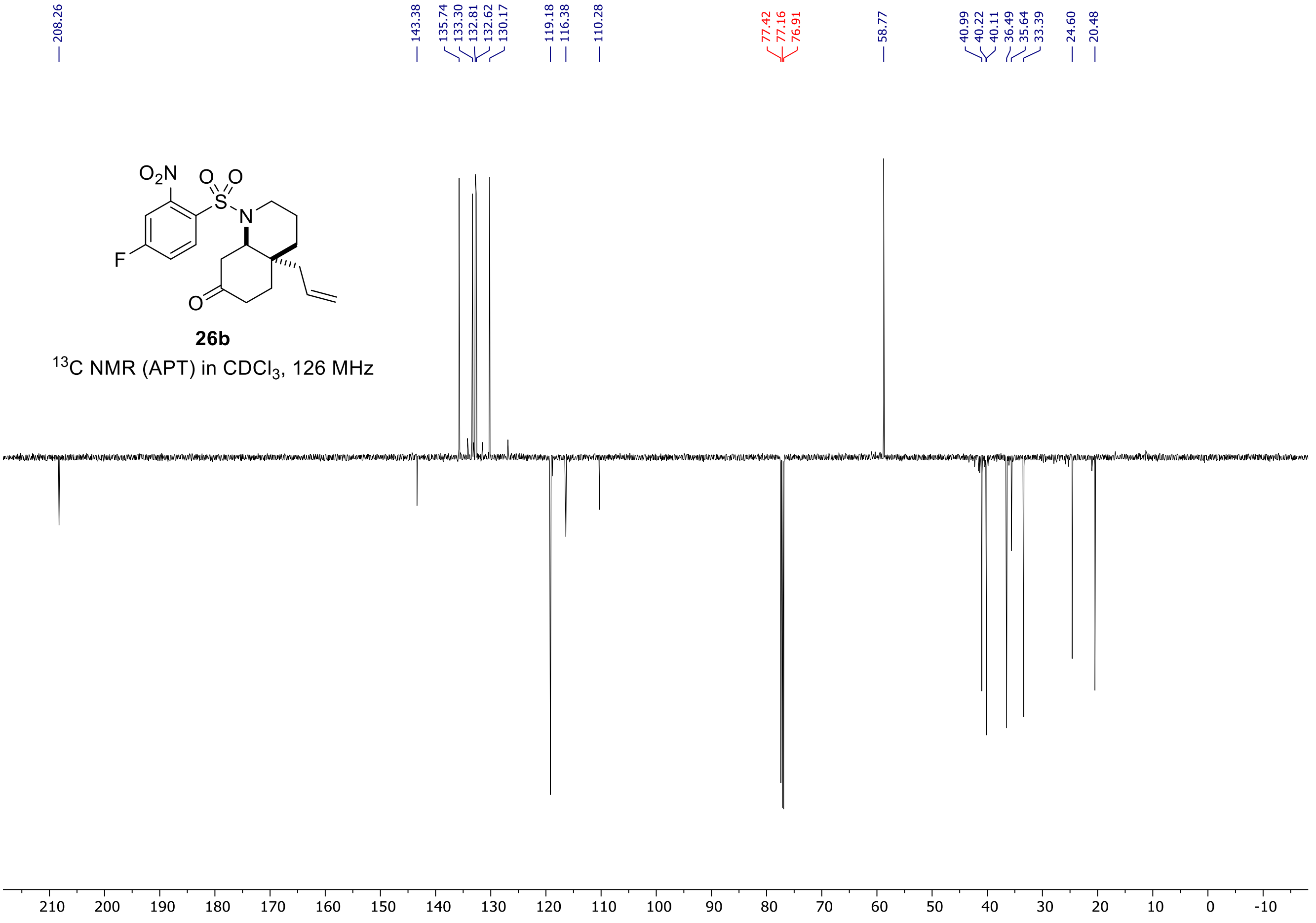
26b
^{13}C NMR (APT) in $CDCl_3$, 126 MHz

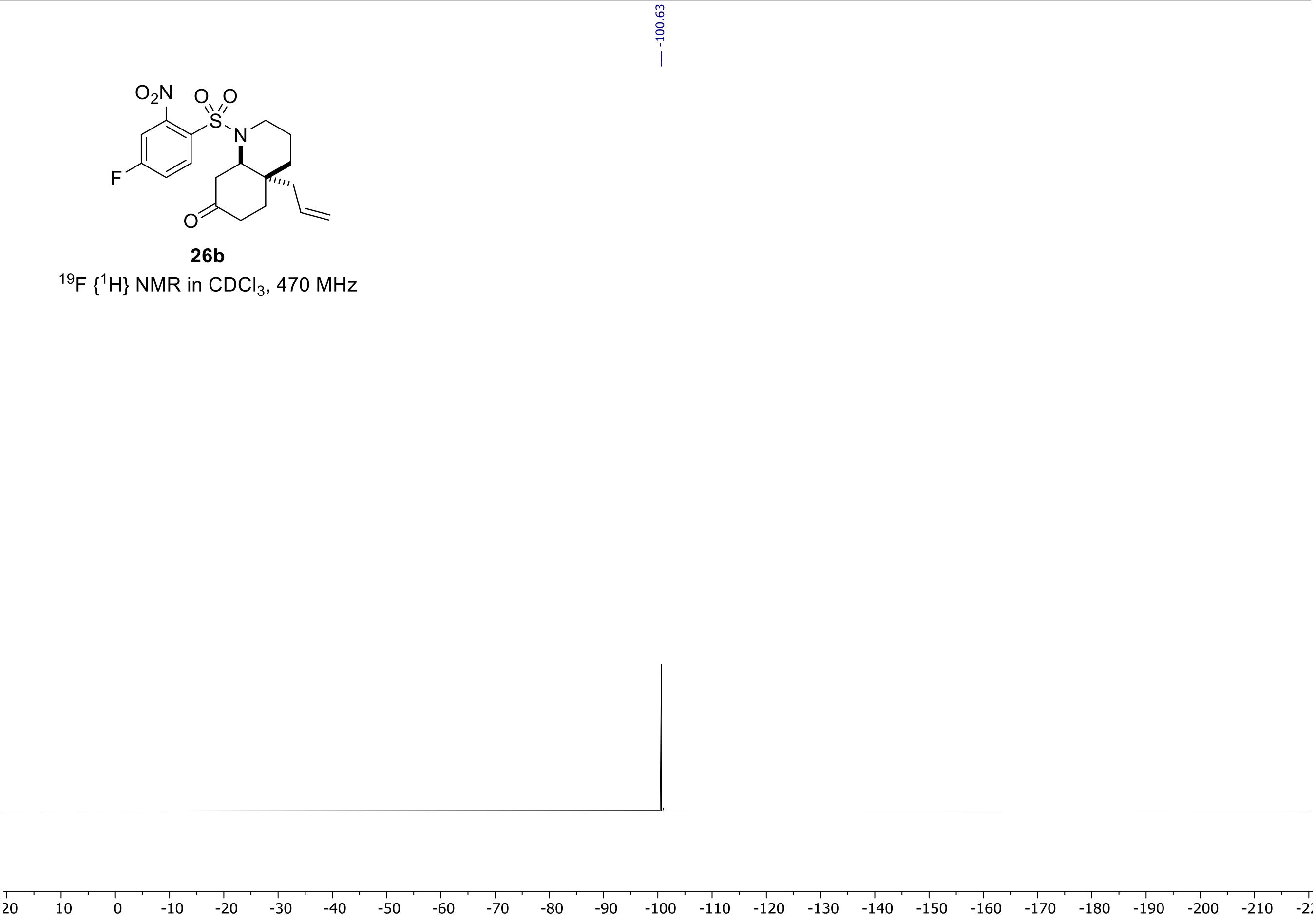

26b

$^{19}F\ \{^1H\}$ NMR in $CDCl_3$, 470 MHz

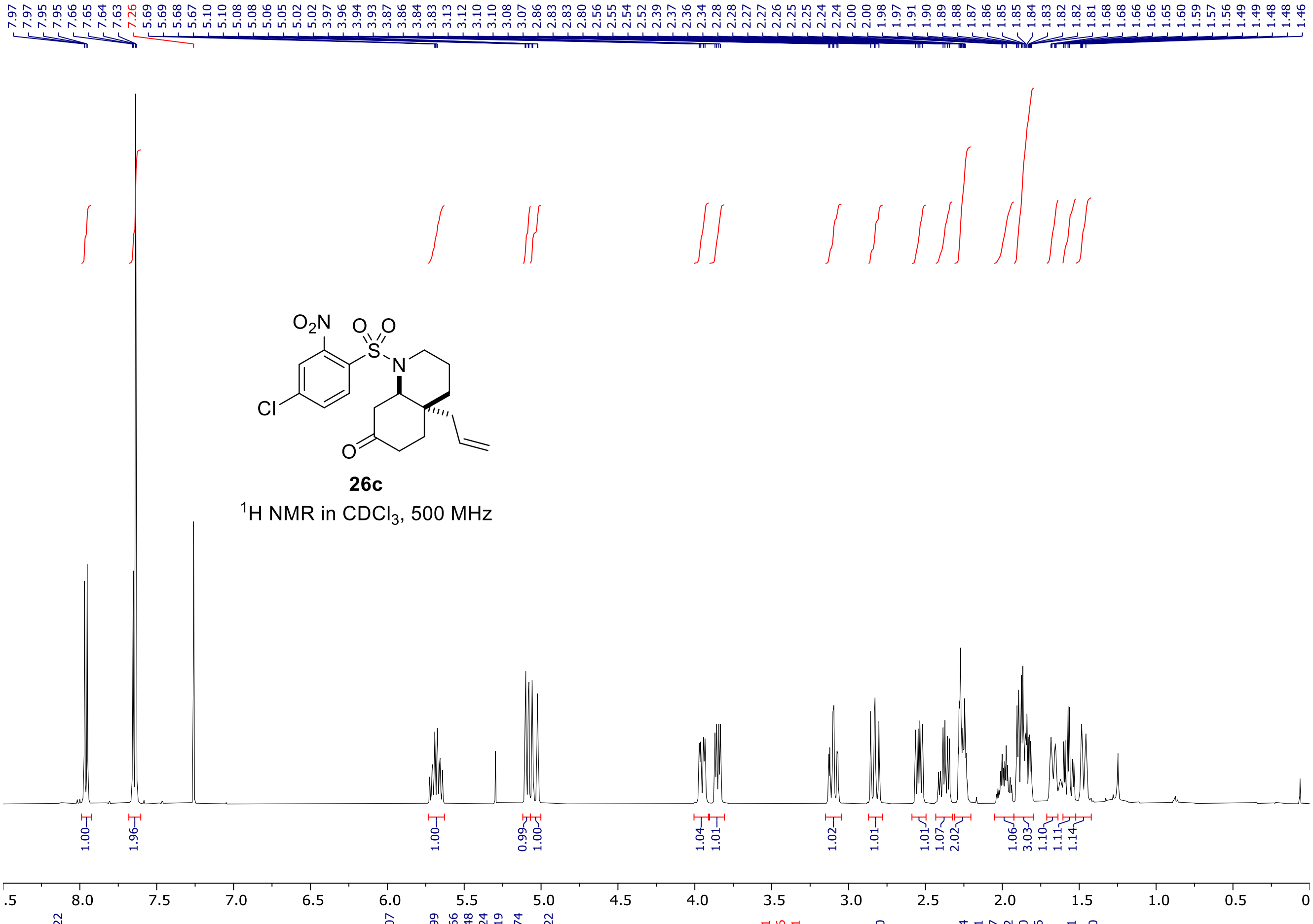
26c
^{1}H NMR in $CDCl_3$, 500 MHz

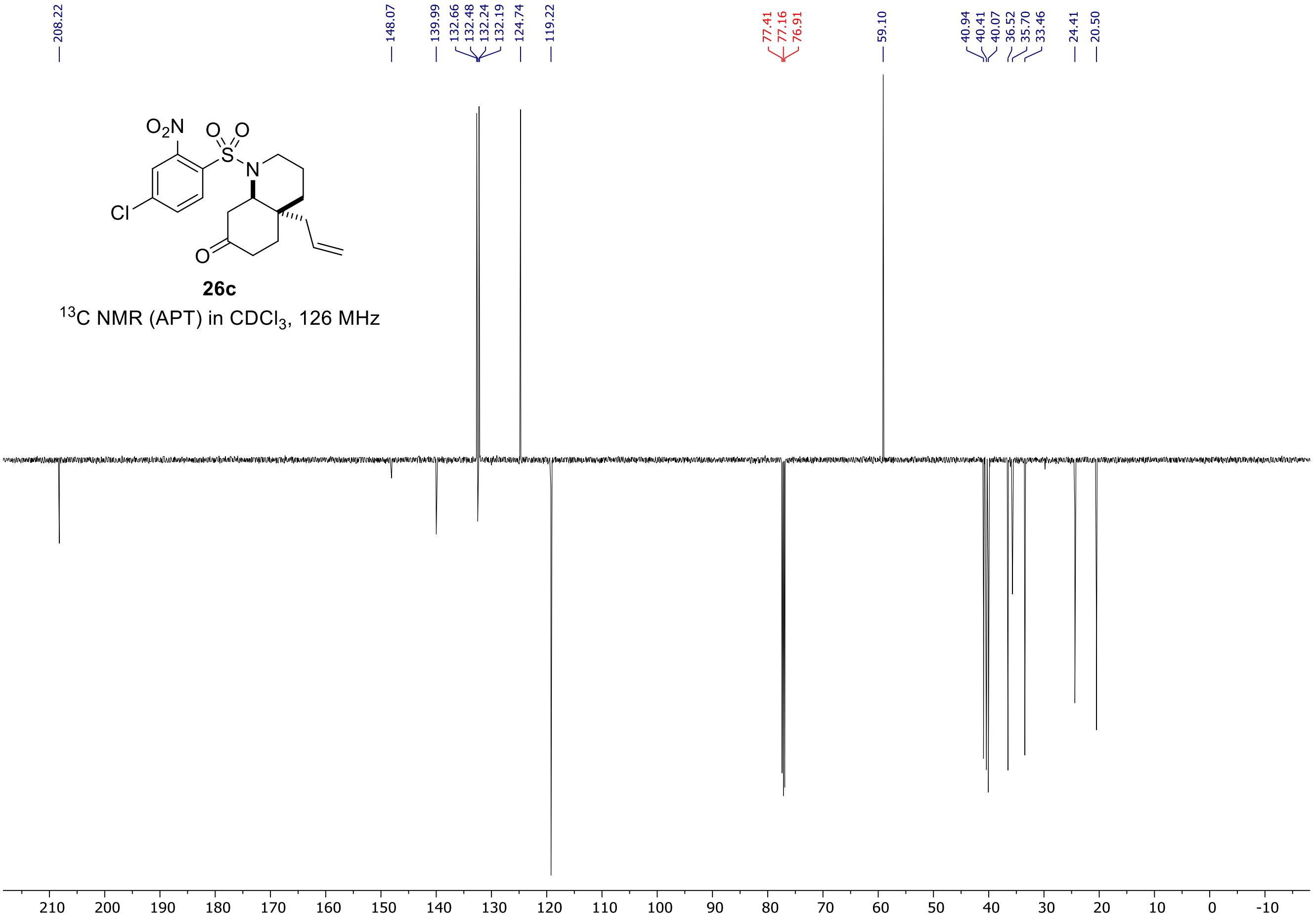
26c
^{13}C NMR (APT) in $CDCl_3$, 126 MHz

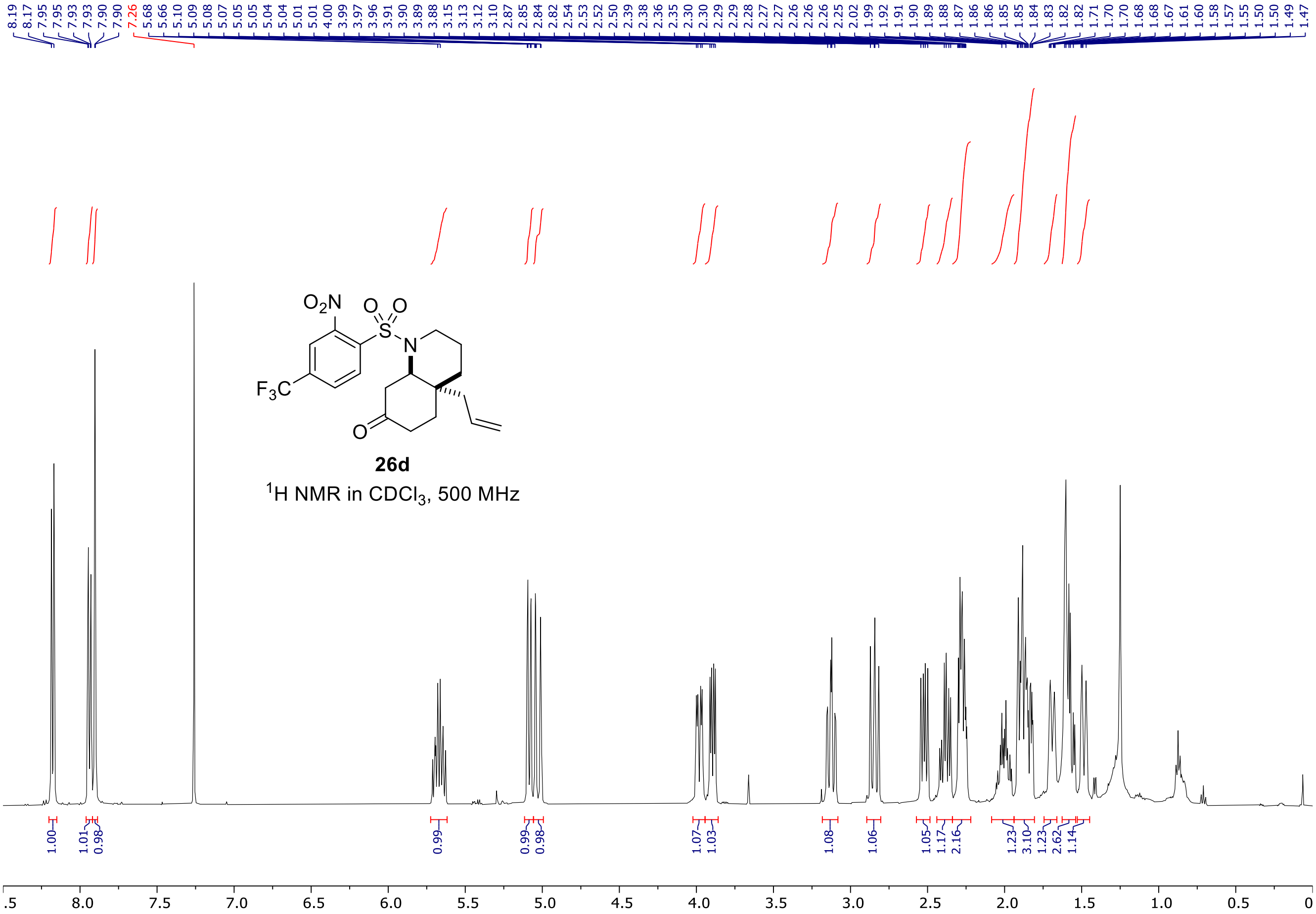
8.19 8.17 7.95 7.95 7.93 7.93 7.90 7.90 7.26 5.68 5.66 5.10 5.09 5.08 5.07 5.05 5.05 5.04 5.04 5.01 5.01 4.00 3.99 3.97 3.96 3.91 3.90 3.89 3.88 3.15 3.13 3.12 3.10 2.87 2.85 2.84 2.82 2.54 2.53 2.52 2.50 2.39 2.38 2.36 2.35 2.30 2.30 2.29 2.29 2.28 2.27 2.27 2.26 2.26 2.26 2.25 2.02 1.99 1.92 1.91 1.90 1.89 1.88 1.87 1.86 1.86 1.85 1.85 1.84 1.83 1.82 1.82 1.71 1.70 1.70 1.68 1.68 1.67 1.61 1.60 1.58 1.57 1.55 1.50 1.50 1.49 1.47
O2N
O O
S
N
F3C
O
26d
1H NMR in CDCl3, 500 MHz
1.00 1.01 0.98 0.99 0.99 0.98 1.07 1.03 1.08 1.06 1.05 1.17 2.16 1.23 3.10 1.23 2.62 1.14
.5 8.0 7.5 7.0 6.5 6.0 5.5 5.0 4.5 4.0 3.5 3.0 2.5 2.0 1.5 1.0 0.5 0

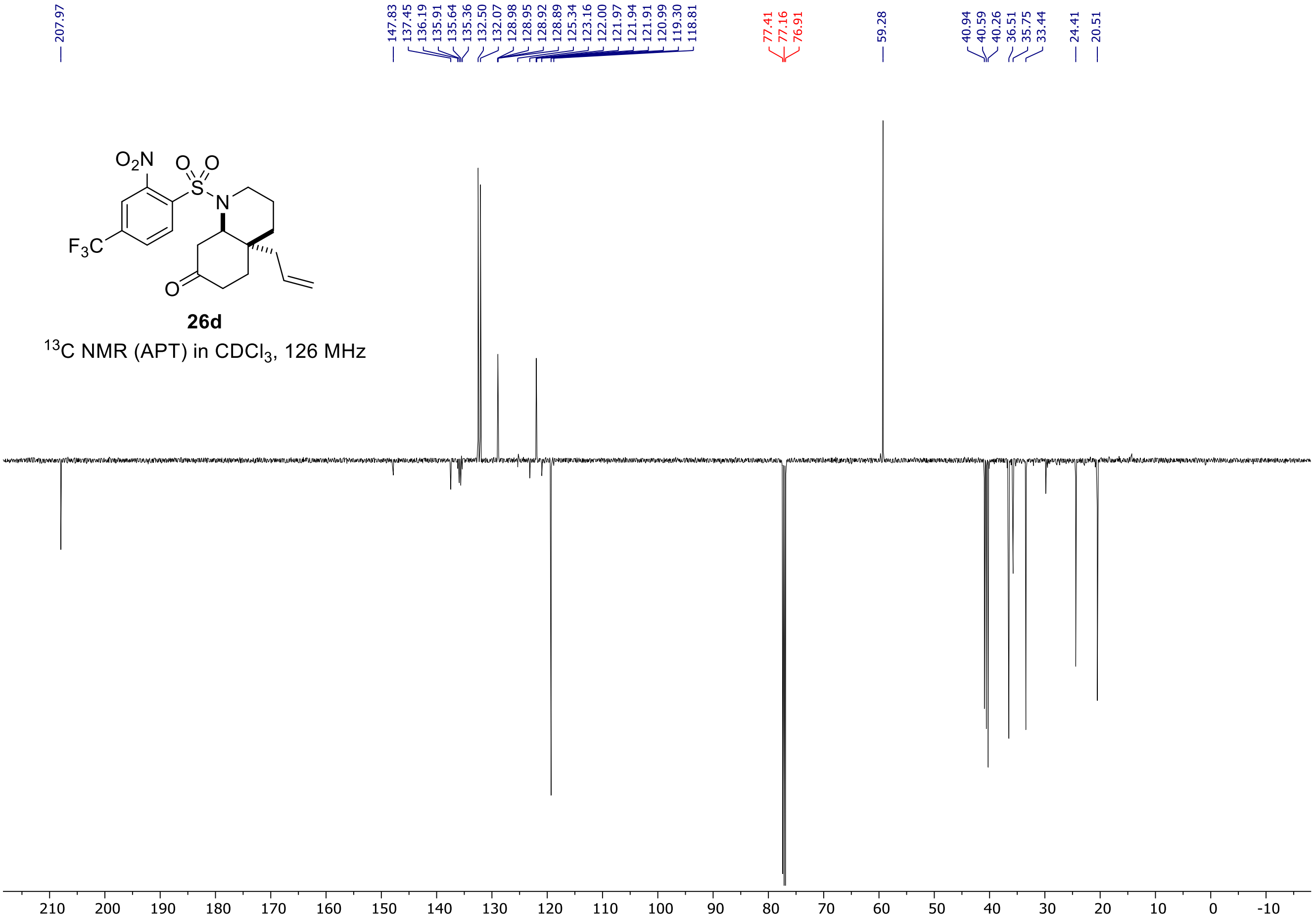
207.97
147.83 137.45 136.19 135.91 135.64 135.36 132.50 132.07 128.98 128.95 128.92 128.89 125.34 123.16 122.00 121.97 121.94 121.91 120.99 119.30 118.81
77.41 77.16 76.91
59.28
40.94 40.59 40.26 36.51 35.75 33.44
24.41 20.51
O2N
O O
S
N
F3C
O
26d
13C NMR (APT) in CDCl3, 126 MHz
210 200 190 180 170 160 150 140 130 120 110 100 90 80 70 60 50 40 30 20 10 0 -10

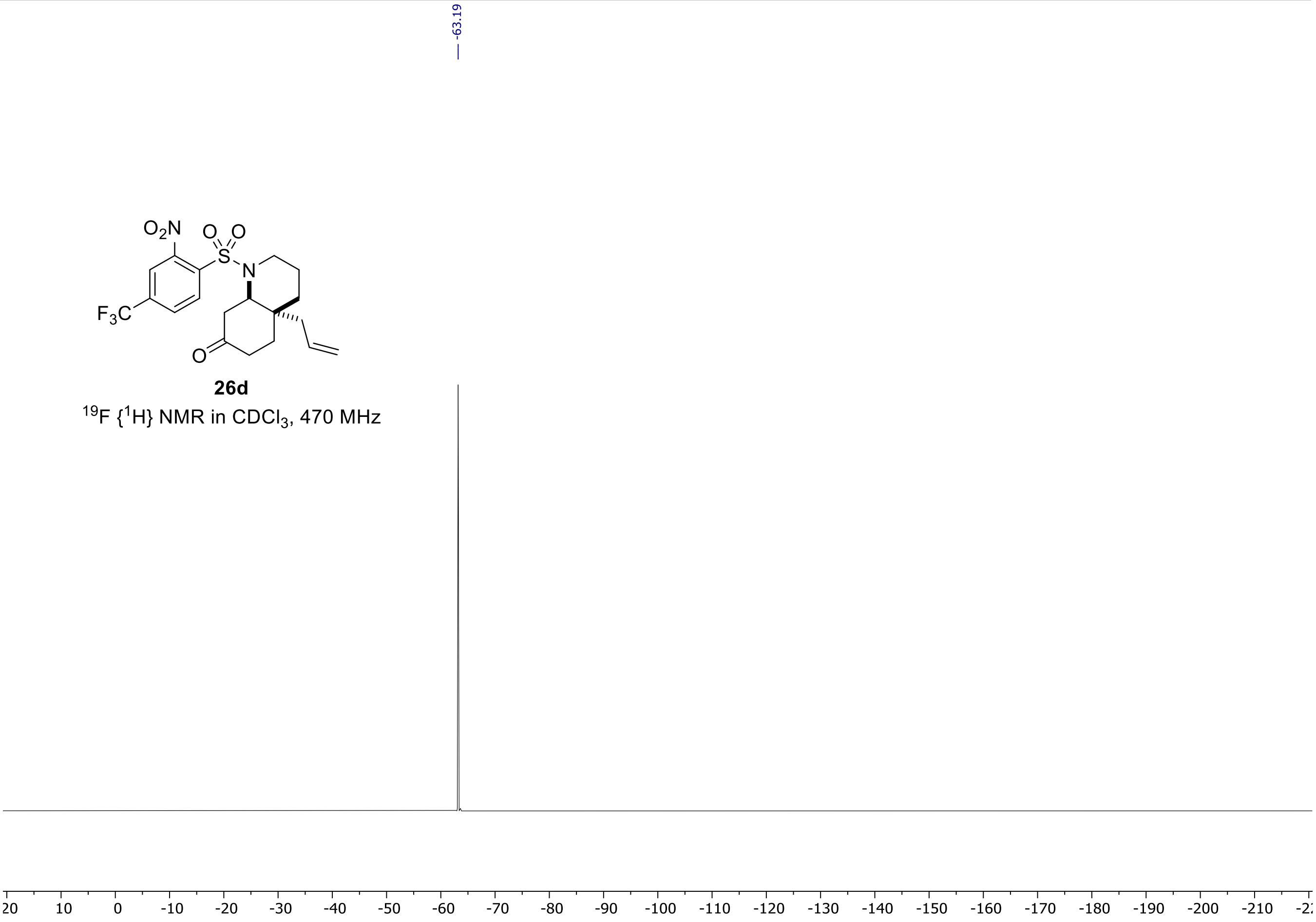

26d

$^{19}F\ \{^{1}H\}$ NMR in $CDCl_3$, 470 MHz

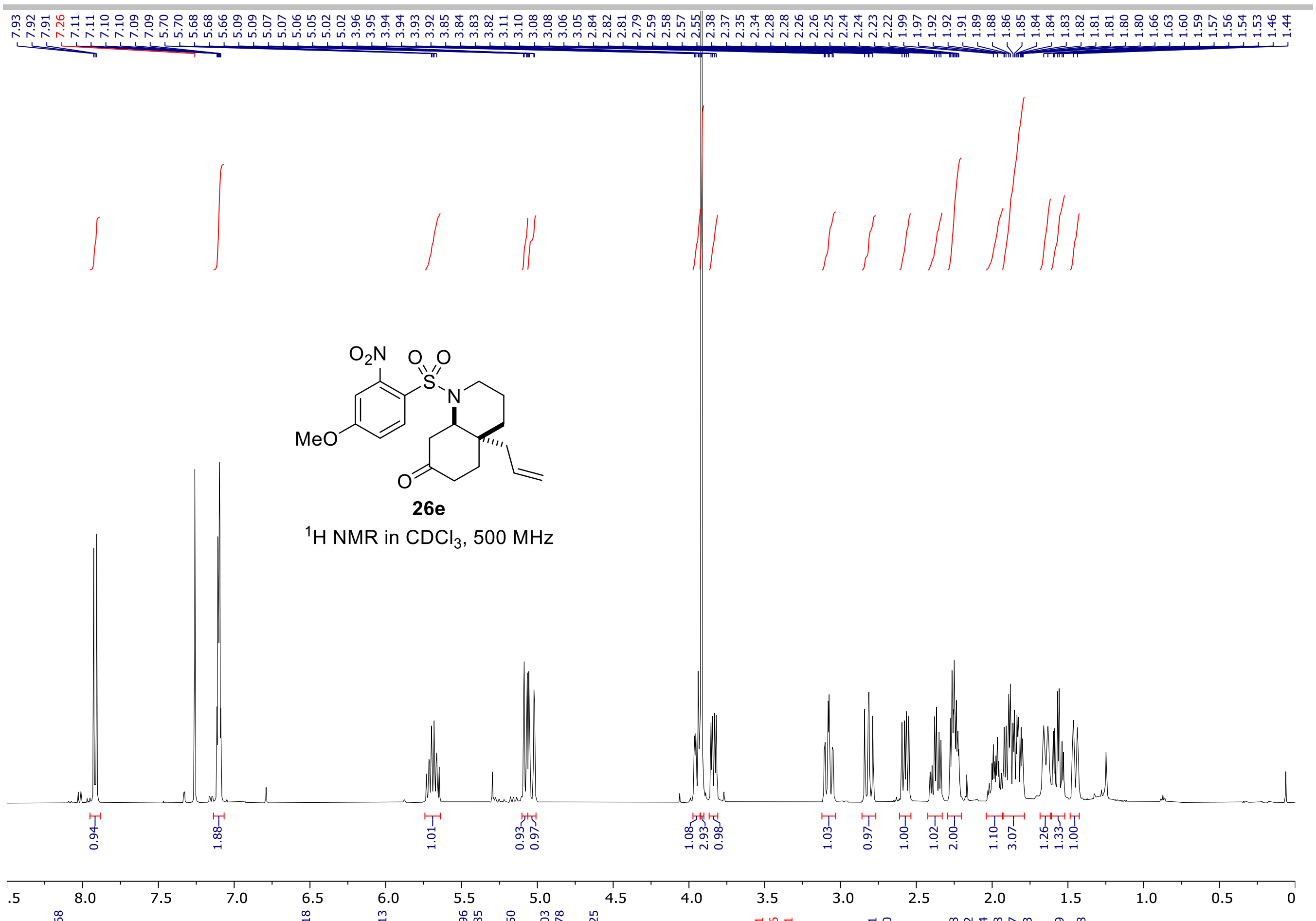
O2N
MeO
26e
1H NMR in CDCl3, 500 MHz

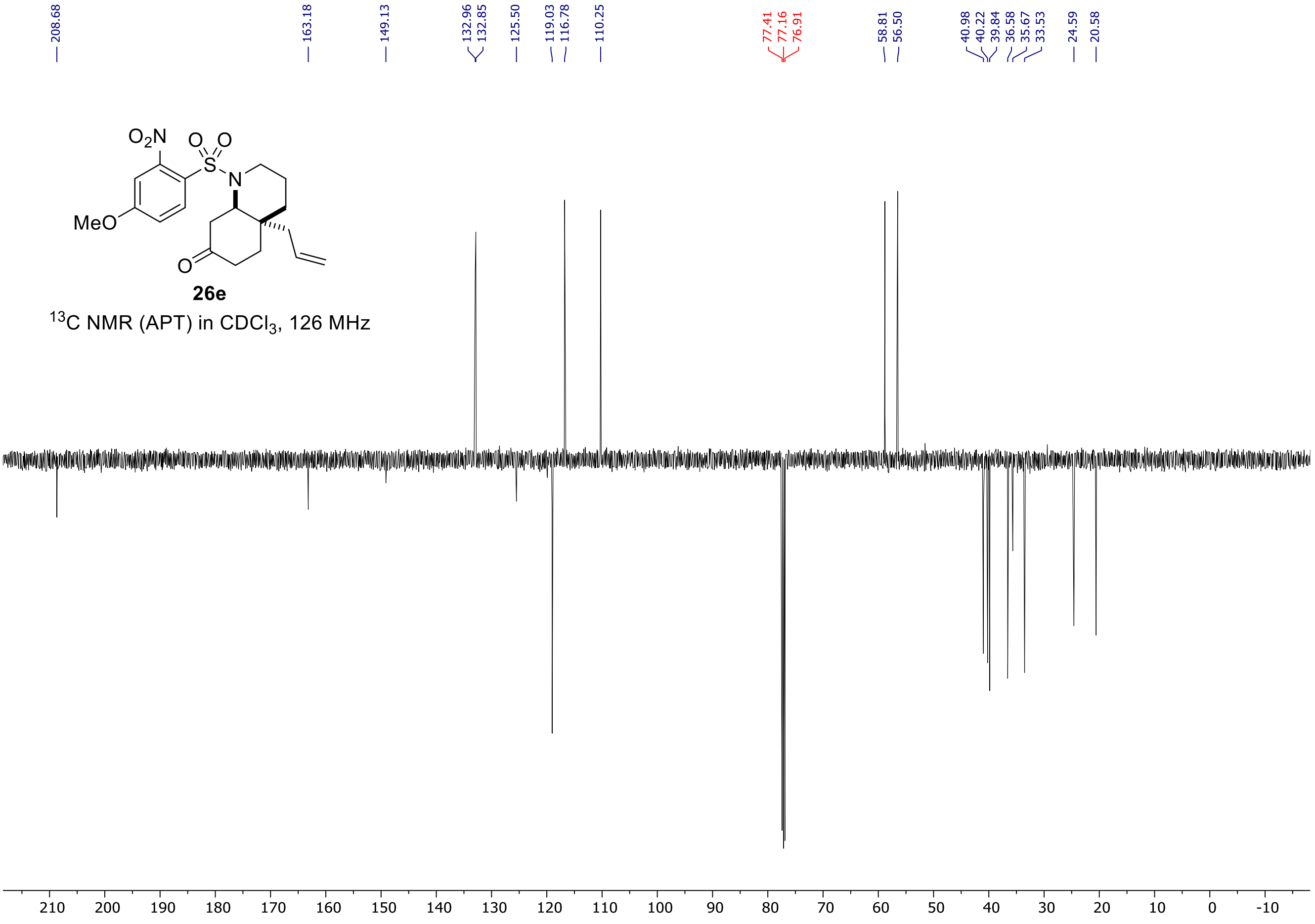
O2N
MeO
26e
13C NMR (APT) in CDCl3, 126 MHz

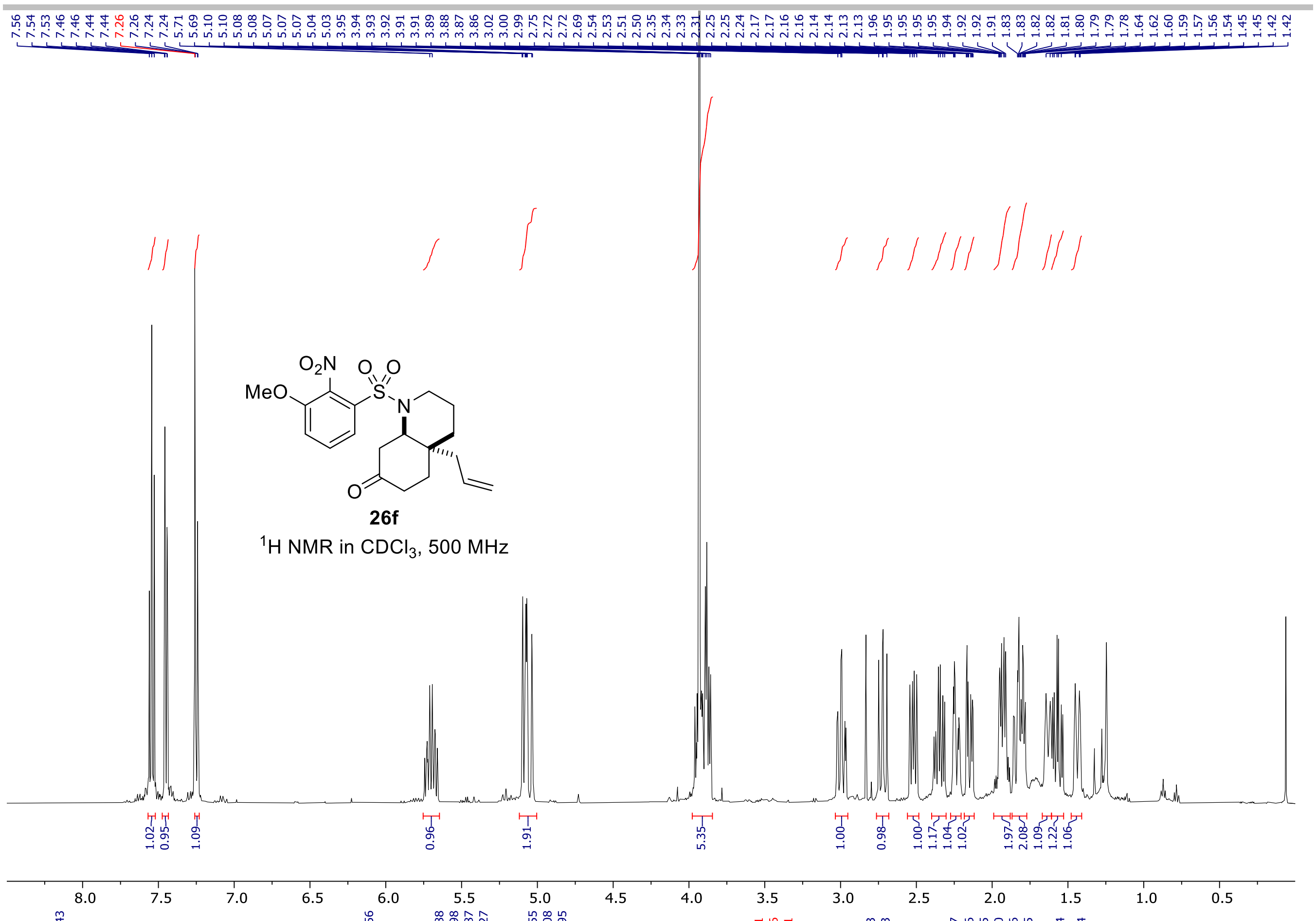
O2N
MeO
O O
S
N
O
26f
1H NMR in CDCl3, 500 MHz

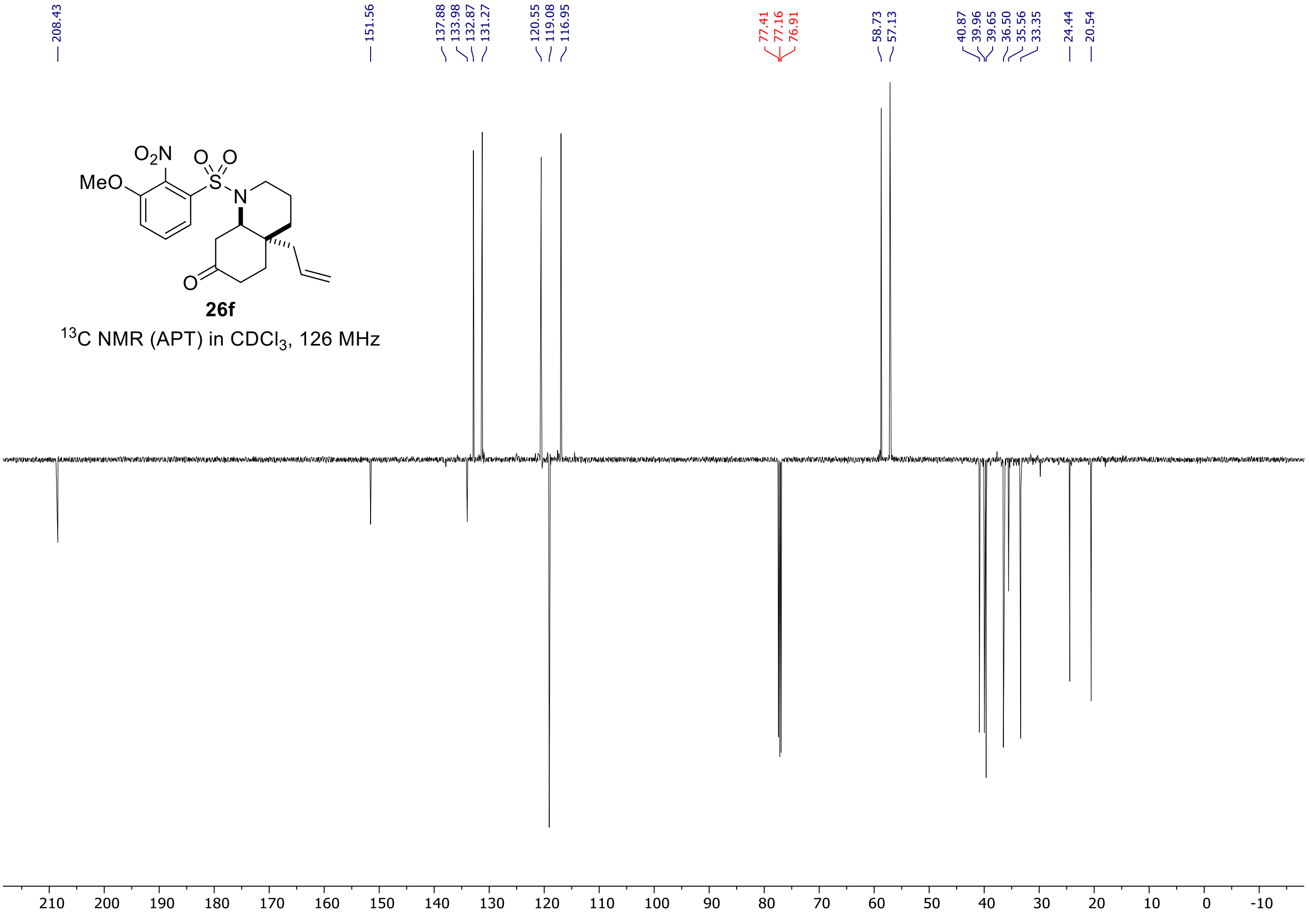
208.43
151.56
137.88
133.98
132.87
131.27
120.55
119.08
116.95
77.41
77.16
76.91
58.73
57.13
40.87
39.96
39.65
36.50
35.56
33.35
24.44
20.54
O2N
MeO
O O
S
N
O
26f
13C NMR (APT) in CDCl3, 126 MHz

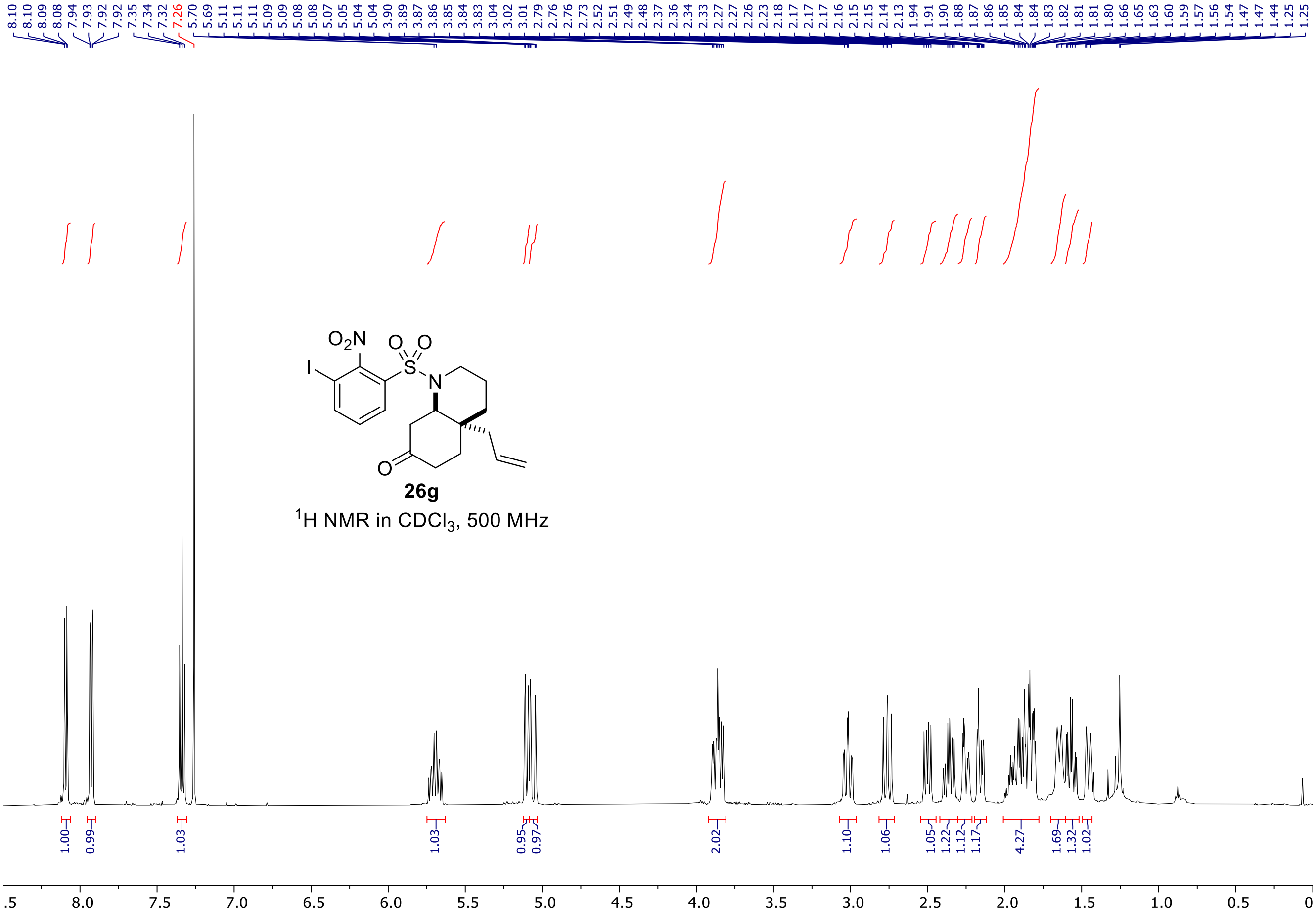
8.10 8.10 8.09 8.08 7.94 7.93 7.92 7.92 7.35 7.34 7.32 7.26 5.70 5.69 5.11 5.11 5.09 5.09 5.08 5.08 5.07 5.05 5.04 5.04 3.90 3.89 3.87 3.86 3.85 3.84 3.83 3.04 3.02 3.01 2.79 2.76 2.76 2.73 2.52 2.51 2.49 2.48 2.37 2.36 2.34 2.33 2.27 2.27 2.26 2.23 2.18 2.17 2.17 2.17 2.16 2.15 2.15 2.14 2.13 1.94 1.91 1.90 1.88 1.87 1.86 1.85 1.84 1.84 1.83 1.82 1.81 1.81 1.80 1.66 1.65 1.63 1.60 1.59 1.57 1.56 1.54 1.47 1.47 1.44 1.25 1.25
O2N
I
O O
S
N
O
26g
1H NMR in CDCl3, 500 MHz
1.00 0.99 1.03 1.03 0.95 0.97 2.02 1.10 1.06 1.05 1.22 1.12 1.17 4.27 1.69 1.32 1.02

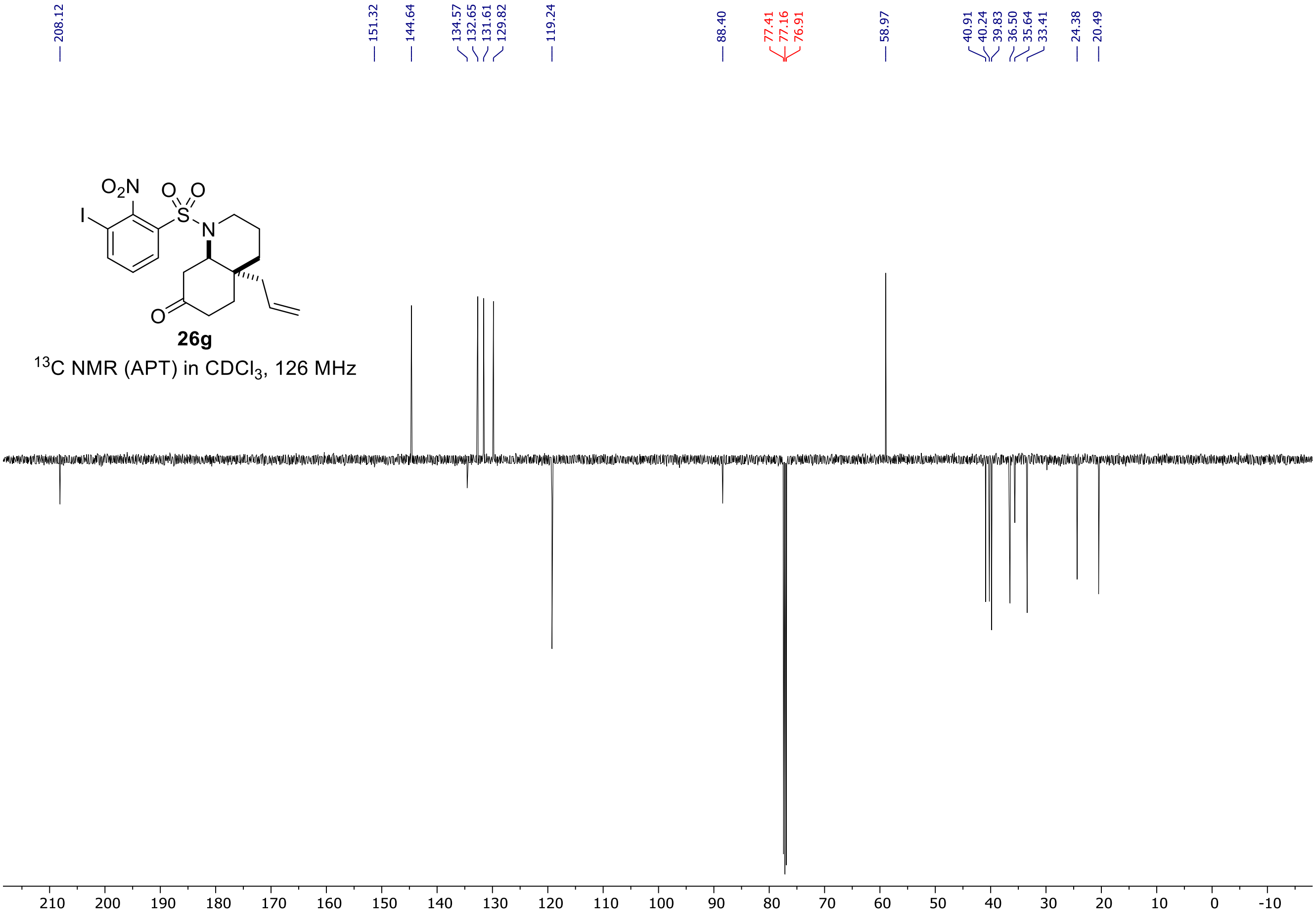
208.12 151.32 144.64 134.57 132.65 131.61 129.82 119.24 88.40 77.41 77.16 76.91 58.97 40.91 40.24 39.83 36.50 35.64 33.41 24.38 20.49
O2N
I
O O
S
N
O
26g
13C NMR (APT) in CDCl3, 126 MHz

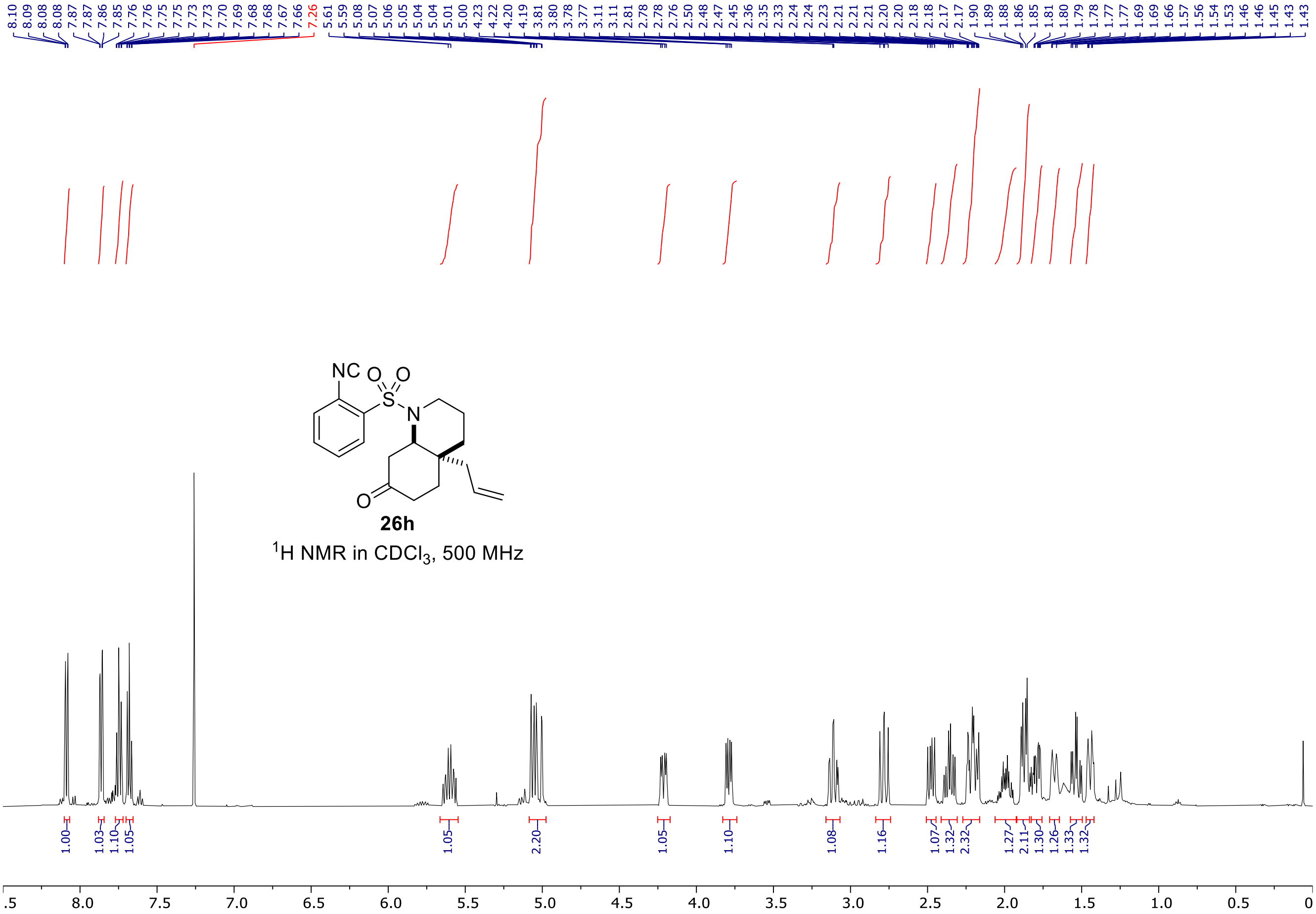
NC
O
O
S
N
O
26h
1H NMR in CDCl3, 500 MHz

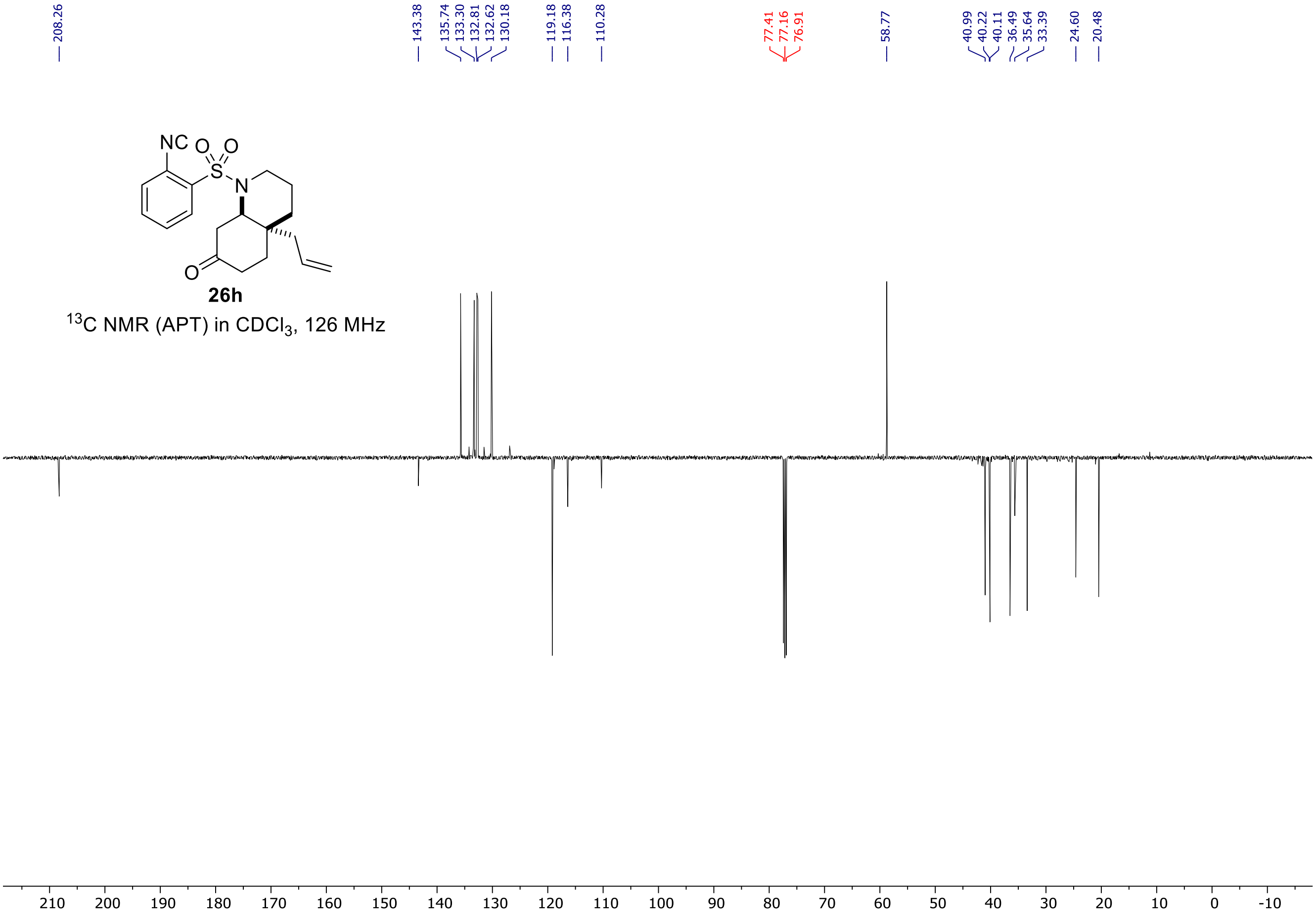
208.26
143.38
135.74
133.30
132.81
132.62
130.18
119.18
116.38
110.28
77.41
77.16
76.91
58.77
40.99
40.22
40.11
36.49
35.64
33.39
24.60
20.48
NC
O
O
S
N
O
26h
13C NMR (APT) in CDCl3, 126 MHz

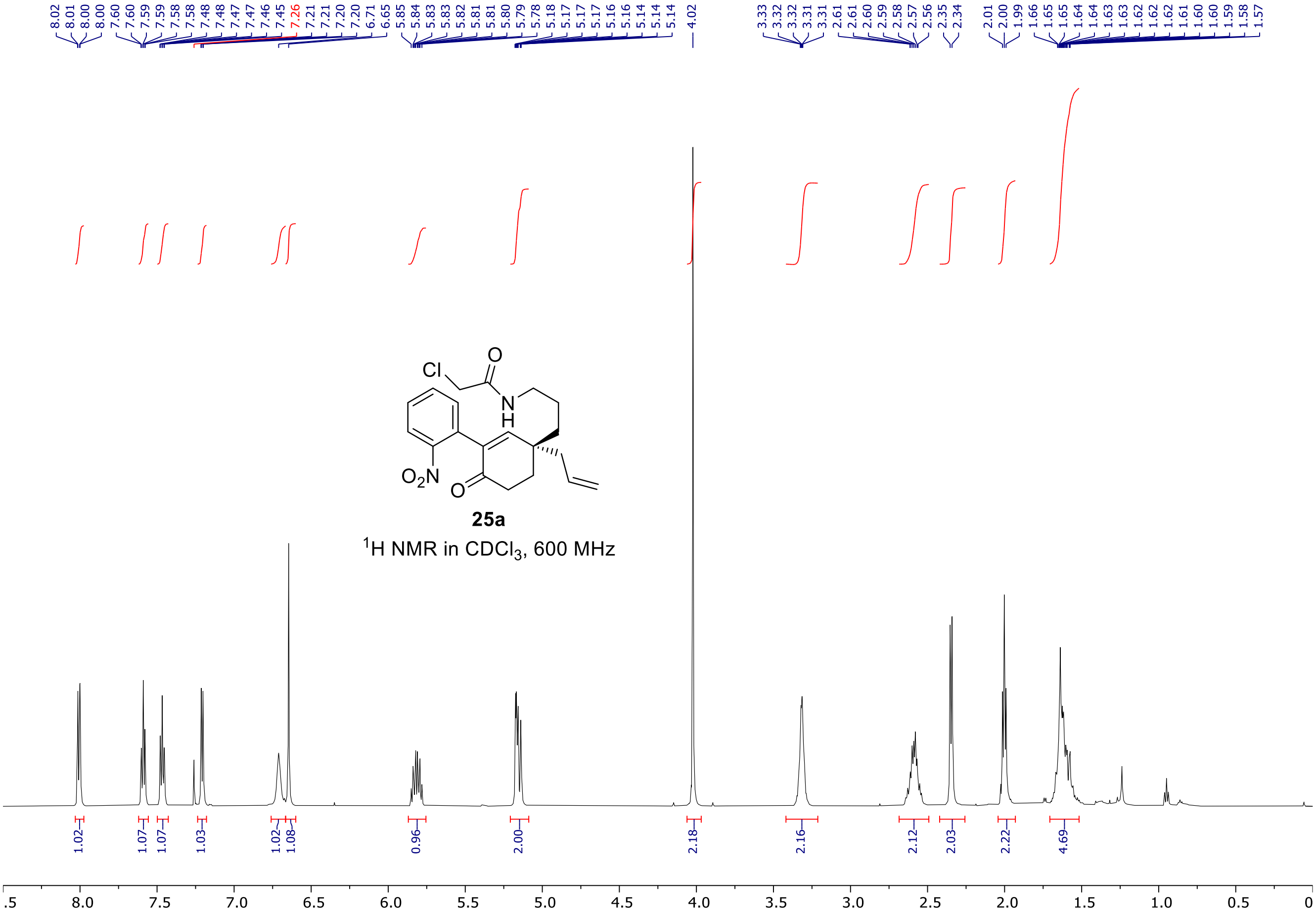
25a
^{1}H NMR in CDCl$_3$, 600 MHz

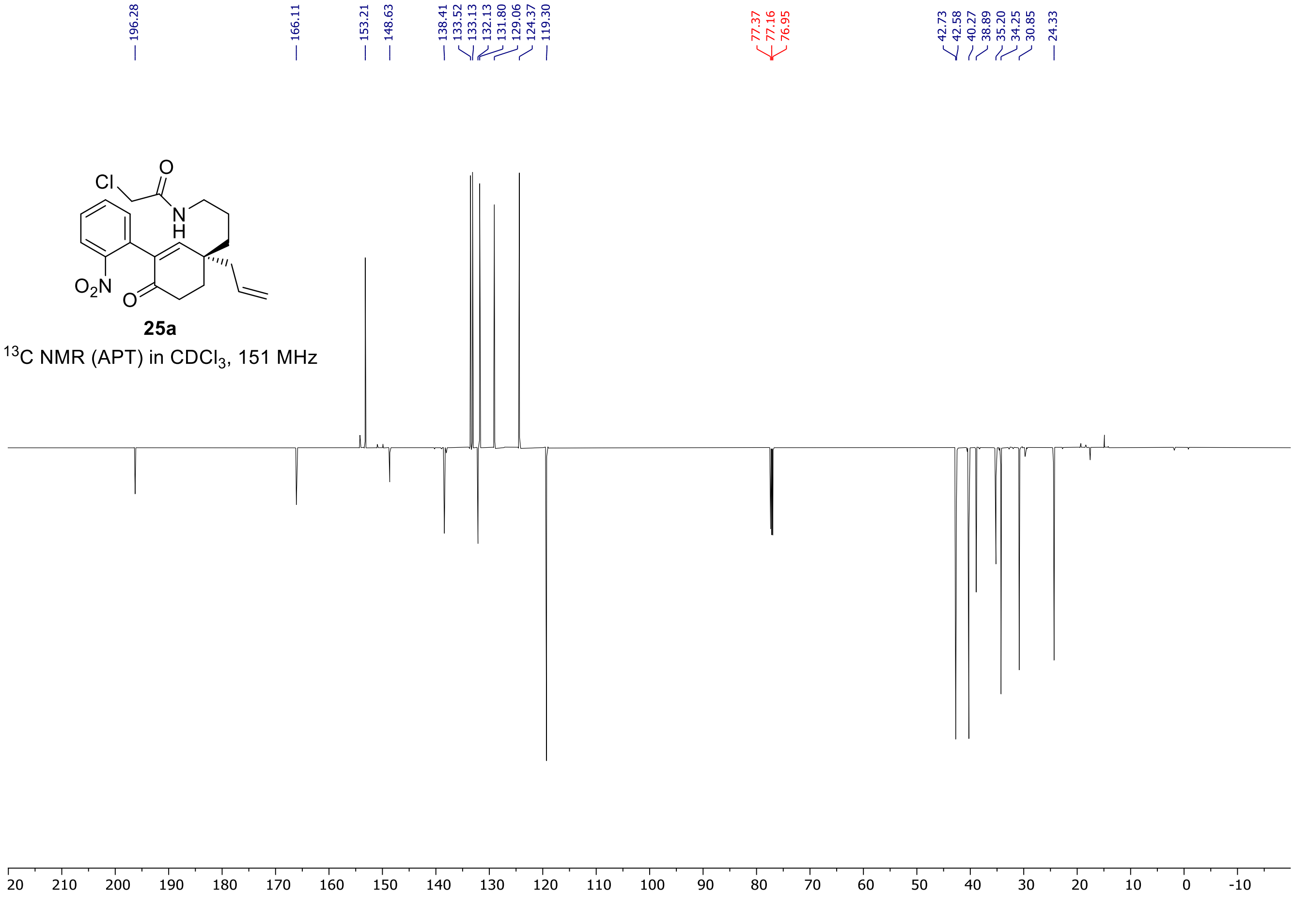
25a
^{13}C NMR (APT) in CDCl$_3$, 151 MHz

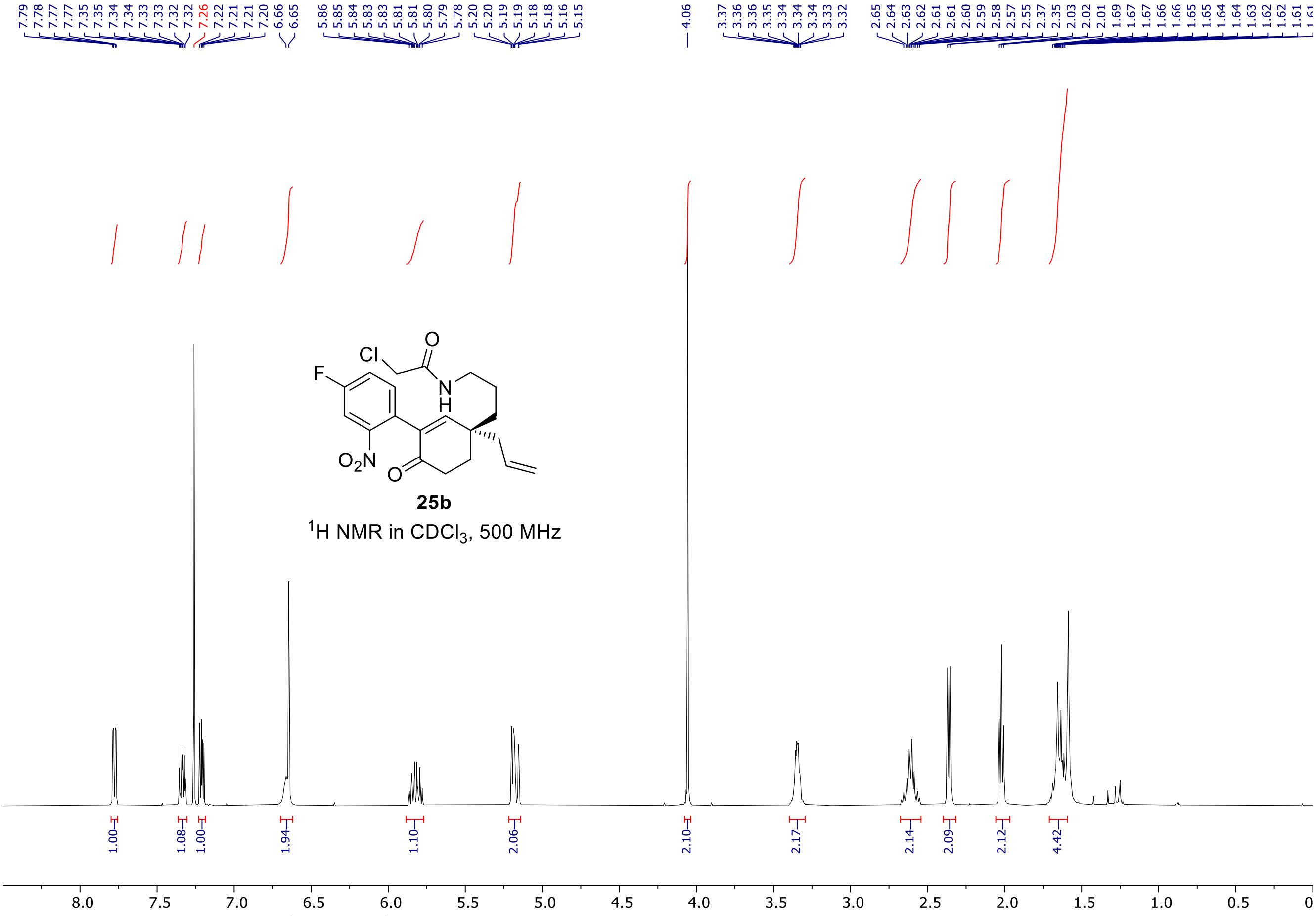
7.79 7.78 7.77 7.77 7.35 7.34 7.34 7.33 7.33 7.32 7.32 7.32 7.26 7.22 7.21 7.21 7.20 6.66 6.65 5.86 5.85 5.84 5.83 5.83 5.81 5.81 5.80 5.79 5.78 5.20 5.20 5.19 5.19 5.18 5.18 5.16 5.15 4.06 3.37 3.36 3.36 3.35 3.34 3.34 3.34 3.33 3.32 2.65 2.64 2.63 2.62 2.61 2.61 2.60 2.59 2.58 2.57 2.55 2.37 2.35 2.03 2.02 2.01 1.69 1.67 1.66 1.66 1.65 1.65 1.64 1.64 1.63 1.62 1.62 1.61
Cl
O
F
N
H
O_2N
O
25b
^{1}H NMR in $CDCl_3$, 500 MHz
1.00 1.08 1.00 1.94 1.10 2.06 2.10 2.17 2.14 2.09 2.12 4.42
8.0 7.5 7.0 6.5 6.0 5.5 5.0 4.5 4.0 3.5 3.0 2.5 2.0 1.5 1.0 0.5 0

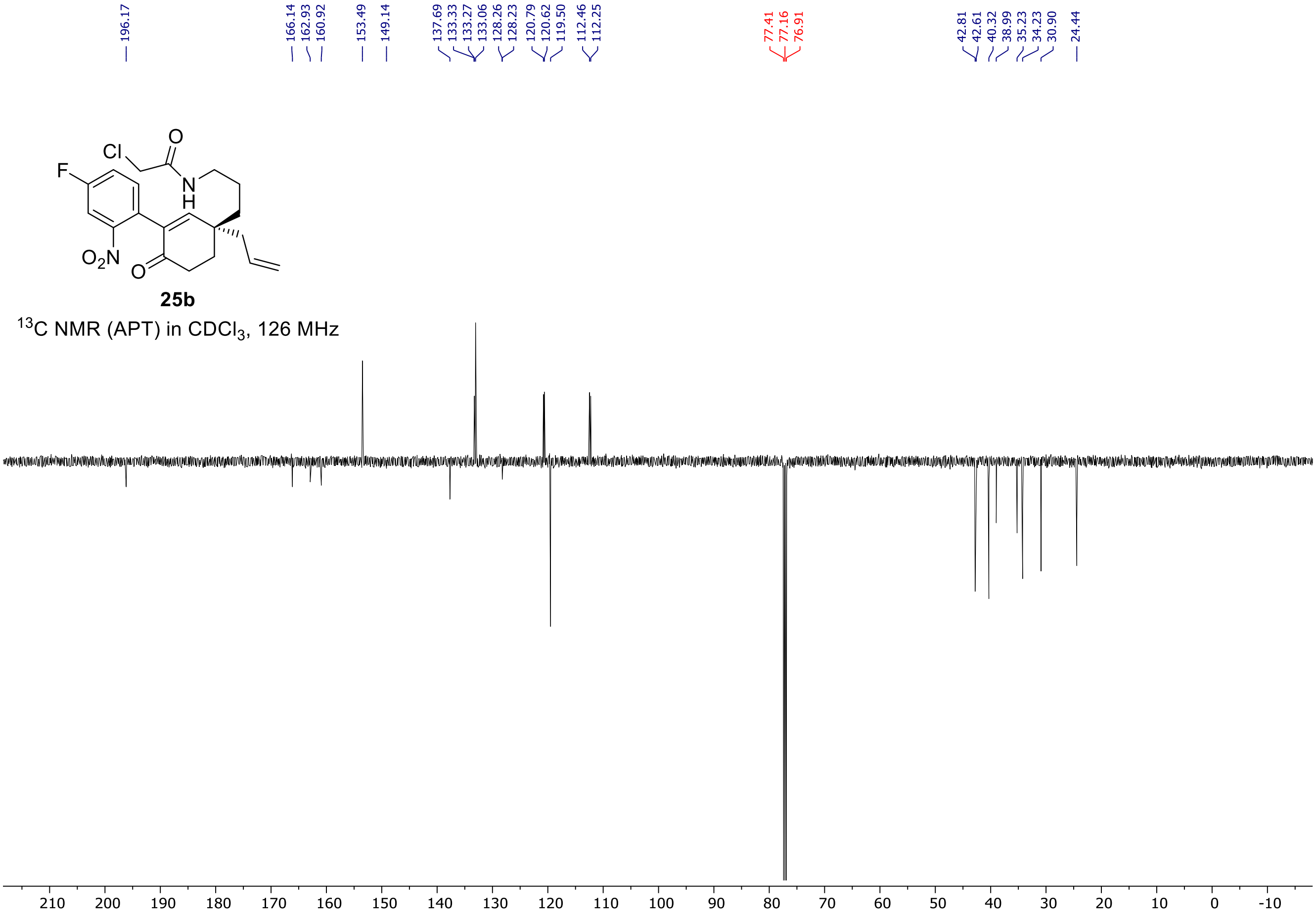
196.17 166.14 162.93 160.92 153.49 149.14 137.69 133.33 133.27 133.06 128.26 128.23 120.79 120.62 119.50 112.46 112.25 77.41 77.16 76.91 42.81 42.61 40.32 38.99 35.23 34.23 30.90 24.44
Cl
O
F
N
H
O_2N
O
25b
^{13}C NMR (APT) in $CDCl_3$, 126 MHz
210 200 190 180 170 160 150 140 130 120 110 100 90 80 70 60 50 40 30 20 10 0 -10

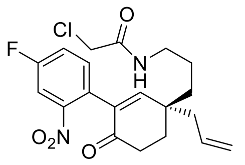

25b

$^{19}F\ \{^{1}H\}$ NMR in $CDCl_3$, 470 MHz

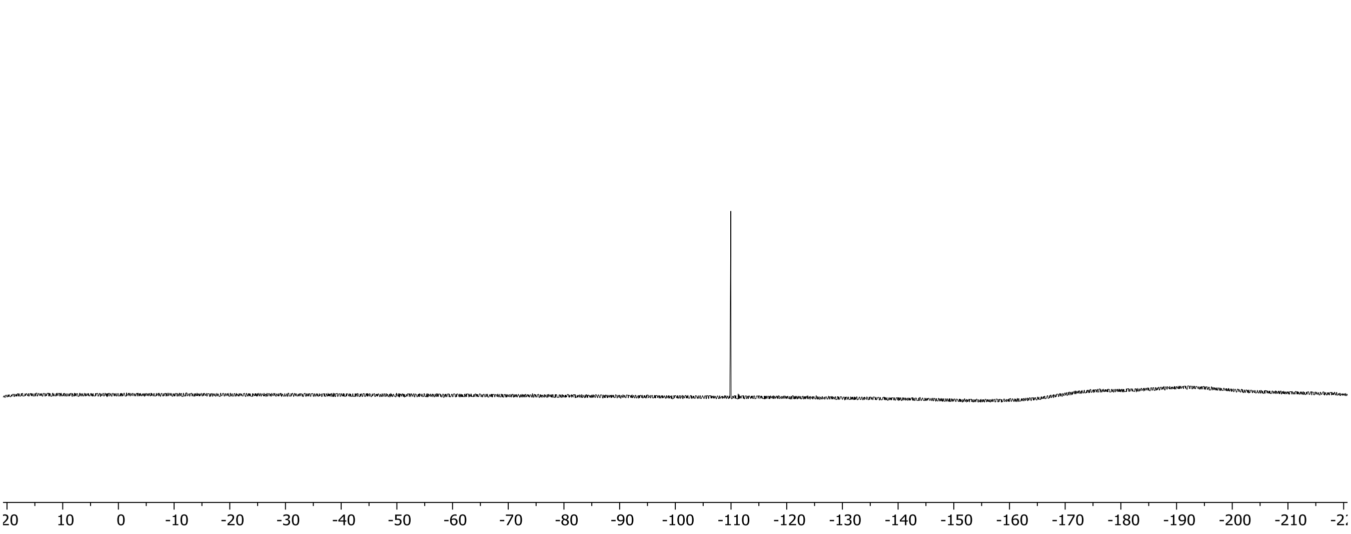

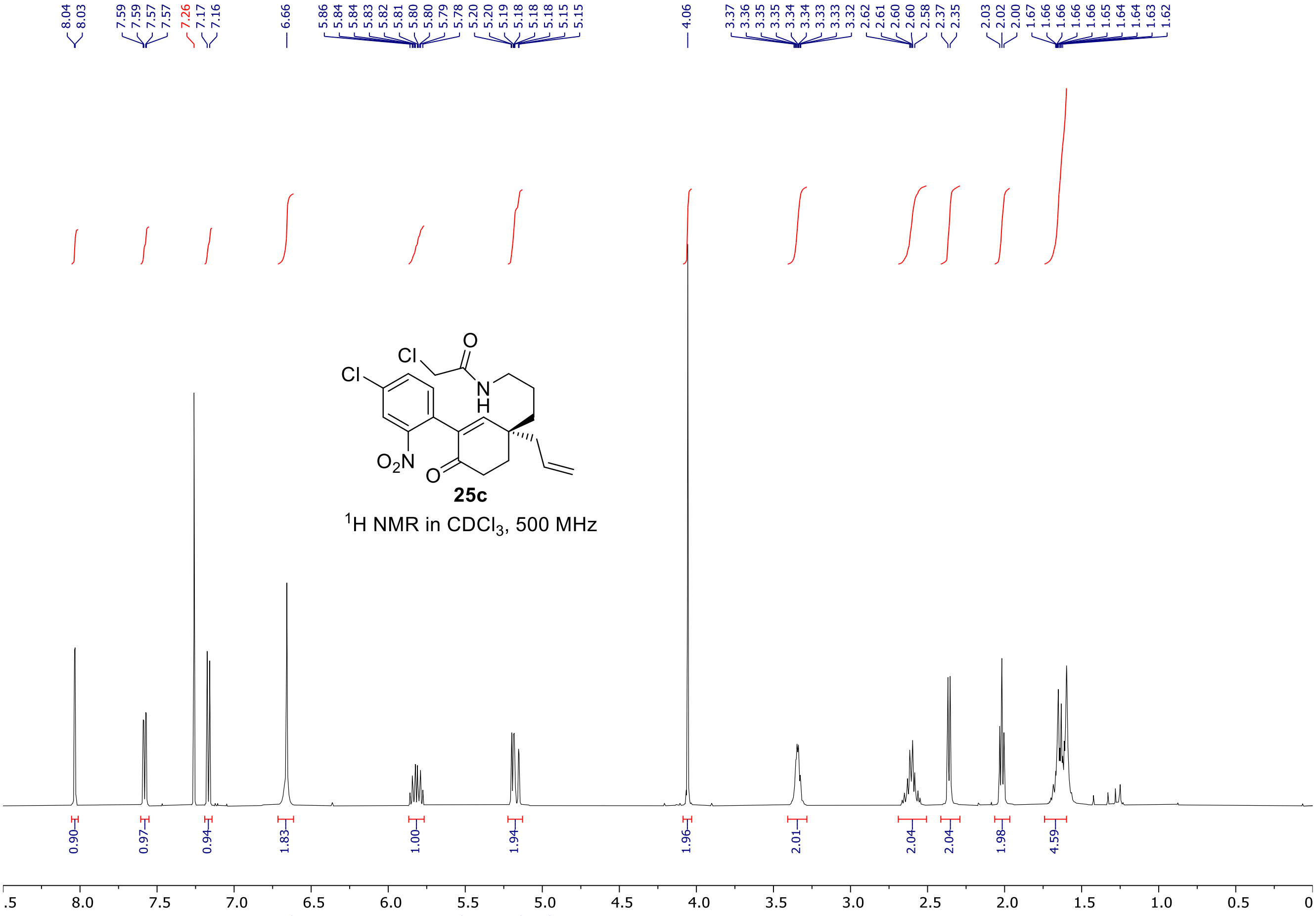
8.04 8.03 7.59 7.59 7.57 7.57 7.26 7.17 7.16 6.66 5.86 5.84 5.84 5.83 5.82 5.81 5.80 5.80 5.79 5.78 5.20 5.20 5.19 5.18 5.18 5.18 5.15 5.15 4.06 3.37 3.36 3.35 3.35 3.34 3.34 3.33 3.33 3.32 2.62 2.61 2.60 2.60 2.58 2.37 2.35 2.03 2.02 2.00 1.67 1.66 1.66 1.66 1.65 1.64 1.64 1.63 1.62
Cl
O
Cl
N
H
O_2N
O
25c
^{1}H NMR in $CDCl_3$, 500 MHz
0.90 0.97 0.94 1.83 1.00 1.94 1.96 2.01 2.04 2.04 1.98 4.59
.5 8.0 7.5 7.0 6.5 6.0 5.5 5.0 4.5 4.0 3.5 3.0 2.5 2.0 1.5 1.0 0.5 0

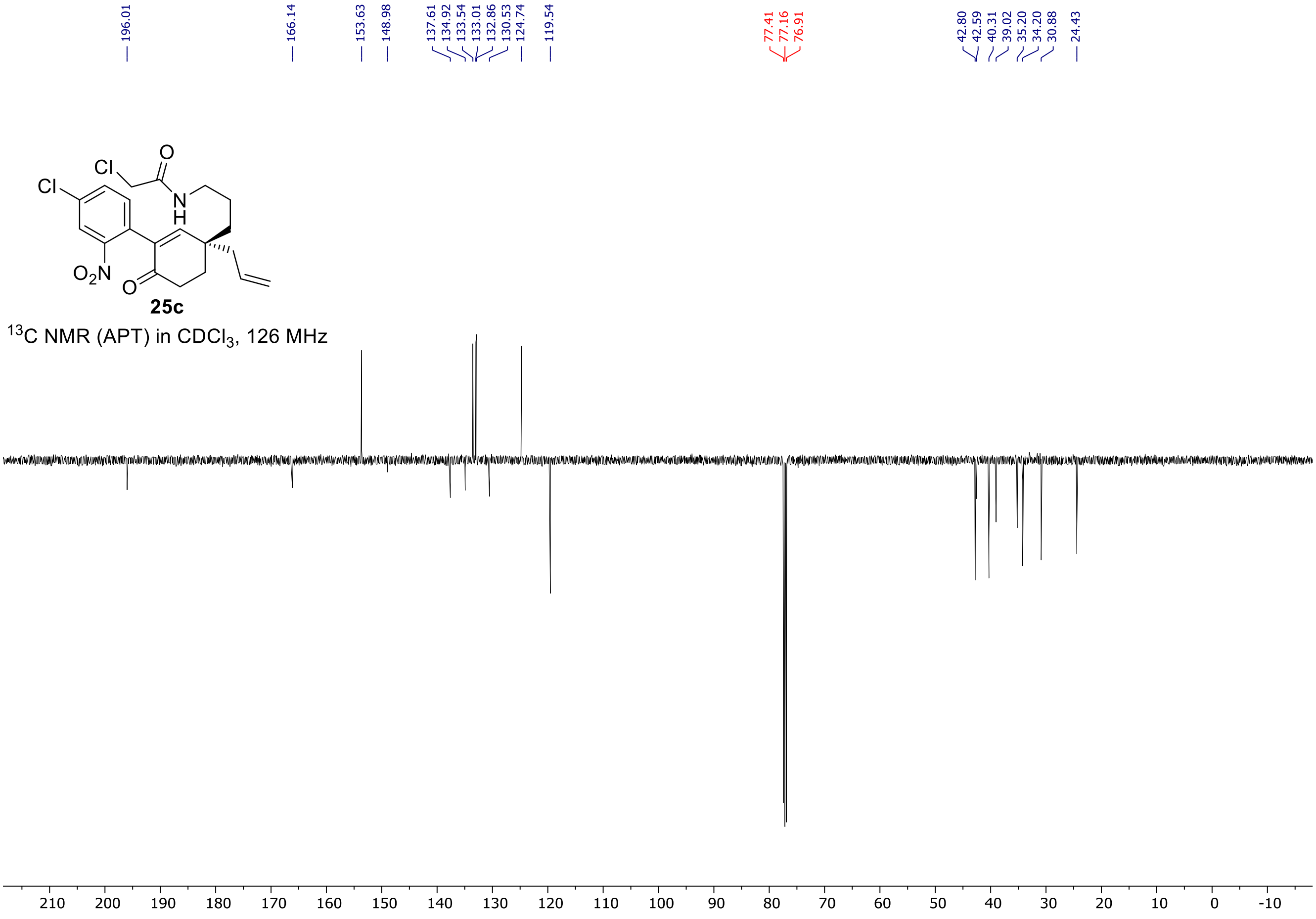
196.01 166.14 153.63 148.98 137.61 134.92 133.54 133.01 132.86 130.53 124.74 119.54 77.41 77.16 76.91 42.80 42.59 40.31 39.02 35.20 34.20 30.88 24.43
Cl
O
Cl
N
H
O_2N
O
25c
^{13}C NMR (APT) in $CDCl_3$, 126 MHz
210 200 190 180 170 160 150 140 130 120 110 100 90 80 70 60 50 40 30 20 10 0 -10

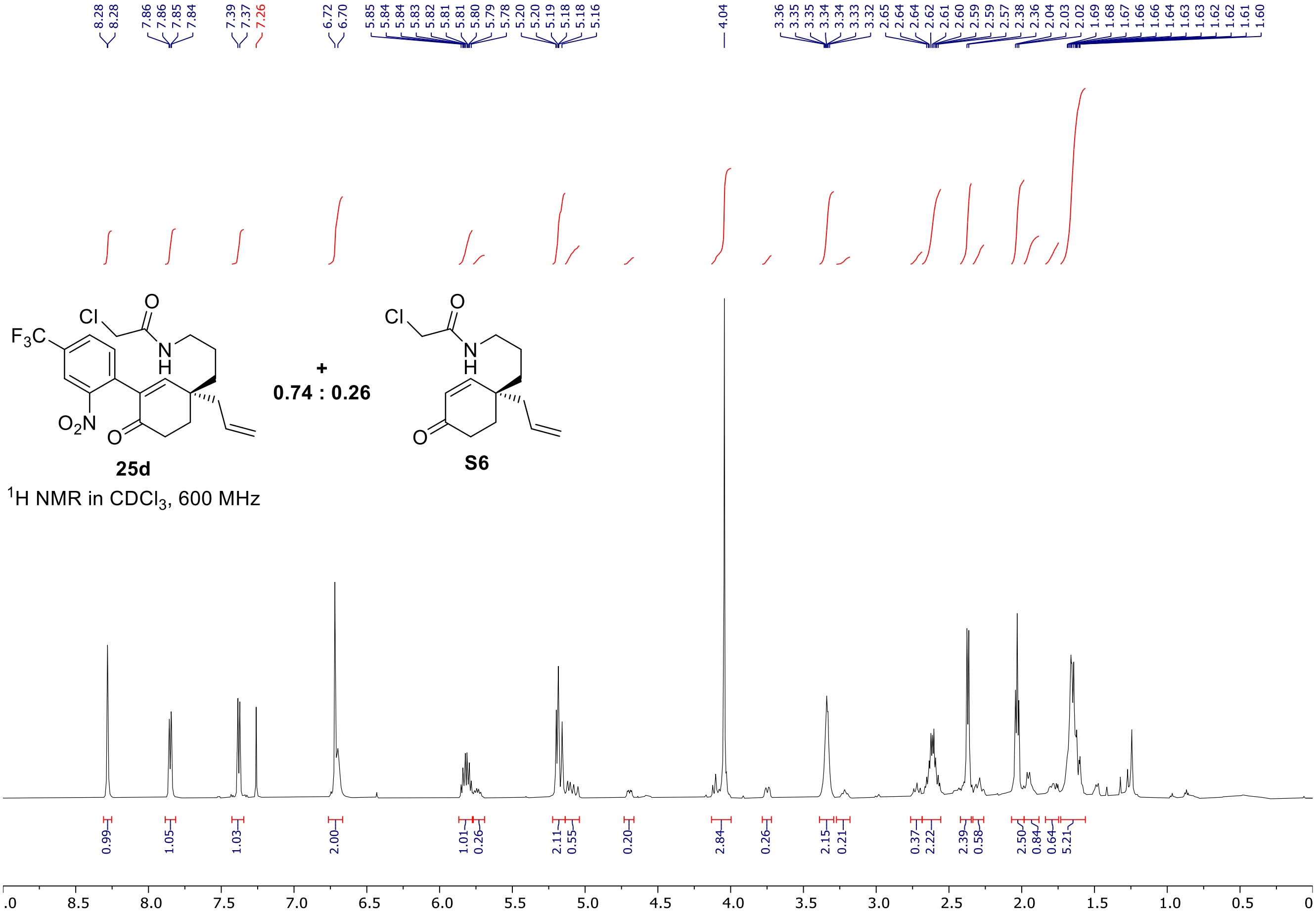
8.28 8.28 7.86 7.86 7.85 7.84 7.39 7.37 7.26 6.72 6.70 5.85 5.84 5.84 5.83 5.82 5.81 5.81 5.80 5.79 5.78 5.20 5.20 5.19 5.18 5.18 5.16 4.04 3.36 3.35 3.35 3.34 3.34 3.33 3.32 2.65 2.64 2.64 2.62 2.61 2.60 2.59 2.59 2.57 2.38 2.36 2.04 2.03 2.02 1.69 1.68 1.67 1.66 1.66 1.64 1.63 1.63 1.62 1.62 1.61 1.60
Cl
O
F3C
N
H
O2N
O
+
0.74 : 0.26
S6
25d
1H NMR in CDCl3, 600 MHz
0.99 1.05 1.03 2.00 1.01 0.26 2.11 0.55 0.20 2.84 0.26 2.15 0.21 0.37 2.22 2.39 0.58 2.50 0.84 0.64 5.21
.0 8.5 8.0 7.5 7.0 6.5 6.0 5.5 5.0 4.5 4.0 3.5 3.0 2.5 2.0 1.5 1.0 0.5 0

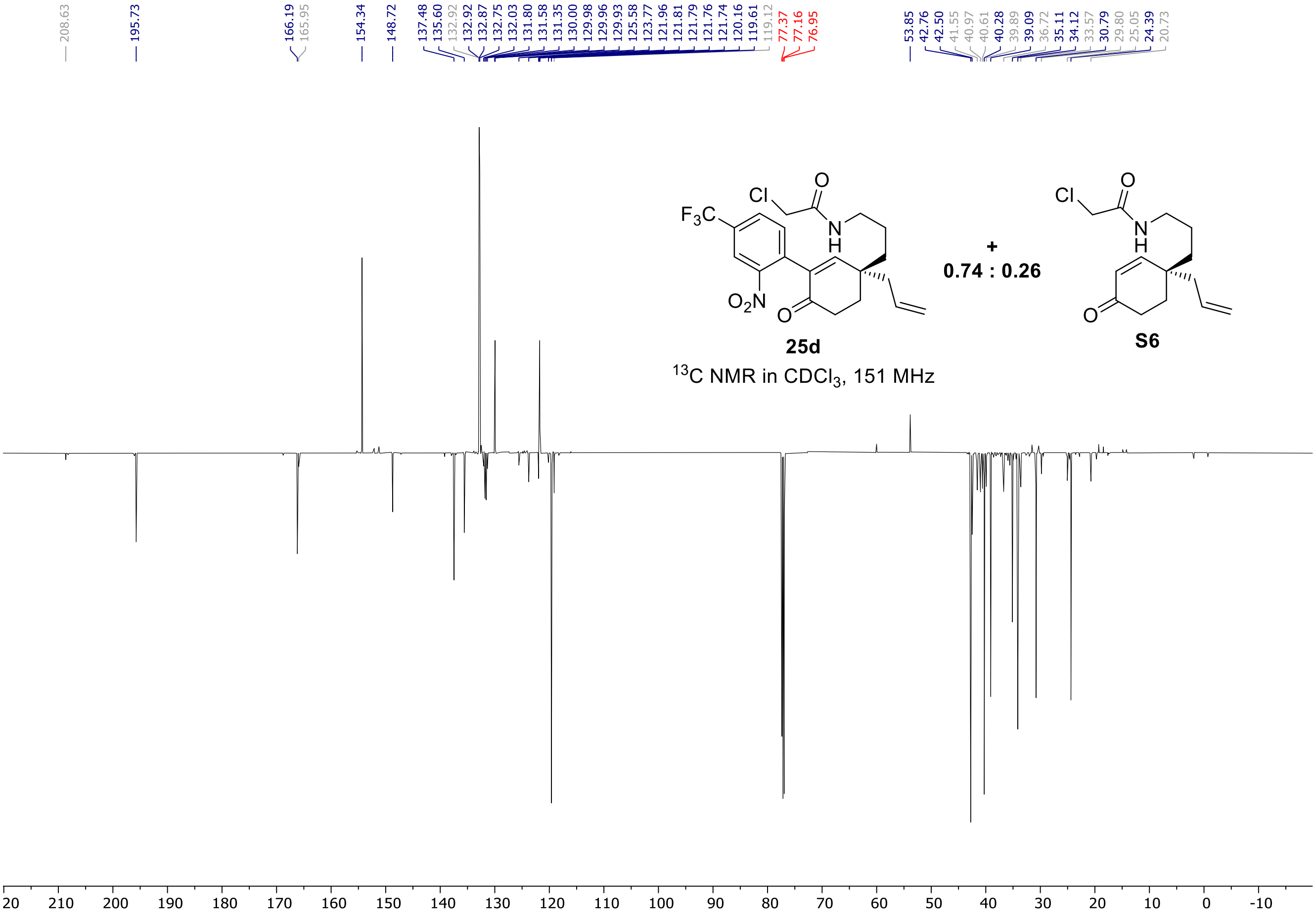
208.63 195.73 166.19 165.95 154.34 148.72 137.48 135.60 132.92 132.92 132.87 132.75 132.03 131.80 131.58 131.35 130.00 129.98 129.96 129.93 125.58 123.77 121.96 121.81 121.79 121.76 121.74 120.16 119.61 119.12 77.37 77.16 76.95 53.85 42.76 42.50 41.55 40.97 40.61 40.28 39.89 39.09 36.72 35.11 34.12 33.57 30.79 29.80 25.05 24.39 20.73
Cl
O
F3C
N
H
O2N
O
+
0.74 : 0.26
25d
S6
13C NMR in CDCl3, 151 MHz
20 210 200 190 180 170 160 150 140 130 120 110 100 90 80 70 60 50 40 30 20 10 0 -10

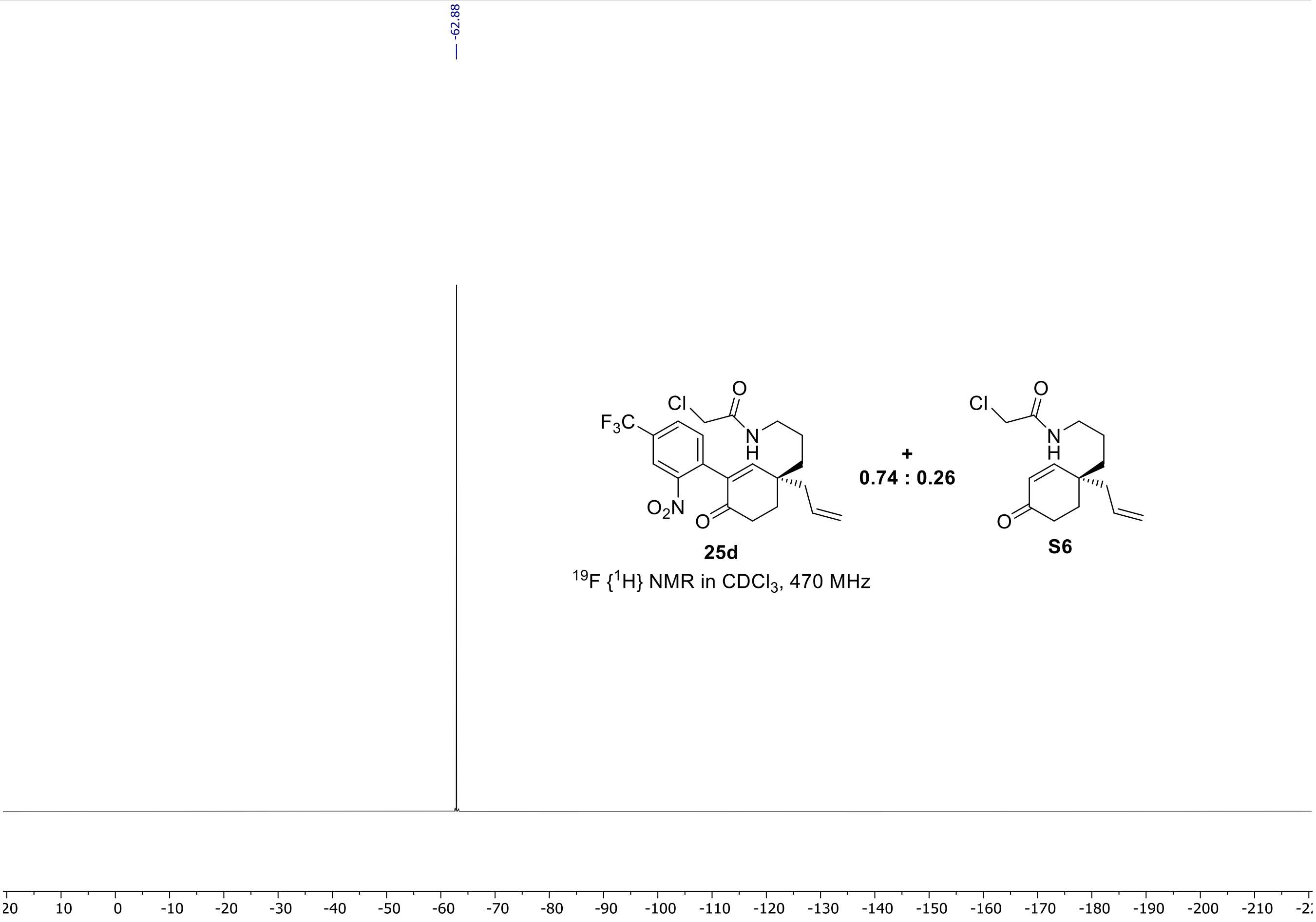
-62.88
F3C
Cl
O
N
H
O2N
O
25d
+
0.74 : 0.26
Cl
O
N
H
O
S6
19F {1H} NMR in CDCl3, 470 MHz
20
10
0
-10
-20
-30
-40
-50
-60
-70
-80
-90
-100
-110
-120
-130
-140
-150
-160
-170
-180
-190
-200
-210

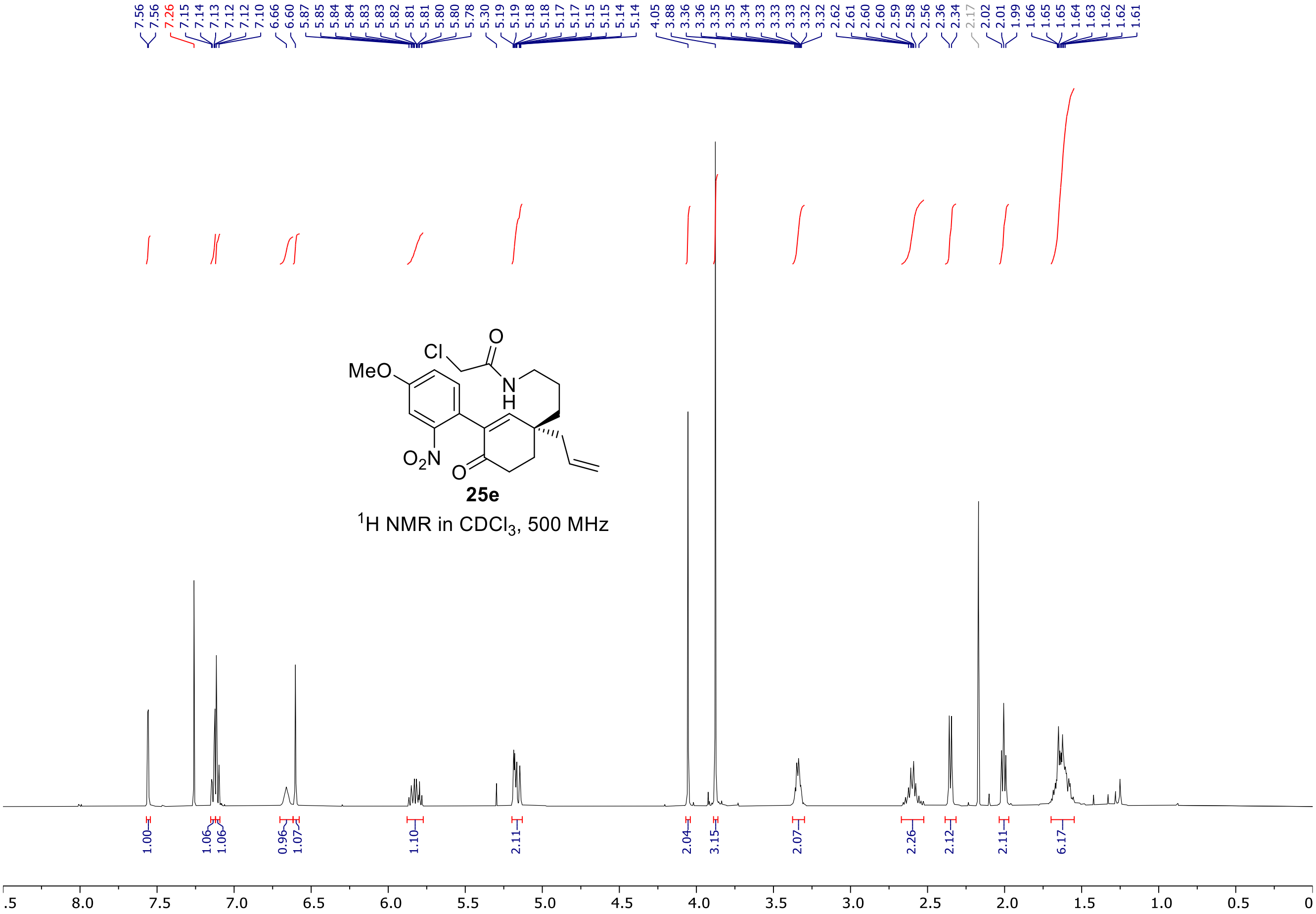
7.56 7.56 7.26 7.15 7.14 7.13 7.12 7.12 7.10 6.66 6.60 5.87 5.85 5.84 5.84 5.83 5.83 5.82 5.81 5.81 5.80 5.80 5.78 5.30 5.19 5.19 5.18 5.18 5.17 5.17 5.15 5.15 5.14 5.14 4.05 3.88 3.36 3.36 3.35 3.35 3.34 3.33 3.33 3.33 3.32 3.32 2.62 2.61 2.60 2.60 2.59 2.58 2.56 2.36 2.34 2.17 2.02 2.01 1.99 1.66 1.65 1.65 1.64 1.63 1.62 1.62 1.61
MeO
Cl
O
N
H
O_2N
O
25e
^1H NMR in $CDCl_3$, 500 MHz
1.00 1.06 1.06 0.96 1.07 1.10 2.11 2.04 3.15 2.07 2.26 2.12 2.11 6.17
.5 8.0 7.5 7.0 6.5 6.0 5.5 5.0 4.5 4.0 3.5 3.0 2.5 2.0 1.5 1.0 0.5 0

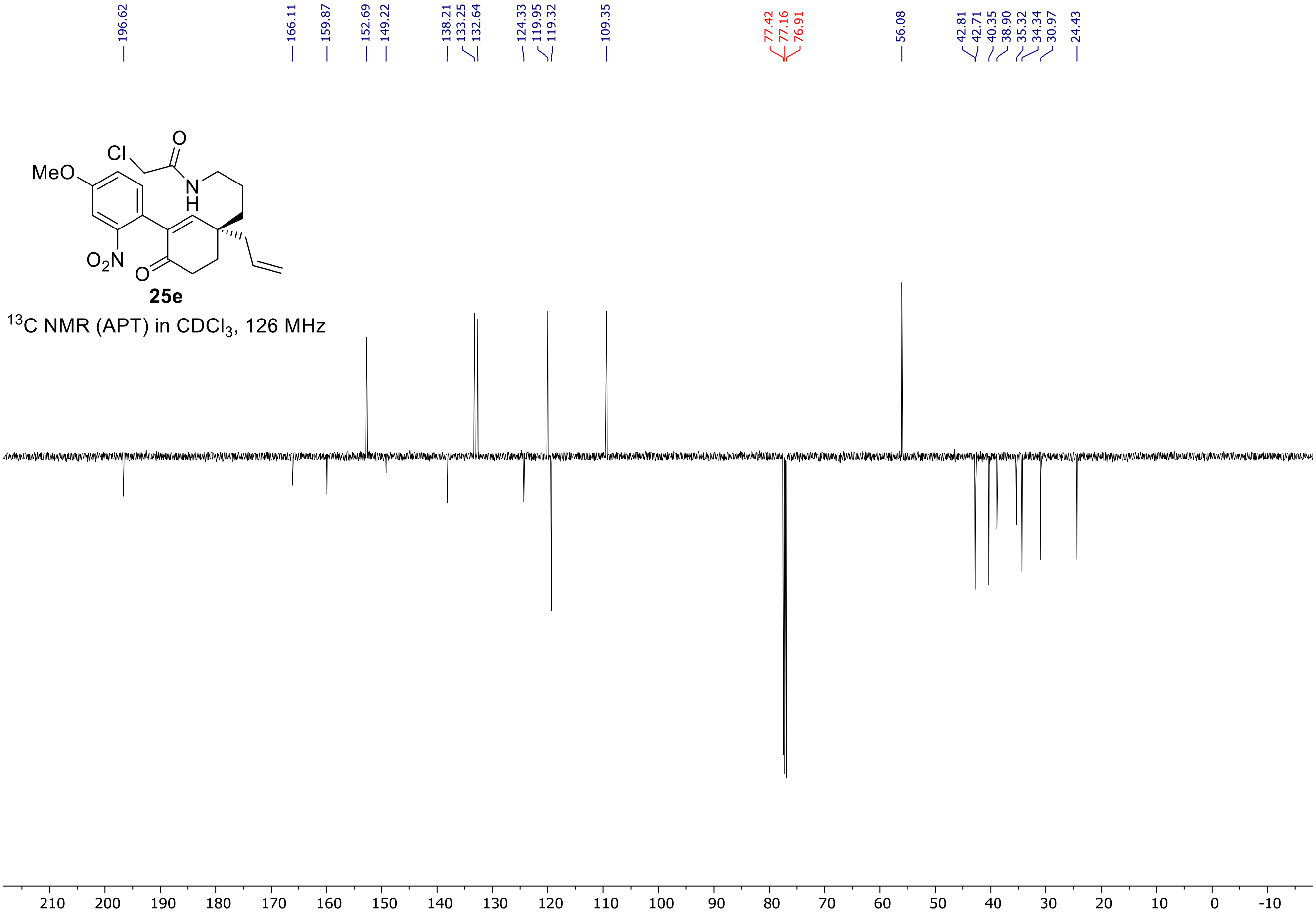
196.62 166.11 159.87 152.69 149.22 138.21 133.25 132.64 124.33 119.95 119.32 109.35 77.42 77.16 76.91 56.08 42.81 42.71 40.35 38.90 35.32 34.34 30.97 24.43
MeO
Cl
O
N
H
O_2N
O
25e
^{13}C NMR (APT) in $CDCl_3$, 126 MHz
210 200 190 180 170 160 150 140 130 120 110 100 90 80 70 60 50 40 30 20 10 0 -10

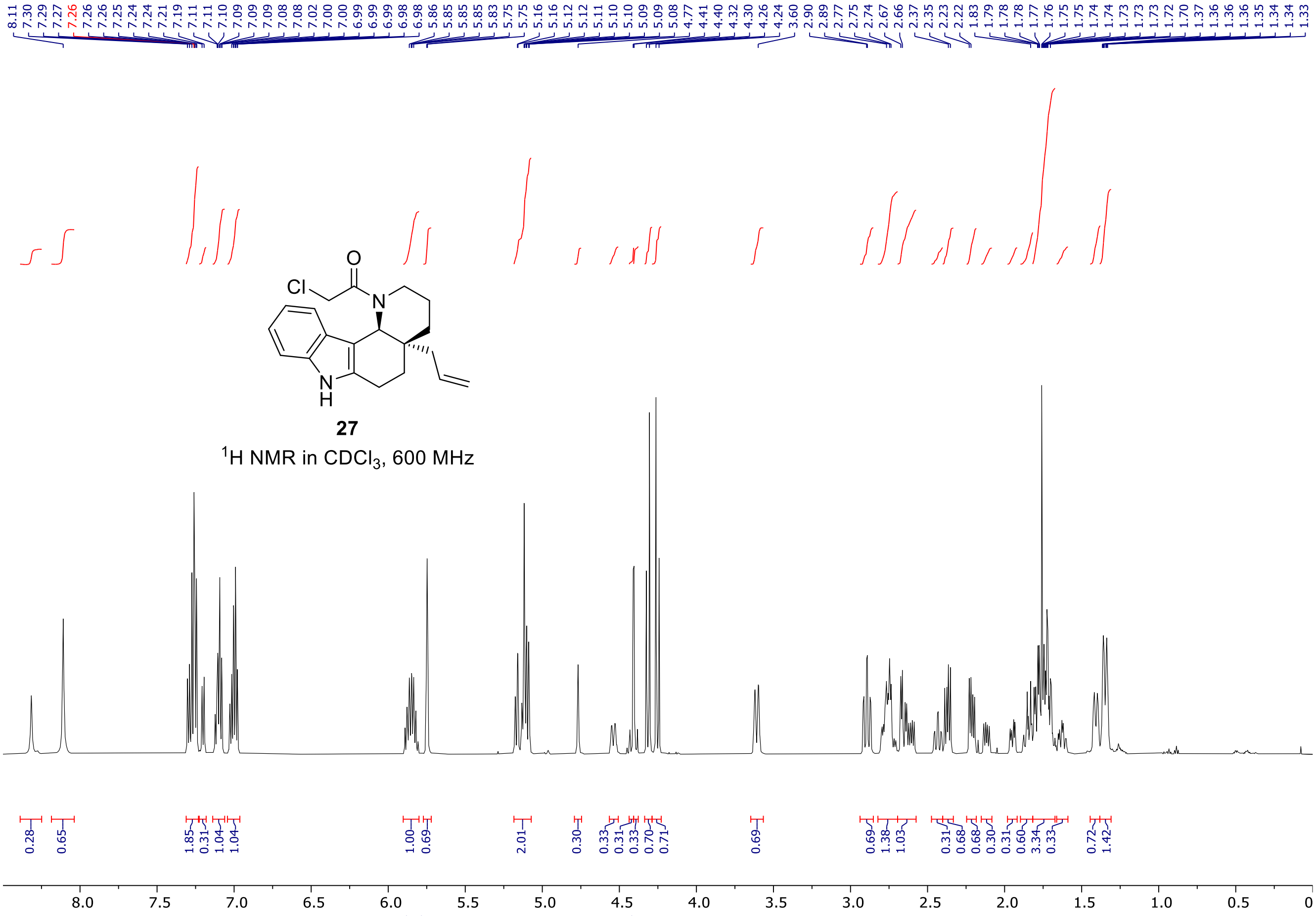

27

^{1}H NMR in $CDCl_3$, 600 MHz

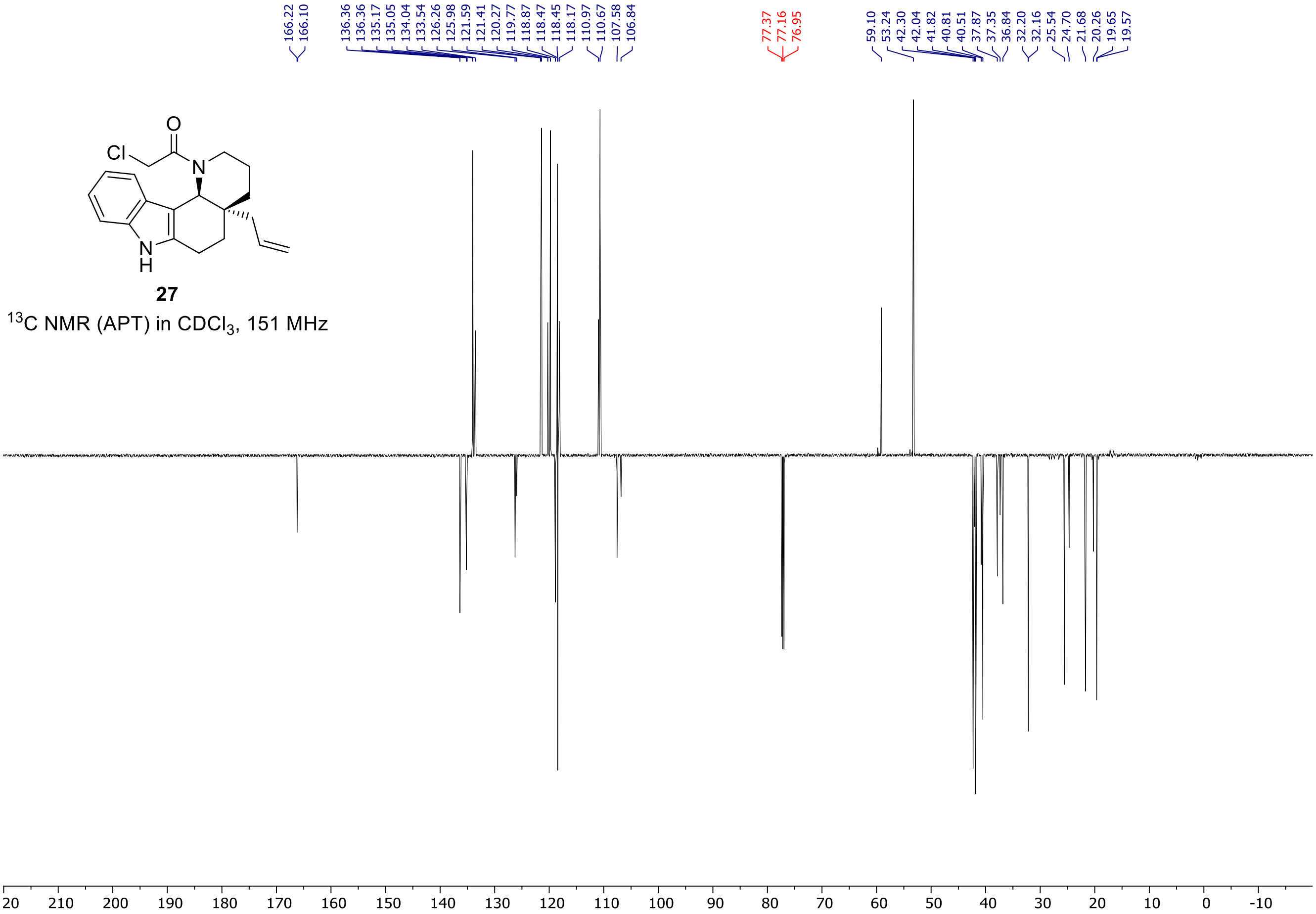

27

^{13}C NMR (APT) in $CDCl_3$, 151 MHz

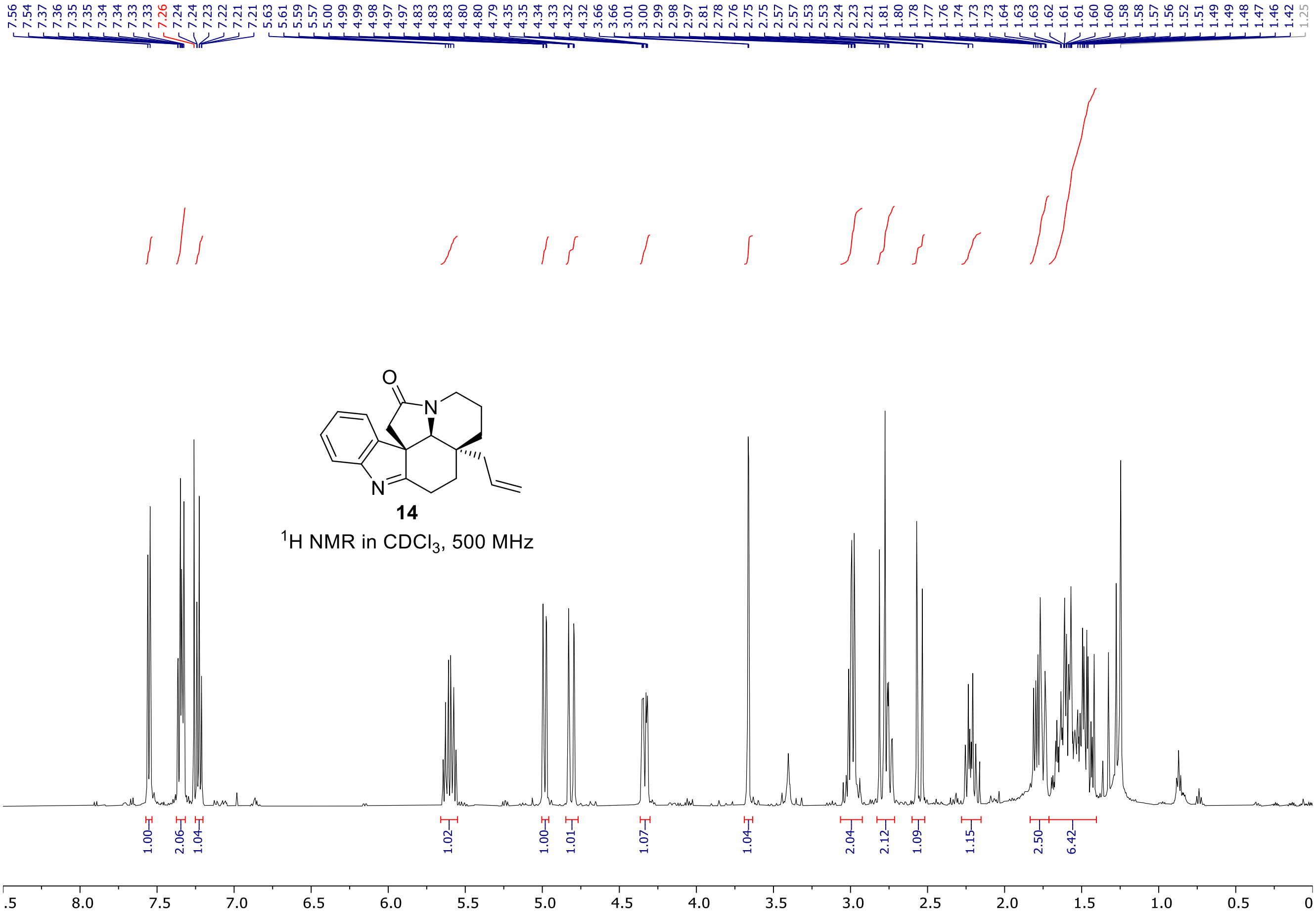
14
^{1}H NMR in $CDCl_3$, 500 MHz

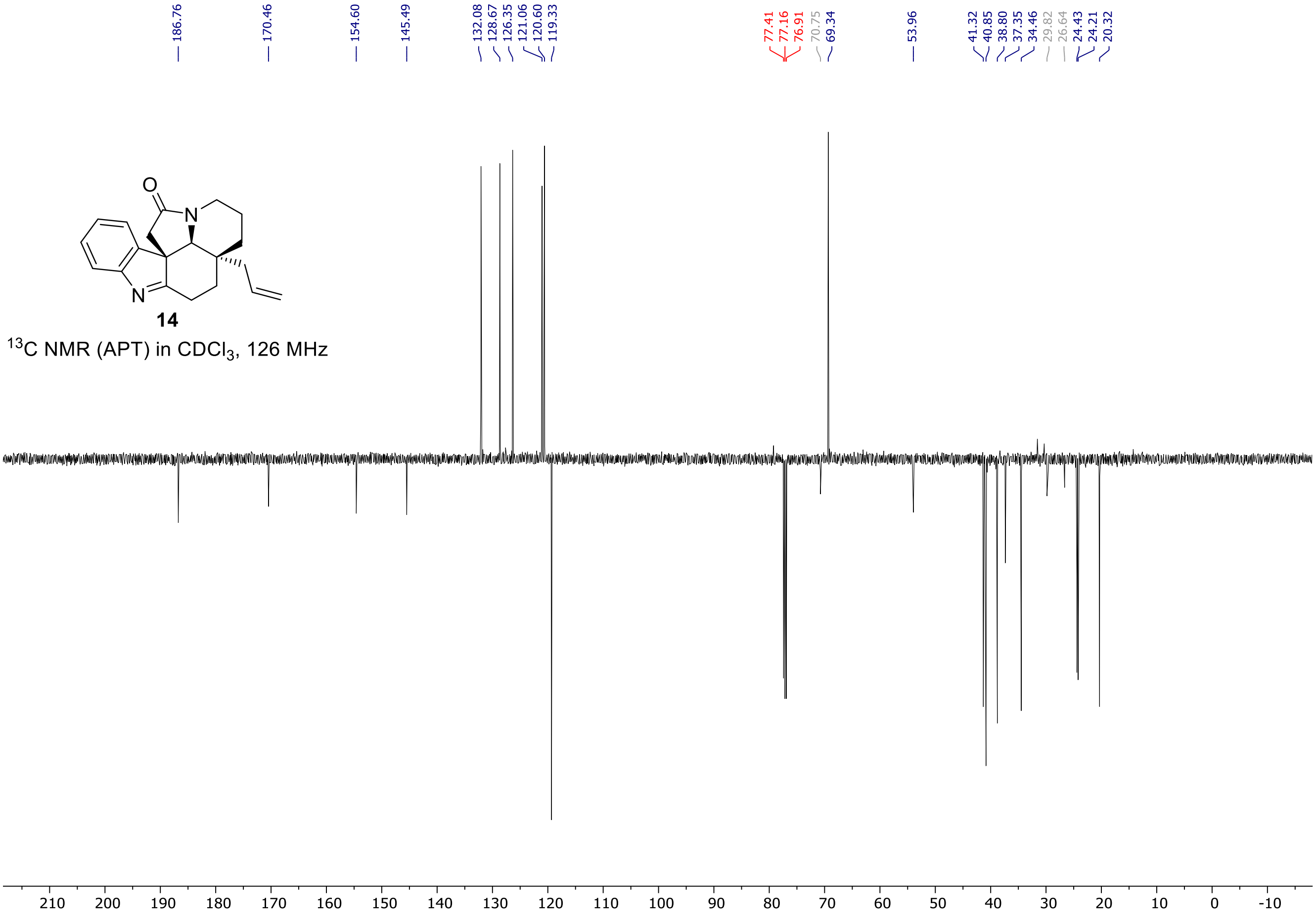
14
^{13}C NMR (APT) in $CDCl_3$, 126 MHz

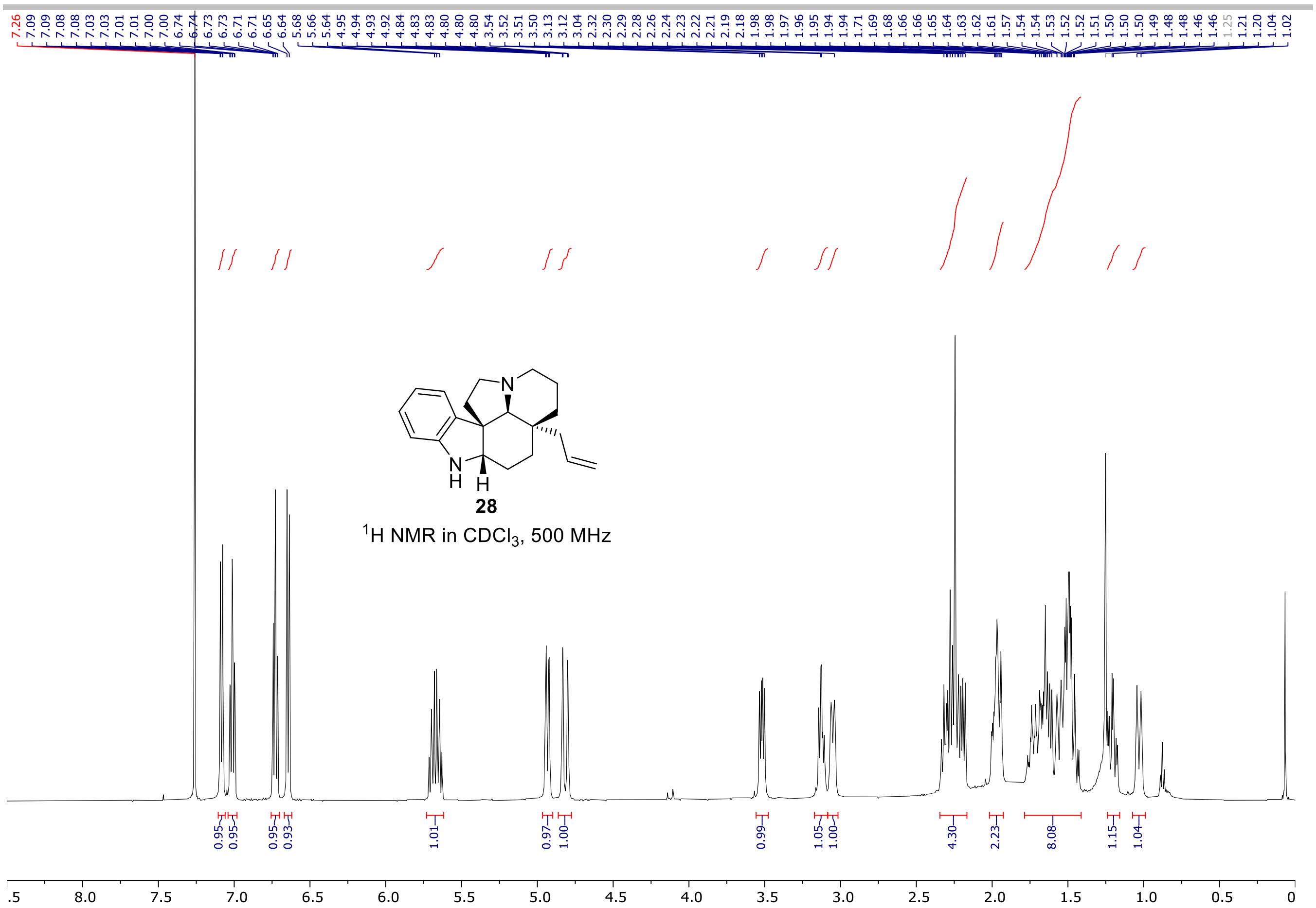
7.26
7.09
7.09
7.08
7.08
7.03
7.03
7.01
7.01
7.00
7.00
6.74
6.74
6.73
6.73
6.71
6.71
6.65
6.64
5.68
5.66
5.64
4.95
4.94
4.93
4.92
4.84
4.83
4.83
4.80
4.80
4.80
3.54
3.52
3.51
3.50
3.13
3.12
3.04
2.32
2.30
2.29
2.28
2.26
2.24
2.23
2.22
2.21
2.19
2.18
1.98
1.98
1.97
1.96
1.95
1.94
1.94
1.71
1.69
1.68
1.66
1.66
1.65
1.64
1.63
1.62
1.61
1.57
1.54
1.54
1.53
1.52
1.52
1.51
1.50
1.50
1.50
1.49
1.48
1.48
1.46
1.25
1.21
1.20
1.04
1.02
N
H
H
28
^{1}H NMR in $CDCl_3$, 500 MHz
0.95
0.95
0.95
0.93
1.01
0.97
1.00
0.99
1.05
1.00
4.30
2.23
8.08
1.15
1.04

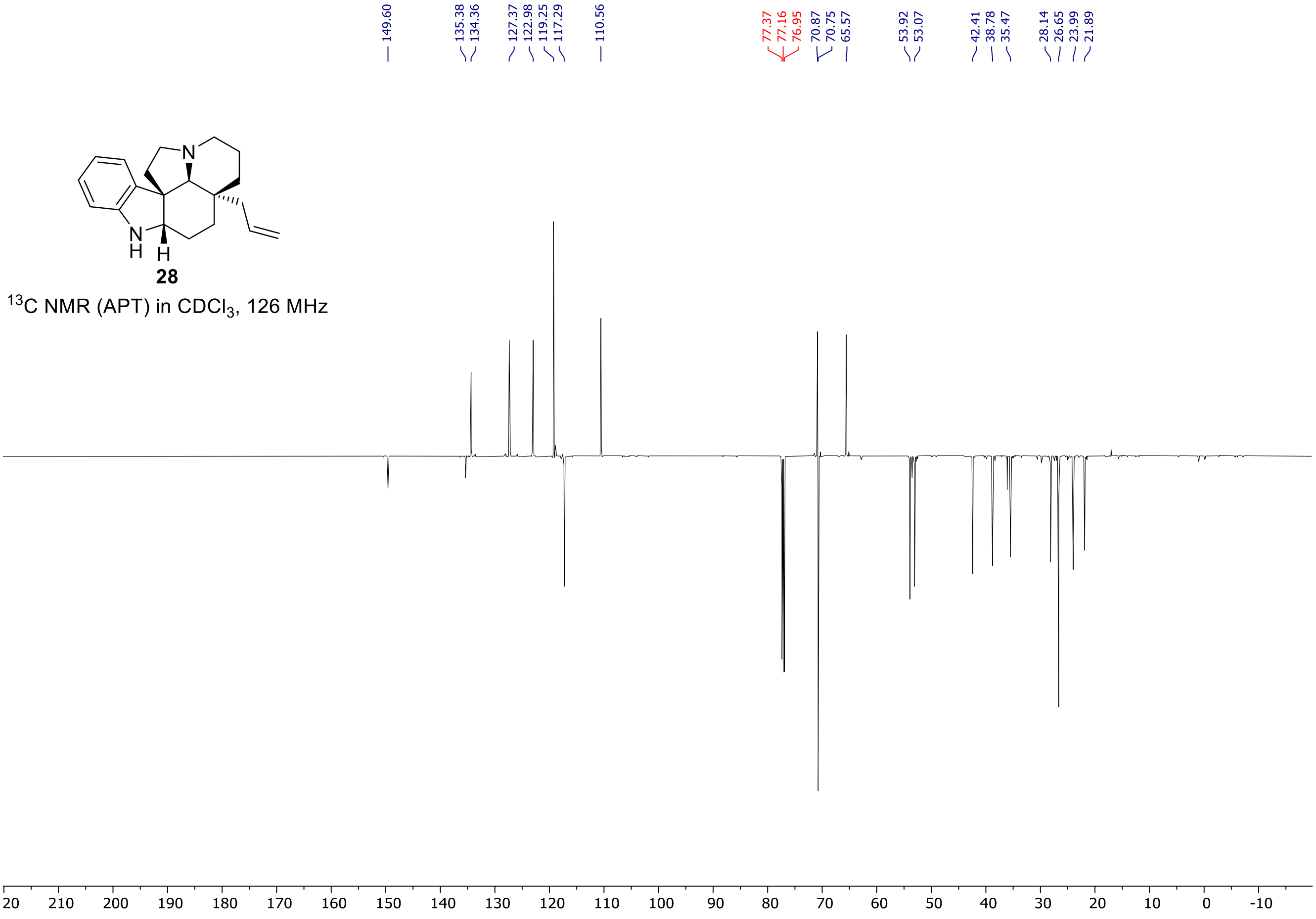
149.60
135.38
134.36
127.37
122.98
119.25
117.29
110.56
77.37
77.16
76.95
70.87
70.75
65.57
53.92
53.07
42.41
38.78
35.47
28.14
26.65
23.99
21.89
N
H
H
28
^{13}C NMR (APT) in $CDCl_3$, 126 MHz

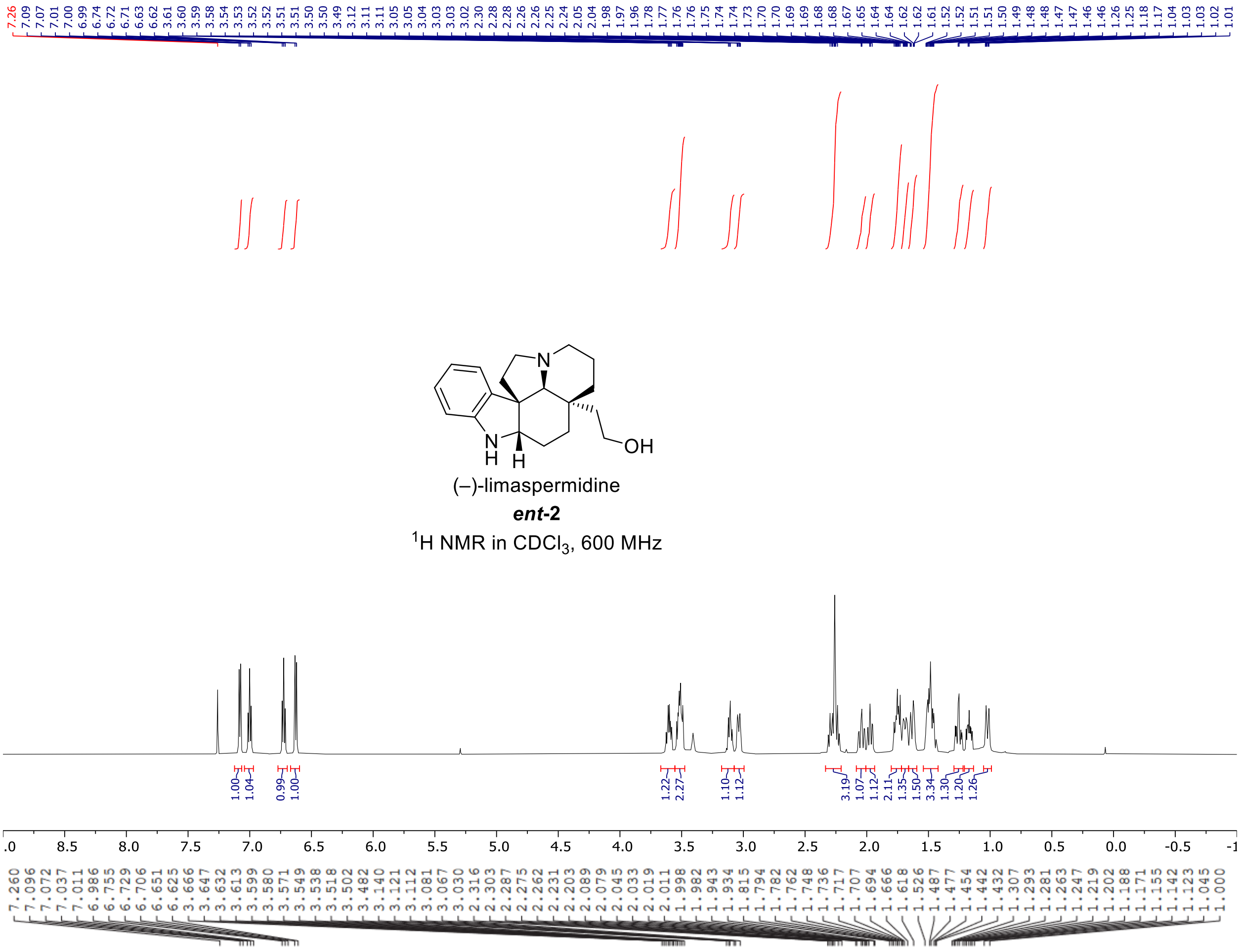

(–)-limaspermidine

***ent*-2**

^{1}H NMR in $CDCl_3$, 600 MHz

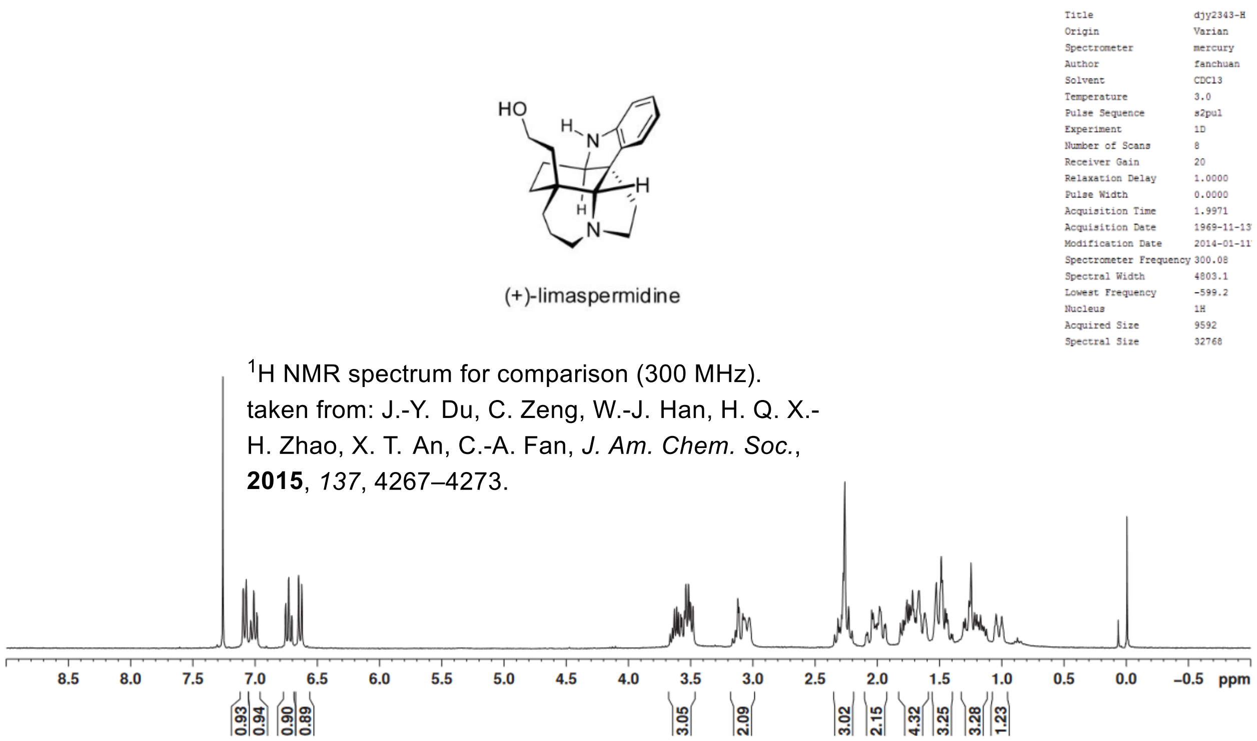

(+)-limaspermidine

^{1}H NMR spectrum for comparison (300 MHz). taken from: J.-Y. Du, C. Zeng, W.-J. Han, H. Q. X.-H. Zhao, X. T. An, C.-A. Fan, *J. Am. Chem. Soc.*, **2015**, *137*, 4267–4273.

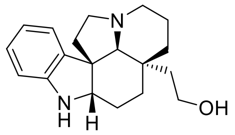

(–)-limaspermidine
***ent*-2**
^{13}C NMR (APT) in $CDCl_3$, 151 MHz

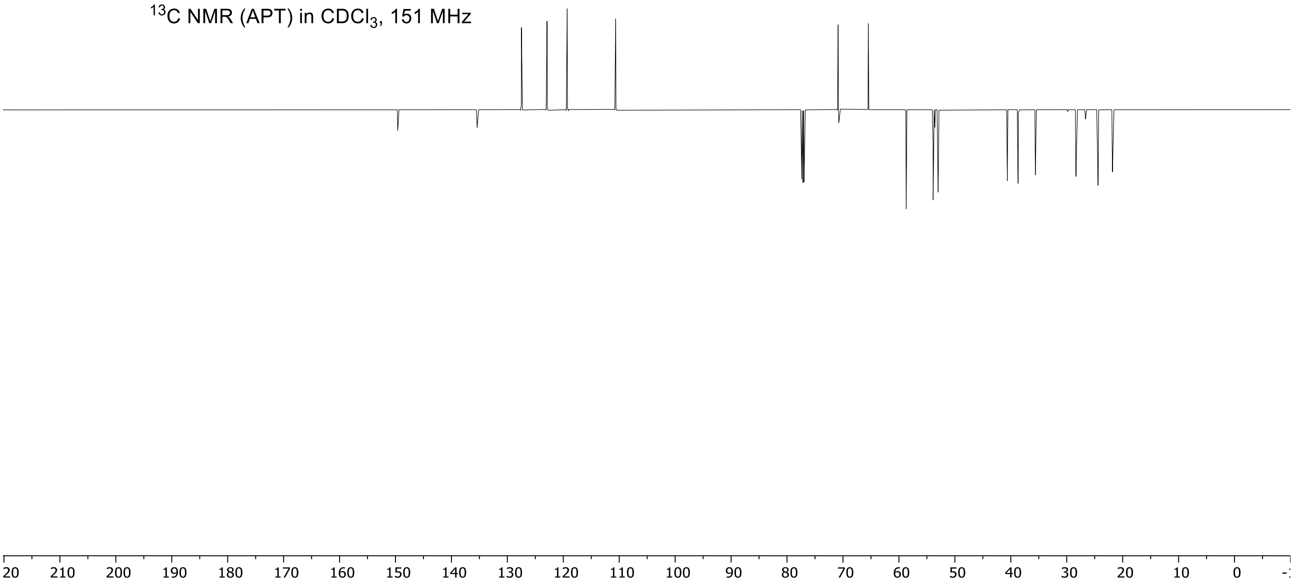

Title	djy2343-C(600MHz)
Origin	Varian
Spectrometer	inova
Author	liusongq
Solvent	CDCl3
Temperature	22.0
Pulse Sequence	s2pul
Experiment	1D
Number of Scans	849
Receiver Gain	60
Relaxation Delay	1.0000
Pulse Width	0.0000
Acquisition Time	1.3005
Acquisition Date	2014-01-13T15:07:25
Modification Date	2014-01-13T16:06:48
Spectrometer Frequency	150.85
Spectral Width	41797.3
Lowest Frequency	-3174.1
Nucleus	13C
Acquired Size	54356
Spectral Size	131072

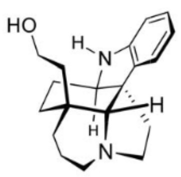

(+)-limaspermidine

^{13}C NMR spectrum for comparison (151 MHz). taken from: J.-Y. Du, C. Zeng, W.-J. Han, H. Q. X.-H. Zhao, X. T. An, C.-A. Fan, *J. Am. Chem. Soc.*, **2015**, *137*, 4267–4273.

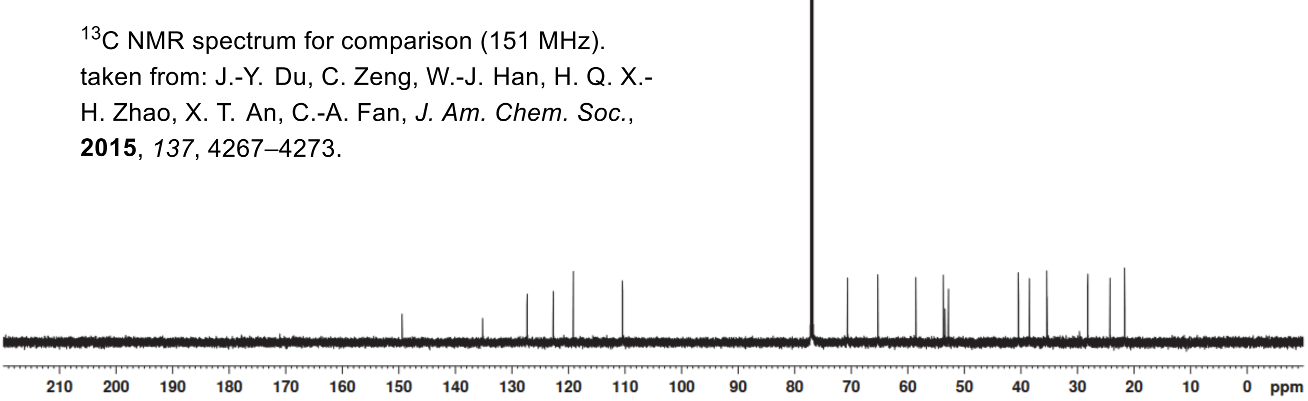

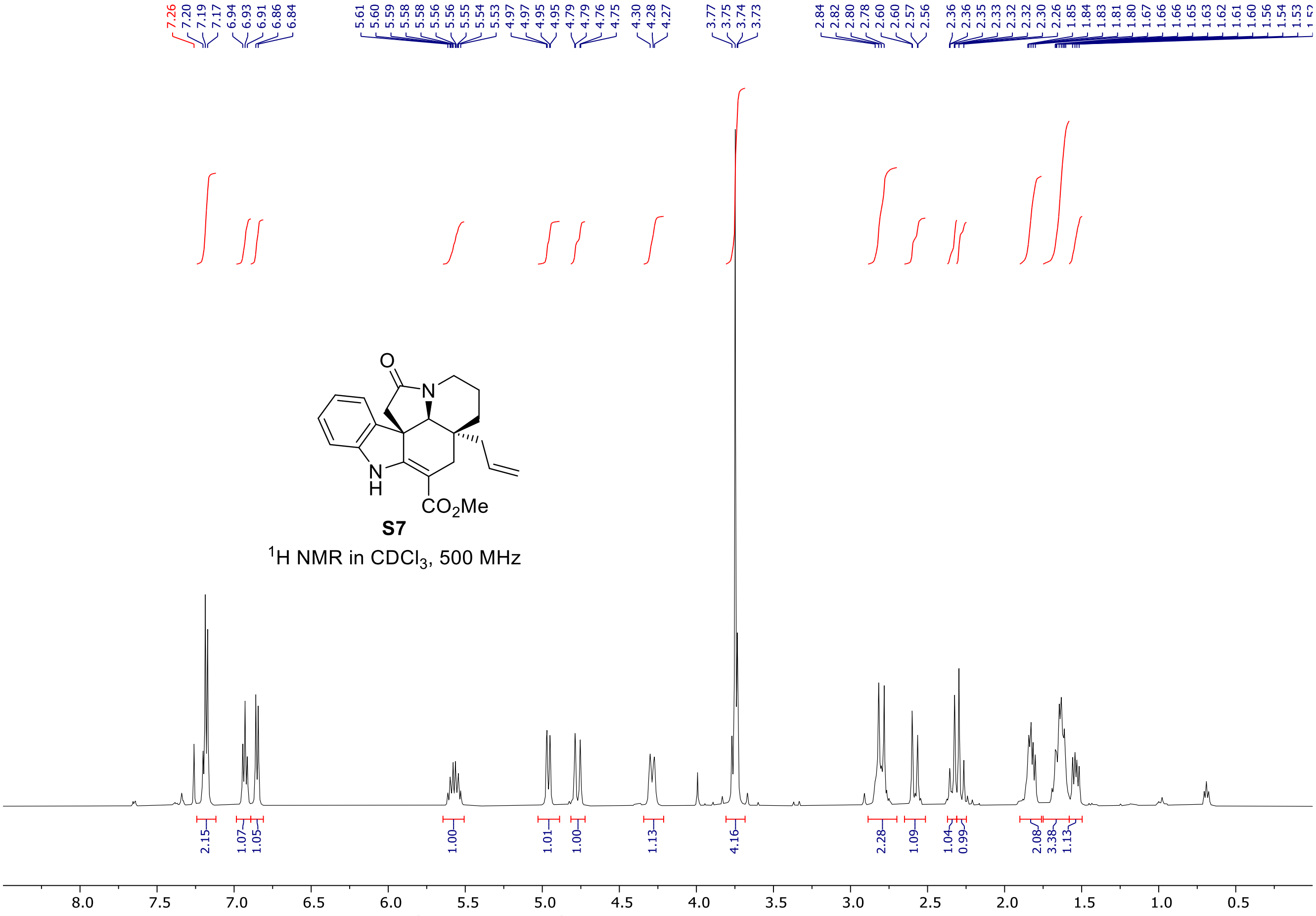
O
N
N
H
CO2Me
S7
1H NMR in CDCl3, 500 MHz

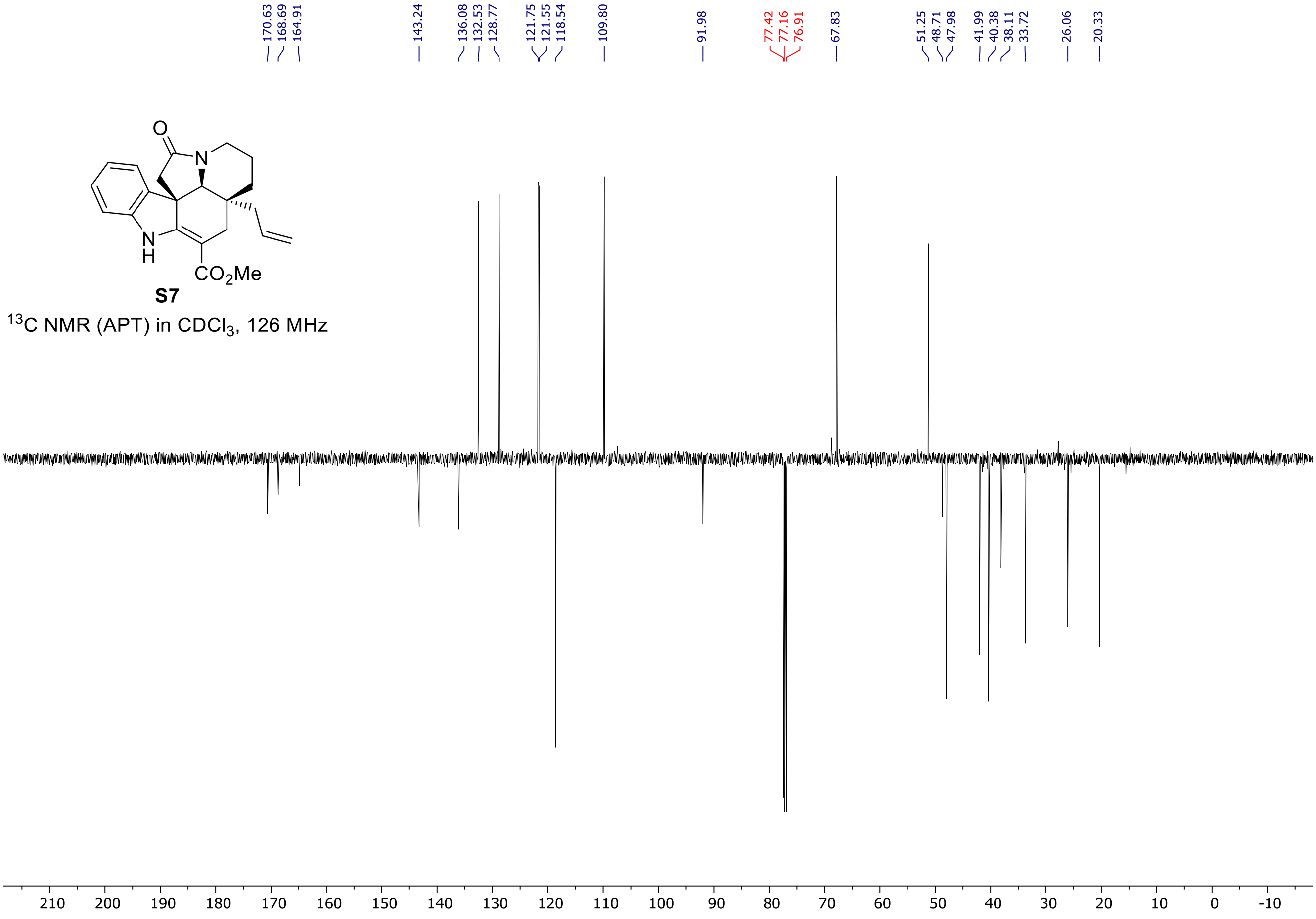
O
N
N
H
CO2Me
S7
13C NMR (APT) in CDCl3, 126 MHz

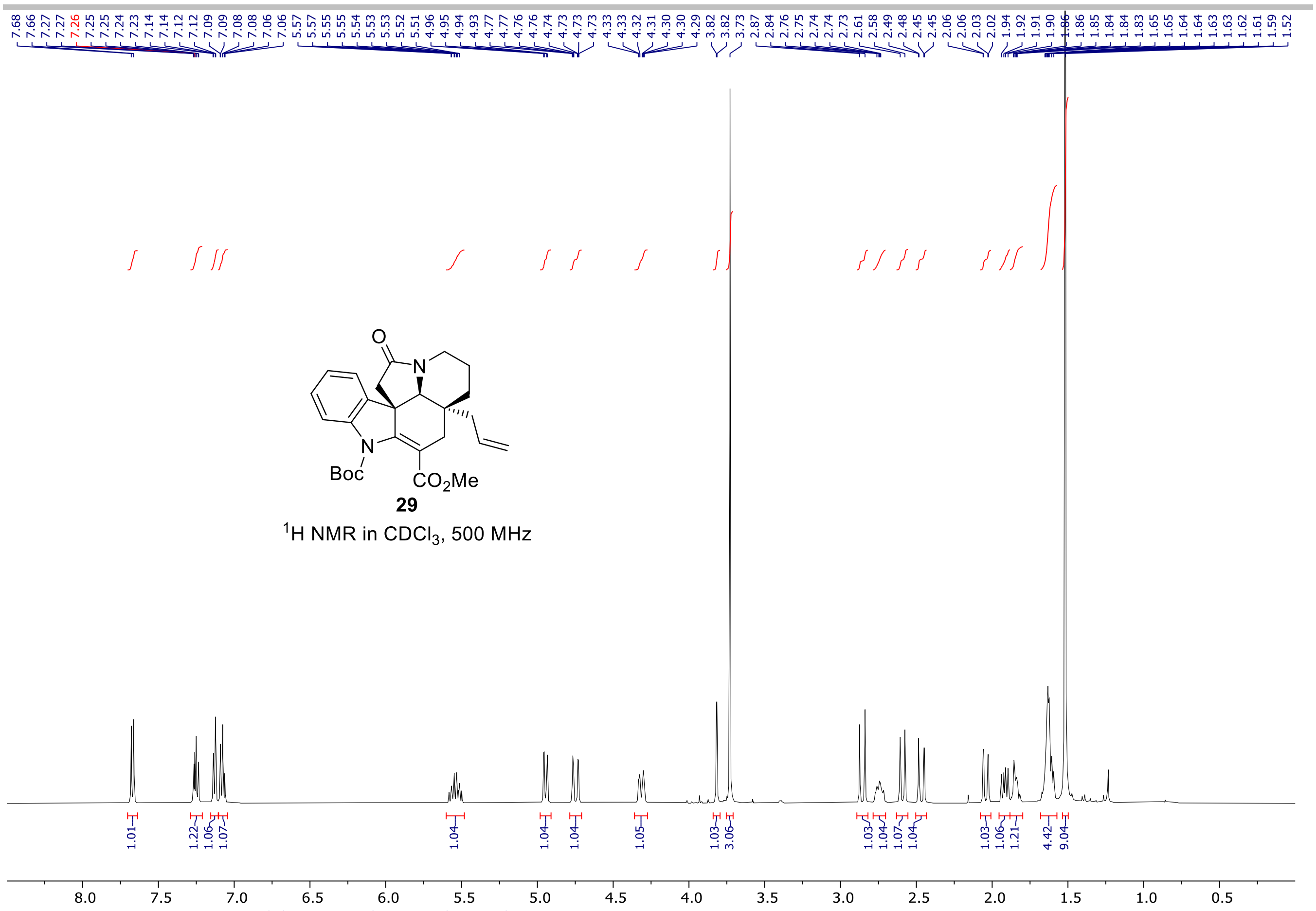

29

^{1}H NMR in $CDCl_3$, 500 MHz

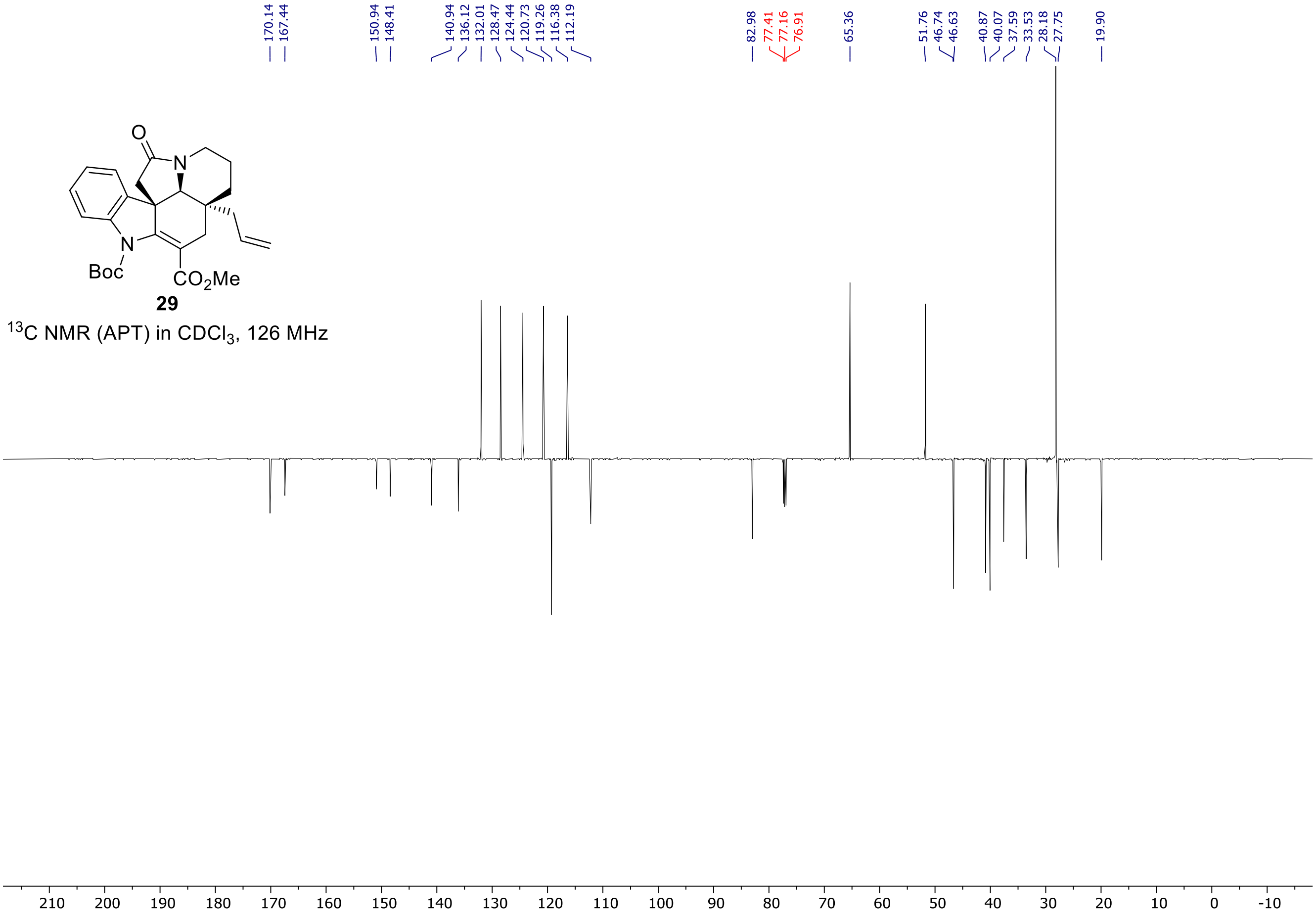

29

^{13}C NMR (APT) in $CDCl_3$, 126 MHz

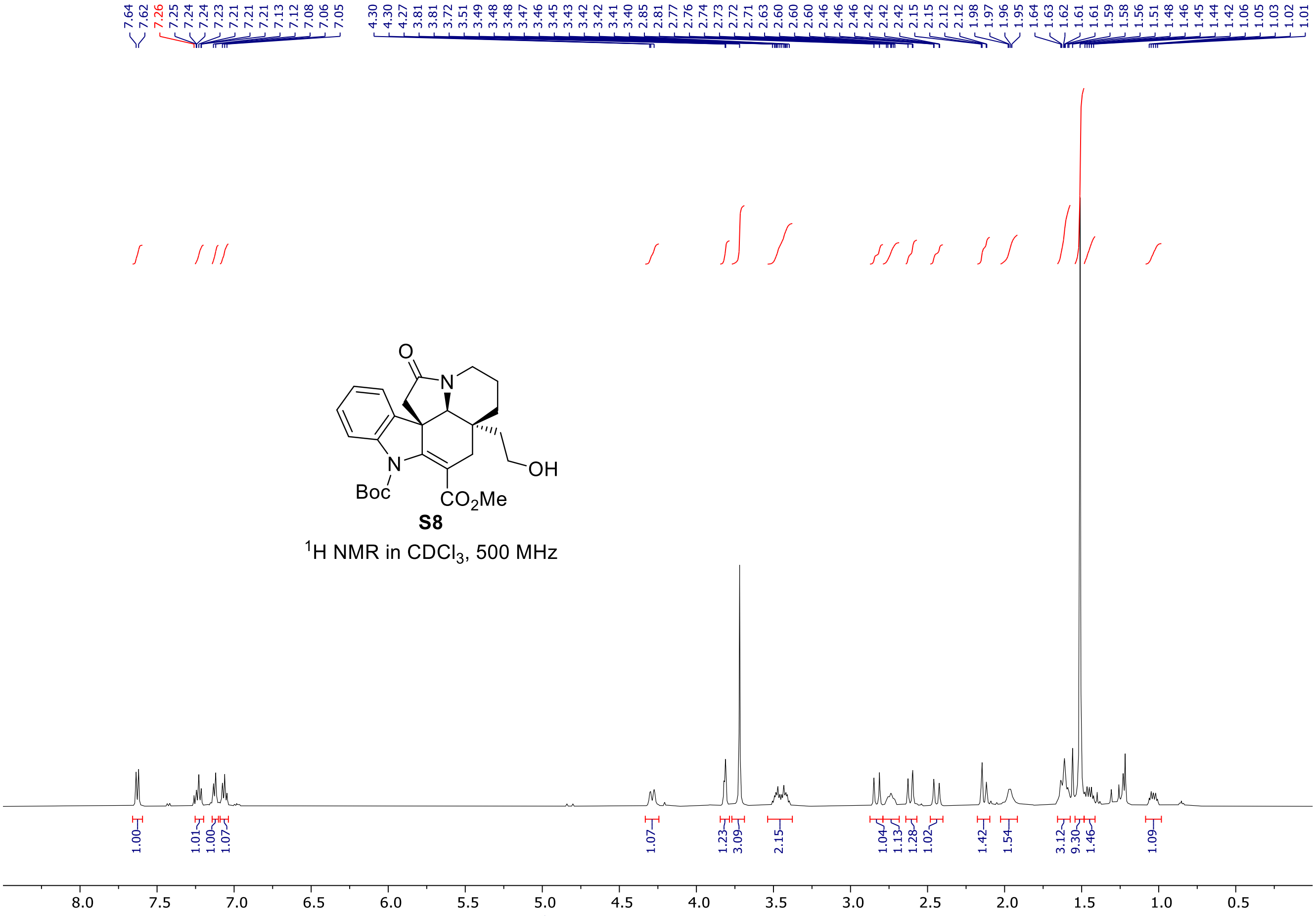
7.64
7.62
7.26
7.25
7.24
7.24
7.23
7.21
7.21
7.21
7.13
7.12
7.08
7.06
7.05
4.30
4.30
4.27
3.81
3.81
3.72
3.51
3.49
3.48
3.48
3.47
3.46
3.45
3.43
3.42
3.42
3.41
3.40
2.85
2.81
2.77
2.76
2.74
2.73
2.72
2.72
2.71
2.63
2.60
2.60
2.60
2.46
2.46
2.42
2.42
2.42
2.15
2.15
2.12
2.12
1.98
1.97
1.96
1.95
1.64
1.63
1.62
1.61
1.61
1.59
1.58
1.56
1.51
1.48
1.46
1.45
1.44
1.42
1.06
1.05
1.03
1.02
1.01
O
N
N
Boc
OH
CO2Me
S8
1H NMR in CDCl3, 500 MHz
1.00
1.01
1.00
1.07
1.07
1.23
3.09
2.15
1.04
1.13
1.28
1.02
1.42
1.54
3.12
9.30
1.46
1.09
8.0
7.5
7.0
6.5
6.0
5.5
5.0
4.5
4.0
3.5
3.0
2.5
2.0
1.5
1.0
0.5

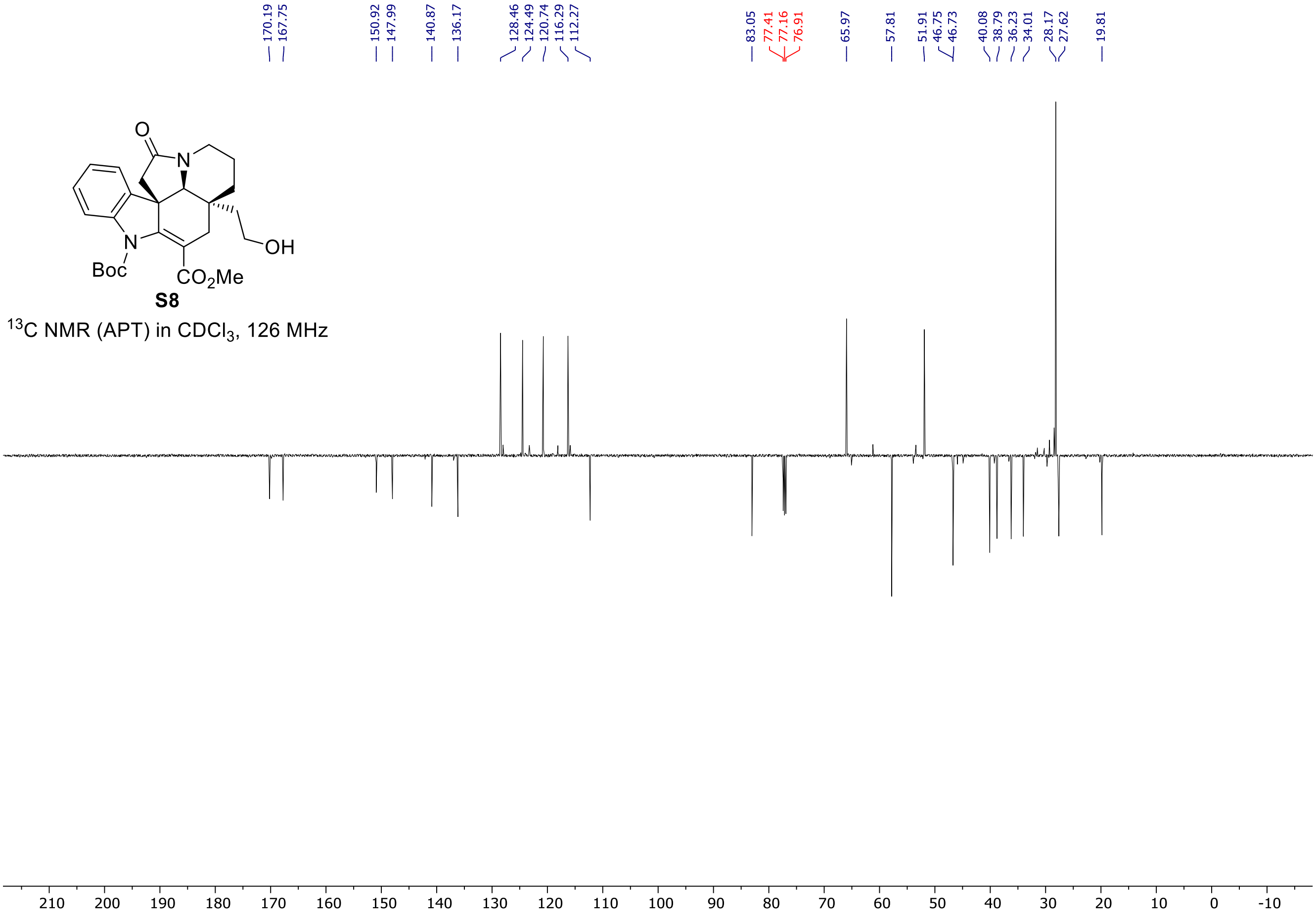
170.19
167.75
150.92
147.99
140.87
136.17
128.46
124.49
120.74
116.29
112.27
83.05
77.41
77.16
76.91
65.97
57.81
51.91
46.75
46.73
40.08
38.79
36.23
34.01
28.17
27.62
19.81
O
N
N
Boc
OH
CO2Me
S8
13C NMR (APT) in CDCl3, 126 MHz
210
200
190
180
170
160
150
140
130
120
110
100
90
80
70
60
50
40
30
20
10
0
-10

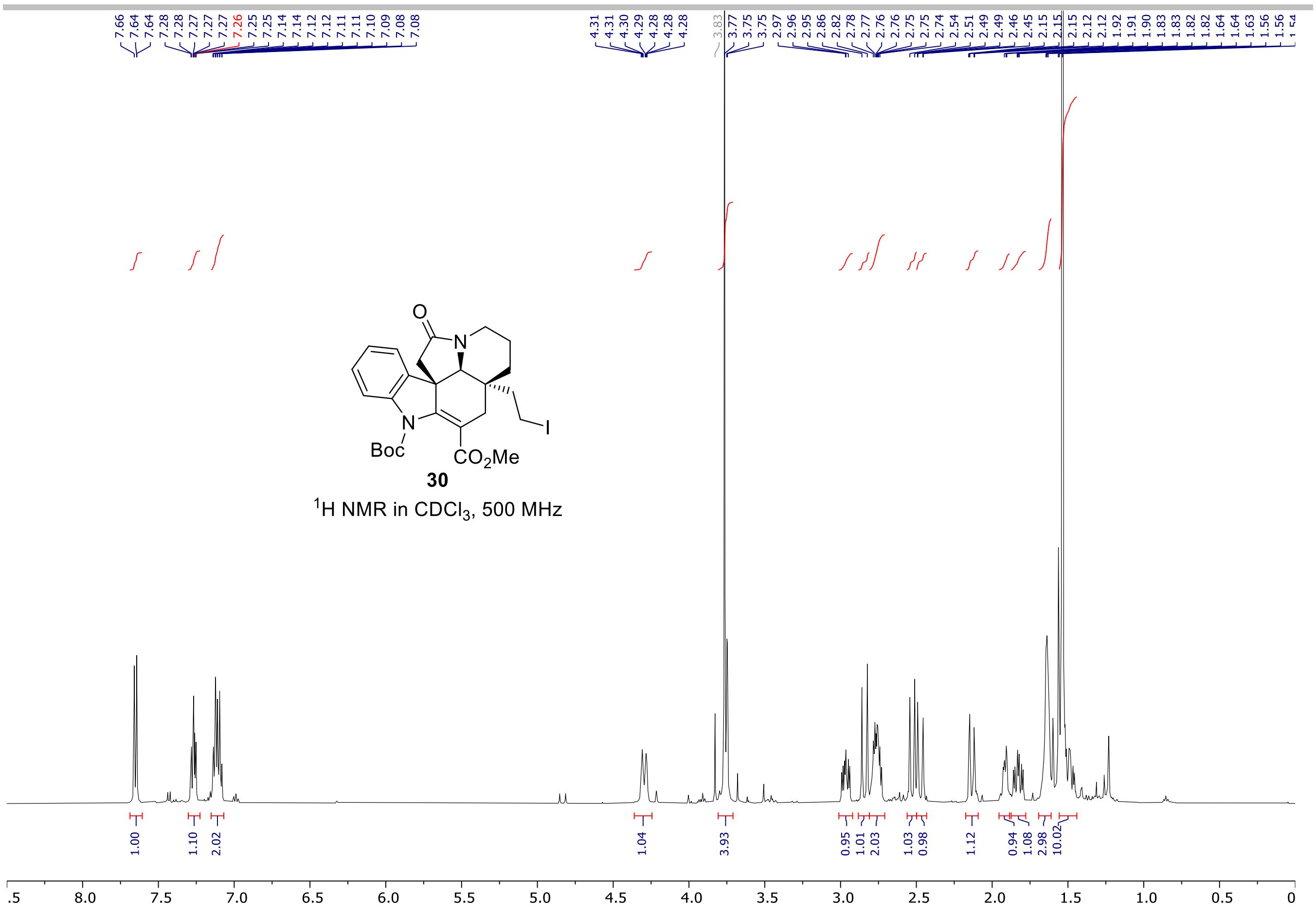
O
N
N
Boc
CO$_2$Me
I
30
^{1}H NMR in CDCl$_3$, 500 MHz

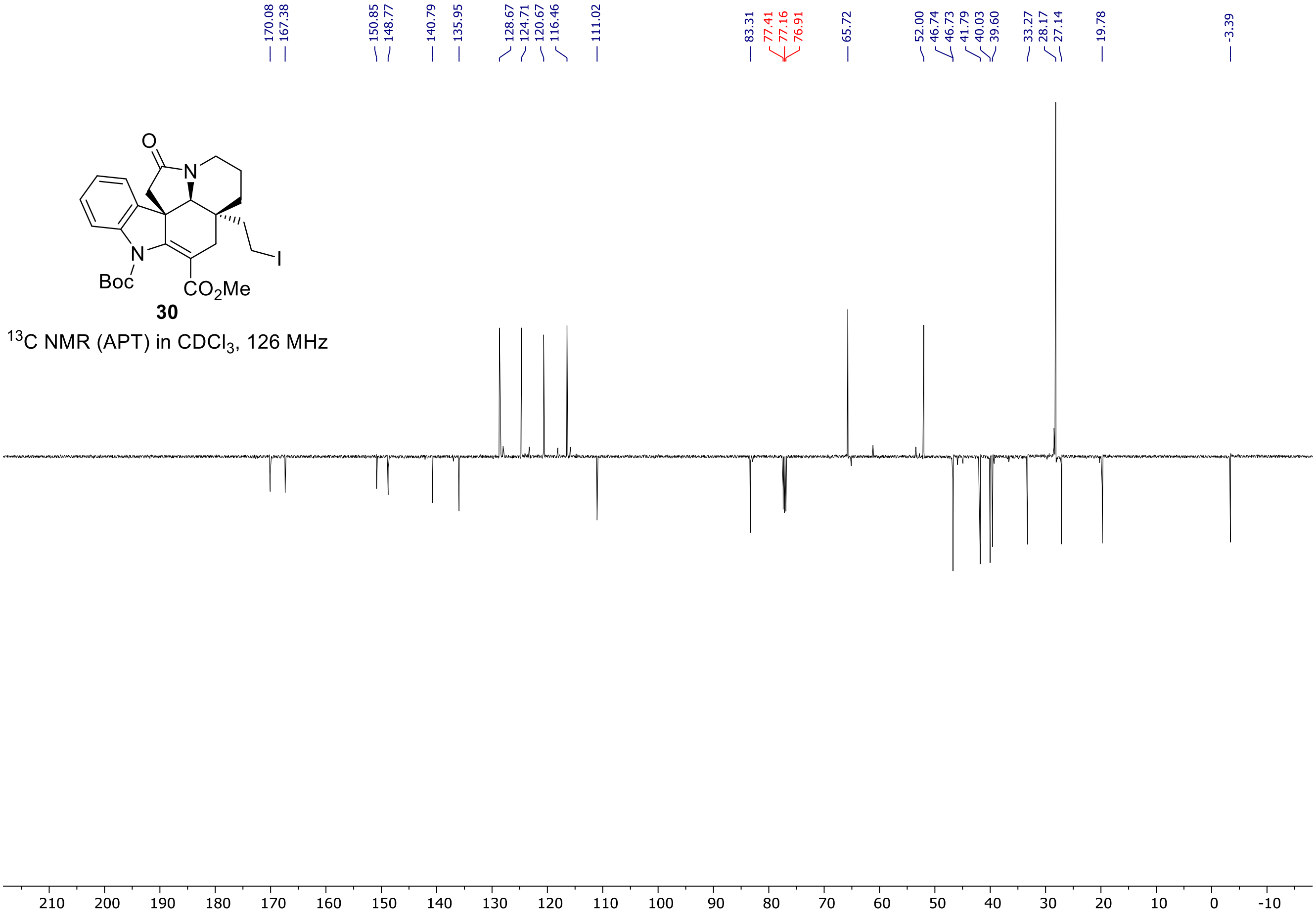
O
N
N
Boc
CO$_2$Me
I
30
^{13}C NMR (APT) in CDCl$_3$, 126 MHz

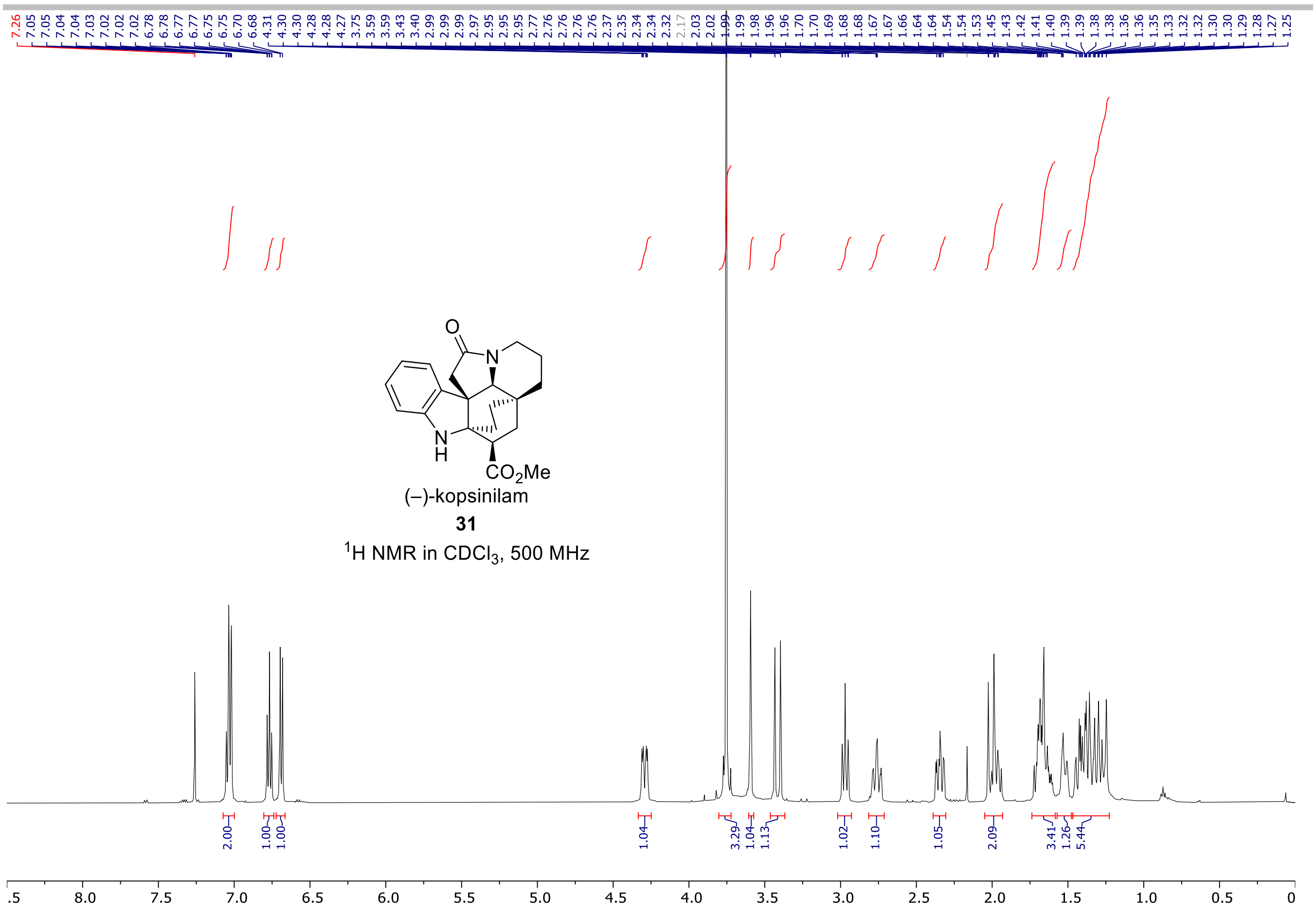
O
N
NH
CO2Me
(–)-kopsinilam
31
1H NMR in CDCl3, 500 MHz

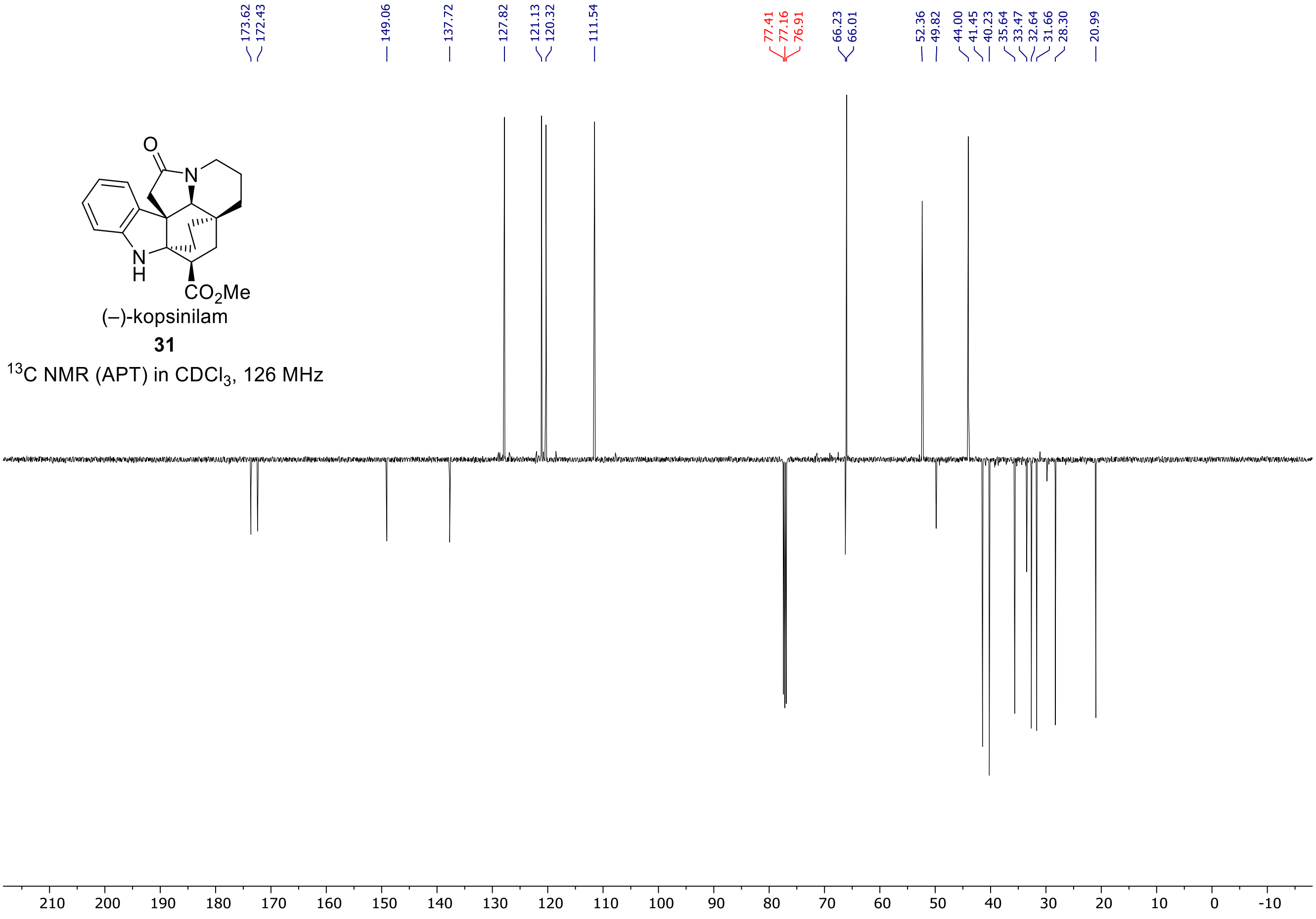
173.62
172.43
149.06
137.72
127.82
121.13
120.32
111.54
77.41
77.16
76.91
66.23
66.01
52.36
49.82
44.00
41.45
40.23
35.64
33.47
32.64
31.66
28.30
20.99
O
N
NH
CO2Me
(–)-kopsinilam
31
13C NMR (APT) in CDCl3, 126 MHz

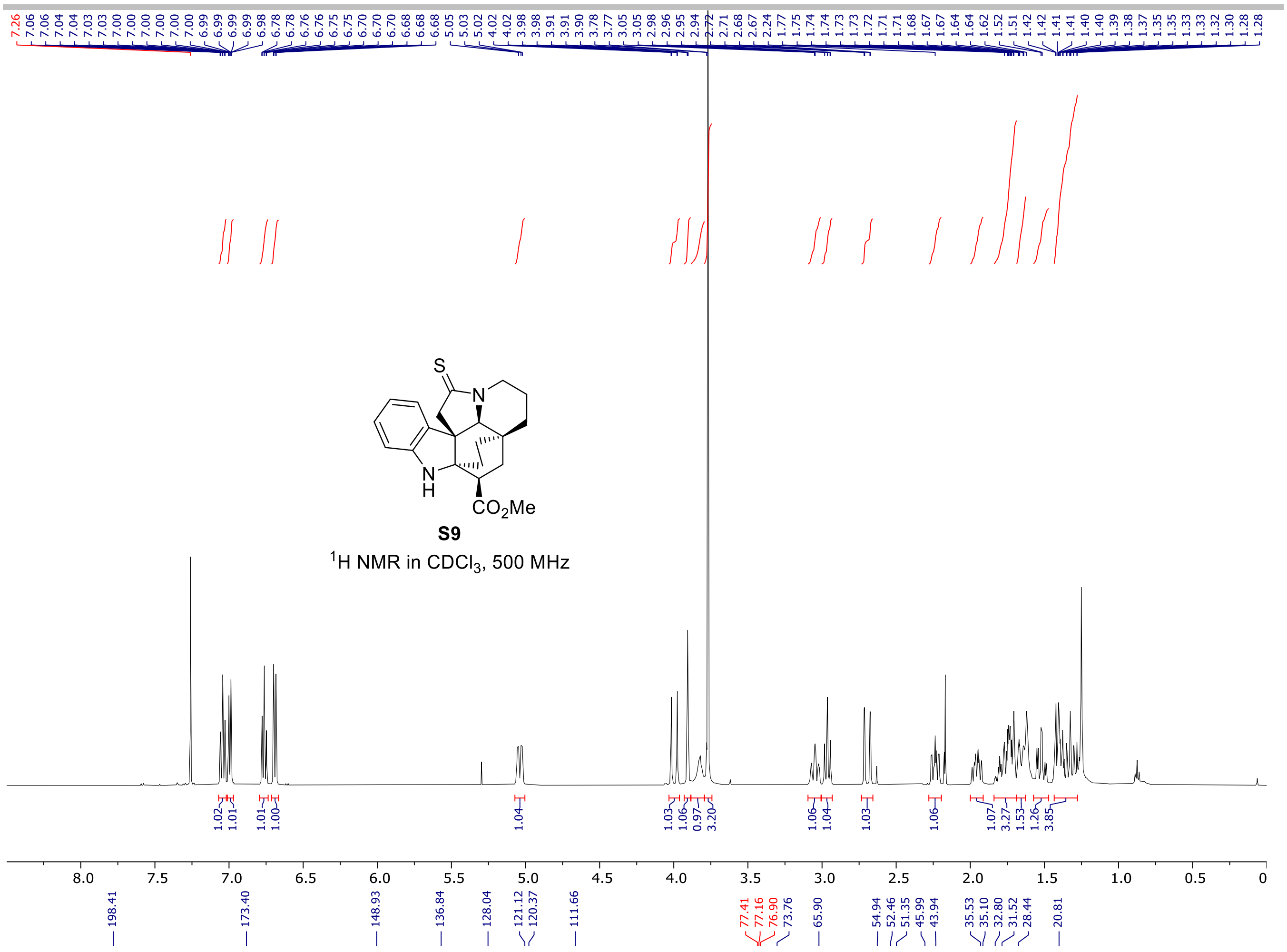
S
N
NH
CO2Me
S9
1H NMR in CDCl3, 500 MHz
8.0 7.5 7.0 6.5 6.0 5.5 5.0 4.5 4.0 3.5 3.0 2.5 2.0 1.5 1.0 0.5 0

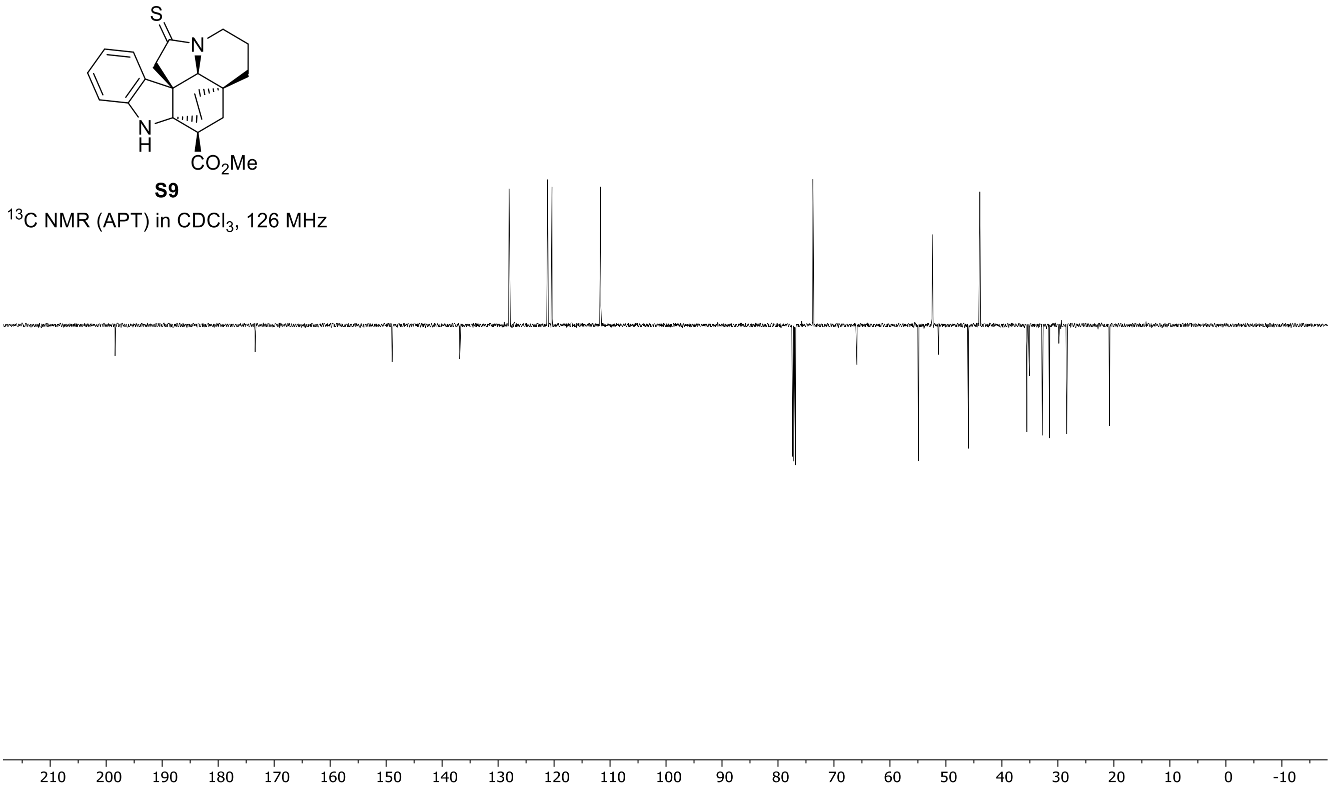
S
N
NH
CO2Me
S9
13C NMR (APT) in CDCl3, 126 MHz
210 200 190 180 170 160 150 140 130 120 110 100 90 80 70 60 50 40 30 20 10 0 -10

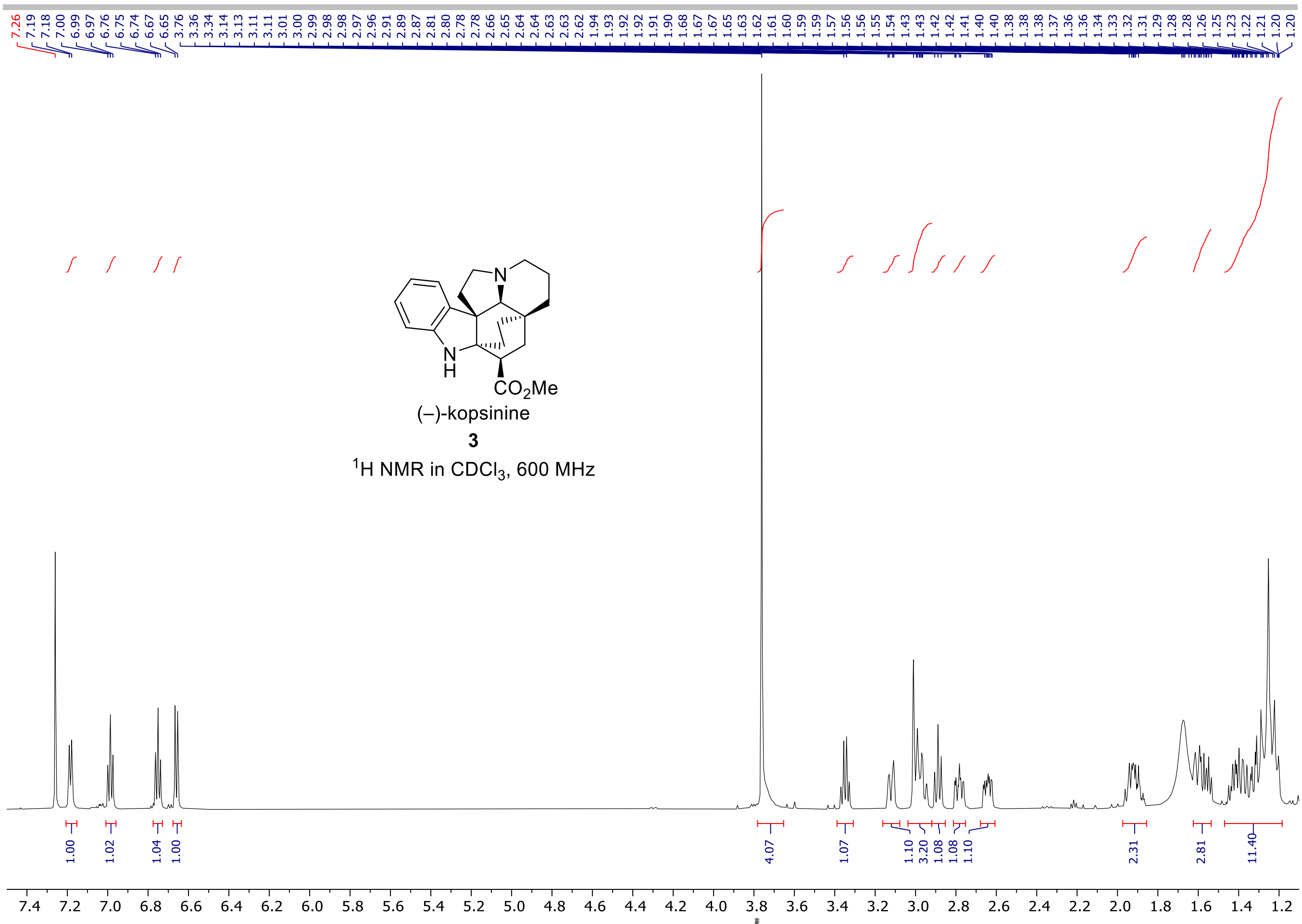
7.26 7.19 7.18 7.00 6.99 6.97 6.76 6.75 6.74 6.67 6.65 3.76 3.36 3.34 3.14 3.13 3.11 3.11 3.01 3.00 2.99 2.98 2.98 2.97 2.96 2.91 2.89 2.87 2.81 2.80 2.78 2.78 2.66 2.65 2.64 2.64 2.63 2.63 2.62 1.94 1.93 1.92 1.92 1.92 1.91 1.90 1.68 1.67 1.67 1.65 1.63 1.62 1.61 1.60 1.59 1.59 1.57 1.56 1.56 1.55 1.54 1.43 1.43 1.42 1.42 1.41 1.40 1.40 1.38 1.38 1.38 1.37 1.36 1.36 1.34 1.33 1.32 1.31 1.29 1.28 1.28 1.26 1.25 1.23 1.22 1.21 1.20 1.20
N
N
H
CO2Me
(–)-kopsinine
3
1H NMR in CDCl3, 600 MHz
1.00 1.02 1.04 1.00 4.07 1.07 1.10 3.20 1.08 1.08 1.10 2.31 2.81 11.40
7.4 7.2 7.0 6.8 6.6 6.4 6.2 6.0 5.8 5.6 5.4 5.2 5.0 4.8 4.6 4.4 4.2 4.0 3.8 3.6 3.4 3.2 3.0 2.8 2.6 2.4 2.2 2.0 1.8 1.6 1.4 1.2

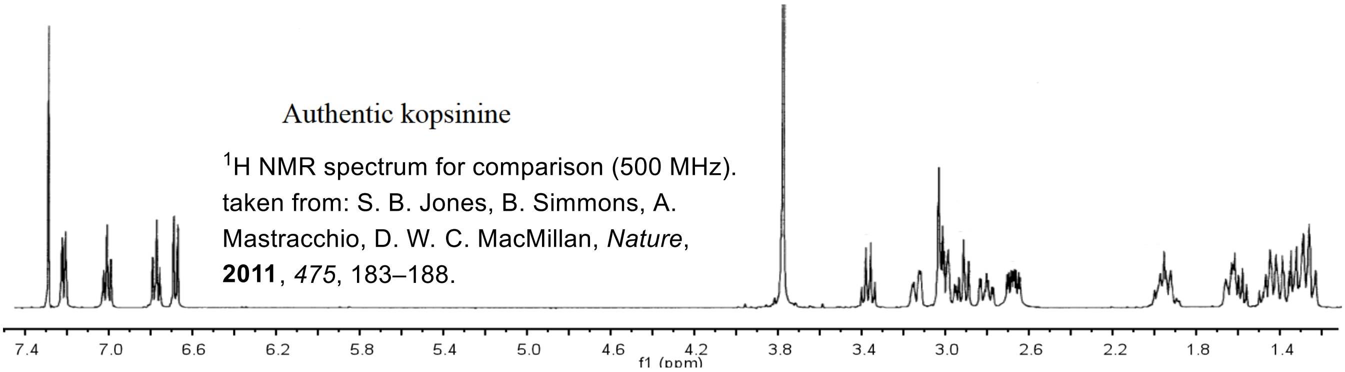
Authentic kopsinine
1H NMR spectrum for comparison (500 MHz).
taken from: S. B. Jones, B. Simmons, A. Mastracchio, D. W. C. MacMillan, Nature, 2011, 475, 183–188.
7.4 7.0 6.6 6.2 5.8 5.4 5.0 4.6 4.2 3.8 3.4 3.0 2.6 2.2 1.8 1.4
f1 (ppm)

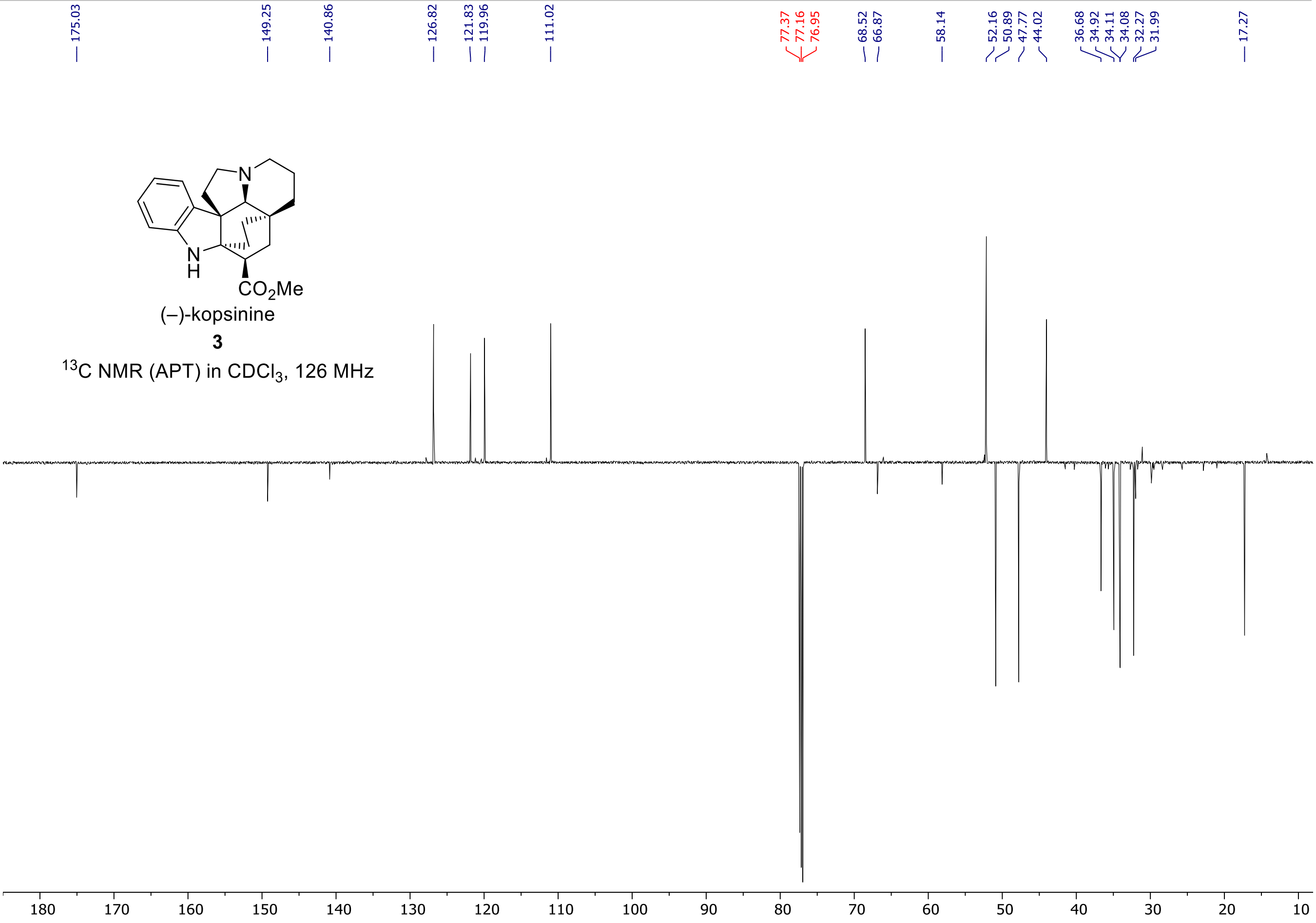
175.03
149.25
140.86
126.82
121.83
119.96
111.02
77.37
77.16
76.95
68.52
66.87
58.14
52.16
50.89
47.77
44.02
36.68
34.92
34.11
34.08
32.27
31.99
17.27
N
N
H
CO_2Me
(–)-kopsinine
3
^{13}C NMR (APT) in $CDCl_3$, 126 MHz
180 170 160 150 140 130 120 110 100 90 80 70 60 50 40 30 20 10

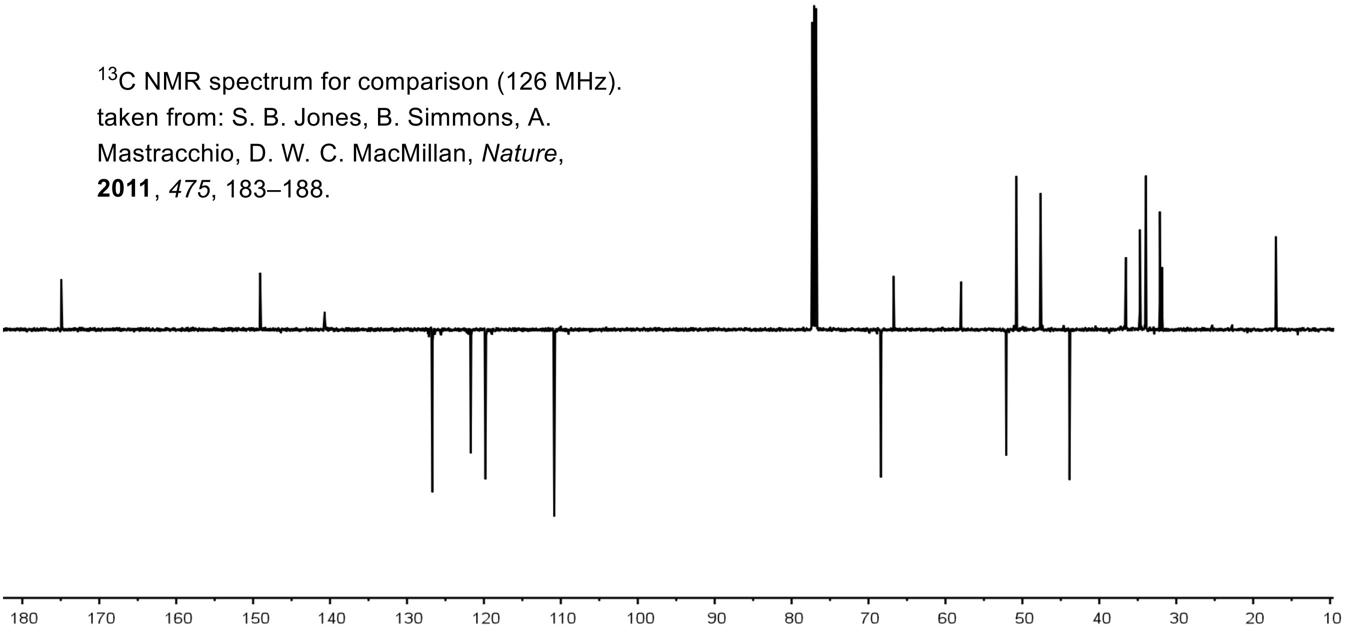
^{13}C NMR spectrum for comparison (126 MHz).
taken from: S. B. Jones, B. Simmons, A. Mastracchio, D. W. C. MacMillan, *Nature*, **2011**, *475*, 183–188.
180 170 160 150 140 130 120 110 100 90 80 70 60 50 40 30 20 10

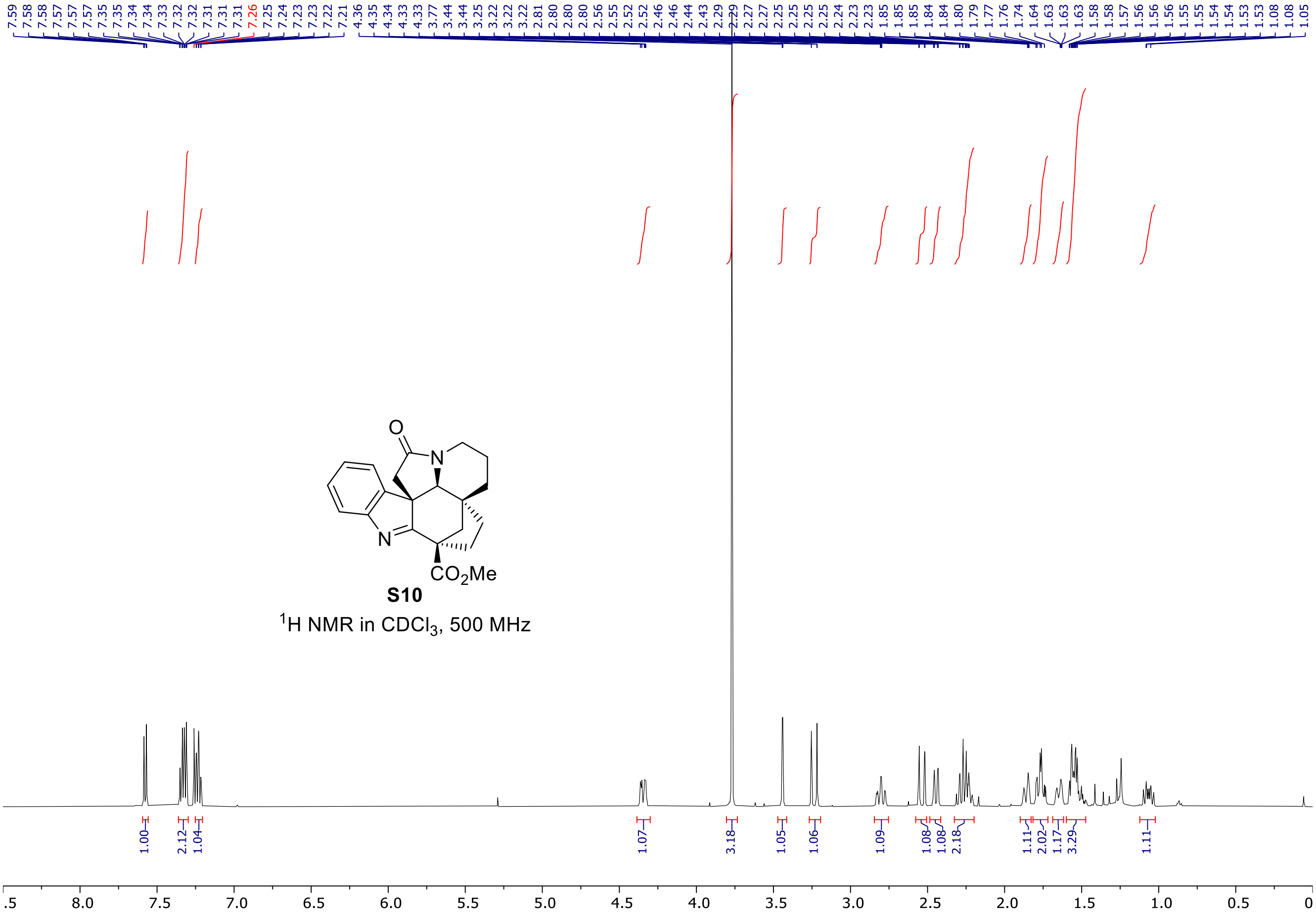
7.59 7.58 7.58 7.57 7.57 7.35 7.35 7.34 7.34 7.33 7.32 7.32 7.31 7.31 7.31 7.26 7.25 7.24 7.23 7.23 7.22 7.21 4.36 4.35 4.34 4.33 4.33 3.77 3.44 3.44 3.25 3.22 3.22 3.22 2.81 2.80 2.80 2.80 2.56 2.55 2.52 2.52 2.46 2.44 2.43 2.29 2.29 2.27 2.25 2.25 2.25 2.25 2.24 2.23 2.23 1.85 1.85 1.85 1.84 1.84 1.80 1.79 1.77 1.76 1.74 1.64 1.63 1.63 1.63 1.58 1.58 1.57 1.56 1.56 1.56 1.55 1.55 1.54 1.54 1.53 1.53 1.08 1.08 1.05
O
N
N
CO2Me
S10
1H NMR in CDCl3, 500 MHz
1.00 2.12 1.04 1.07 3.18 1.05 1.06 1.09 1.08 1.08 2.18 1.11 2.02 1.17 3.29 1.11
.5 8.0 7.5 7.0 6.5 6.0 5.5 5.0 4.5 4.0 3.5 3.0 2.5 2.0 1.5 1.0 0.5 0

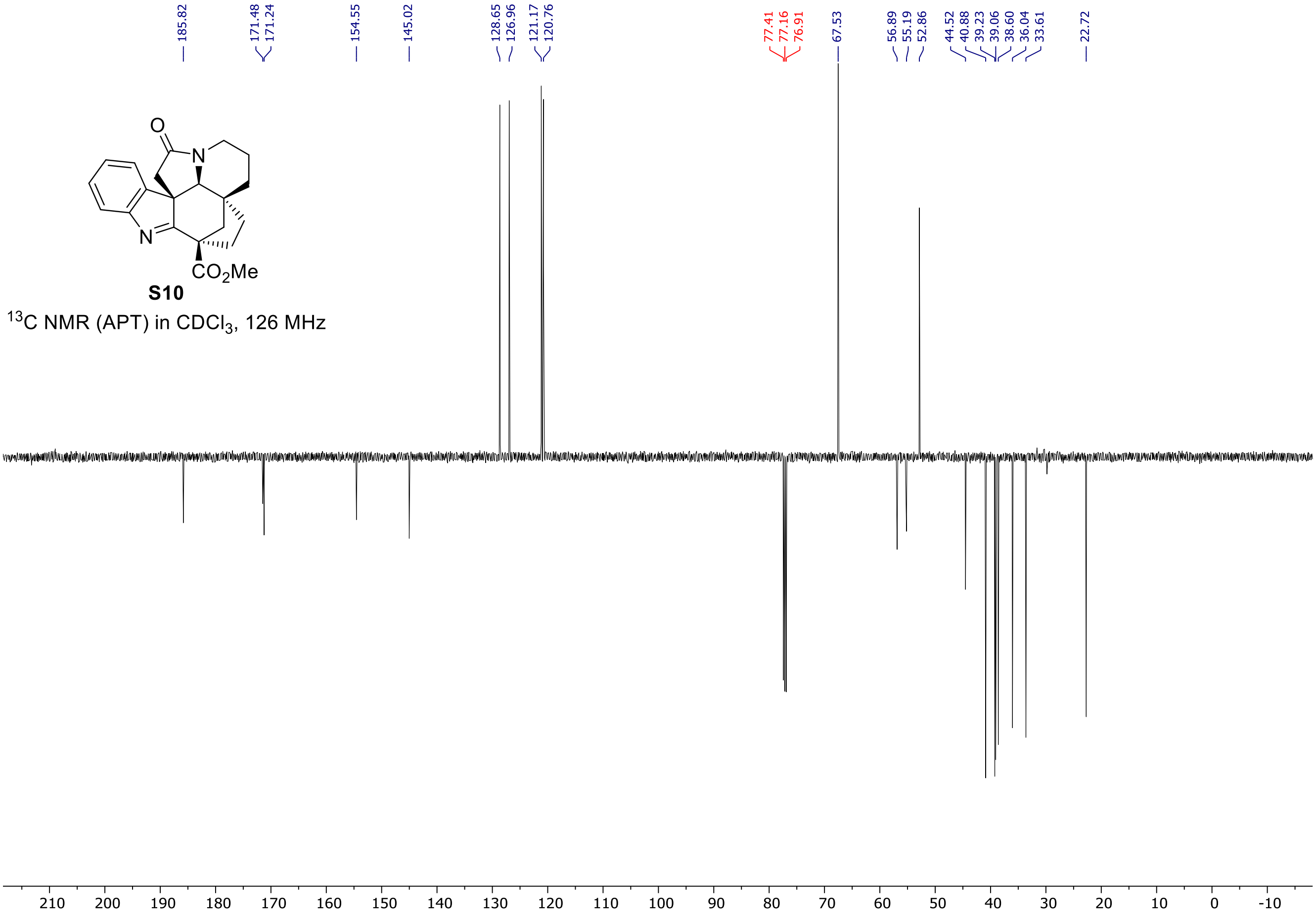
185.82 171.48 171.24 154.55 145.02 128.65 126.96 121.17 120.76 77.41 77.16 76.91 67.53 56.89 55.19 52.86 44.52 40.88 39.23 39.06 38.60 36.04 33.61 22.72
O
N
N
CO2Me
S10
13C NMR (APT) in CDCl3, 126 MHz
210 200 190 180 170 160 150 140 130 120 110 100 90 80 70 60 50 40 30 20 10 0 -10

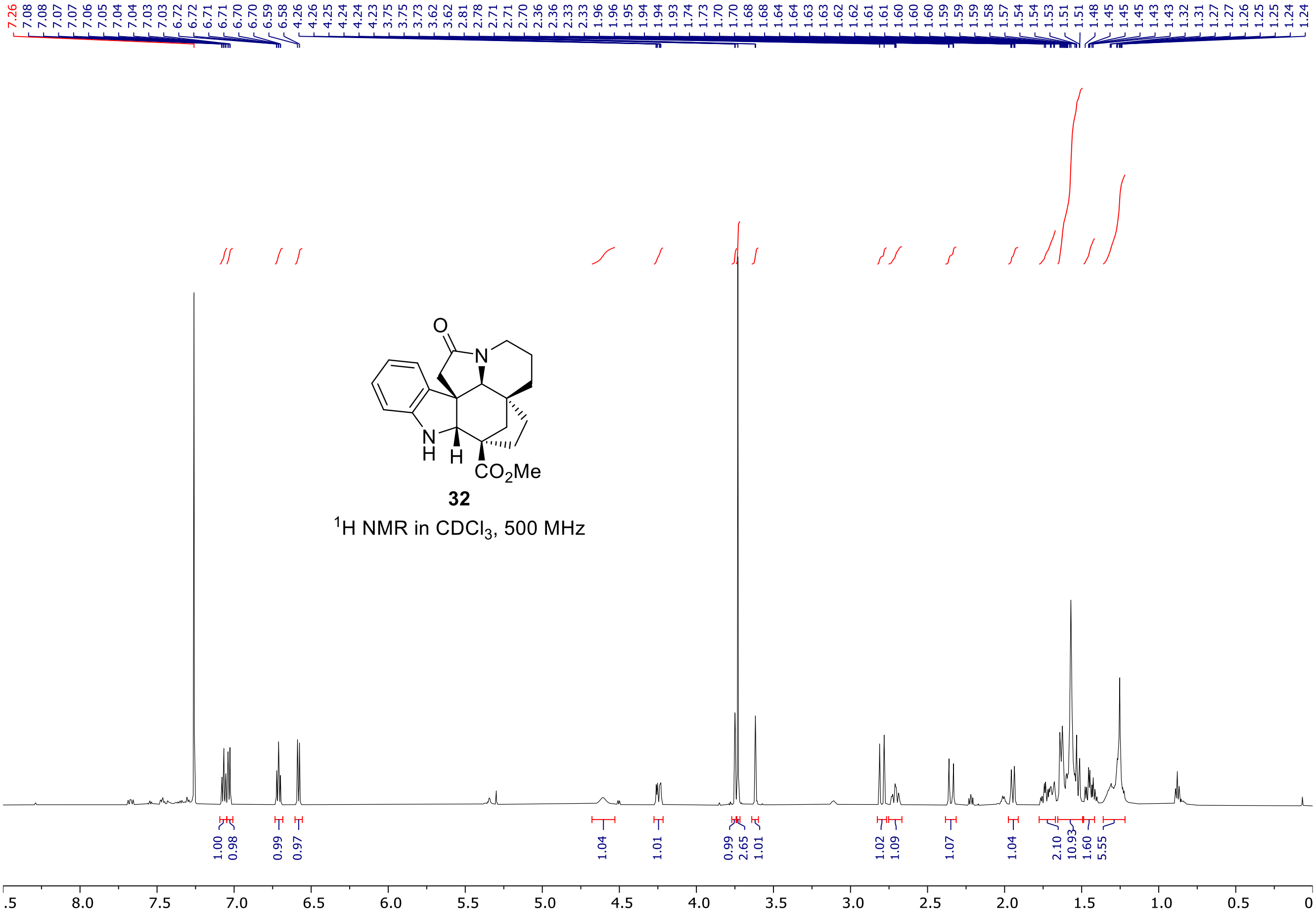
O
N
H
H
CO_2Me
32
^1H NMR in $CDCl_3$, 500 MHz

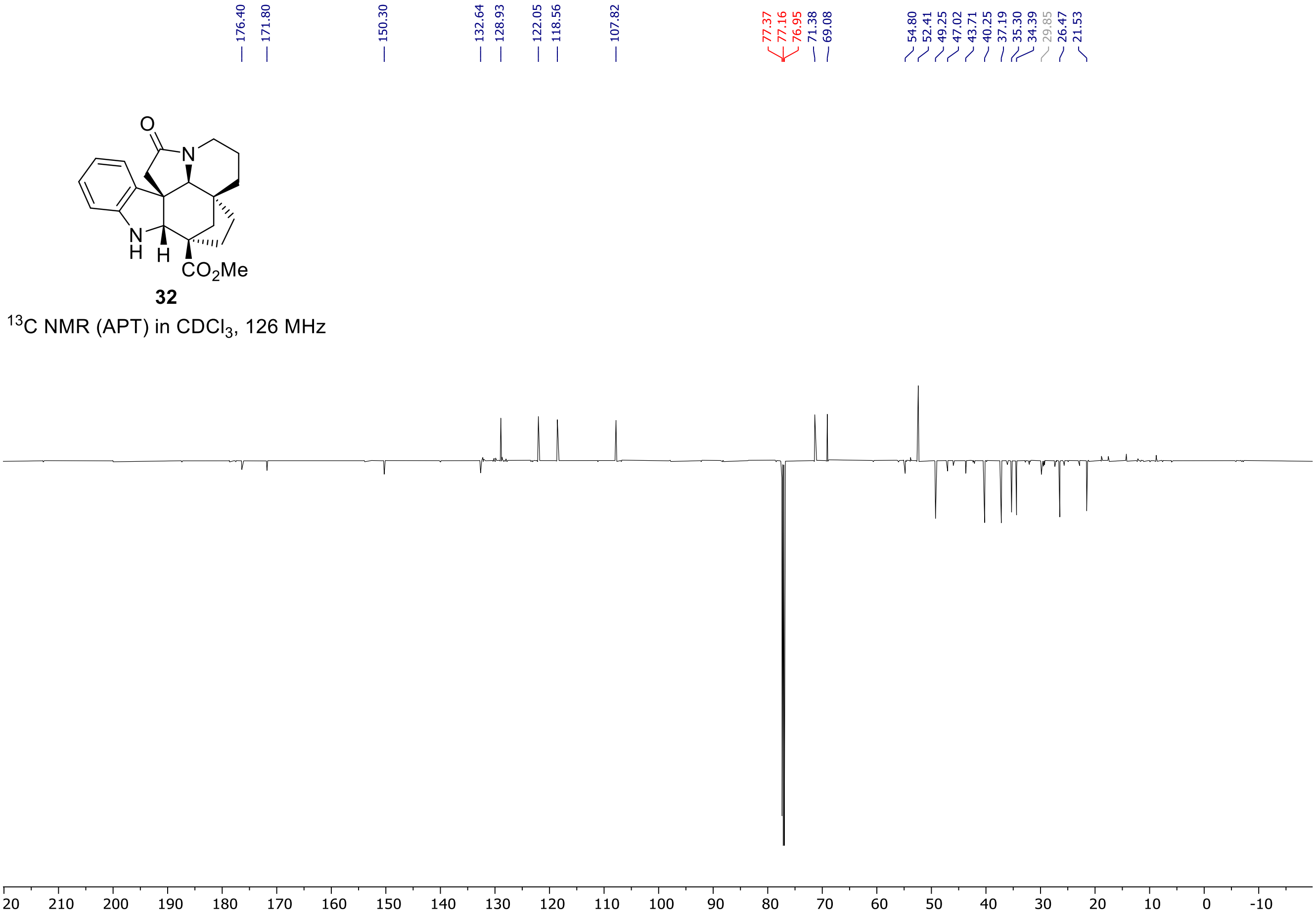
176.40
171.80
150.30
132.64
128.93
122.05
118.56
107.82
77.37
77.16
76.95
71.38
69.08
54.80
52.41
49.25
47.02
43.71
40.25
37.19
35.30
34.39
29.85
26.47
21.53
O
N
H
H
CO_2Me
32
^{13}C NMR (APT) in $CDCl_3$, 126 MHz

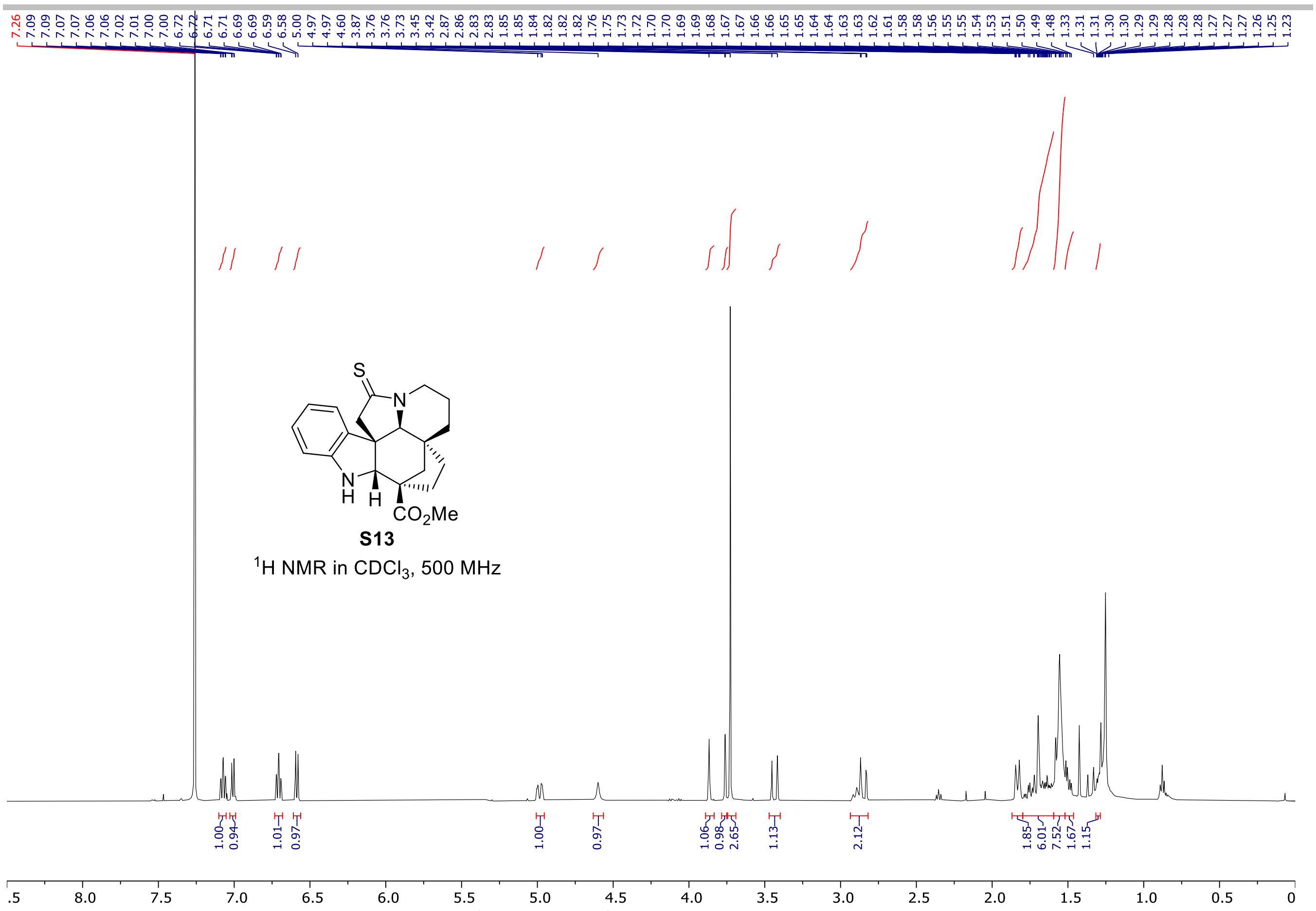
S13
^{1}H NMR in CDCl$_3$, 500 MHz

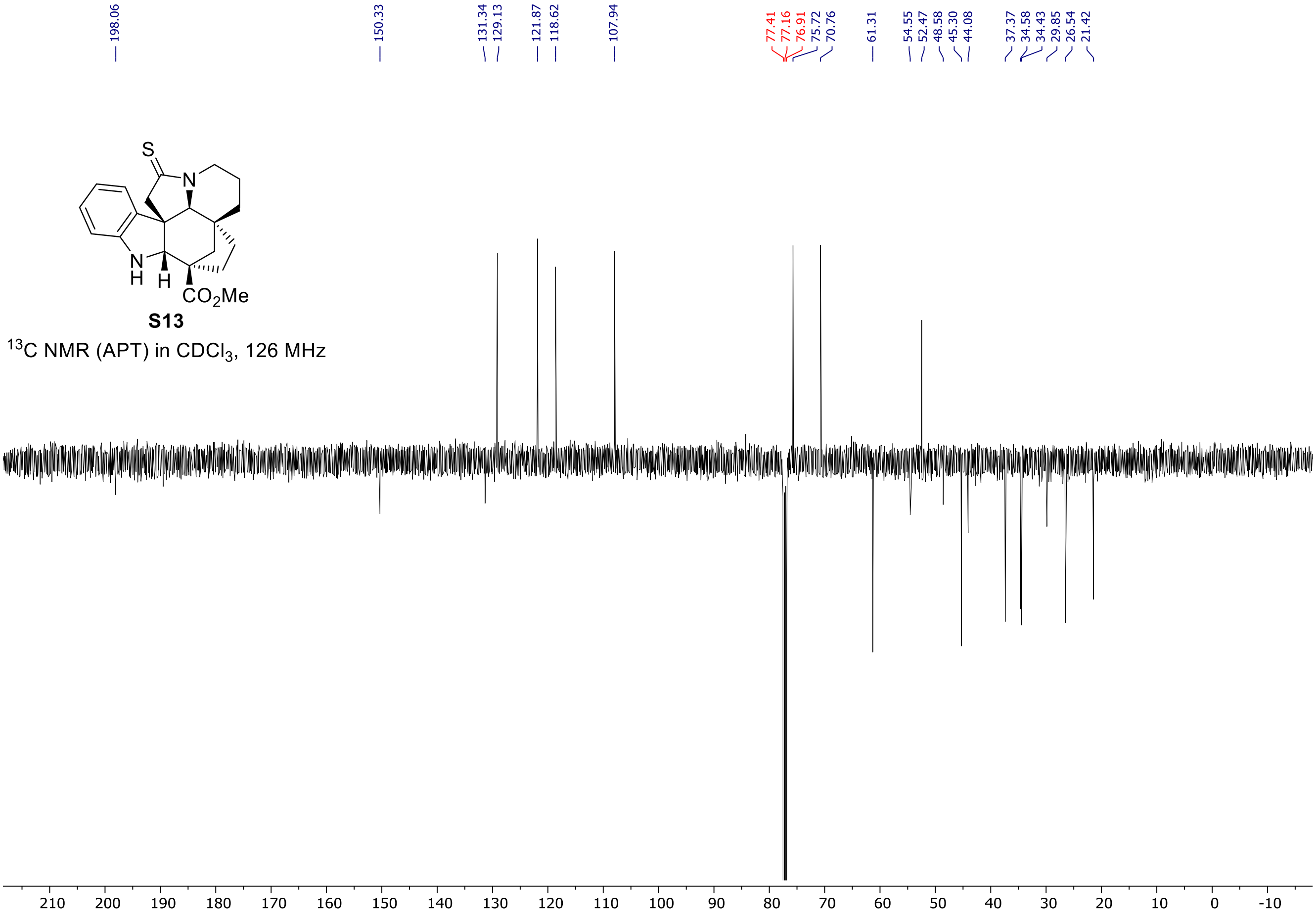
S13
^{13}C NMR (APT) in CDCl$_3$, 126 MHz

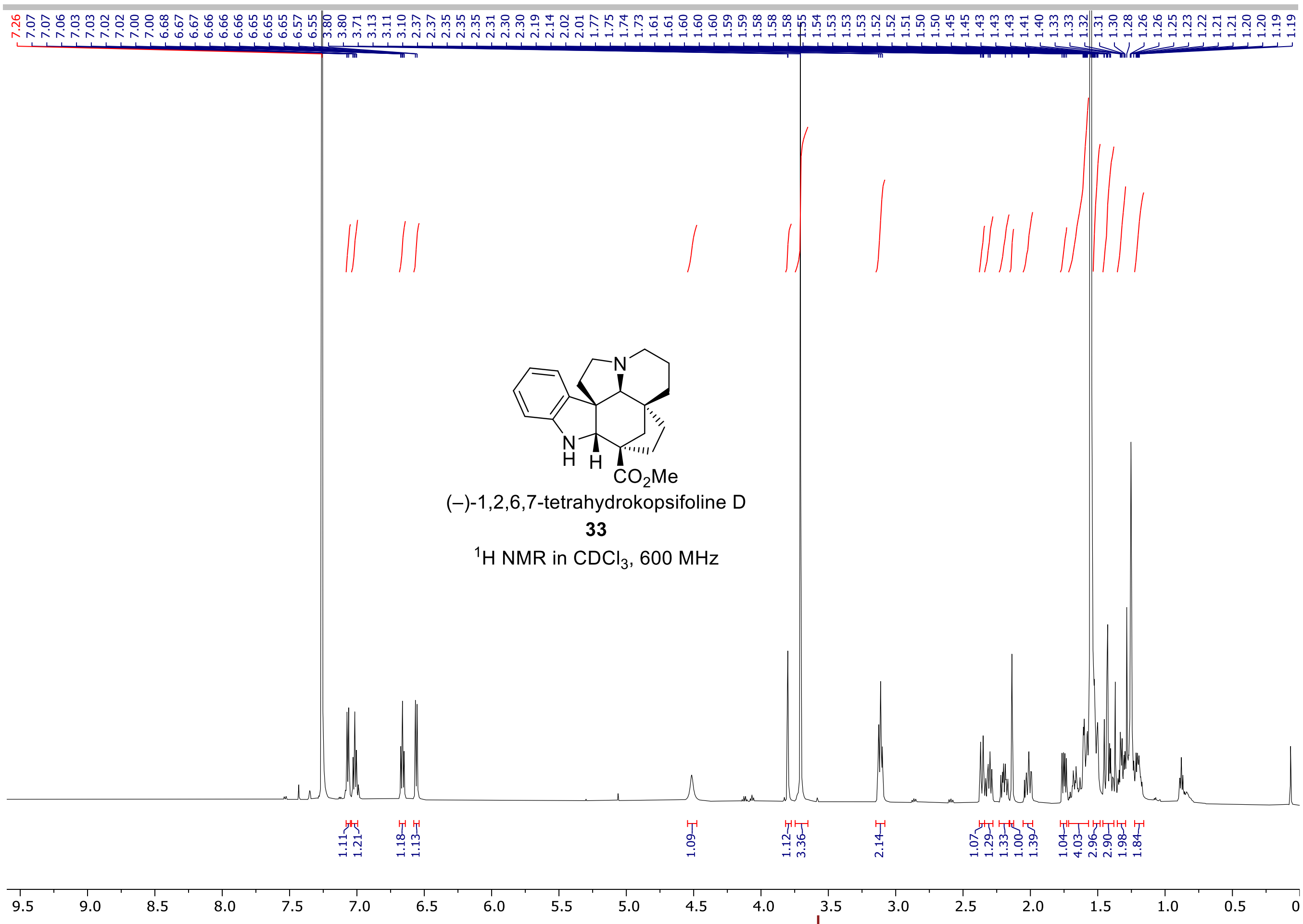

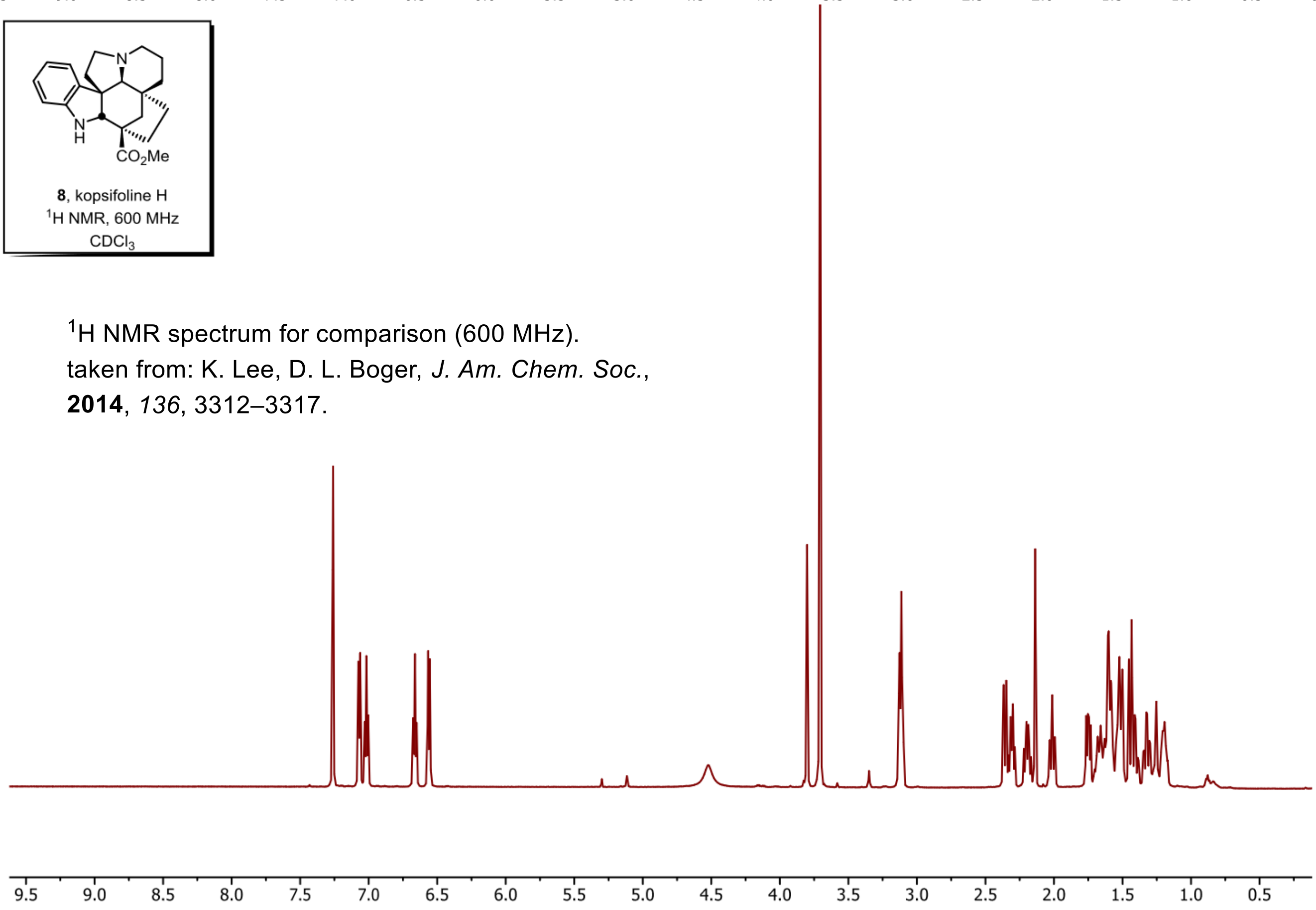

^{1}H NMR spectrum for comparison (600 MHz).
taken from: K. Lee, D. L. Boger, *J. Am. Chem. Soc.*, **2014**, *136*, 3312–3317.

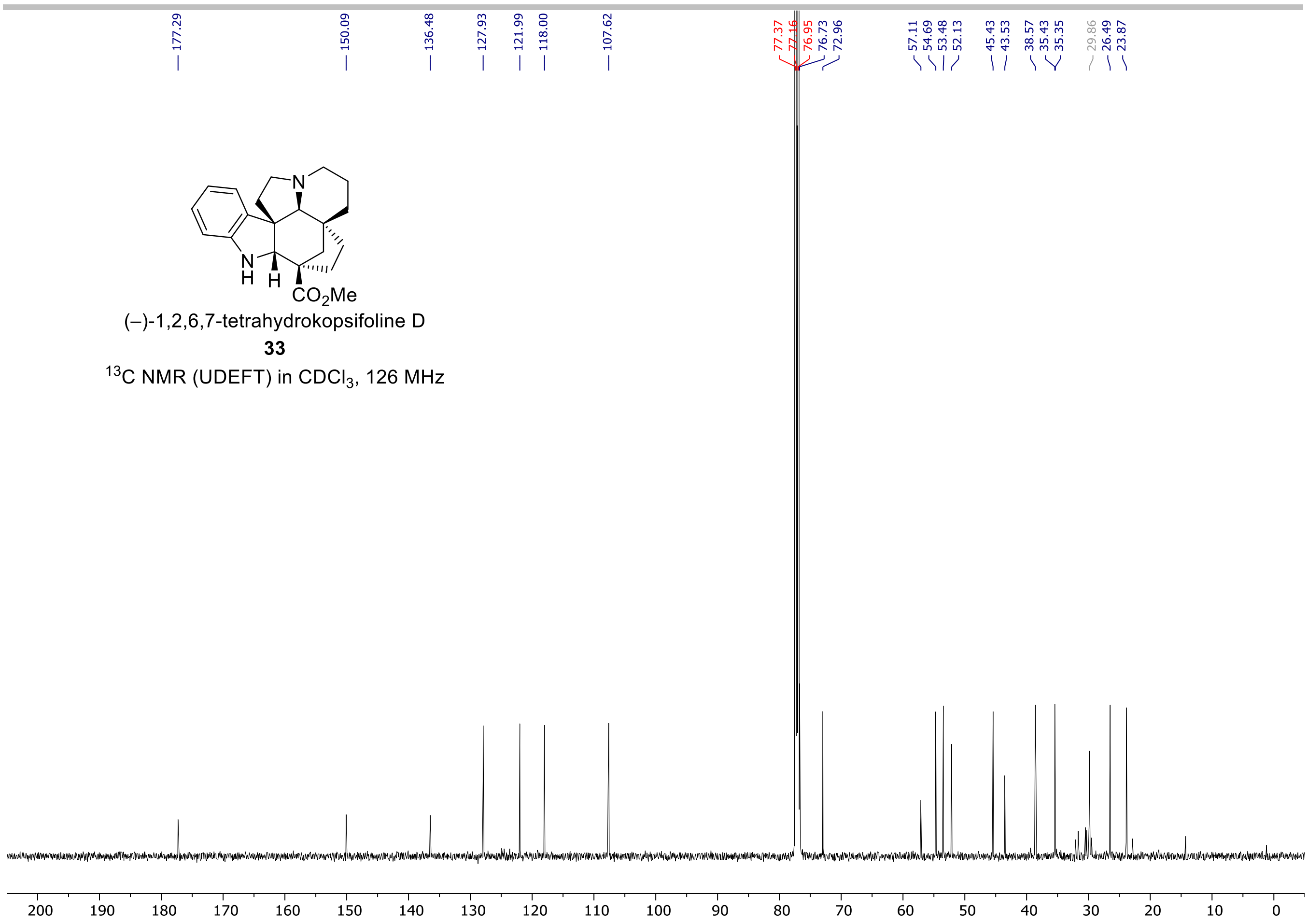
177.29
150.09
136.48
127.93
121.99
118.00
107.62
77.37
77.16
76.95
76.73
72.96
57.11
54.69
53.48
52.13
45.43
43.53
38.57
35.43
35.35
29.86
26.49
23.87
N
NH H
CO_2Me
(–)-1,2,6,7-tetrahydrokopsifoline D
33
^{13}C NMR (UDEFT) in $CDCl_3$, 126 MHz
200 190 180 170 160 150 140 130 120 110 100 90 80 70 60 50 40 30 20 10 0

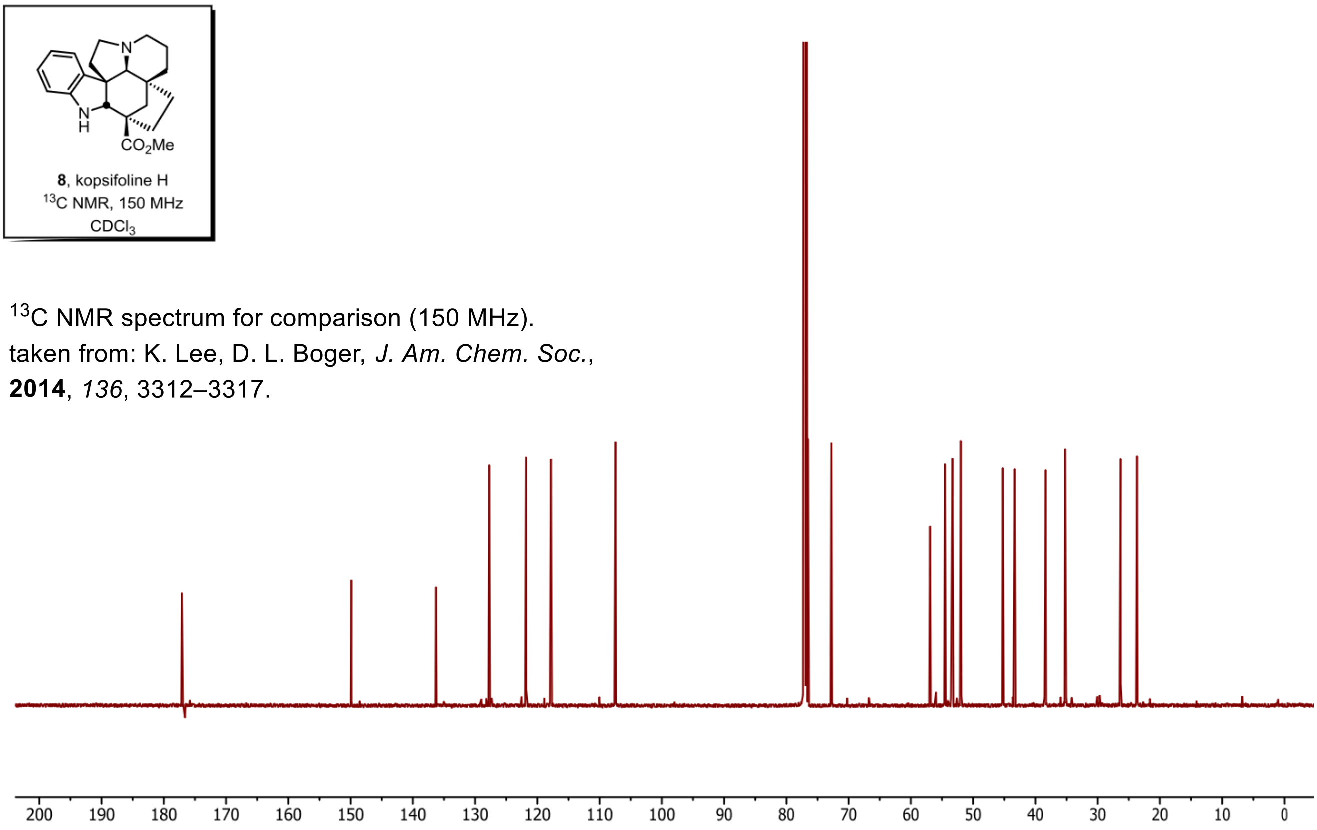
N
NH
CO_2Me
8, kopsifoline H
^{13}C NMR, 150 MHz
$CDCl_3$
^{13}C NMR spectrum for comparison (150 MHz).
taken from: K. Lee, D. L. Boger, J. Am. Chem. Soc., 2014, 136, 3312–3317.
200 190 180 170 160 150 140 130 120 110 100 90 80 70 60 50 40 30 20 10 0

Chiral SFC traces of 17, optimization of enantioselective decarboxlative Tsuji-Trost

PDA Chromatogram(MTN04rac6_001.lcd)

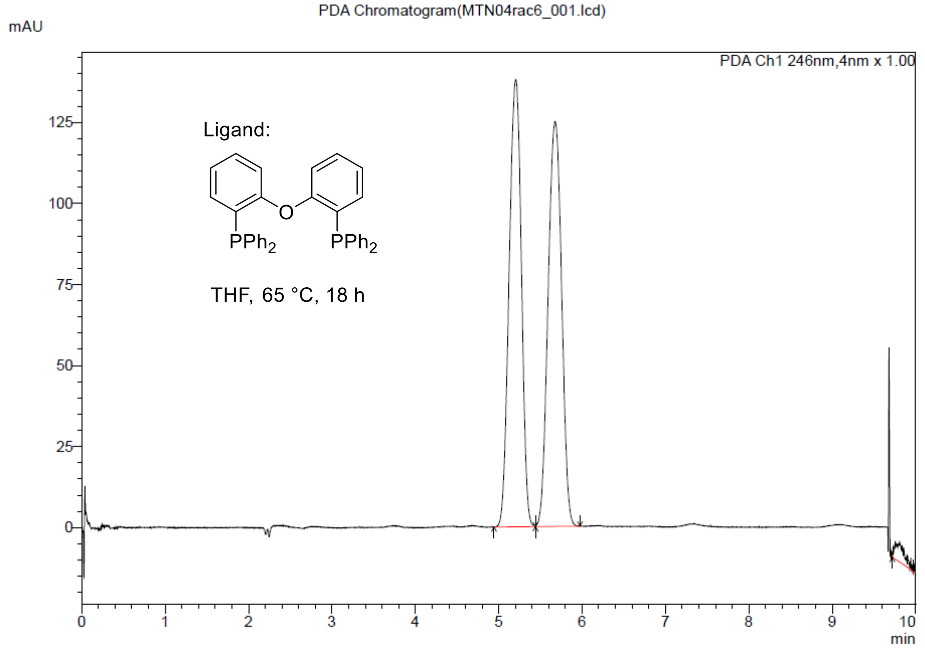

PDA Peak Table(MTN04rac6_001.lcd)

PDA Ch1 246nm

Peak#	Ret. Time	Area	Area%
1	5.204	1410200	49.264
2	5.676	1406222	49.125
3	9.757	46103	1.611
Total		2862525	100.000

PDA Chromatogram(MTN04B_002.lcd)

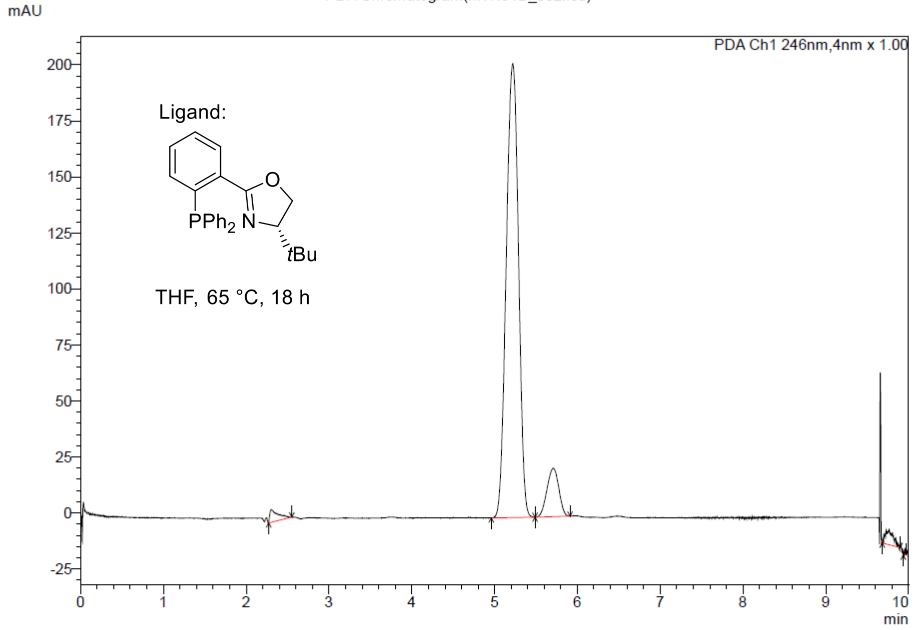

PDA Peak Table(MTN04B_002.lcd)

PDA Ch1 246nm

Peak#	Ret. Time	Area	Area%
1	2.305	39703	1.668
2	5.219	2080355	87.408
3	5.708	214516	9.013
4	9.764	43807	1.841
5	9.956	1662	0.070
Total		2380043	100.000

PDA Chromatogram(MTN04P_005.lcd)

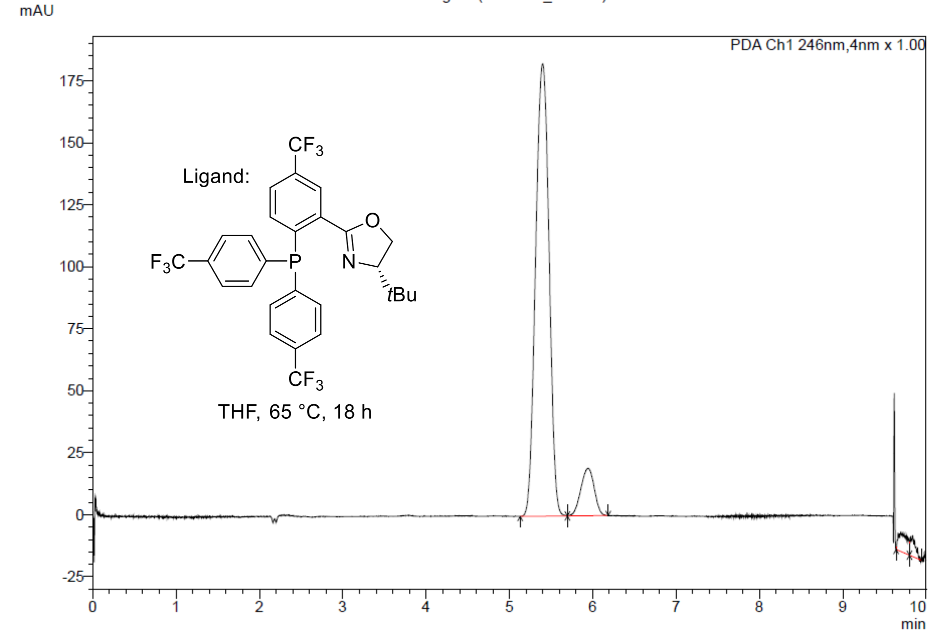

PDA Peak Table(MTN04P_005.lcd)

PDA Ch1 246nm

Peak#	Ret. Time	Area	Area%
1	5.398	2046543	86.935
2	5.942	214562	9.114
3	9.695	59216	2.515
4	9.816	33797	1.436
Total		2354118	100.000

PDA Chromatogram(MTN04N_003.lcd)

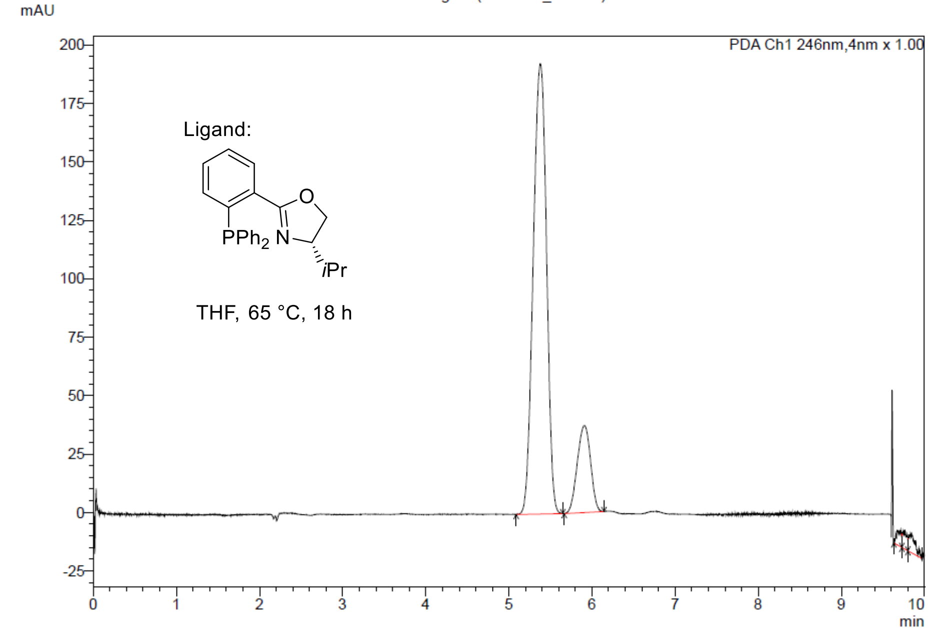

PDA Peak Table(MTN04N_003.lcd)

PDA Ch1 246nm

Peak#	Ret. Time	Area	Area%
1	5.378	2140593	80.654
2	5.907	414209	15.607
3	9.680	26572	1.001
4	9.760	28033	1.056
5	9.832	44647	1.682
Total		2654054	100.000

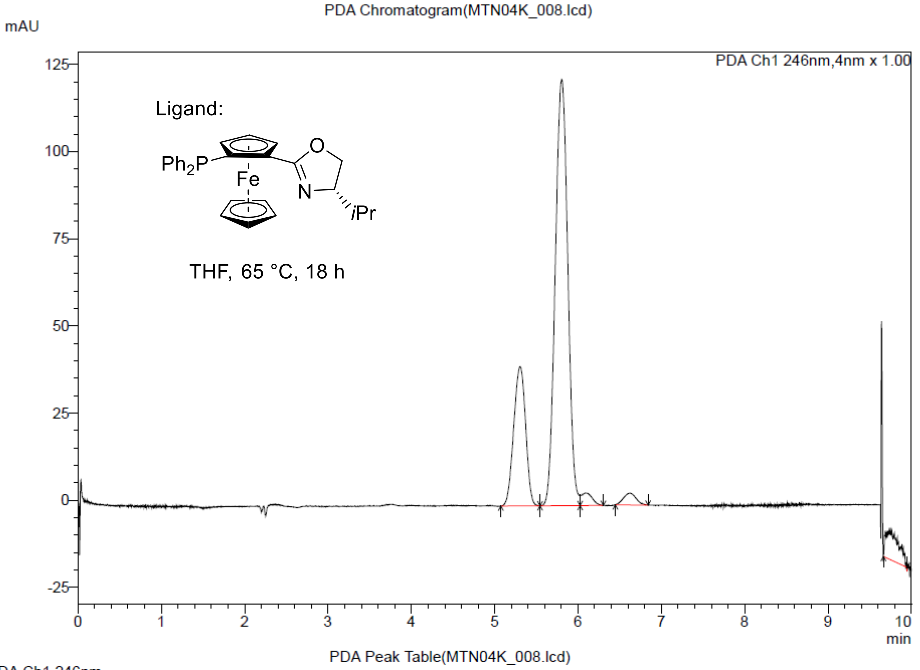

PDA Peak Table(MTN04K_008.lcd)

PDA Ch1 246nm

Peak#	Ret. Time	Area	Area%
1	5.304	397339	21.661
2	5.804	1276541	69.589
3	6.120	32152	1.753
4	6.623	35487	1.935
5	9.735	92874	5.063
Total		1834392	100.000

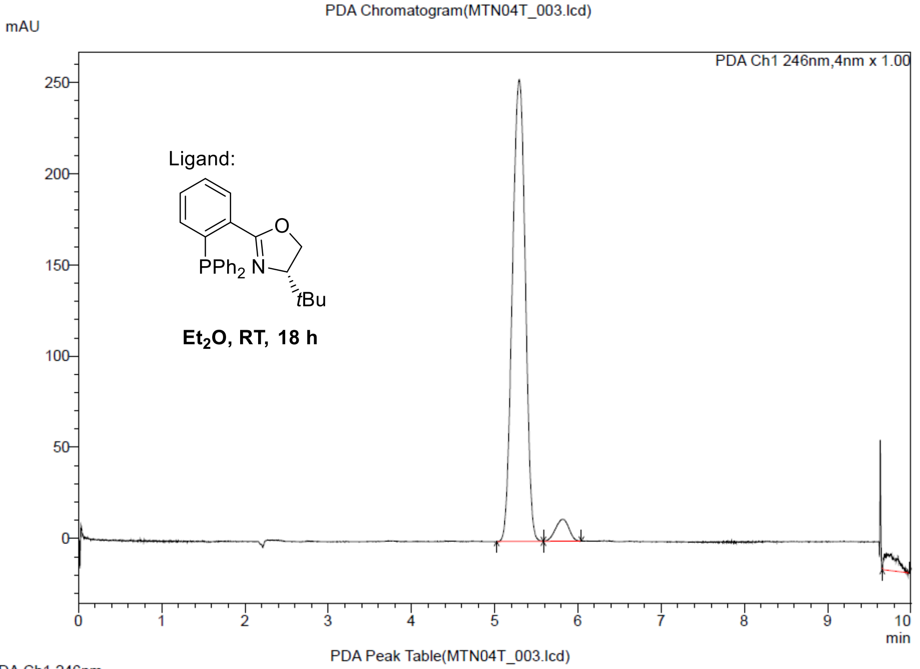

PDA Peak Table(MTN04T_003.lcd)

PDA Ch1 246nm

Peak#	Ret. Time	Area	Area%
1	5.295	2726915	92.093
2	5.816	133948	4.524
3	9.723	100197	3.384
Total		2961061	100.000

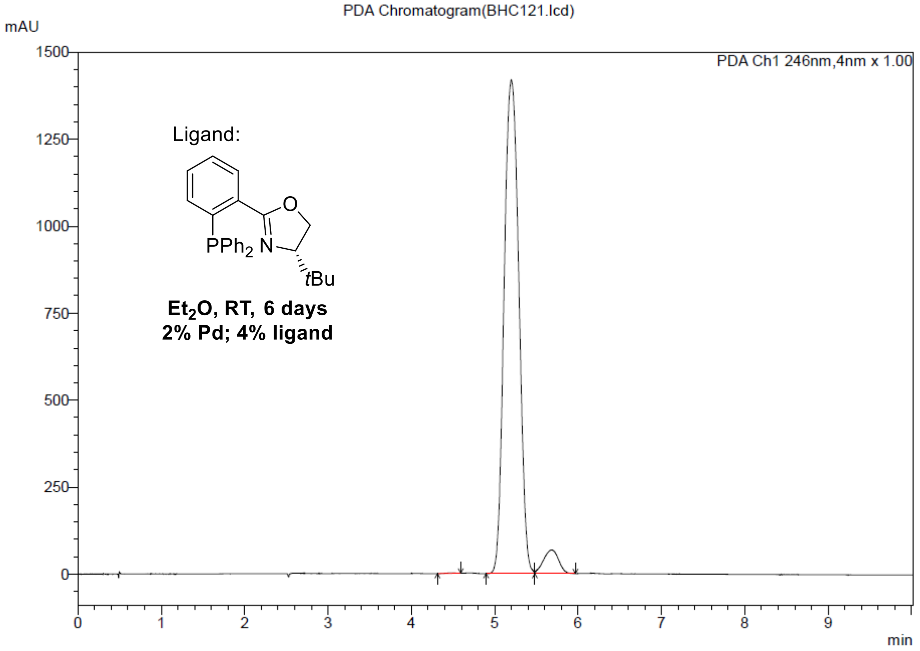

PDA Peak Table(BHC121.lcd)

PDA Ch1 246nm

Peak#	Ret. Time	Area	Area%
1	4.490	11827	0.065
2	5.199	17391234	95.267
3	5.683	852228	4.668
Total		18255289	100.000

Enantiomeric enrichment by crystallization of compound 27

PDA Chromatogram(BHC052rac_c9.lcd)

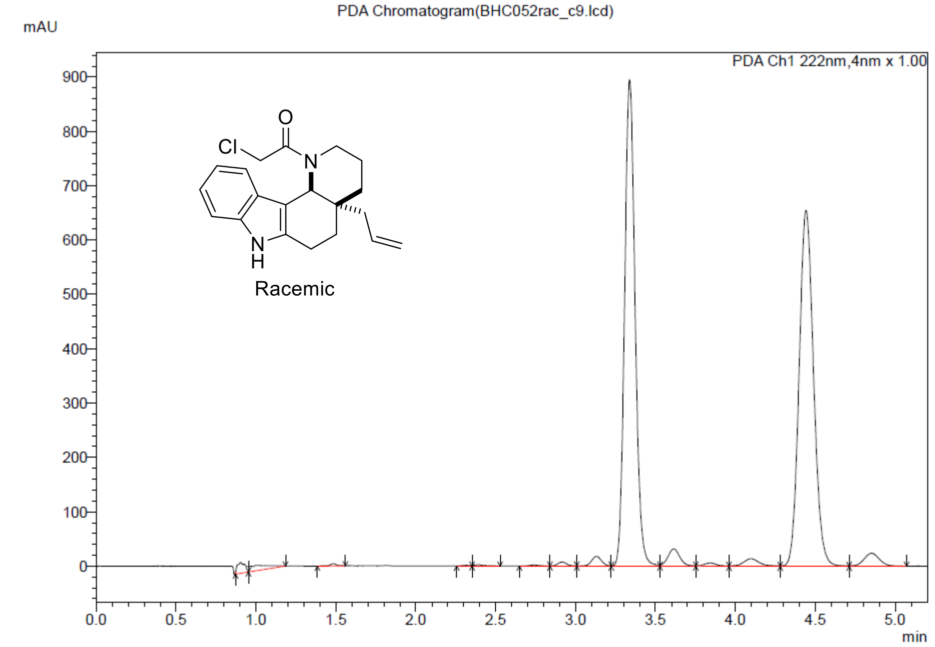

PDA Peak Table(BHC052rac_c9.lcd)

PDA Ch1 222nm

Peak#	Ret. Time	Area	Area%
1	0.904	70868	0.792
2	1.005	81125	0.907
3	1.485	12495	0.140
4	2.312	5519	0.062
5	2.389	10253	0.115
6	2.738	8960	0.100
7	2.915	29838	0.333
8	3.130	79751	0.891
9	3.338	4088708	45.691
10	3.614	166134	1.857
11	3.843	31618	0.353
12	4.097	93529	1.045
13	4.442	4108367	45.911
14	4.853	161424	1.804
Total		8948589	100.000

PDA Chromatogram(BHC123p.lcd)

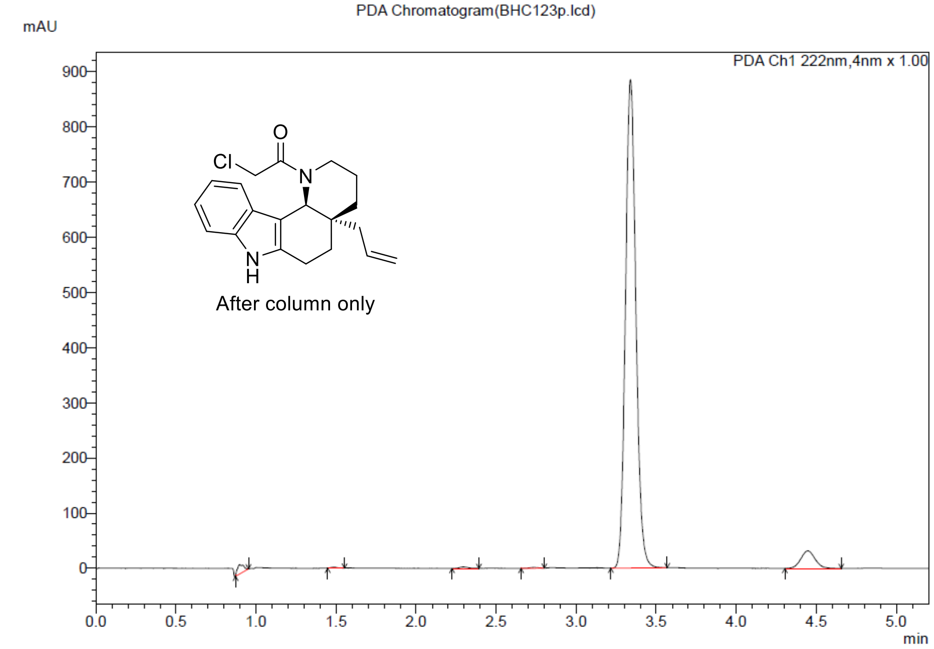

PDA Peak Table(BHC123p.lcd)

PDA Ch1 222nm

Peak#	Ret. Time	Area	Area%
1	0.900	44928	1.053
2	1.487	4490	0.105
3	2.295	8841	0.207
4	2.738	4388	0.103
5	3.339	4007859	93.952
6	4.447	195352	4.579
Total		4265859	100.000

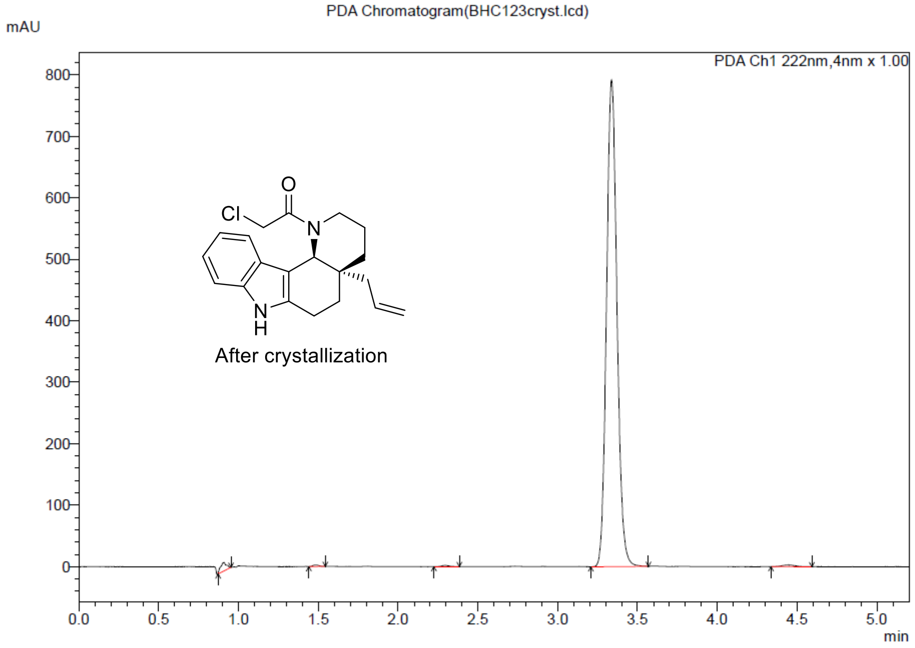

PDA Peak Table(BHC123cryst.lcd)

PDA Ch1 222nm

Peak#	Ret. Time	Area	Area%
1	0.907	37261	1.015
2	1.485	6002	0.163
3	2.294	5848	0.159
4	3.337	3606688	98.233
5	4.446	15764	0.429
Total		3671563	100.000

Crystallographic data for 27

A block-like specimen of $C_{20}H_{23}ClN_2O$, approximate dimensions 0.300 mm x 0.200 mm x 0.150 mm, was used for the X-ray crystallographic analysis. The X-ray intensity data were measured (λ = 1.54178 Å).

Table S3: Data collection details for 210714_BHC129_Cryst2.

Axis	dx/mm	2θ/°	ω/°	φ/°	χ/°	Width/°	Frames	Time/s	Wavelength/Å	Voltage/kV	Current/mA	Temperature/K
Phi	50.107	95.00	95.31	0.00	-35.00	0.50	720	2.00	1.54184	50	0.5	100

A total of 720 frames were collected. The total exposure time was 0.40 hours. The frames were integrated with the Bruker SAINT software package using a narrow-frame algorithm. The integration of the data using an orthorhombic unit cell yielded a total of 26169 reflections to a maximum θ angle of 68.27° (0.83 Å resolution), of which 3152 were independent (average redundancy 8.302, completeness = 98.6%, R_{int} = 2.51%, R_{sig} = 2.05%) and 3095 (98.19%) were greater than $2\sigma(F^2)$. The final cell constants of a = 8.7525(2) Å, b = 11.1428(2) Å, c = 17.9882(4) Å, volume = 1754.34(6) $Å^3$, are based upon the refinement of the XYZ-centroids of 9932 reflections above 20 σ(I) with 9.335° < 2θ < 136.5°. Data were corrected for absorption effects using the Multi-Scan method (SADABS). The ratio of minimum to maximum apparent transmission was 0.605.

The structure was solved and refined using the Bruker SHELXTL Software Package, using the space group P 21 21 21, with Z = 4 for the formula unit, $C_{20}H_{23}ClN_2O$. The final anisotropic full-matrix least-squares refinement on F^2 with 227 variables converged at R1 = 2.26%, for the observed data and wR2 = 5.80% for all data. The goodness-of-fit was 1.018. The largest peak in the final difference electron density synthesis was 0.157 $e^-/Å^3$ and the largest hole was -0.160 $e^-/Å^3$ with an RMS deviation of 0.032 $e^-/Å^3$. On the basis of the final model, the calculated density was 1.298 g/cm^3 and F(000), 728 e^-.

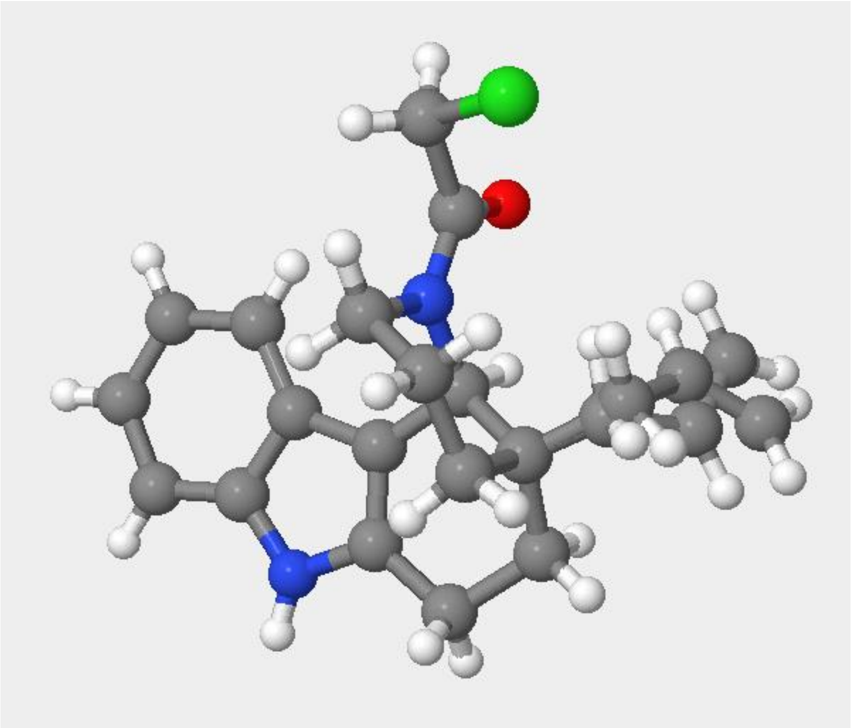

Table S4. Crystal and refinement data for 210714_BHC129_Cryst2.

**Identification code**	210714_BHC129_Cryst2	
**Chemical formula**	$C_{20}H_{23}ClN_2O$	
**Formula weight**	342.85 g/mol	
**Temperature**	100(2) K	
**Wavelength**	1.54178 Å	
**Crystal size**	0.300 x 0.200 x 0.150 mm	
**Crystal system**	orthorhombic	
**Space group**	P 21 21 21	
**Unit cell dimensions**	a = 8.7525(2) Å	α = 90°
	b = 11.1428(2) Å	β = 90°
	c = 17.9882(4) Å	γ = 90°
**Volume**	1754.34(6) $Å^3$	
**Z**	4	
**Density (calculated)**	1.298 g/cm^3	
**Absorption coefficient**	1.984 mm^{-1}	
**F(000)**	728	
**Theta range for data collection**	4.67 to 68.27°	
**Index ranges**	-10<=h<=10, -12<=k<=12, -21<=l<=19	
**Reflections collected**	26169	
**Independent reflections**	3152 [R(int) = 0.0251]	
**Coverage of independent reflections**	98.6%	
**Absorption correction**	Multi-Scan	
**Structure solution technique**	direct methods	
**Structure solution program**	APEX3 SHELXL-2019	
**Refinement method**	Full-matrix least-squares on F^2	
**Refinement program**	SHELXL-2019/1 (Sheldrick, 2019)	
**Function minimized**	$\Sigma w(F_o^2 - F_c^2)^2$	
**Data / restraints / parameters**	3152 / 11 / 227	
**Goodness-of-fit on F2**	1.018	
**Final R indices**	3095 data; I>2σ(I)	R1 = 0.0226, wR2 = 0.0576
	all data	R1 = 0.0231, wR2 = 0.0580
**Weighting scheme**	$w=1/[\sigma^2(F_o^2)+(0.0317P)^2+0.3263P]$ where $P=(F_o^2+2F_c^2)/3$	
**Absolute structure parameter**	0.029(3)	
**Largest diff. peak and hole**	0.157 and -0.160 $eÅ^{-3}$	
**R.M.S. deviation from mean**	0.032 $eÅ^{-3}$	

Table S5. Atomic coordinates and equivalent isotropic atomic displacement parameters (Å^2) for 210714_BHC129_Cryst2.
U(eq) is defined as one third of the trace of the orthogonalized U_{ij} tensor

	x/a	y/b	z/c	U(eq)
N1	0.62211(16)	0.79997(13)	0.22738(7)	0.0217(3)
C2	0.54850(19)	0.75375(15)	0.16611(9)	0.0201(4)
C3	0.6049(2)	0.72357(16)	0.09619(10)	0.0248(4)
C5	0.3472(2)	0.66650(16)	0.06129(9)	0.0254(4)
C6	0.29084(19)	0.69706(15)	0.13059(9)	0.0212(3)
C7	0.39121(18)	0.74055(15)	0.18477(9)	0.0178(3)
C9	0.23921(17)	0.78201(15)	0.31062(8)	0.0161(3)
C8	0.37703(18)	0.77772(15)	0.26109(9)	0.0168(3)
C4	0.5026(2)	0.67920(17)	0.04430(10)	0.0277(4)
C10	0.28593(19)	0.78853(15)	0.39375(9)	0.0191(3)
C11	0.4099(2)	0.88585(16)	0.40214(9)	0.0226(4)
C12	0.55804(19)	0.85974(16)	0.35952(10)	0.0242(4)
C13	0.51886(18)	0.81277(16)	0.28426(9)	0.0194(3)
C14	0.34884(19)	0.66641(16)	0.42008(9)	0.0225(4)
C15	0.24571(19)	0.56054(16)	0.40126(9)	0.0228(4)
C16	0.20546(18)	0.56115(15)	0.31883(9)	0.0199(3)
N17	0.13832(15)	0.67712(12)	0.29813(7)	0.0167(3)
C18	0.99540(18)	0.69399(16)	0.27333(8)	0.0180(3)
C19	0.89148(19)	0.58542(16)	0.26716(9)	0.0241(4)
Cl20	0.81805(5)	0.54825(5)	0.35745(3)	0.03958(15)
O21	0.94460(13)	0.79400(11)	0.25552(6)	0.0224(3)
C31B	0.1775(10)	0.8240(4)	0.4323(10)	0.0193(4)
C32B	0.1307(5)	0.9528(4)	0.4462(5)	0.0218(4)
C33B	0.0065(6)	0.0020(6)	0.4193(6)	0.0281(5)
C31	0.1437(2)	0.82210(14)	0.44330(11)	0.0193(4)
C32	0.06311(19)	0.93723(12)	0.42205(9)	0.0218(4)
C33	0.0717(2)	0.03710(15)	0.46027(10)	0.0281(5)

Supporting references

[1] Following a modified procedure from: W. Yang, J. Cao, M. Zhang, R. Lan, L. Zhu, G. Du, S. He, C. S. Lee, *J. Org. Chem.* **2015**, *80*, 836–846. Isolation following: R. Hara, T. Furukawa, H. Kashima, H. Kusama, Y. Horiguchi, I. Kuwajima, *J. Am. Chem. Soc.* **1999**, *121*, 3072–3082.

[2] Adapted from: S. Boyce, W. Buhr, S. Burckhardt, V. A. Corden, S. M. Courtney, T. Davenport, G. Dawson, F. Duerrenberger, A. A. Ellenbroek, F. Funk, P. O. Geisser, M. P. Ridgill, M. Slack, C. J. Yarnold (Vifor [International] AG), WO 2012/110603 A1, **2012**.

[3] Inspired by: A. Béchamp, *Justus Liebigs Ann. Chem.* **1854**, *92*, 401–403.

[4] Following a modified procedure from: M. A. Toczko, C. H. Heathcock, *J. Org. Chem.* **2000**, *65*, 2642–2645.

[5] a) S. R. Crabtree, W. L. A. Chu, L. N. Mander, *Synlett* **1990**, *1990*, 169–170; b) B. Cheng, J. D. Sunderhaus, S. F. Martin, *Org. Lett.* **2010**, *12*, 3622–3625.

[6] Following a modified procedure from: P. Magnus, P. Brown, *J. Chem. Soc. Chem. Commun.* **1985**, 184–186.
